# Structural snapshots of an Al–Cu bond-mediated transformation of terminal acetylenes†

**DOI:** 10.1039/d3sc00240c

**Published:** 2023-02-15

**Authors:** Han-Ying Liu, Samuel E. Neale, Michael S. Hill, Mary F. Mahon, Claire L. McMullin

**Affiliations:** a Department of Chemistry, University of Bath Claverton Down Bath BA2 7AY UK msh27@bath.ac.uk cm2025@bath.ac.uk

## Abstract

The copper(i) alumanyl derivative, [{SiN^Dipp^}Al–Cu(NHC^iPr^)] (SiN^Dipp^ = {CH_2_SiMe_2_NDipp}_2_; Dipp = 2,6-di-isopropylphenyl; NHC^iPr^ = *N*,*N*′-di-isopropyl-4,5-dimethyl-2-ylidene), reacts in a stepwise fashion with up to three equivalents of various terminal alkynes. This reactivity results in the sequential formation of cuprous (hydrido)(alkynyl)aluminate, (alkenyl)(alkynyl)aluminate and bis(alkynyl)aluminate derivatives, examples of which have been fully characterised. The process of alkene liberation resulting from the latter reaction step constitutes a unique case of alkyne transfer semi-hydrogenation in which the C–H acidic alkyne itself acts as a source of proton, with the Cu–Al bond providing the requisite electrons to effect reduction. This reaction sequence is validated by DFT calculations, which rationalise the variable stability of the initially formed heterobimetallic hydrides.

## Abstract 2

The C–Al bond of [{SiN^Dipp^}Al–Cu(NHC^iPr^)] reacts sequentially with three equivalents of terminal alkyne to provide cuprous (hydrido)(alkynyl)aluminate, (*E-*alkenyl)(alkynyl)aluminate and bis(alkynyl)aluminate derivatives.

## Graphical abstract

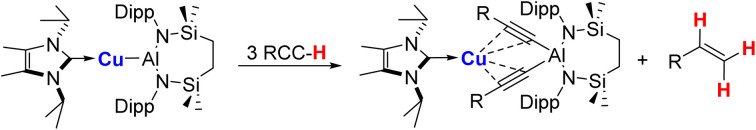

## Introduction

The transformation of alkynes provides a key reaction step in many organic syntheses.^1^ The selective reduction of the C≡C triple bond of an aryl acetylene, for example, provides a route to styrene synthesis. Although transition metal-catalysed hydrogenation can provide for the efficient synthesis of highly functionalised fine chemicals and natural products,^2^ and is well established using heterogeneous palladium (Lindlar and Lindlar-type) catalysts,^3^ over-reduction to alkane products is relatively facile and remains a particular issue for the semi-hydrogenation of terminal alkynes.^4^ Furthermore, on a smaller scale, the replacement of H_2_ by low-cost hydrogen donors (transfer hydrogenation) is attractive with regard to safety concerns and a need for more specialised gas handling equipment.^5^ The transfer semi-hydrogenation of alkynes has, thus, been achieved through the action of a variety of transition metal-based systems with formally protic^6–8^ (alcohol, water, formic acid) and hydridic^9,10^ (silane, borane or amine borane) hydrogen donors. In this regard, the employment of *N*-heterocyclic carbene adducts of Cu(i) is germane to the current study.^9*d*–*j*,10*a*,*b*^ In these cases, the commonly proposed reaction mechanism implicates the formation of a Cu(i) hydride, which inserts the alkyne substrate to yield an alkenyl intermediate. This is then released as the alkene product through reaction at the Cu–C bond with the exogenous source of hydrogen.

**Fig. 1:**
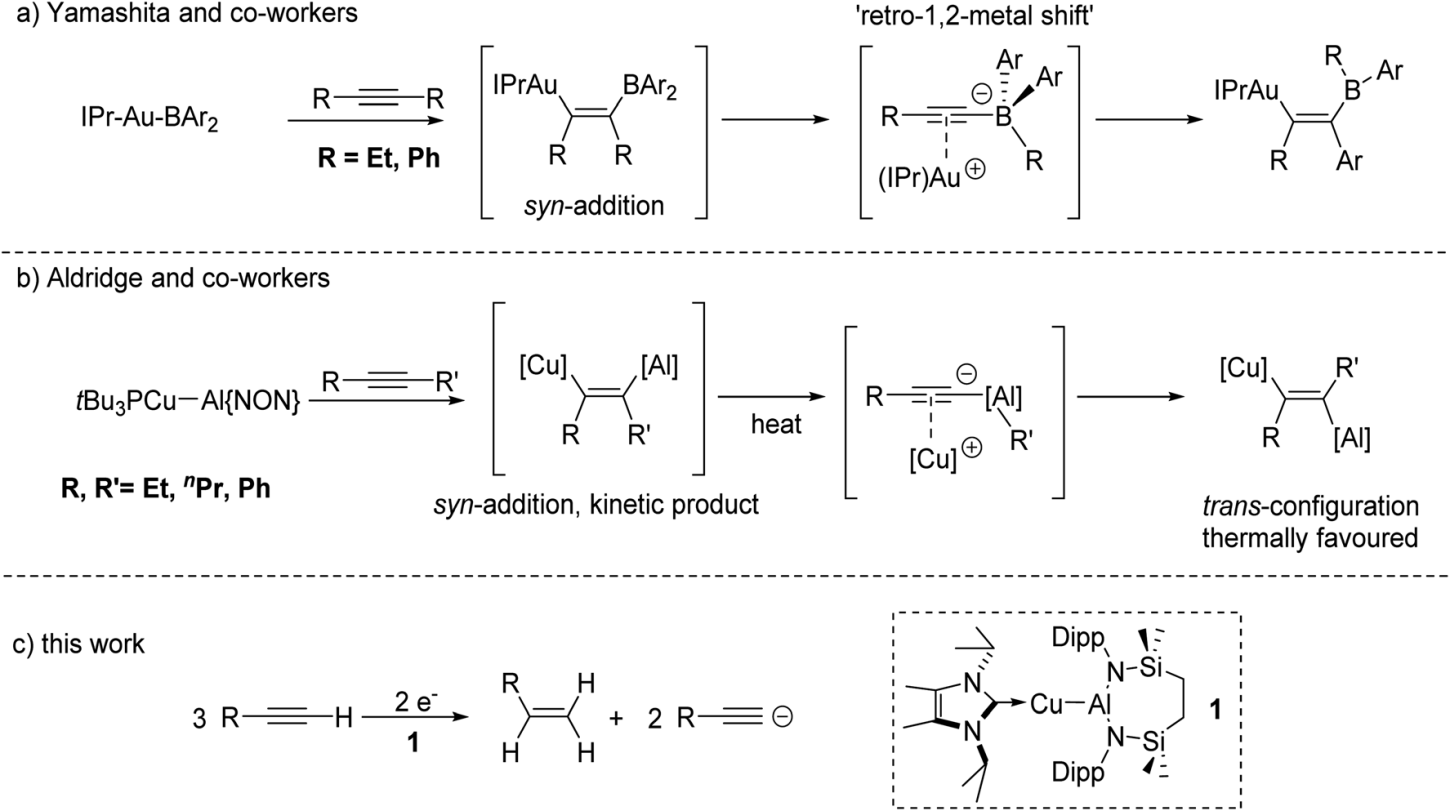
Reactivity of group 11-to-group 13 bonds towards C≡C triple bonds.

In related recent, but stoichiometric, observations of group 11 reactivity, Yamashita and co-workers have described the behaviour of a gold–boryl complex, [(IPr)AuBAr_2_] (IPr = 1,3-bis(2,6-diisopropylphenyl-imidazole-2-ylidene); Ar = *o*-tolyl), towards internal alkynes (Fig. 1a).^11^ Although this reactivity displayed some dependence on alkyne identity, a transiently formed *syn* insertion product was observed to isomerise to an alternative *syn-*disubstituted borylalkenylgold complex *via* an interchange of an initial alkyne R group and an aryl substituent of the BAr_2_ unit. Density functional theory (DFT) calculations suggested that a common intermediate in this reaction was a gold alkynyl-borate, which can facilitate a 1,2-shift of the organic substituents. In a similar vein, Aldridge and co-workers have very recently demonstrated that the phosphine-stabilised copper–alumanyl complex, [*t*-Bu_3_PCu–Al(NON)] (NON = 4,5-bis(2,6-diisopropylanilido)-2,7-di-*tert*-butyl-9,9-dimethylxanthene) undergoes addition across internal alkynes to provide a vinylcopper compound featuring a *syn* configuration of the two metal centres (Fig. 1b).^12*a*^ This initially formed species then further rearranges on heating *via* C–C bond cleavage and an inferred aluminate intermediate, before providing the corresponding *trans*-vinylcopper isomers as the thermodynamically favoured products.

The results summarised in Fig. 1a and b pertain to reactivity between unsupported Au–B and Cu–Al bonds and internal alkynes. Neither of these studies, however, addressed variations arising from the introduction of terminal alkynes to these systems. We have recently described a family of molecules bearing unsupported group 11-aluminium bonds, in which the coinage metal centres are stabilised by various neutral donors, and the aluminium atom is supported by a six-membered diamide chelate.^13^ In this contribution, therefore, we describe the reactivity arising from the copper alumanyl species, [{SiN^Dipp^}Al–Cu(NHC^iPr^)] (SiN^Dipp^ = {CH_2_SiMe_2_NDipp}_2_; Dipp = 2,6-di-isopropylphenyl; NHC^iPr^ = *N*,*N*′-di-isopropyl-4,5-dimethyl-2-ylidene), (1) and a variety of terminal alkynes. This chemistry provides an unusual alkyne-to-alkene transformation that may be viewed as an alkyne transfer semi-hydrogenation in which reduction is facilitated by the Cu–Al bond and in which the requisite protons are provided by the terminal alkyne itself (Fig. 1c).

## Results and discussion

**Scheme 1:**
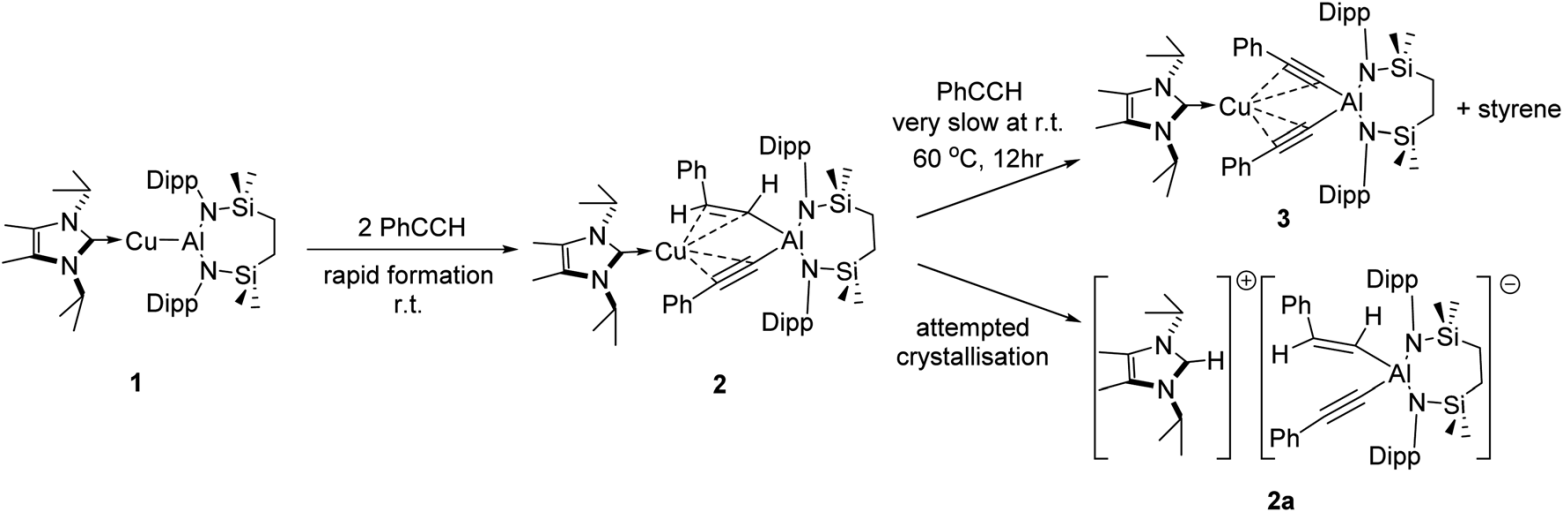
Stepwise reaction of compound 1 with phenylacetylene.

**Fig. 2:**
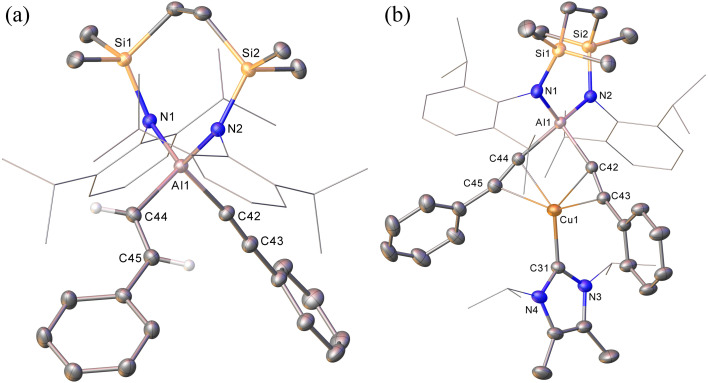
(a) Displacement ellipsoid (30% probability) plots of the anion of compound 2a and (b) of compound 3. Hydrogen atoms, apart from those attached to C44 and C45 in 2a, have been omitted while Dipp and *iso*-propyl substituents are shown as wireframe for clarity.

The NHC-stabilised copper-alumanyl (1) was treated with an equimolar quantity of phenylacetylene. Although monitoring of the reaction mixture by ^1^H NMR spectroscopy indicated complete consumption of the phenylacetylene and the formation of a single predominant new compound (2), this process consumed only half of the compound 1 utilised in the reaction. A fresh reaction mixture was, thus, prepared with compound 1 and two molar equivalents of phenylacetylene. This adjustment in stoichiometry enabled the quantitative formation of compound 2, which was verified by ^1^H NMR spectroscopy to be complete within 15 minutes. The ^1^H NMR spectrum of 2 in *d*_6_-benzene exhibited two sets of mutually coupled doublet signals (*δ*_H_ = 6.08, 5.26 ppm; *J*_HH_ = 21.3 Hz), diagnostic of an *E*-alkenyl moiety. Two septet (*δ*_H_ = 4.60, 4.41 ppm) and two singlet resonances were assigned to the respective and differentiated *iso*-propyl and SiMe_2_ substituents (*δ*_H_ = 0.54, 0.47 ppm) of the {SiN^Dipp^} ligand (Fig. S1†). On this basis, compound 2 was identified as a copper(i) (*E*-phenylethenyl)(phenylethynyl)aluminate complex (Scheme 1). Although all attempts to isolate 2 were unsuccessful due to the labile nature of the molecule, the viability of the implied aluminate anion was confirmed by the isolation of several single crystals of compound 2a, which were deposited during storage of the reaction mixture. The resultant X-ray diffraction analysis (Fig. 2a, Table 1) revealed that compound 2a comprises a charge separated diorganoaluminate anion in which the SiN^Dipp^-supported Al1 centre is bonded to phenylacetylide [C42–C43 1.203(2) Å] and *E*-phenylvinylide [C44–C45 1.327(2) Å] subunits. Although the fate of the copper centre of 1 and the necessary hydrogen source could not be identified, charge balance is maintained by a wholly organic imidazolium cation arising from protonation of the NHC^iPr^ co-ligand. The C–C distance [C44–C45 1.327(2) Å] within the vinylaluminium unit is consistent with its attribution as a double bond while the relevant aluminium to carbon bond [Al1–C44 1.9876(14) Å] is commensurate with those observed in previously reported Al–C_alkenyl_ structures.^14^

**Table 1:** Selected bond lengths (Å) and bond angles (^°^) of compound 2a, 3, 7, 8 and 10

	2a	3	7	8	10
Al1–N1	1.8745(11)	1.836(3)	1.8429(12)	1.8449(19)	1.8523(11)
Al1–N2	1.8799(11)	1.845(3)	1.8507(12)	1.8537(19)	—
Al1–C42	1.9959(14)	1.990(3)	1.9972(14)	1.983(2)	1.9852(15)^a^
Al1–C44	1.9876(14)	1.979(3)	1.9980(14)	1.975(3)	—
C42–C43	1.203(2)				
C44–C45	1.327(2)				
Cu1–C31	—	1.960(3)	1.9614(13)	1.957(2)	1.980(2)^b^
Cu1–C42	—	2.135(3)	2.1595(13)	2.163(2)	2.1846(13)^c^
Cu1–C43	—	2.239(3)	2.1641(14)	2.209(3)	2.3534(14)^d^
Cu1–C44	—	2.158(3)	2.1698(13)	2.131(2)	—
Cu1–C45	—	2.245(3)	2.1763(14)	2.200(3)	—
N1–Al1–N2	112.07(5)	115.80(12)	113.13(5)	114.61(9)	113.23(7)^e^
C44–Al1–C42	103.76(6)	92.39(12)		92.19(10)	91.70(8)^f^
N1–Al1–C42	105.57(5)	113.40(12)		116.88(9)	105.04(5)^g^
N2–Al1–C44	106.21(5)	117.32(12)		117.71(9)	—

a Al1–C22.

b Cu1–C16.

c Cu1–C22.

d Cu1–C23.

e N1^1^–Al1–N1.

f C22–Al1–C22^1^.

g N1–Al1–C22.

Continued spectroscopic monitoring of reaction mixtures containing 2 over the course of several days at room temperature also revealed the presence of trace amounts of styrene and minor quantities of a further new species, compound 3. A solution of compound 2 prepared *in situ* was, therefore, treated with a further equivalent of phenylacetylene and heated at 60 °C for 12 hours. This process initiated complete conversion to compound 3 and the liberation of an equimolar quantity of styrene, which was readily identifiable in the resultant ^1^H NMR spectrum and was deduced to have arisen from the apparent protonolysis of the *E*-phenylvinylide unit of 2 by the acidic proton (p*K*_a_*ca.* 26)^15^ of the additional phenylacetylene equivalent (Scheme 1). The ^1^H NMR signals arising from the NHC^iPr^ and SiN^Dipp^ ligand environments associated with compound 3 were consistent with a *C*_2v_-symmetric species, an inference subsequently confirmed by single-crystal X-ray diffraction analysis (Fig. 2b). This solid-state characterisation identified 3 as a molecular heterobimetallic complex, comprising a NHC^iPr^-coordinated copper centre that is further ligated by twofold dihapto binding to a SiN^Dipp^-supported bis(phenylethynyl)aluminate anion. The similarity of the Al–C_sp_ distances in 3 [Al1–C42 = 1.990(3) Å, Al1–C34 = 1.979(3) Å] and the comparable Al–C bond of the unperturbed anion of compound 2a [Al1–C42 1.9959(14) Å], lend credence to the continued attribution of a formally anionic nature to the aluminium centre in 3.

**Scheme 2:**
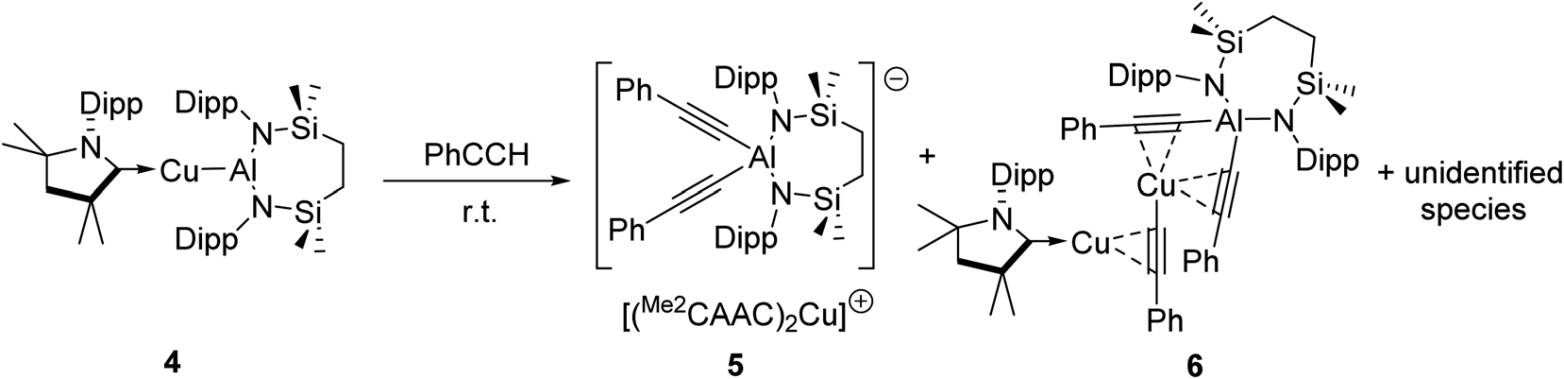
Reaction of [(^Me2^CAAC)CuAl{SiN^Dipp^}] (4) with phenylacetylene.

We have previously described the impact on the reactivity of the Cu–Al bond induced through variation of the copper-coordinated carbene ligand and the differentiated behaviour observed across the metals of the group 11 triad.^13^ To investigate the generality of the transformations observed to provide compounds 2 and 3, therefore, phenylacetylene was reacted with further compounds comprising unsupported group 11–Al{SiN^Dipp^} bonds. In stark contrast to the exclusive production of 2, treatment of [(^Me2^CAAC)CuAl{SiN^Dipp^}] (4), containing the more basic ^Me2^CAAC carbene (^Me2^CAAC = 1-(2,6-di-isopropylphenyl)-3,3,5,5-tetramethylpyrrolidin-2-ylidene), with phenylacetylene provided a mixture of several products, even at room temperature and irrespective of the reaction stoichiometry. Although fractional crystallisation of these reaction mixtures yielded single crystals of two further species, compounds 5 and 6, and comprising the [(PhC≡C)_2_Al{SiN^Dipp^}]^−^ anion observed in compound 3, the introduction of the ^Me2^CAAC ligand evidently results in more labile heterobimetallic species and no pure, bulk samples could be isolated (Scheme 2). Compound 5 was identified by a single crystal X-ray analysis as a further charge separated heterobimetallic species, in which charge balance with the aluminate is maintained by a linear [(^Me2^CAAC)_2_Cu]^+^ cation (Fig. S48†). Similarly, X-ray diffraction analysis identified compound 6 as a heterotrimetallic {Cu_2_Al} complex, which may be viewed as resulting from the formal insertion of a [(PhC≡C)Cu] unit into the copper-to-carbene bond of a ^Me2^CAAC derivative, with an aluminate structure otherwise analogous to that of compound 3 (Fig. S49†). To a similar end, the reactivity of Ag–Al and Au–Al bonds towards phenylacetylene was explored by exploiting the previously reported heavier group-11 analogues of 1, [(NHC^iPr^)AgAl{SiN^Dipp^}] and [(NHC^iPr^)AuAl{SiN^Dipp^}].^13*b*^ In neither case, however, was any reaction observed at ambient temperature, while the application of external heating invariably resulted in decomposition and the deposition of a black precipitate assumed to be the elemental group 11 metal.

### Substituent effect of terminal alkynes

With these observations in hand, it was evident that the initially selected phenylacetylene reagent provided insufficient kinetic discrimination to allow the identification of further potential species formed prior to the generation of the (alkenyl)(alkynyl)aluminate exemplified by compound 2 (2a). We, thus, turned our attention to a broader assay of the reactivity of compound 1 with the alternative terminal alkynes, 1-hexyne, 3,3-dimethylbut-1-yne, and trimethylsilylacetylene.

**Scheme 3:**
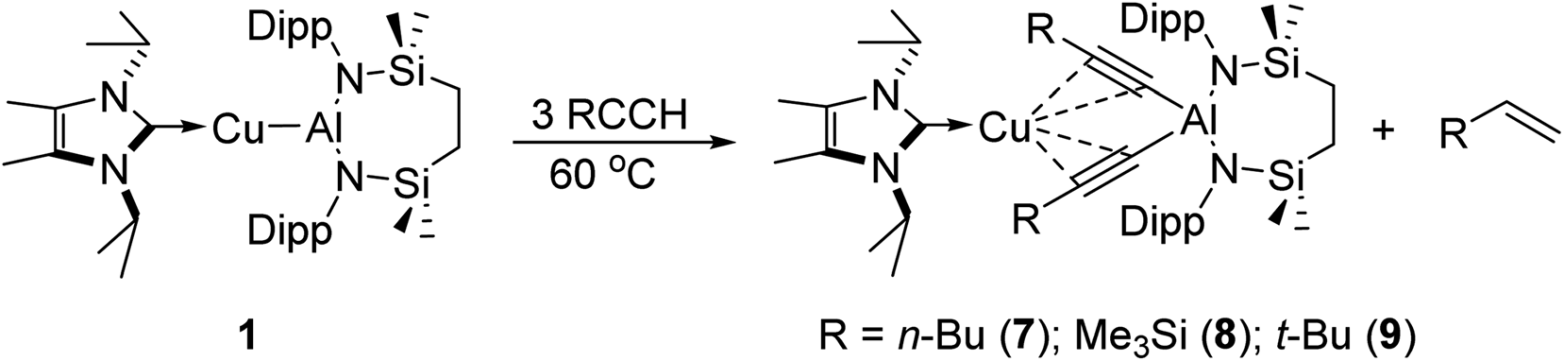
Reaction of compound 1 with 3 equivalents of various terminal alkynes.

**Fig. 3:**
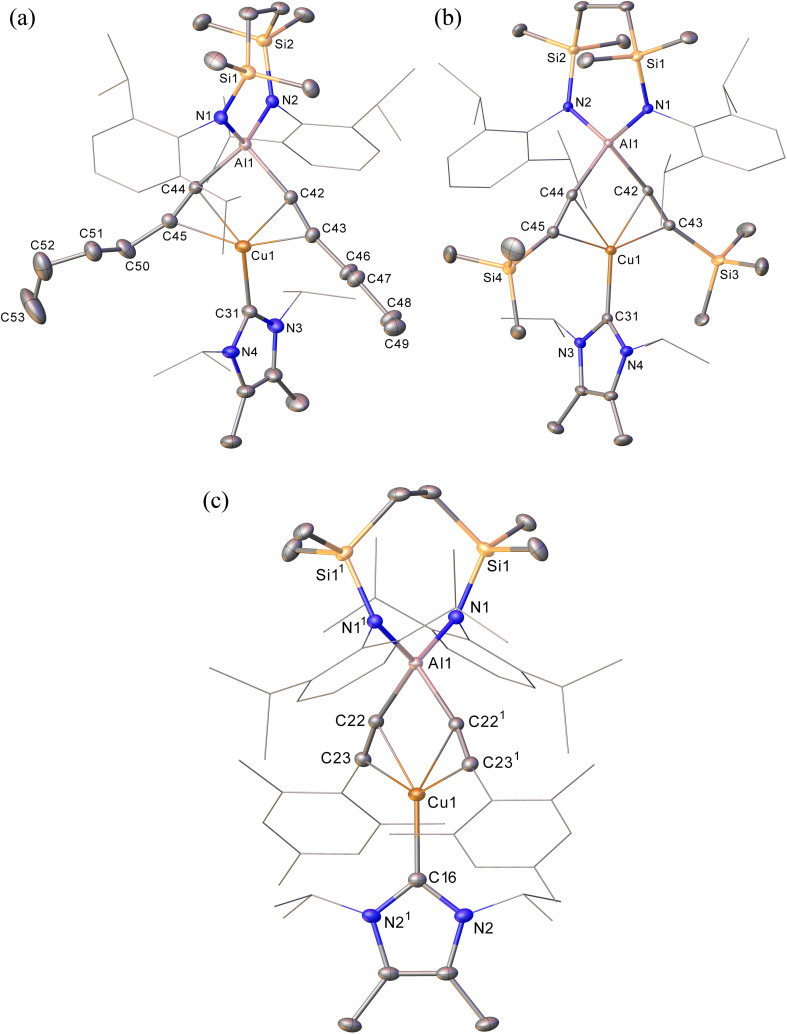
(a) Displacement ellipsoid (30% probability) plots of (a) compound 7; (b) compound 8; (c) compound 10. For clarity, hydrogen atoms, disorder (in 7 and 8) and solvent (in 10), have been omitted while Dipp, *mes* and *iso*-propyl substituents are shown as wireframe. Symmetry operations: 10,^1^ 2 − *x*, *y*, 3/2 − *z*.

Initial studies assessed the generality of the chemistry leading to compound 3. A series of reactions were performed between three equivalents of each alkyne and compound 1 in C_6_D_6_ solution. The progress of each reaction at 60 °C was then monitored through the acquisition of their ^1^H NMR spectra until the starting materials had been completely consumed. While the reaction of 1 with the less sterically demanding 1-hexyne was again complete within approximately 12 hours, the bulkier acetylenes required *ca*. 3 days to achieve complete conversion. In a comparable fashion to the reactivity observed between 1 and phenylacetylene, each of these reactions resulted in the generation of a single stoichiometric equivalent of the corresponding terminal alkene and the production of a series of complexes, [(NHC^iPr^)Cu{(RC≡C)_2_Al{SiN^Dipp^}] (7–9) (Scheme 3). All three compounds displayed ^1^H and ^13^C{^1^H} spectra consistent with solution structures comparable to that identified for compound 3, an assignment that was confirmed by single crystal X-ray diffraction analysis for compounds 7 and 8 (Fig. 3a and b, Table 1). The molecular structures of both compounds exhibit similar features to that of 3, with the coordination sphere of a {SiN^Dipp^}-supported aluminate completed by two σ-bonded acetylides, with each of these anions chelated to the metal centre of the {Cu(NHC^iPr^)} components of the molecules *via* a pair of η^2^–π-interactions. Although the resultant metal-carbon distances show some dependence on the steric bulk of the alkynyl substituent (Table 1), the similarity of the structures to that of compound 3 obviates further necessary comment.

**Scheme 4:**
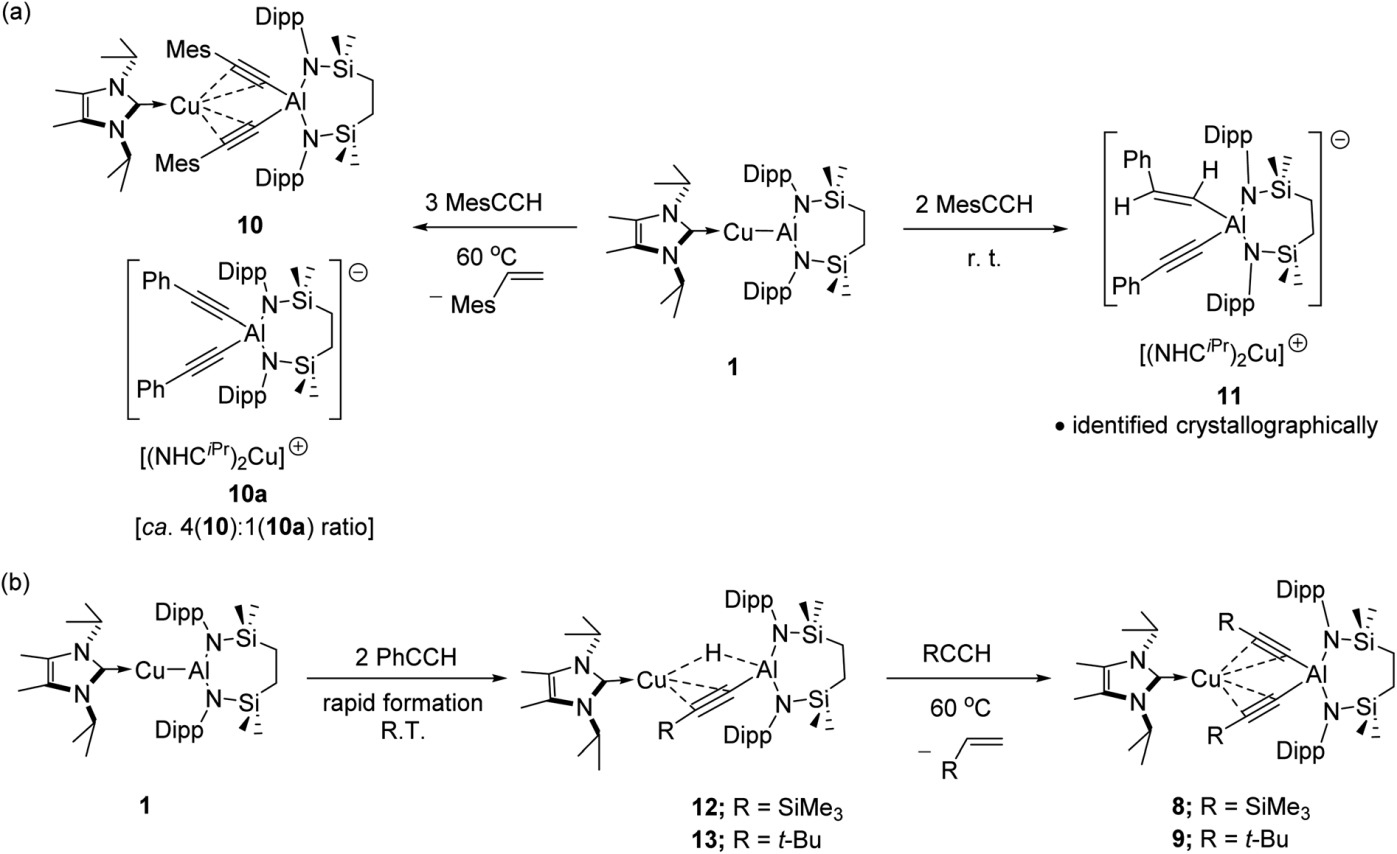
(a) Reactivity of 1 toward MesCCH; (b) stepwise reaction of 1 with RCCH (R = SiMe_3_; *t*-Bu).

The selective formation of compounds 7–9 indicates that the Al–Cu bond of 1 displays analogous reactivity toward terminal acetylenes irrespective of any electronic or steric variations. Attempted extension of this study to the reaction of 1 with three equivalents of 2,4,6-trimethylphenylacetylene at the same 60 °C temperature, however, resulted in a mixture of two compounds, 10 and 10a, which could be tentatively identified in an approximate 4 : 1 ratio by analysis of the ^1^H NMR spectrum provided by the crude reaction mixture. Although pure bulk samples of neither compound could be obtained, crystallisation of the reaction solution and mechanical separation of the resultant single crystals enabled the identification of 10 (Scheme 4a, Fig. 3c and Table 1) and 10a (Scheme 4a and Fig. S50†) as the respective molecular and charge separated analogues of compounds 3 and 5. Suspecting the reduced stability of the heterobimetallic species 10 towards extrusion of the copper cation to be consequence of the modified steric profile of the 2,4,6-trimethylphenylacetylide unit under the thermal conditions used in its synthesis, the reaction was repeated at room temperature. This procedure, however, resulted in the deposition of a further compound, 11, as a colourless crystalline solid. Although the subsequent insolubility of 11 precluded its further characterisation in solution, its identity as a further charge separated derivative, [(NHC^iPr^)Cu(NHC^iPr^)][(MesCHCH)(MesCC)Al{SiN^Dipp^}], comprising a (mesitylethenyl)(*E-*mesitylethynyl)aluminate analogous to that characterised in the structure of 2a, but in this case with charge balance achieved by a bis-carbene ligated copper cation, was confirmed by a further X-ray diffraction analysis (Scheme 4a and Fig. S51†).

**Fig. 4:**
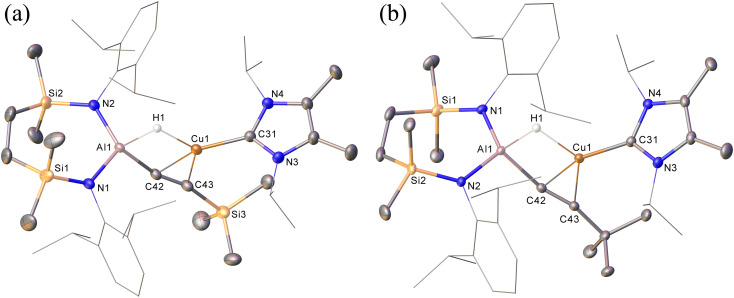
(a) Displacement ellipsoid (30% probability) plots of (a) compound 12; (b) compound 13. For clarity, hydrogen atoms, apart from H1 in both structures, have been omitted while Dipp and *iso*-propyl substituents are shown as wireframe. Selected bond lengths (Å) and angles (^°^): (12) Al1–N1 1.837(2), Al1–N2 1.836(2), Al1–C42 1.963(2), Cu1–C31 1.951(2), Cu1–C42 2.084(2), Cu1–C43, 2.157(2), N1–Al1–C42 109.35(10), N2–Al1–N1 115.80(10), N2–Al1–C42, 116.55(10); (13) Al1–N1 1.8373(12), Al1–N2 1.8441(12), Al1–C42 1.9622(15), Cu1–C31 1.9494(15), Cu1–C42 2.0703(14), Cu1–C43 2.1740(14), N1–Al1–N2 115.51(6), N1–Al1–C42 117.29(6), N2–Al1–C42 107.97(6).

Prompted by the significantly extended reaction times required in the synthesis of compounds 8 and 9, equimolar reactions between 1 and the more sterically encumbered trimethylsilylacetylene and 3,3-dimethylbut-1-yne were performed at room temperature. Assessment by *in situ*^1^H NMR spectroscopy evidenced the selective formation of single predominant new species in both reactions, which were characterised by a loss of the *C*_2_ symmetry associated with the chelated SiN^Dipp^ ligand of compound 1. Crystallisation of the reaction mixtures in both cases afforded single crystals suitable for X-ray crystallography, which revealed their identities to be the μ-hydride- and η^2^-Cu-κ^1^-acetylide-bridged copper species differing solely in their respective, trimethylsilyl-(12) and *tert*-butylalkynyl (13) substituents (Scheme 4b, Fig. 4). Although copper hydride species are by no means uncommon,^16^ molecular derivatives comprising comparable Al-μ-H-Cu bridging are limited to a single report of several compounds arising from the treatment of β-diketiminato Cu(i) complexes with similarly ligated aluminium dihydrides.^17^

In contrast to similar reactions performed with both phenylacetylene (Scheme 1) and 1-hexyne, no evidence for the generation of copper-(alkenyl)(alkynyl)-aluminate species analogous to 2/2a could be observed. Heating (60 °C), however, of both heterobimetallic hydrides 12 and 13 in the presence of additional equivalents of the relevant acetylenes provided the corresponding bis(alkynyl)aluminate derivatives, 8 and 9, respectively (Scheme 4b).

### Computational mechanistic studies

**Scheme 5:**
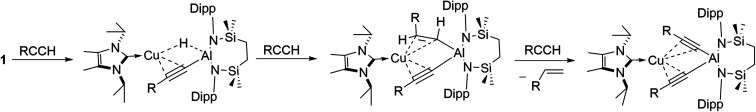
Proposed stepwise transformation of 1 with acetylenes.

The reactivity instigated by treatment of 1 with various terminal acetylenes is suggestive of the common stepwise reaction sequence depicted in Scheme 5. Under this regime, initial activation of the first alkynyl C_sp_–H bond is achieved through its reduction at the Cu–Al bond. The resultant hydride derivatives akin to compounds 12 and 13 react *via syn* addition across the resultant Al–H bond to yield species analogous to compound 2 and comprising (alkenyl)(alkynyl)aluminate anions exemplified by the solid-state structures of 2a and 11. Alkene release is then achieved by protonolysis of the more basic vinylaluminium residue by a further equivalent of the terminal alkyne.

**Fig. 5:**
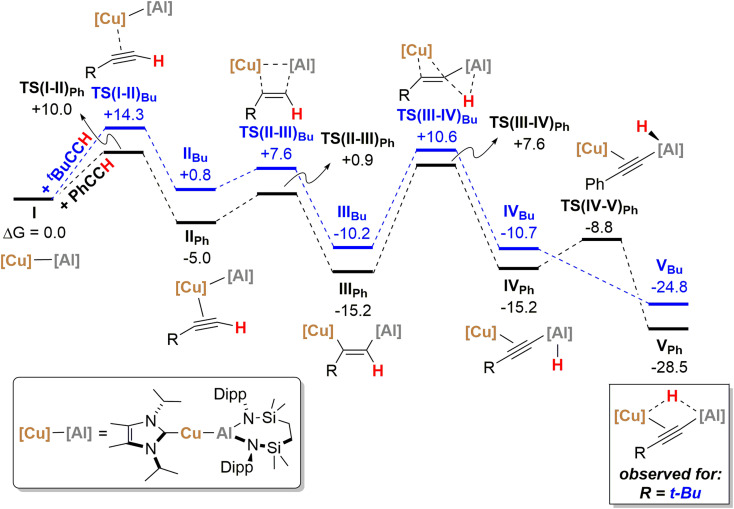
Computed free energy profile (BP86-D3BJ(PCM = C_6_H_6_)/BS2//BP86/BS1 level, energies quoted in kcal mol^−1^) of formation of bridging hydridocopperalkynylalumanyl species from Cu–Al species 1/I with both phenylacetylene (black) and 3,3-dimethylbut-1-yne (blue).

**Fig. 6:**
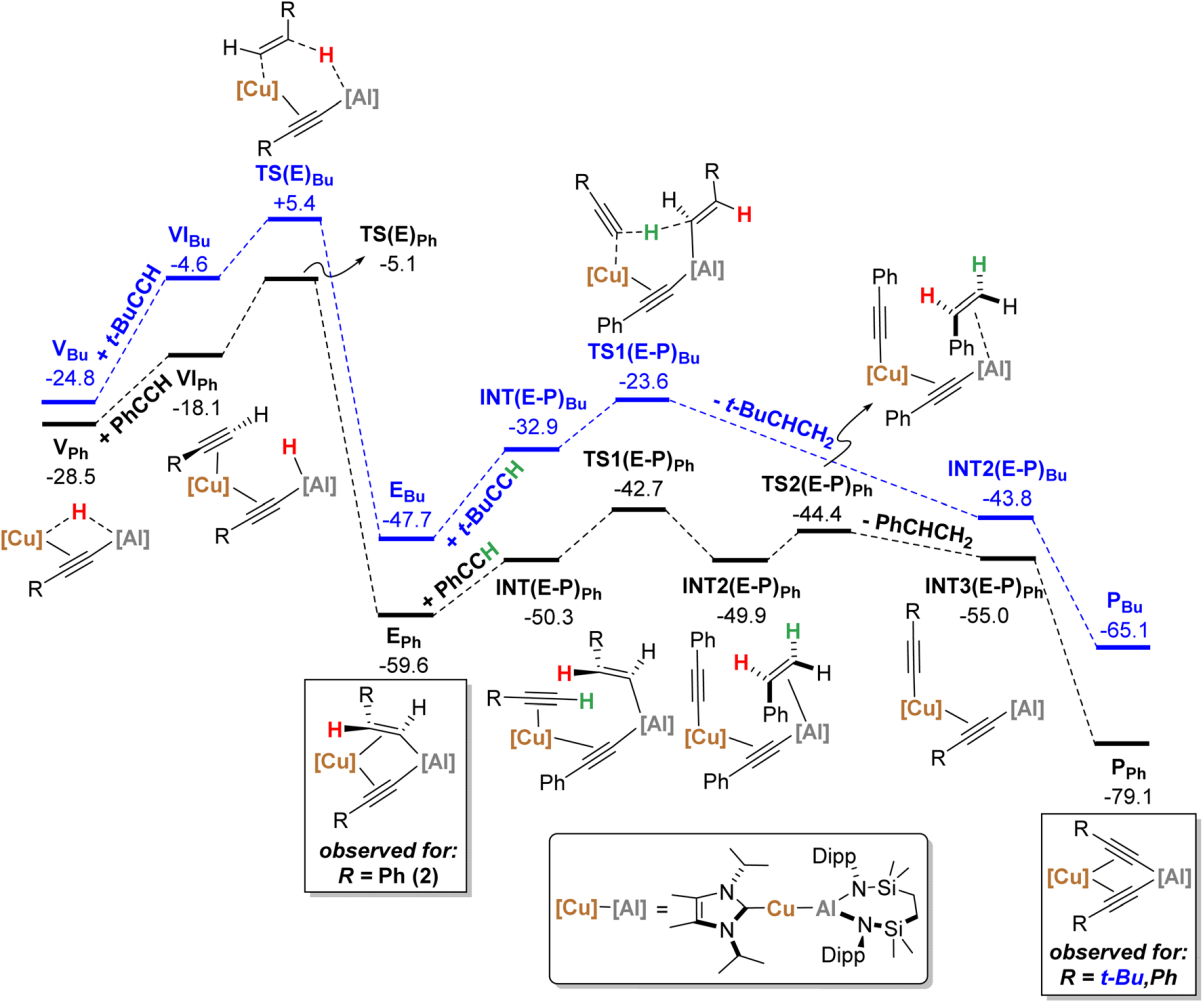
Computed free energy profile (BP86-D3BJ(PCM = C_6_H_6_)/BS2//BP86/BS1 level, energies quoted in kcal mol^−1^) of onwards formation of P_Ph_ (black) and P_Bu_ (blue) from V_Ph_ and V_Bu_, respectively.

With the experimental results and the proposed stepwise mechanism in hand, the reactivity of compound 1 with both phenylacetylene (PhCCH) and 3,3-dimethylbut-1-yne (*t-*BuCCH) was assessed by DFT at the BP86-D3BJ, (PCM = C_6_H_6_)/BS2//BP86/BS1 level of theory (see the ESI† for full computational details and results). Initial calculations focussed on these two substrates owing to the evidently contrasting kinetic facility of their reactivity with 1 under ambient conditions. Although a common pathway may be assumed, the hydride-bridged species 13 could be characterised upon addition of two equivalents of *t-*BuCCH at room temperature (Scheme 4), with onwards addition of further acetylene equivalents only affording 10 upon heating. Conversely, no evidence of hydride formation could be observed during reactions of 1 with phenylacetylene, with the (*E-*phenylethenyl)(phenylethynyl)aluminate 2 as the first observable product prior to the formation of 3. Fig. 5 details the free energy pathway of formation of bridging hydride species for both terminal acetylenes, while Fig. 6 illustrates the onward reactivity to form 3 and 10, respectively for PhCCH and *t-*BuCCH. For ease of discrimination, compound 1 is relabelled as I in this computational study.

Beginning with PhCCH (black, Fig. 5), alkyne coordination to the Cu centre of I*via* an η^2^ interaction takes place *via*TS(I-II)_Ph_ (+10.0 kcal mol^−1^) and results in the exergonic formation of II_Ph_ (−5.0 kcal mol^−1^). Subsequently facile Al–C bond formation proceeds with simultaneous Cu–Al cleavage *via*TS(II-III)_Ph_, (+0.9 kcal mol^−1^) to form the *syn-*addition product III_Ph_ (−15.2 kcal mol^−1^). C–H cleavage then takes place *via*TS(III-IV)Ph (+7.6 kcal mol^−1^) with an activation barrier of 22.8 kcal mol^−1^, in which Al–H bond formation occurs concomitantly with slippage of the NHC^iPr^–Cu moiety across the alkynyl group to yield IV_Ph_ (−15.2 kcal mol^−1^). It must be emphasised that this mode of C–H activation differs to that of direct Ph–C≡C–H deprotonation at the alumanyl centre, with subsequent Al–C formation. Migration of the NHC^iPr^–Cu unit then occurs through rotation about the Ph–C≡C– vector *via*TS(IV-V)_Ph_ (−8.8 kcal mol^−1^) to afford the bridging hydridocopper species V_Ph_ (−28.5 kcal mol^−1^). Moving to the *t-*BuCCH profile (blue, Fig. 5), alkyne addition *via*TS(I-II)_Bu_ (+14.3 kcal mol^−1^) proceeds with an identical mode of η^2^-coordination to the Cu centre, but with a higher barrier than that of PhCCH. The resultant *Z*-alkenyl species III_Bu_ (−10.2 kcal mol^−1^) ultimately forms in an analogous manner to that of PhCCH, albeit less exergonically. Consistent with the observation of 12, however, the formation of the bridging hydride species V_Bu_ is also kinetically feasible at room temperature from III_Bu_*via* the energetically accessible TS(III-IV) (Δ*G*^‡^ = 20.8 kcal mol^−1^).^18^ The calculations, thus, validate the kinetic and thermodynamic viability of the bridging hydride intermediates for both alkynes. The contrasting stabilities of V_Ph_ and V_Bu_ evident from the synthetic study, however, may be attributed to their subsequent facility towards reactivity with further equivalents of alkyne, as depicted in Scheme 5 and Fig. 6.

From V_Ph_, addition of a second equivalent of PhCCH (black, Fig. 6) affords VI_Ph_ (−18.1 kcal mol^−1^) *via* η^2^-coordination with Cu and concomitant cleavage of the Cu–H bridging interaction. Formation of the *E*-phenylvinylide adduct, E_Ph_ (−59.6 kcal mol^−1^), is then initiated by hydride transfer *via*TS(E)_Ph_ (−5.1 kcal mol^−1^) and an energetic span of 23.4 kcal mol^−1^ (relative to V_Ph_).^19^ It should be noted that kinetically viable H-transfer routes were also identified from VI_Ph_ that afford 1,1-phenylvinylidenyl-(*via*TS(1,1)_Ph_, −6.9 kcal mol^−1^) and *Z*-phenylvinylidyl- (*via*TS(Z)_Ph_, −6.6 kcal mol^−1^) derivatives. While these processes have comparable activation barriers, the ultimate product, E_Ph_, is thermodynamically favoured, suggesting that this product dominates in a post-H-transfer equilibration given that the calculations indicate H-transfer is irreversible (see the ESI† for further details). From E_Ph_, uptake of a third equivalent of PhCCH affords INT(E-P)_Ph_ (−50.3 kcal mol^−1^), whereupon subsequent proton transfer to the *E*-phenylvinylide moiety *via*TS(E-P)_Ph_ (−42.7 kcal mol^−1^) is facile to yield INT2(E-P)_Ph_ (−49.9 kcal mol^−1^). Finally, styrene-dissociation from the Al centre, simultaneous with Cu to Al acetylide transfer, *via*TS2(E-P)_Ph_ (−44.4 kcal mol^−1^), releases styrene to form INT3(E-P)_Ph_ (−55.0 kcal mol^−1^), and ultimately, P_Ph_ (−79.1 kcal mol^−1^, equivalent to 3 in the synthetic study) following Ph–C≡C transfer to the Al centre.

Characterisation of *t*-BuCCH addition to V_Bu_ (blue, Fig. 6) reveals an analogous η^2^-adduct, VI_Bu_ (−4.6 kcal mol^−1^), and subsequent H-transfer to form the *E*-alkyenyl adduct E_Bu_ (−47.7 kcal mol^−1^) proceeds *via*TS(E)_Bu_ (+5.4 kcal mol^−1^). The energetic span in this case, however, (from V_Bu_) is 30.2 kcal mol^−1^, indicating that the observation of 12 (V_Bu_) at room temperature is due to the kinetic disinclination of V_Bu_ towards uptake of an additional bulky *t-*BuCCH to initiate H-transfer at ambient conditions to form E_Bu_.

## Conclusion

In summary, we show that the heterometallic Cu–Al σ bond of [{SiN^Dipp^}Al–Cu(NHC^iPr^)] reacts in a stepwise fashion with three equivalents of terminal alkyne to provide sequential formation of cuprous (hydrido)(alkynyl)aluminate, (*E-*alkenyl)(alkynyl)aluminate and bis(alkynyl)aluminate derivatives. The alkene liberated during this latter reaction step constitutes a unique case of alkyne transfer semi-hydrogenation in which the C–H acidic alkyne itself acts as a source of proton and in which the requisite reducing electrons are provided by the copper alumanyl. In effect, therefore, the Cu–Al bond, in conjunction with the C–H acidic terminal alkyne, provides the source of hydride necessary to effect substrate reduction. The reaction sequence deduced from the synthetic study is supported by DFT calculations, which have rationalised the variable stability of the initially formed heterobimetallic intermediates. We are continuing to examine this chemistry and are seeking to apply this unusual source of hydride to the controlled reduction of a wider range multiply-bonded small molecules.

## Data availability

All experimental and computational data associated with this article may be found in the ESI.†

## Author contributions

MSH and CLM conceived and directed the study and finalised the manuscript for submission. HYL authored the first draft of the manuscript, performed all the synthesis and spectroscopic characterisation and collected and solved the single crystal X-ray diffraction solved. SEM carried out and interpreted the results of the computational studies. MFM finalised all the X-ray data for publication.

## Conflicts of interest

There are no conflicts to declare.

## Supplementary Material

SC-014-D3SC00240C-s001

SC-014-D3SC00240C-s002
