# Supplementary material for: Structural snapshots of an Al–Cu bond-mediated transformation of terminal acetylenes
Source: Chem Sci. 2023 Feb 15;14(11):2866–76. doi: 10.1039/d3sc00240c (PMC10016343; doi:10.1039/d3sc00240c)
Supplement: SC-014-D3SC00240C-s001 [file SC-014-D3SC00240C-s001.pdf]

Electronic Supplementary Material (ESI) for Chemical Science.
This journal is © The Royal Society of Chemistry 2023

Structural Snapshots of an Al–Cu Bond-Mediated Transformation of Terminal Acetylenes

Han-Ying Liu, Samuel E. Neale, Michael S. Hill,* Mary F. Mahon and Claire L. McMullin*

Department of Chemistry, University of Bath, Claverton Down, Bath, BA2 7AY, UK

1 Experimental and Supplementary Information

1.1. General information

Except stated otherwise, all the experiments were conducted using standard Schlenk line and/or glovebox techniques under an inert atmosphere of argon. NMR spectra were recorded with an Agilent ProPulse spectrometer (^{1}H at 500 MHz, ^{13}C at 126 MHz). The spectra are referenced relative to residual protio solvent resonances. Elemental analyses were performed at Elemental Microanalysis Ltd., Okehampton, Devon, UK. Solvents were dried by passage through a commercially available solvent purification system and stored under argon in ampoules over 4 Å molecular sieves. C_6D_6 and d_8-THF was purchased from Sigma-Aldrich, dried over a potassium mirror before distilling and storage over molecular sieves. [(NHC^{iPr})Cu-Al{SiN^{Dipp}}] (**1**) and [(^{Me_2}CAAC)Cu-Al{SiN^{Dipp}}] (**4**) were prepared according to reported procedures. [1] All other chemicals were purchased from Merck and used without further purification.

1.2. Synthetic Procedures

Synthesis of [(NHC^{iPr})Cu]…[(PhC^{H}C^{H})(PhCC)-κ^{2}-C,C'-Al{SiN^{Dipp}}] (**2**)

In a J Young's tube, phenylacetylene (5.1 mg, 5.5 μL, 0.05 mmol) was added via micropipette to a colourless solution of [(NHC^{iPr})Cu-Al{SiN^{Dipp}}] (**1**, 19 mg, 0.025 mmol) in C_6D_6. Quantitative generation of **2** was observed by NMR spectroscopy within 30 mins at room temperature. The colourless solution was then put under vacuum to remove all volatiles and give compound **2** as a colourless waxy solid. Yield 22.5mg, 93%. No meaningful elemental analysis was obtained after several attempts. The related structure of **2a** was characterised by X-ray diffraction analysis on a single crystals obtained by keeping a solution of **2** at −30 °C in hexanes. ^{1}H NMR (500 MHz, 298 K, Benzene-d$_6$) δ 7.27-7.23 (m, 2H, *p*-C_6H_5 of AlCCPh), 7.21-7.16 (m, 4H, *m*-C_6H_3) 7.12 – 7.09 (m, 2H, *p*-C_6H_3), 7.00 – 6.91 (m, 4H, *o*- and *m*-C_6H_5 of AlC_2H_2Ph), 6.85 – 6.80 (m, 3H, C_6H_5 of AlCCPh), 6.79 – 6.73 (m, 1H, *p*-C_6H_5 of AlC_2H_2Ph), 6.08 (d, *J* = 21.3 Hz, 1H, AlC_2H_2), 5.26 (d, *J* = 21.3 Hz, 1H, AlC_2H_2), 4.60 (sept, *J* = 6.8 Hz, 2H, C*H*Me$_2$), 4.41 (sept, *J* = 6.8 Hz, 2H, C*H*Me$_2$), 4.08 (sept, *J* = 7.1 Hz, 2H, NC*H*Me$_2$), 1.58 (d, *J* = 6.8 Hz, 6H, CH*Me$_2$* on SiN^{Dipp}), 1.52 (d, *J* = 6.8 Hz, 6H, CH*Me$_2$* on SiN^{Dipp}), 1.47 (d, *J* = 6.8 Hz, 6H, CH*Me$_2$* on SiN^{Dipp}), 1.41 (s$_{br}$, 4H, SiC*H$_2$*), 1.39 (d, *J* = 6.8 Hz, 6H, CH*Me$_2$* on SiN^{Dipp}), 1.27 (s, 6H, NC*Me*), 0.78 (d, *J* = 7.1 Hz, 12H, NCH*Me$_2$*), 0.54 (s, 6H, Si*Me$_2$*), 0.47 (s, 6H, Si*Me$_2$*). ^{13}C NMR (126 MHz, 298 K, Benzene-d$_6$) δ 180.6 (Cu*C*$_{carbene}$), 149.4, 147.9 (*i*- and *o*- *C*$_6$H$_3$ of SiN^{Dipp}), 147.8 (*i*-*C*$_6$H$_5$ of AlCCPh), 141.5 (*i*-*C*$_6$H$_5$ of AlC_2H_2Ph), 140.0 (Al*C*$_2$H$_2$), 132.0^{+}, 128.4*, 127.5^{+} ($^{+}$*o*- and *m*-C_6H_5 of AlC_2H_2Ph; *Al*C*$_2$H$_2$), 127.3, 126.9 (*C*$_6$H$_5$ of AlCCPh), 126.7 (*p*-*C*$_6$H$_3$ of SiN^{Dipp}), 124.6 (N*C*Me), 123.7, 123.7 (*p*-*C*$_6$H$_5$ of AlC_2H_2Ph and C_6H_5 of AlCCPh), 122.2 (*m*-*C*$_6$H$_3$), 113.1 (AlC*C*Ph), 53.8 (N*C*HMe2), 27.9 (*C*HMe$_2$), 27.8(*C*HMe$_2$), 26.5 (CH*Me$_2$*), 26.5(CH*Me$_2$*), 26.2(CH*Me$_2$*), 26.1 (CH*Me$_2$*), 22.2 (NCH*Me$_2$*), 15.4 (Si*C*H$_2$), 9.7 (NC*Me*), 1.9 (Si*Me$_2$*), 1.8 (Si*Me$_2$*). ^{13}C resonances correlated to Al*C*CPh were not observed.

Figure S1. ^{1}H NMR (500 MHz, 298 K, d_6-benzene) spectrum of **2**. *grease

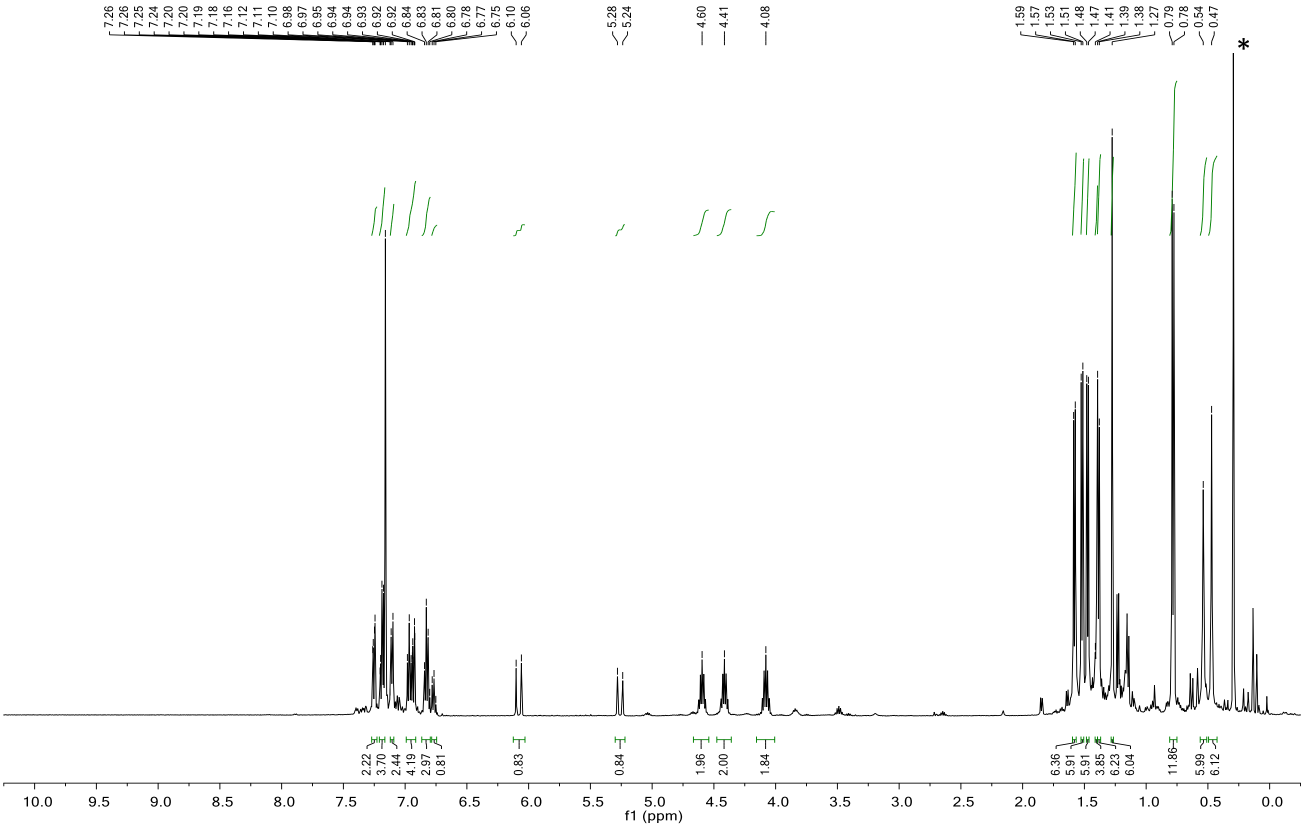

Figure S2. ^{13}C NMR (126 MHz, 298 K, d_6-benzene) spectrum of **2**.

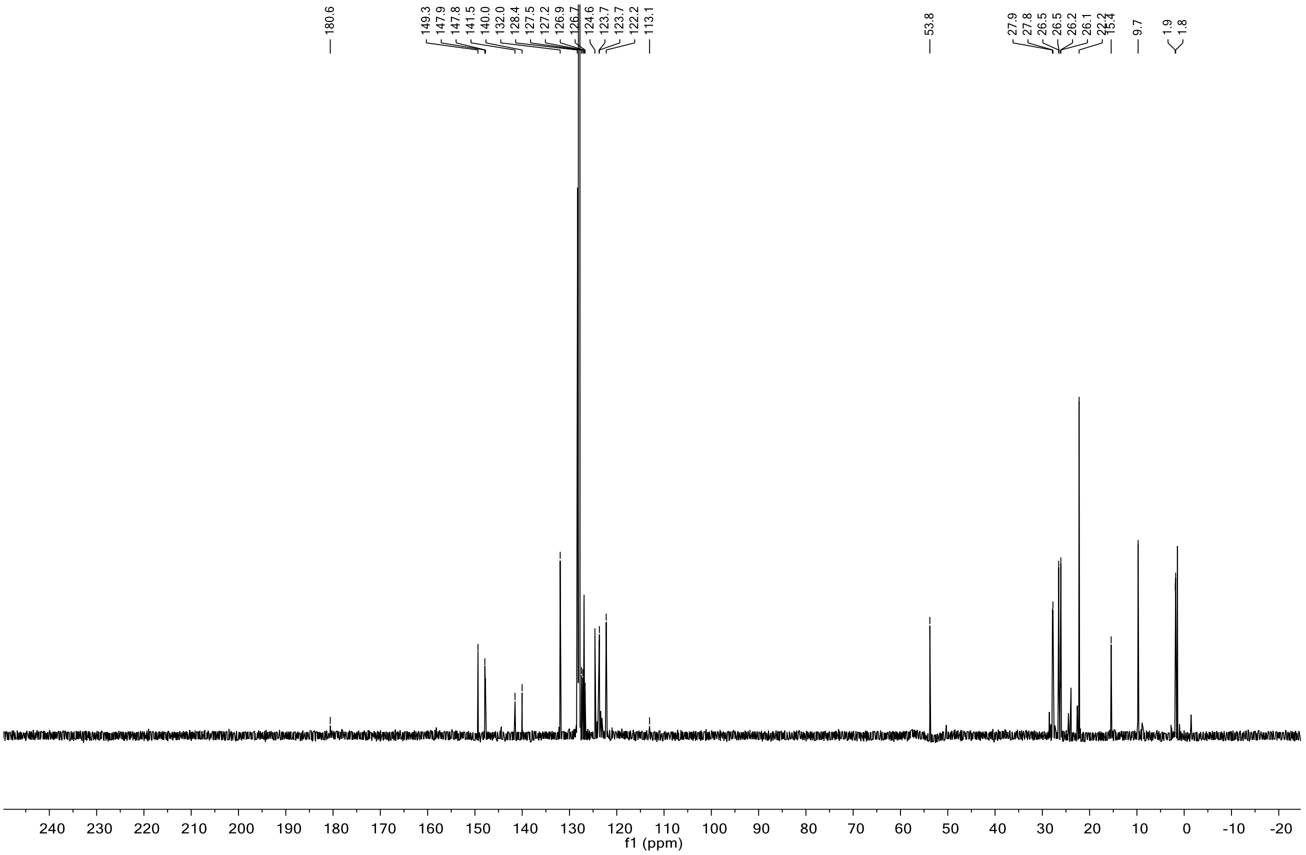

Figure S3. ^{1}H-^{1}H COSY spectrum of **2**.

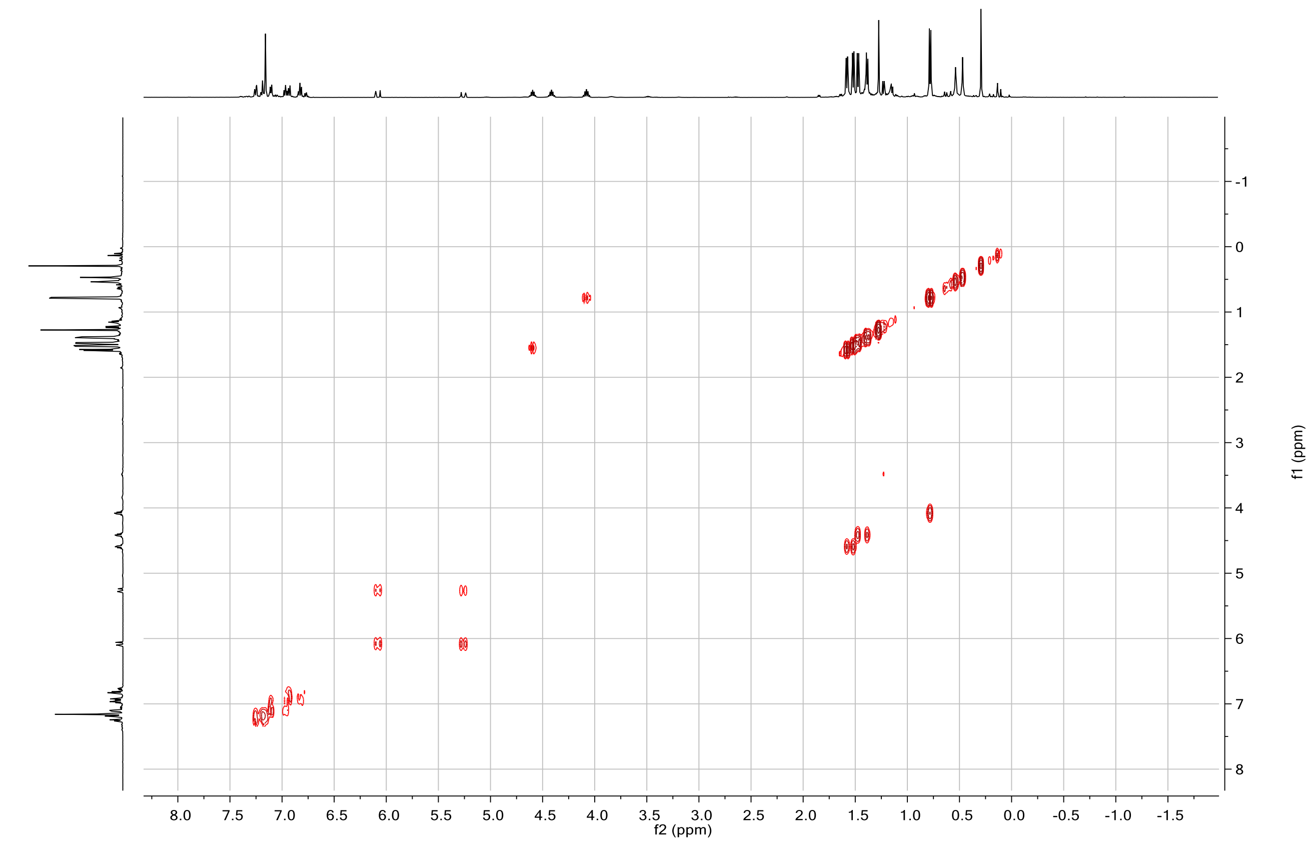

Figure S4. ^{1}H-^{13}C HSQC spectrum of **2**.

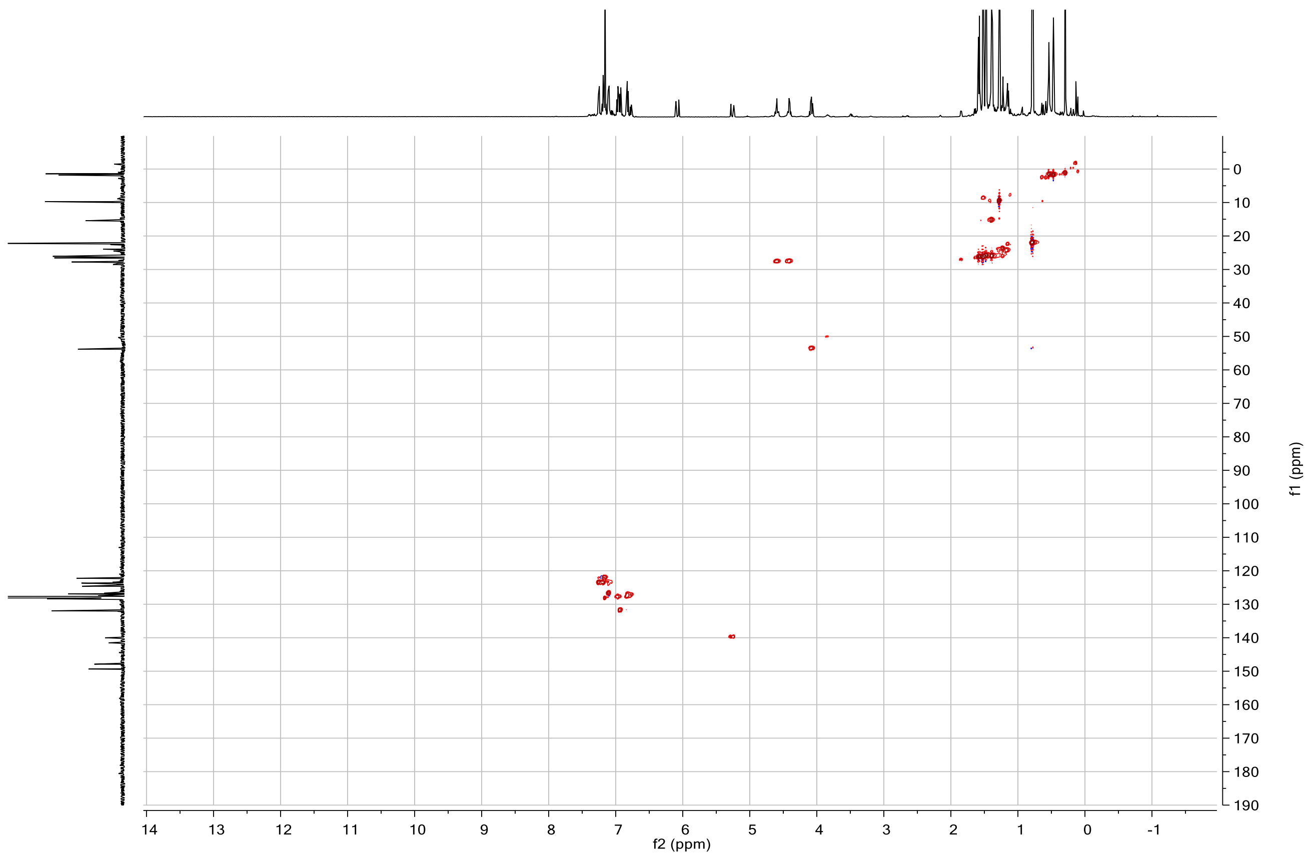

Figure S5. ^{1}H-^{13}C HMBC spectrum of **2**.

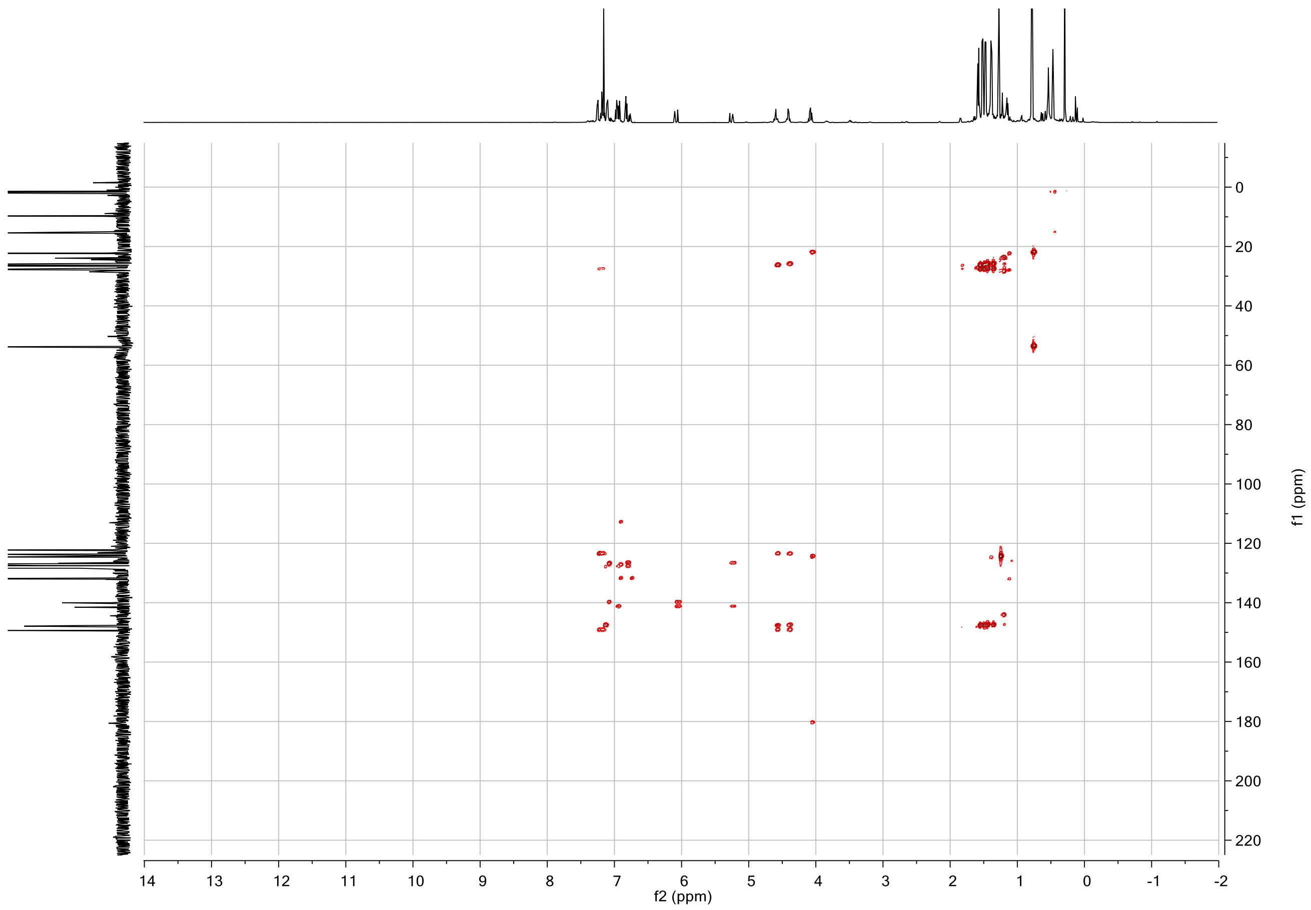

Synthesis of [(NHC^{iPr})Cu…(PhCC)$_2$)-κ^2-C,C’-Al{SiN^{Dipp}}] (**3**)

In a J Young’s tube, phenylacetylene (7.6 mg, 8.2 μL, 0.075 mmol) was added via micropipette to a colourless solution of [(NHC^{iPr})Cu-Al{SiN^{Dipp}}] (**1**, 19 mg, 0.025 mmol) in C_6D_6. The colourless reaction mixture was then kept at 60 °C overnight before full conversion of **1** and generation of styrene was confirmed by NMR spectroscopy. All volatiles were then removed *in vacuo*, affording **3** as a white powder. Yield 20 mg, 83%. Colourless single crystals suitable for X-ray crystallography was obtained by slow evaporation of a hexane solution of **3** at room temperature. Anal Calc’d for $C_{57}H_{81}AlCuN_4Si_2$ (**3**, 969.00) C, 70.65; H, 8.43; N, 5.78 %. Found: C, 70.62; H, 8.36; N, 5.74 %. ^{1}H NMR (500 MHz, 298 K, Benzene-d_6) δ 7.24 (d, *J* = 7.5 Hz, 4H, *m*-C_6H_3 on SiN^{Dipp}), 7.13 (t, *J* = 7.5 Hz, 2H, *p*-C_6H_3 on SiN^{Dipp}), 6.99 – 6.94 (m, 4H, *o*-C_6H_5 on AlCCPh), 6.86 – 6.81 (m, 4H, *m*-C_6H_5 on AlCCPh), 6.80 – 6.74 (m, 2H, *p*-C_6H_5 on AlCCPh), 4.47 (sept, *J* = 6.8 Hz, 4H, C*H*Me$_2$ on SiN^{Dipp}), 4.14 (sept, *J* = 7.0 Hz, 2H, NC*H*Me$_2$), 1.57 (d, *J* = 6.8 Hz, 12H, C*H*Me$_2$ on SiN^{Dipp}), 1.50 (d, *J* = 6.8 Hz, 12H, C*H*Me$_2$ on SiN^{Dipp}), 1.41 (s, 6H, NC*Me*), 1.40 (s, 4H, SiC*H*$_2$), 0.74 (d, *J* = 7.0 Hz, 12H, NCH*Me*$_2$), 0.51 (s (br), 12H, Si*Me*$_2$). ^{13}C NMR (126 MHz, 298 K, Benzene-d_6) δ 181.9 (Cu$C_{carbene}$), 148.5 (*i*-C_6H$_3$ on SiN^{Dipp}), 147.8 (*o*-C_6H$_3$ on SiN^{Dipp}), 132.1 (*o*-C_6H$_5$ of AlCCPh), 127.5 (*m*-C_6H$_5$ of AlCCPh), 126.6 (*p*-C_6H$_5$ of AlCCPh), 125.2 (N*C*Me), 123.4 (*m*-C_6H$_3$ on SiN^{Dipp}), 122.3 (*p*-C_6H$_3$ on SiN^{Dipp}), 110.9 (AlC*C*Ph), 53.9 (N*C*HMe$_2$), 27.9 (*C*HMe2 on SiN^{Dipp}), 26.6 (CH*Me*$_2$ on SiN^{Dipp}), 26.1 (CH*Me*$_2$ on SiN^{Dipp}), 22.1 (NCH*Me*$_2$), 15.5 (Si*C*H$_2$), 9.8 (NC*Me*), 1.5 (Si*Me*$_2$). ^{13}C resonance correlated to Al*C*CPh and *i*-C_6H$_5$ on AlCCPh were not observed.

Figure S6. ^{1}H NMR (500 MHz, 298 K, d_6-benzene) spectrum of **3**. *grease

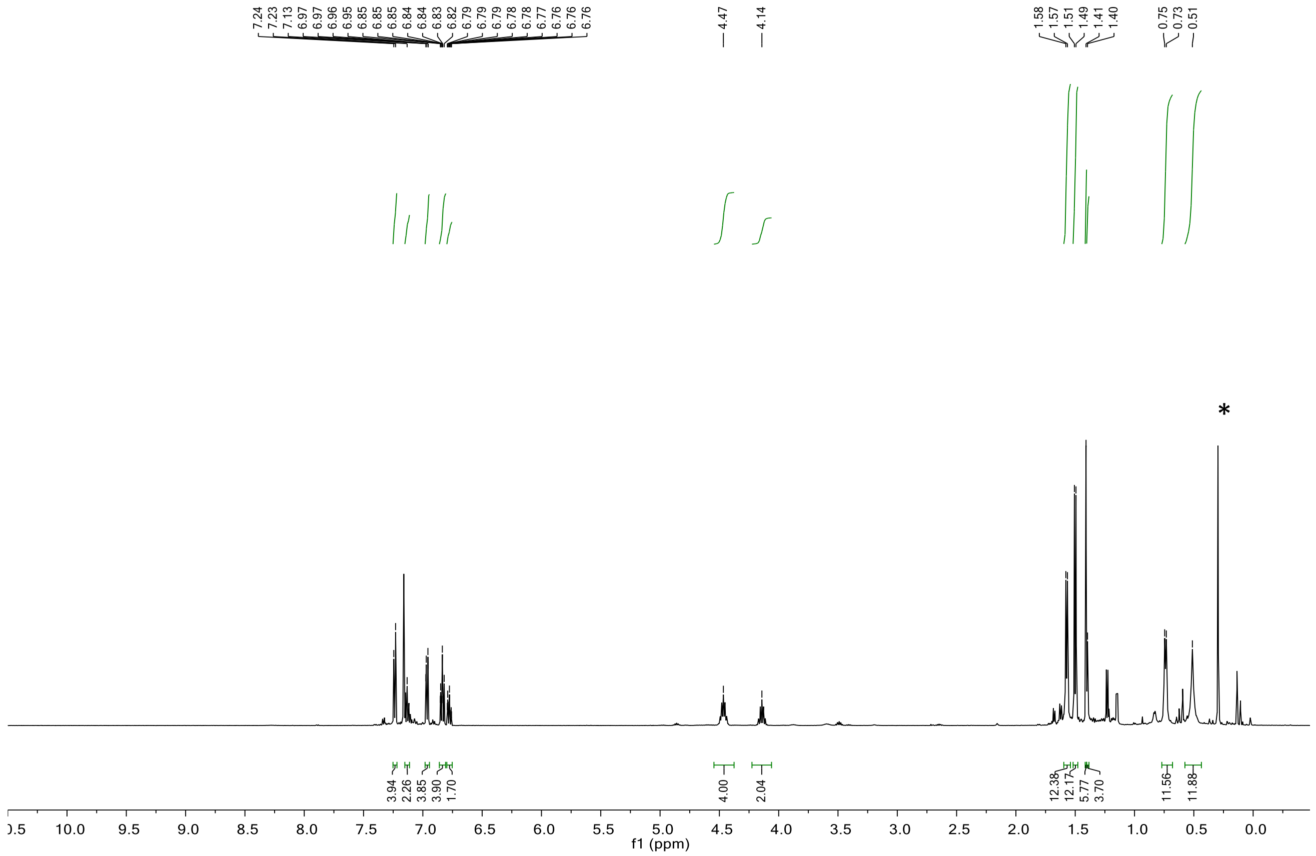

Figure S7. ^{13}C NMR (126 MHz, 298 K, d_6-benzene) spectrum of **3**.

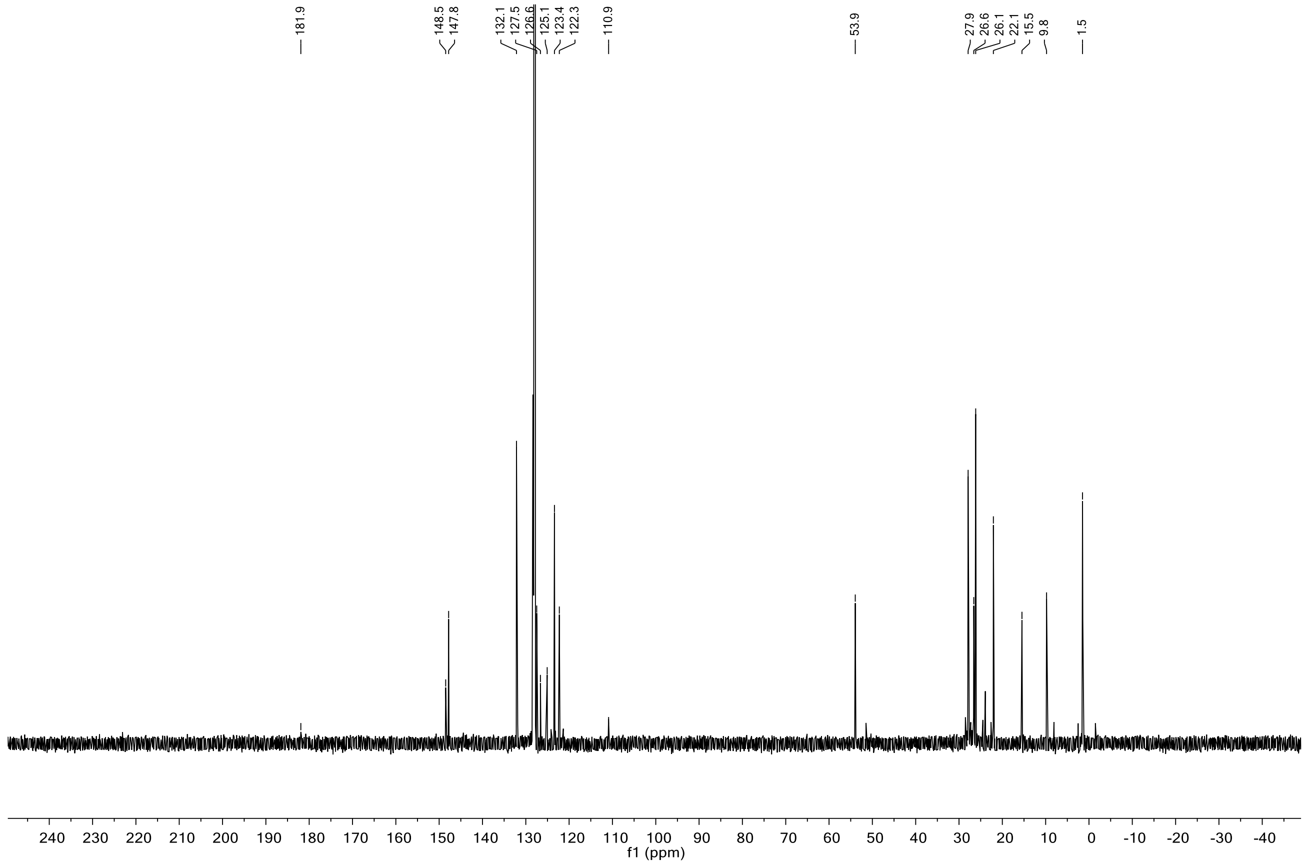

Figure S8. ^{1}H-^{1}H COSY spectrum of **3**.

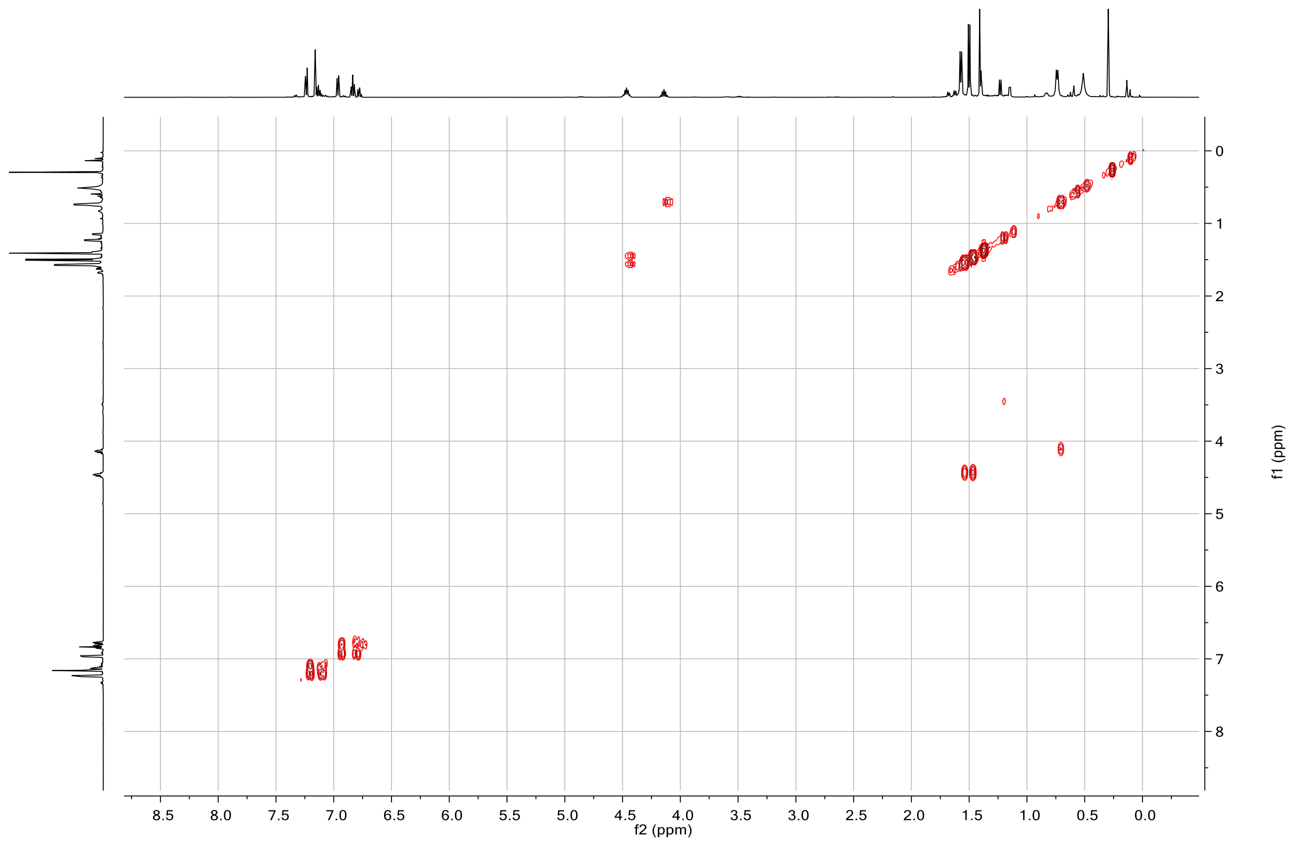

Figure S9. ^{1}H-^{13}C HSQC spectrum of **3**.

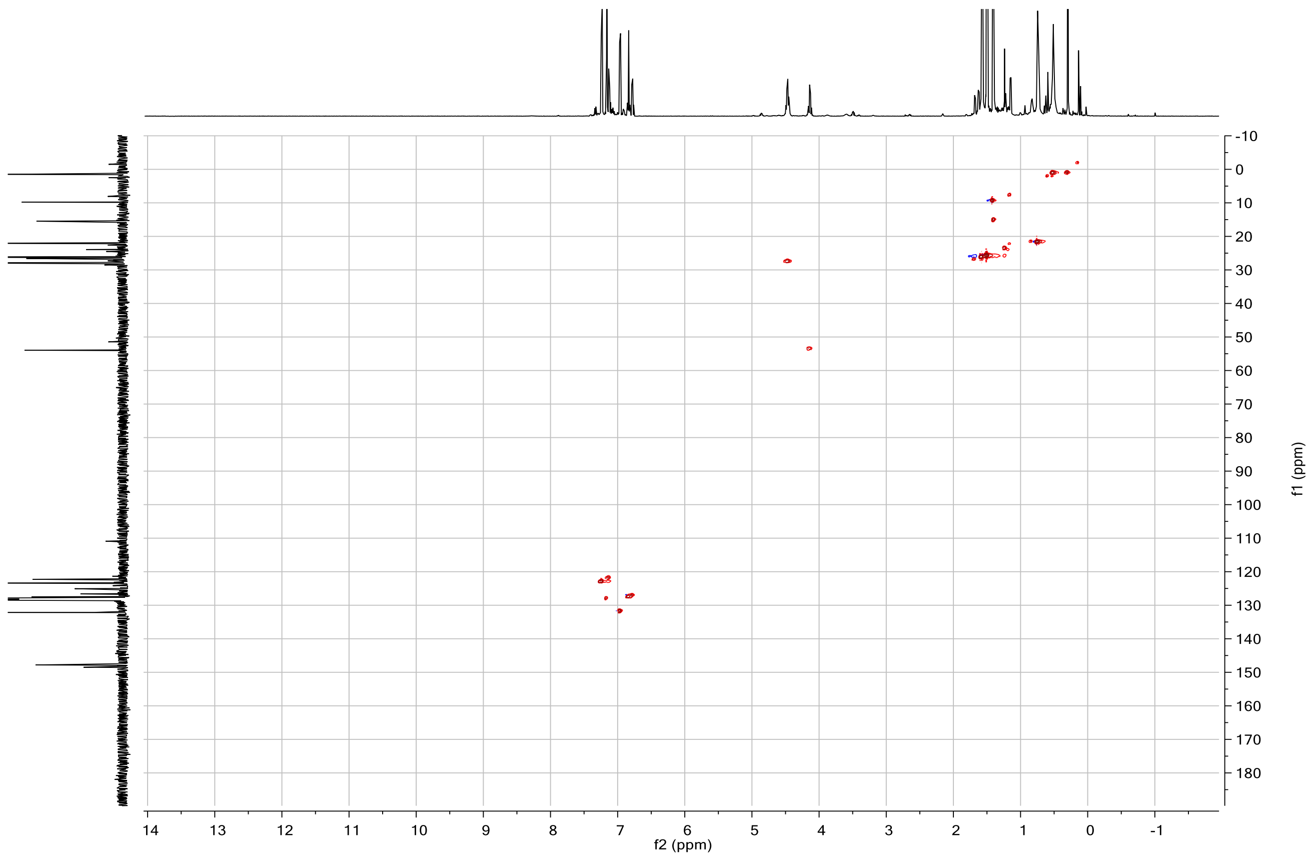

Figure S10. ^{1}H-^{13}C HMBC spectrum of **3**.

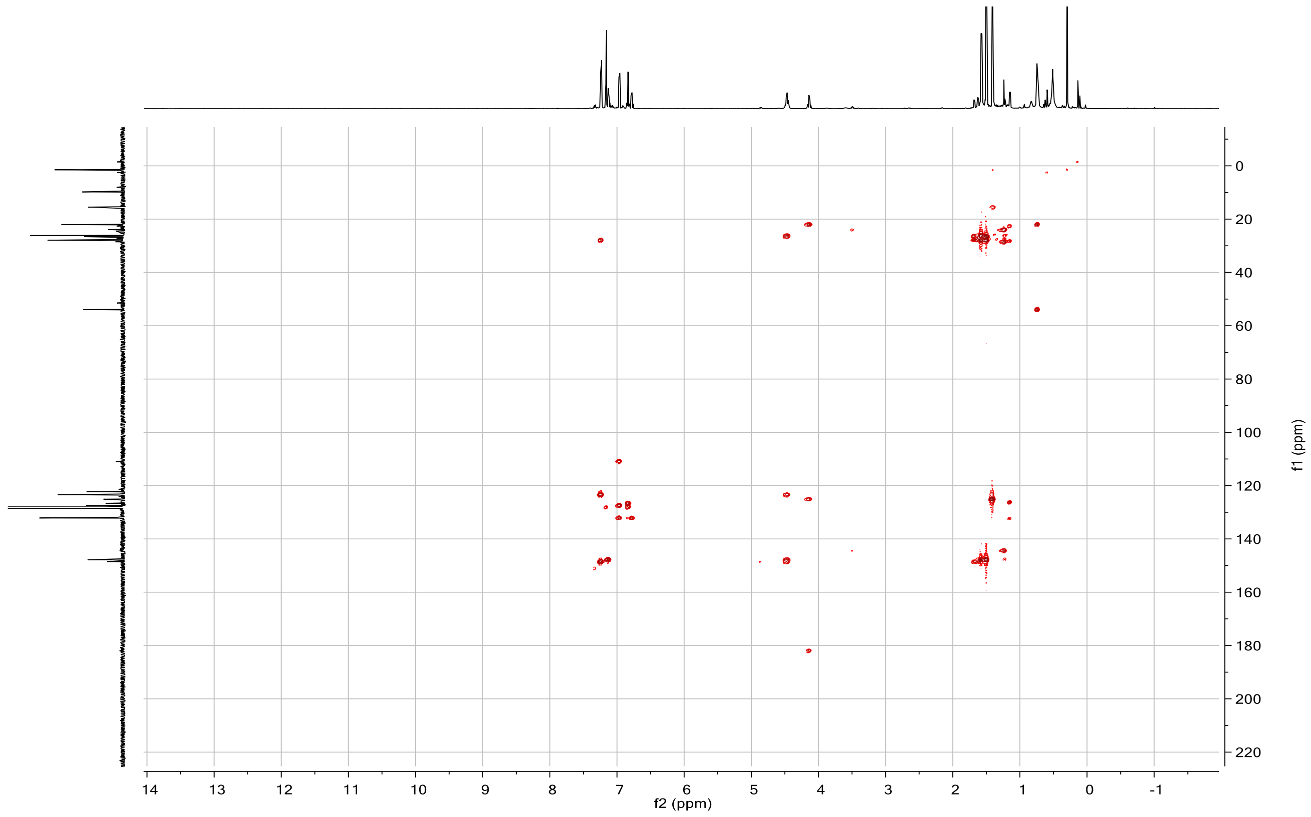

Heating reactions of [(^{Me2}CAAC)Cu-Al{SiN^{Dipp}}] with PhCCH; isolation of **5** and **6**

In a J Young's tube, phenylacetylene (7.6 mg, 8.2 μL, 0.075 mmol) was added via micropipette to a colourless solution of [(^{Me2}CAAC)Cu-Al{SiN^{Dipp}}] (**4**, 22 mg, 0.025 mmol) in C_6D_6. The colourless reaction mixture was then kept at 60°C overnight. Multiple species were observed in the crude reaction mixture by NMR spectroscopy along with some grey precipitates. The reaction mixture was then put under vacuum to remove all volatiles, and a toluene/hexane (2:1) solution of the reaction mixture was then kept at -30°C affording colourless single crystals of **5** along with some pale-green amorphous solids and black powders. The residual solution was then put under vacuum again to remove all volatiles, and slow evaporation of a hexane solution the residual solids at room temperature gives single crystals of **6** alongside dark red oil.

Synthesis of [(NHC^{iPr})Cu]…[(^{n}BuC^{H}C^H)(^{n}BuCC)-κ^2-C,C'-Al{SiN^{Dipp}}] (**7a**)

In a J Young's tube, 1-hexyne (4.1 mg, 5.7 μL, 0.05 mmol) was added via micropipette to a colourless solution of [(NHC^{iPr})Cu-Al{SiN^{Dipp}}] (**1**, 19 mg, 0.025 mmol) in C_6D_6. Quantitative generation of **7a** was observed by NMR spectroscopy within 30 mins at room temperature. The colourless solution was then put under vacuum to remove all volatiles and give compound **7a** as an off-white waxy solid. Yield 20.8 mg, 90%. No meaningful result of elemental analysis was obtained after several attempts. ^{1}H NMR (500 MHz, 298 K, Benzene-d_6) δ 7.20 – 7.17 (m, 2H, *m*-C_6H_3), 7.15 – 7.13 (m, 2H, *m*-C_6H_3), 7.07– 7.04 (m, 2H, *p*-C_6H_3), 5.11 (d, *J* = 20.8 Hz, 1H, AlC_2H_2), 4.48 (sept, *J* = 6.8 Hz, 2H, C*H*Me_2), 4.36 (sept, *J* = 6.8 Hz, 2H, C*H*Me_2), 4.29 (d, *J* = 20.8 Hz, 1H, AlC_2H_2), 4.16 (sept, *J* = 7.0 Hz, 2H, NC*H*Me_2), 1.97 – 1.92 (m, 3H, $(CH_2)_3CH_3$), 1.80 – 1.74 (m, 2H, $(CH_2)_3CH_3$), 1.58 (d, *J* = 6.8 Hz, 6H, CH*Me_2*), 1.52 (d, *J* = 6.8 Hz, 6H, CH*Me_2*), 1.48 (d, *J* = 6.8 Hz, 6H, CH*Me_2*), 1.47 (s, 6H, NC*Me*), 1.44 (d, *J* = 6.8 Hz, 6H, CH*Me_2*), 1.39 (s, 4H, SiCH_2), 1.27 – 1.21 (m, 4H, $(CH_2)_3CH_3$), 1.17 – 1.09 (m, 4H, $(CH_2)_3CH_3$), 1.04 (d, *J* = 7.0 Hz, 12H, NCH*Me_2*), 0.79 – 0.77 (m, 2H, $(CH_2)_3CH_3$), 0.75 – 0.72 (m, 3H, $(CH_2)_3CH_3$), 0.50 (s, 6H, Si*Me_2*), 0.46 (s, 6H, Si*Me_2*). ^{13}C NMR (126 MHz, 298 K, Benzene-d_6) δ 182.2 (Cu$C_{carbene}$), 149.4 (*i*-C_6H_3), 147.4 (*o*-C_6H_3), 147.0 (*o*-C_6H_3), 138.2 (AlC_2H_2), 124.7 (AlCC^nBu), 123.9 (NCMe), 123.4 (*m*-C_6H_3), 123.3 (*m*-C_6H_3), 122.3 (AlC_2H_2), 122.0 (*p*-C_6H_3), 112.5 (AlCC^nBu), 53.6 (NCHMe_2), 39.0 ($(CH_2)_3CH_3$), 32.0 ($(CH_2)_3CH_3$), 30.9 ($(CH_2)_3CH_3$), 28.0 (CHMe_2), 27.7 (CHMe2), 26.5 (CH*Me_2*), 26.2 (CH*Me_2*), 25.9 (CH*Me_2*), 24.5 (CH*Me_2*), 24.2 ($(CH_2)_3CH_3$), 24.0 ($(CH_2)_3CH_3$), 22.6 ($(CH_2)_3CH_3$), 22.4 (NCH*Me_2*), 15.5(SiCH_2), 14.2 ($(CH_2)_3CH_3$), 13.9 ($(CH_2)_3CH_3$), 9.8 (NC*Me*), 2.0 (Si*Me_2*), 1.0 (Si*Me_2*).

Figure S11. ^{1}H NMR (500 MHz, 298 K, d_6-benzene) spectrum of **7a**. *grease

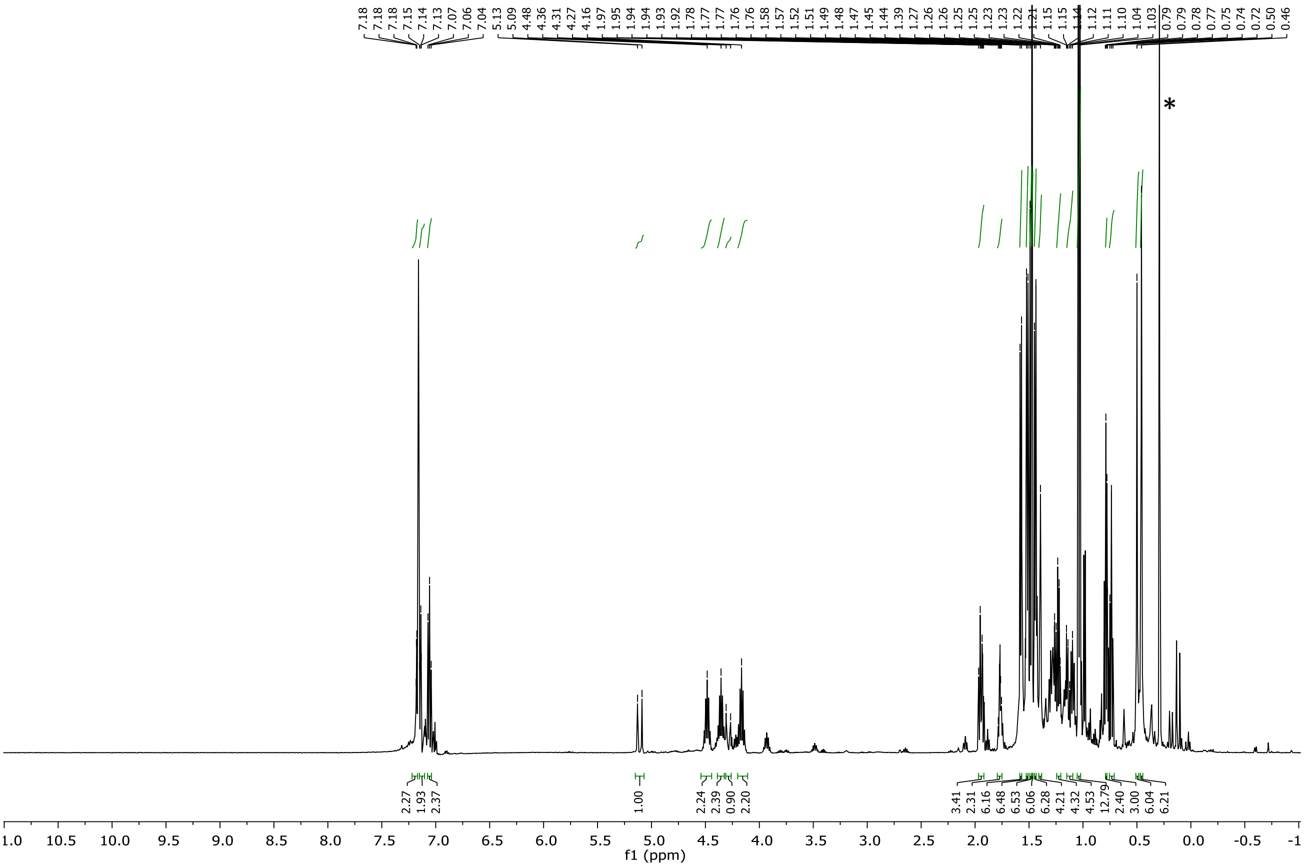

Figure S12. ^{13}C NMR (126 MHz, 298 K, d_6-benzene) spectrum of **7a**.

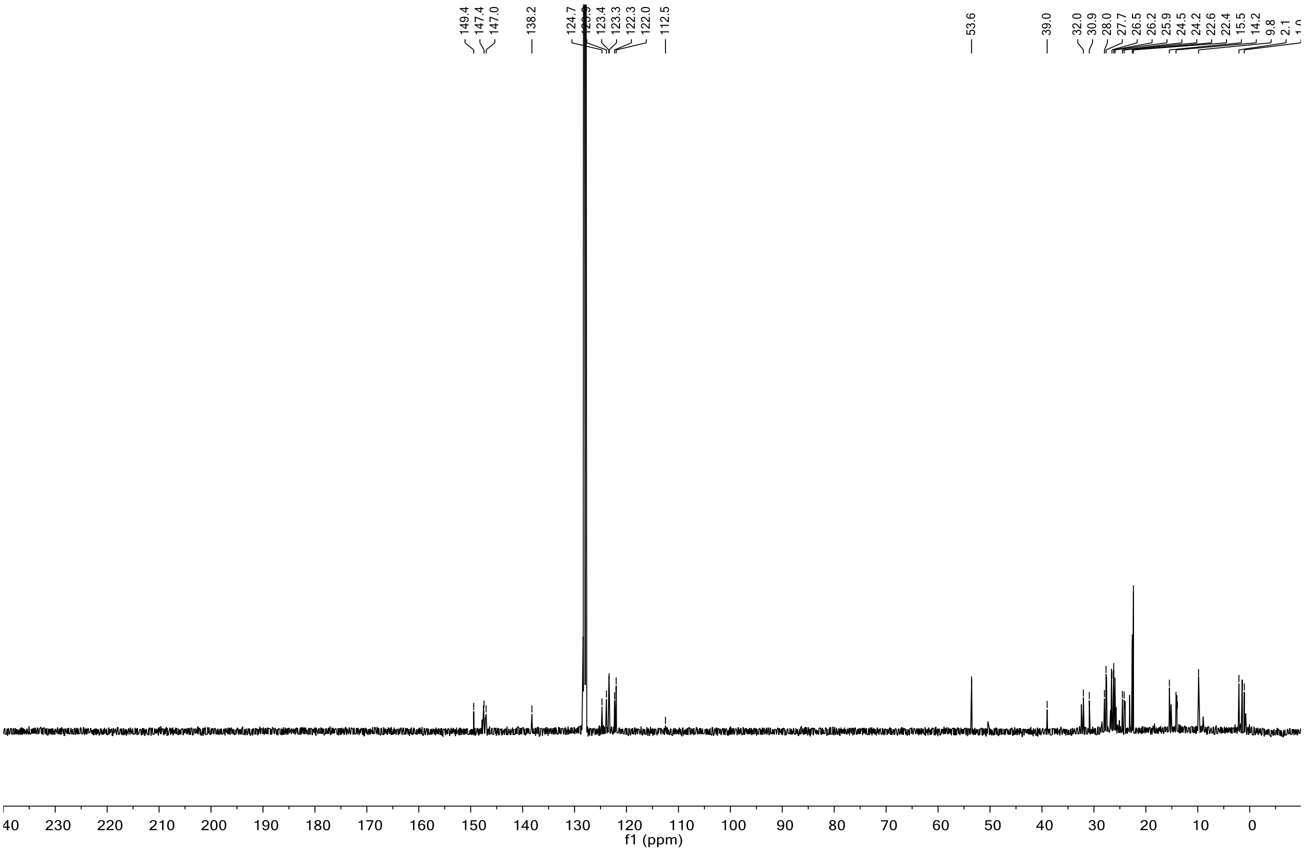

Figure S13. ^{1}H-^{1}H COSY spectrum of **7a**.

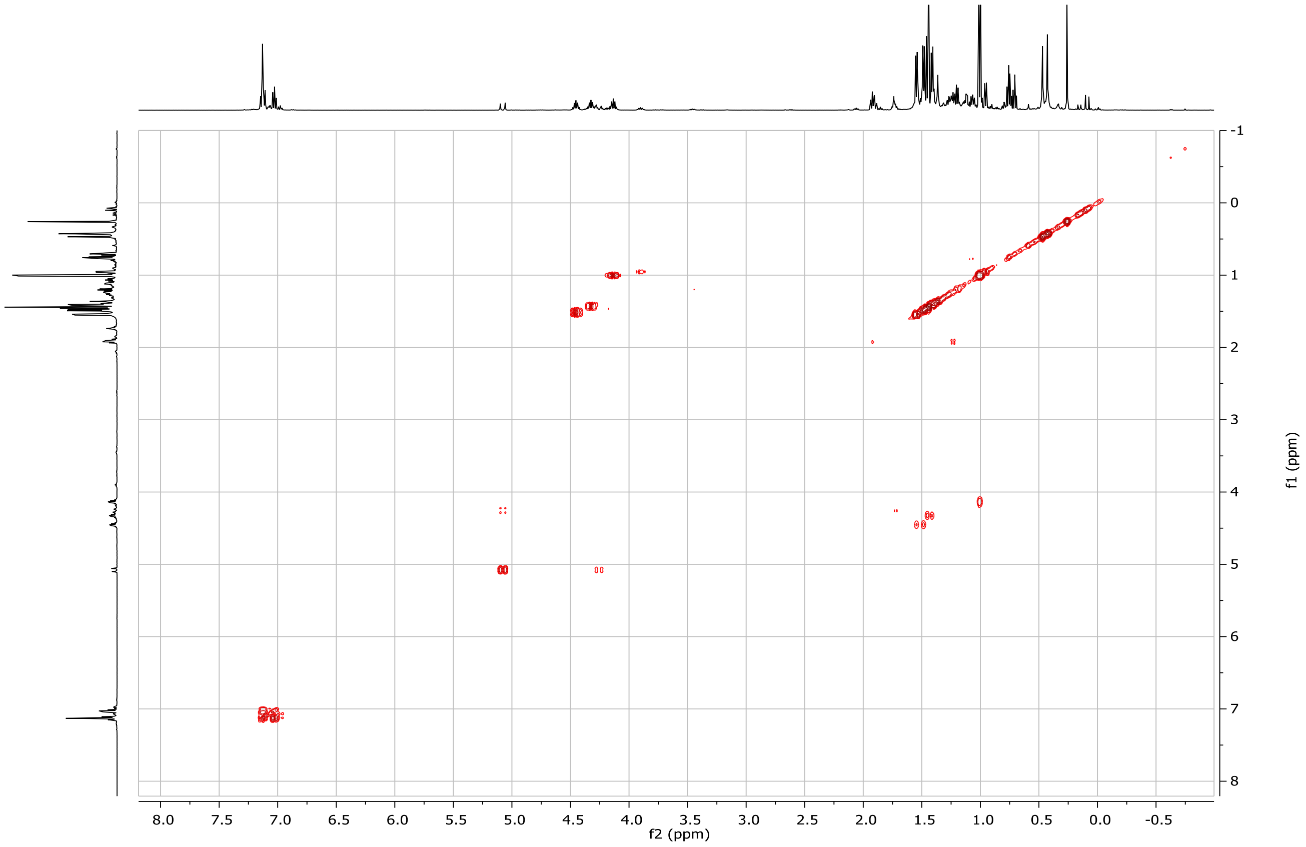

Figure S14. ^{1}H-^{13}C HSQC spectrum of **7a**.

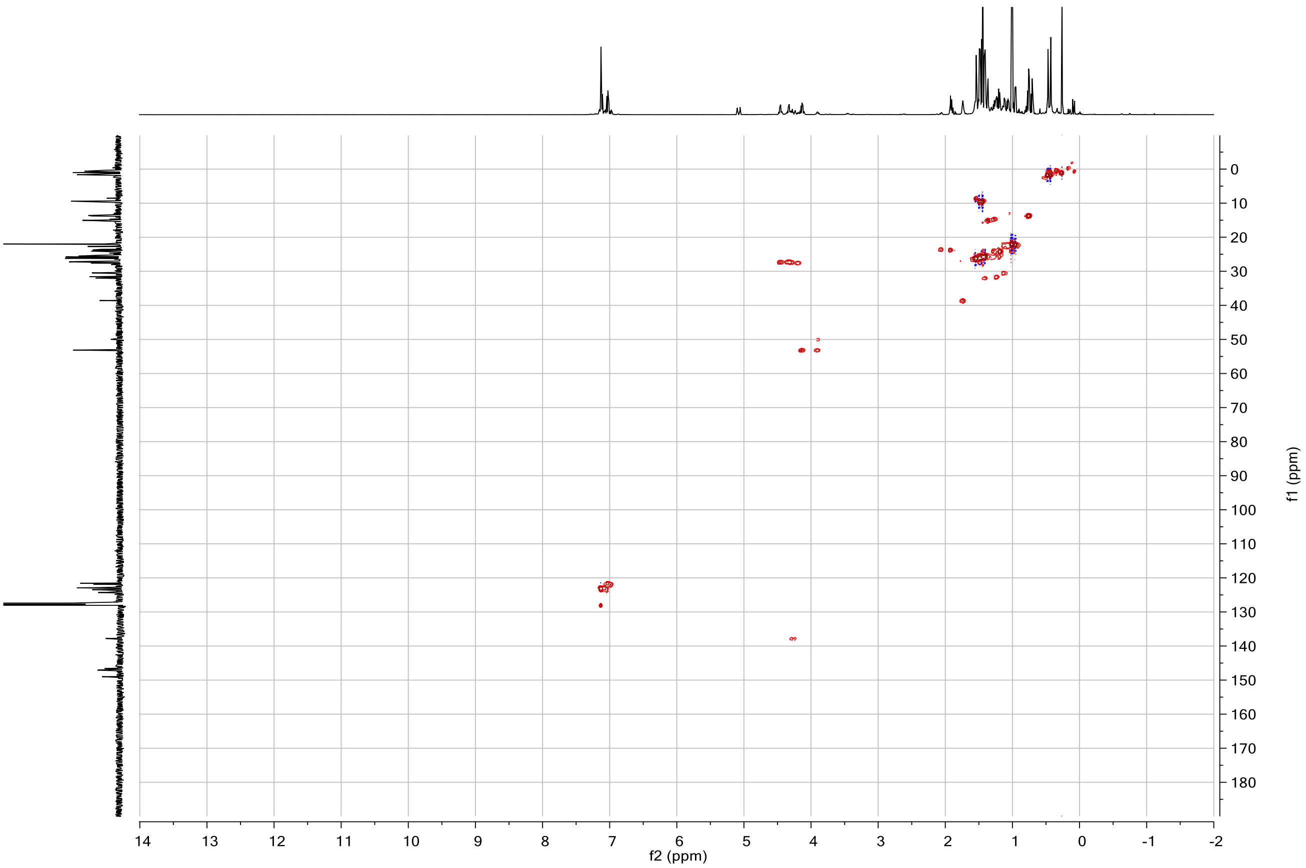

Figure S15. ^1H-^{13}C HMBC spectrum of **7a**.

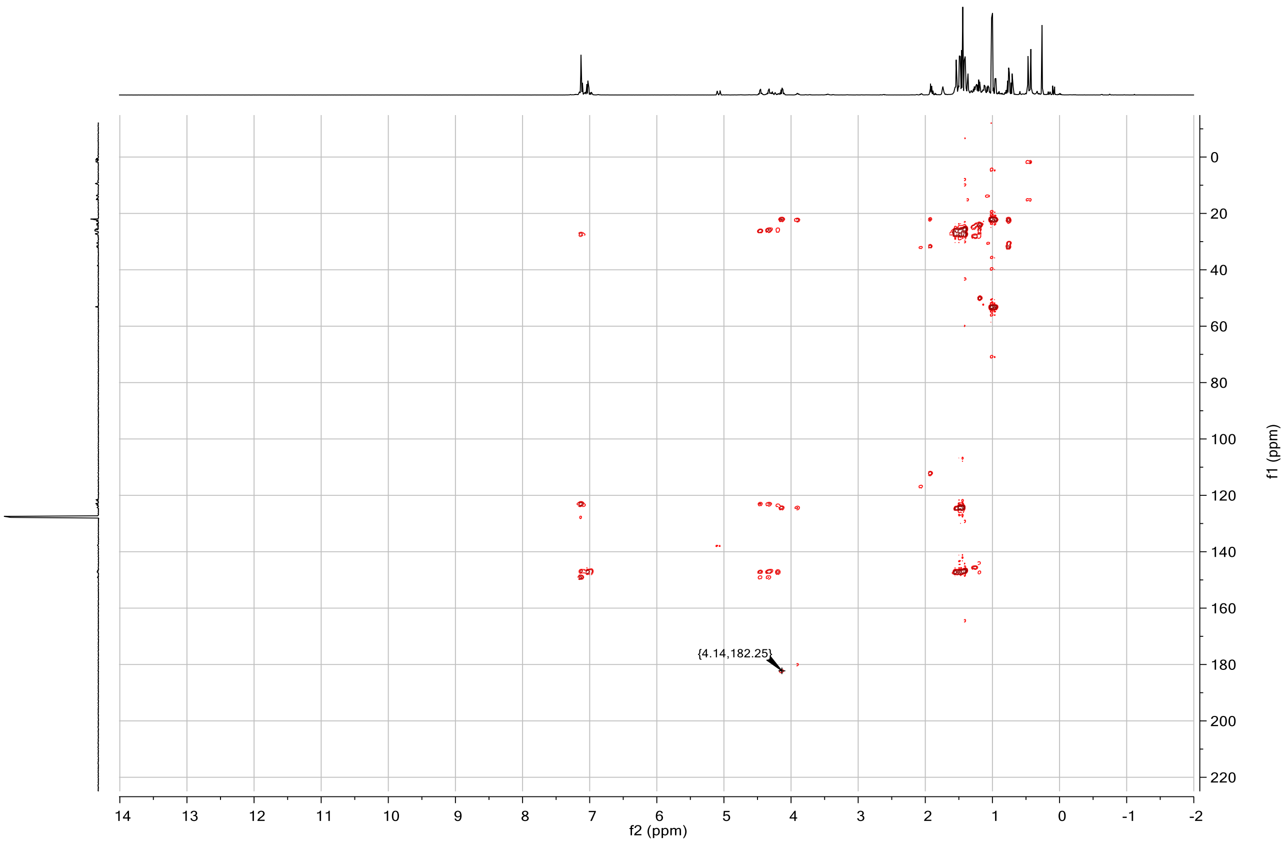

Synthesis of [(NHC^{iPr})Cu…(^{n}BuCC)$_2$-κ^2-C,C'-Al{SiN^{Dipp}}] (**7**)

In a J Young's tube, 1-hexyne (6.1 mg, 8.5 µL, 0.075 mmol) was added via micropipette to a colourless solution of [(NHC^{iPr})Cu-Al{SiN^{Dipp}}] (**1**, 19 mg, 0.025 mmol) in C_6D_6. The colourless reaction mixture was then kept at 60°C overnight before full conversion of **7** and generation of 1-hexene was confirmed by NMR spectroscopy. All volatiles were then removed *in vacuo*, affording **7** as a white powder. Yield 20 mg, 83%. Colourless single crystals suitable for X-ray crystallography was obtained by slow evaporation of a hexane solution of **7** at room temperature. No meaningful elemental analysis was obtained after several attempts. ^{1}H NMR (500 MHz, 298 K, Benzene-d_6) δ 7.18 – 7.15 (m, 4H, *m*-C_6H_3)*overlapping with C_6D_6, 7.07– 7.04 (m, 2H, *p*-C_6H_3), 4.39 (sept, *J* = 6.9 Hz, 4H, C*H*Me$_2$), 4.15 (sept, *J* = 7.2 Hz, 2H, NC*H*Me$_2$), 1.88 (t, *J* = 7.2 Hz, 4H, CCC*H*$_2$(CH$_2$)$_2$CH$_3$), 1.54 (d, *J* = 6.9 Hz, 12H, CH*Me*$_2$)*, 1.53 (s, 6H, NC*Me*)* *overlapping peaks, 1.51 (d, *J* = 6.9 Hz, 12H, CH*Me*$_2$), 1.34 (s, 4H, SiC*H*$_2$), 1.24 – 1.11 (m, 8H, CCCH$_2$(C*H*$_2$)$_2$CH$_3$), 1.02 (d, *J* = 7.2 Hz, 12H, NCH*Me*$_2$), 0.82 (t, *J* = 7.3 Hz, 6H, CCCH$_2$(CH$_2$)$_2$C*H*$_3$), 0.44 (s, 12H, SiMe$_2$). ^{13}C NMR (126 MHz, 298 K, Benzene-d_6) δ 183.2 (Cu*C*$_{carbene}$), 148.6 (*i*-*C*$_6$H$_3$), 147.5 (*o*-*C*$_6$H$_3$), 124.8 (N*C*Me), 123.3 (*m*-*C*$_6$H$_3$), 122.1 (*p*-*C*$_6$H$_3$), 109.9 (C*C*CH$_2$(CH$_2$)$_2$CH$_3$), 53.6 (N*C*HMe$_2$), 32.1 (CC*C*H$_2$(CH$_2$)$_2$CH$_3$), 27.7 (*C*H*Me*$_2$), 26.7 (CH*Me*$_2$), 26.1 (CH*Me*$_2$), 24.1 (CCCH$_2$(*C*H$_2$)$_2$CH$_3$), 22.5 (NCH*Me*$_2$), 22.2 (CCCH$_2$(*C*H$_2$)$_2$*C*H$_3$), 15.5 (Si*C*H$_2$), 14.1 (CCCH$_2$(CH$_2$)$_2$*C*H$_3$), 9.9 (NC*Me*), 1.7 (Si*Me*$_2$). ^{13}C resonance correlated to Al*C*C(CH$_2$)$_3$CH$_3$ not observed.

Figure S16. ^{1}H NMR (500 MHz, 298 K, d_6-benzene) spectrum of **7**. *grease

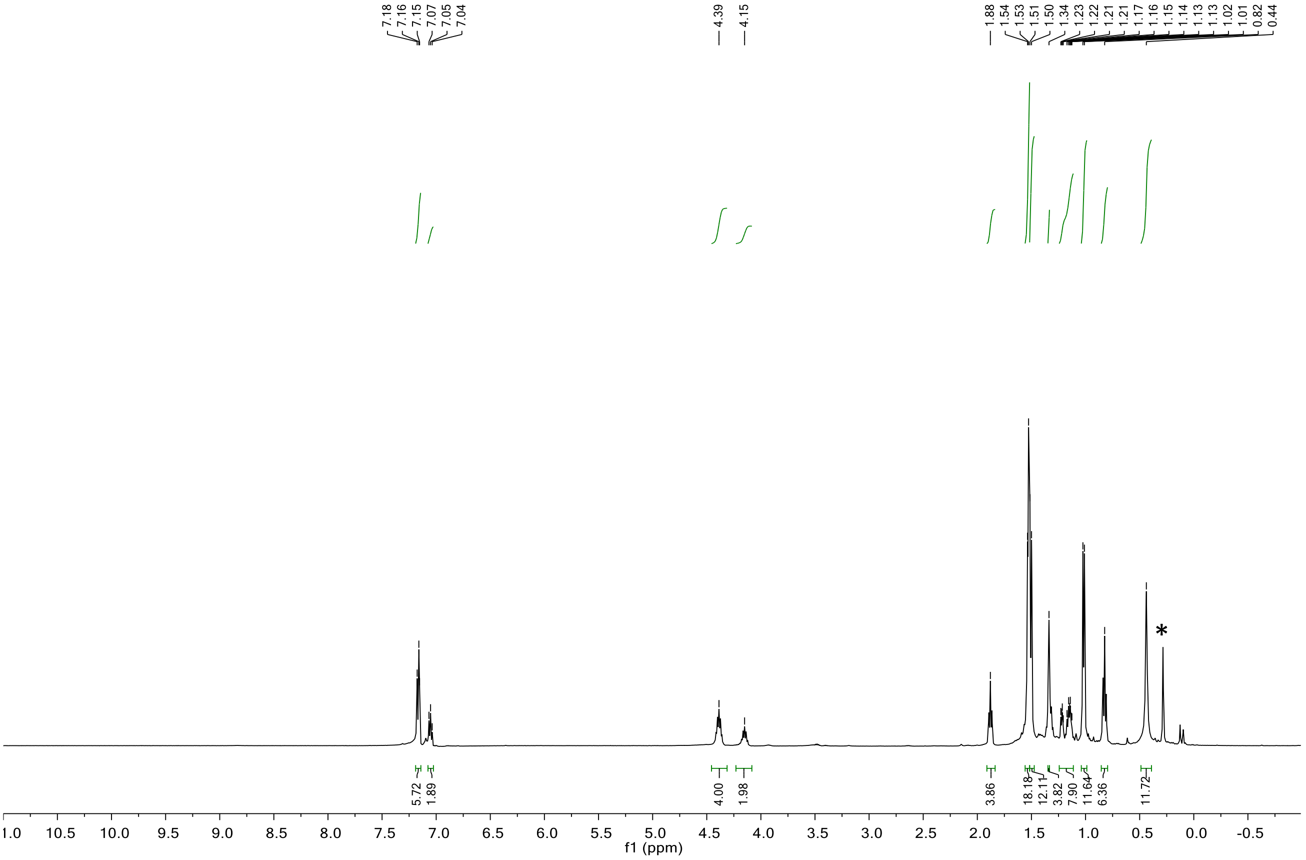

Figure S17. ^{13}C NMR (126 MHz, 298 K, d_6-benzene) spectrum of **7**.

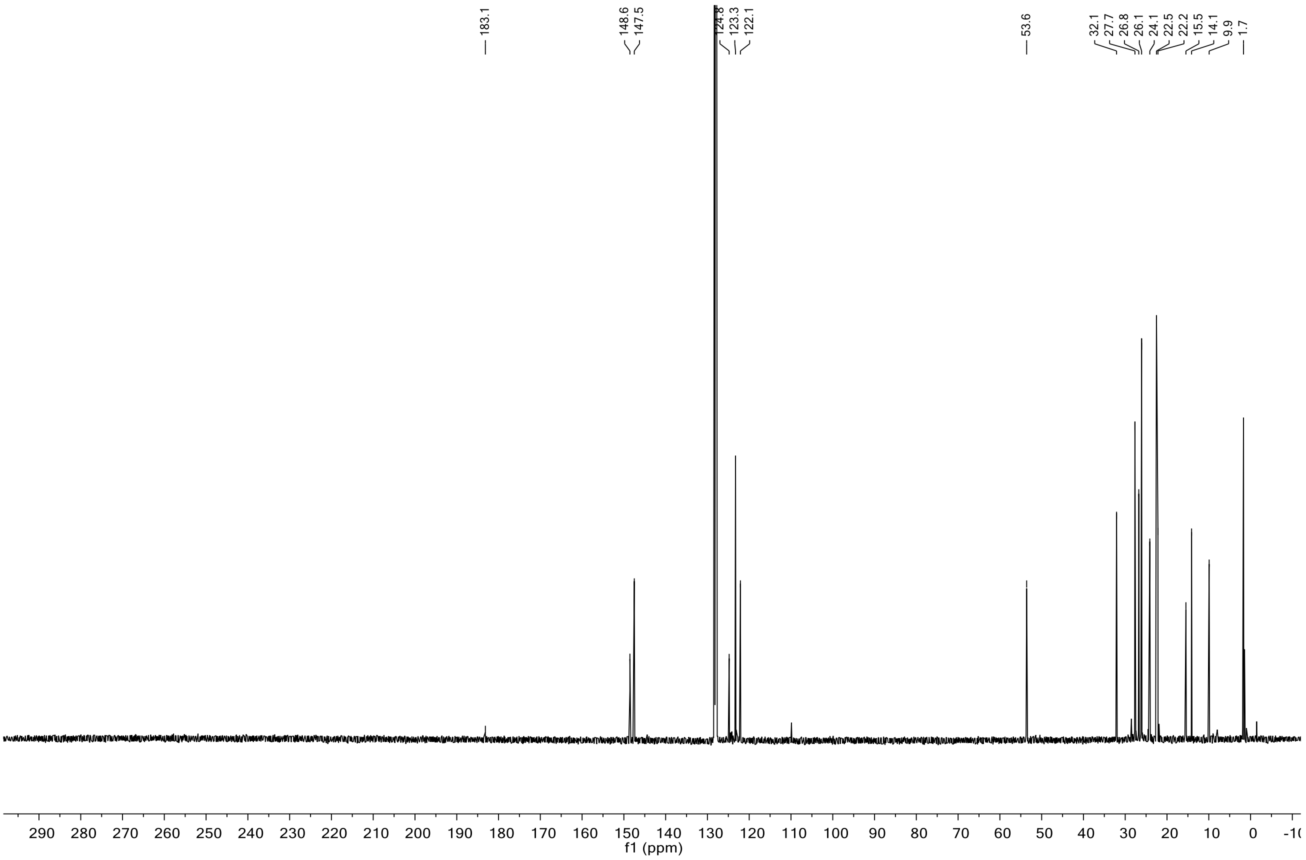

Figure S18. ^1H-^1H COSY spectrum of **7**.

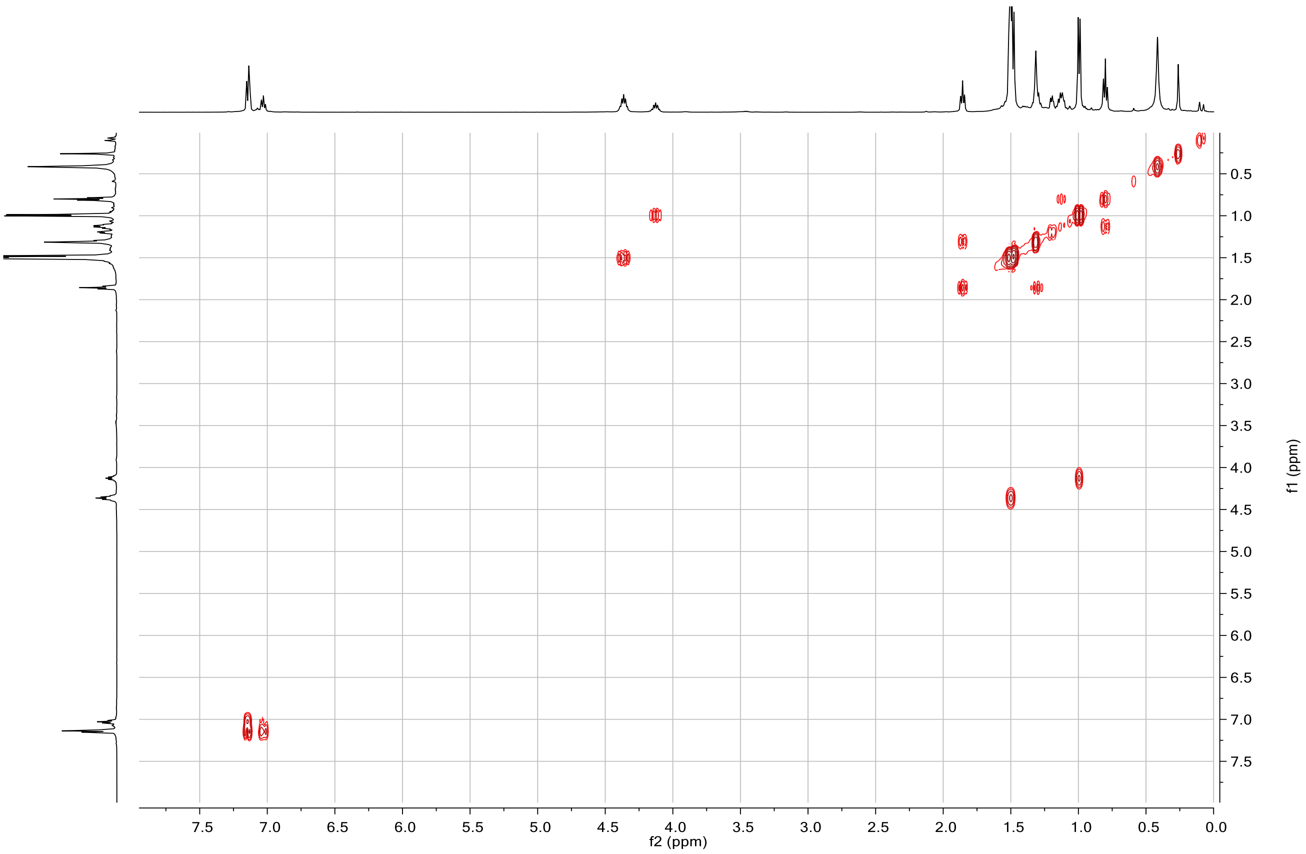

Figure S19. ^1H-^{13}C HSQC spectrum of **7**.

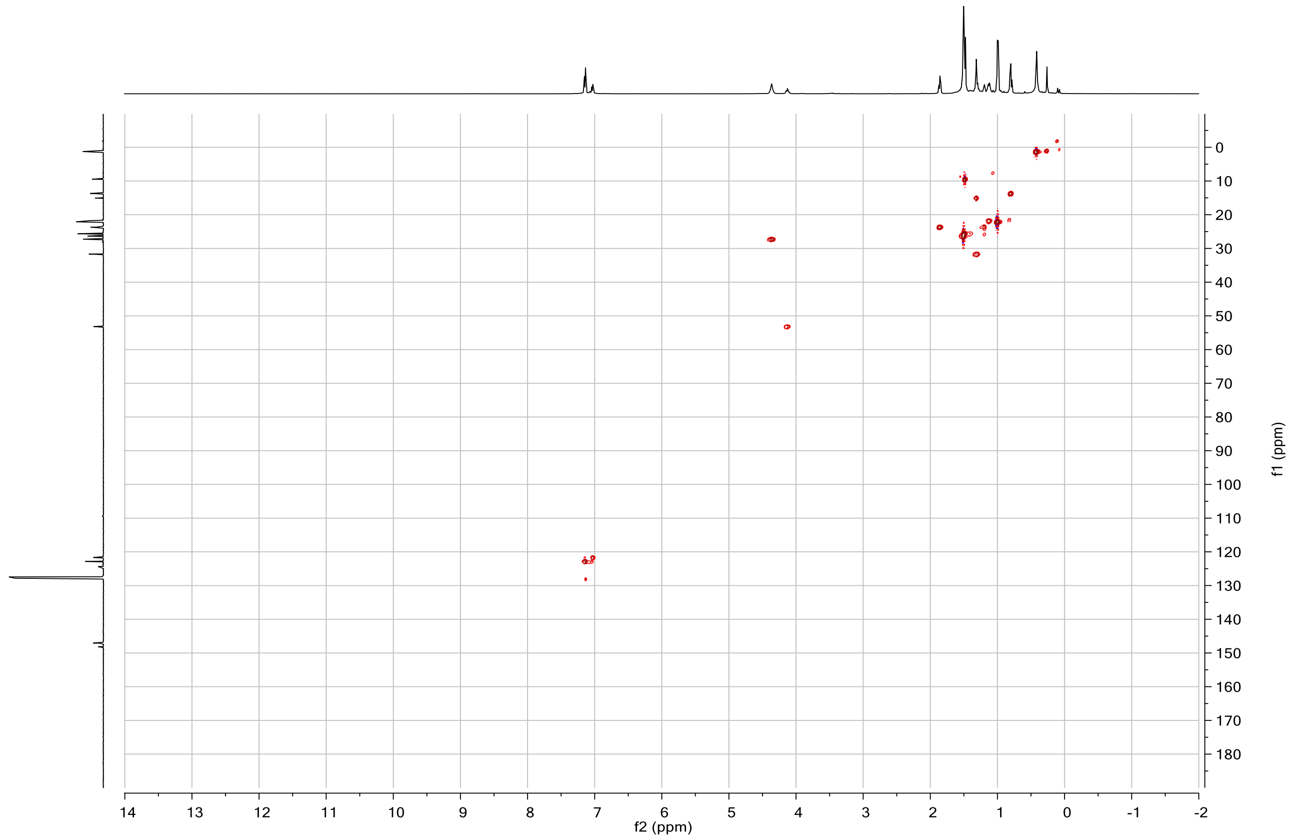

Figure S20 ^{1}H-^{13}C HMBC spectrum of **7**.

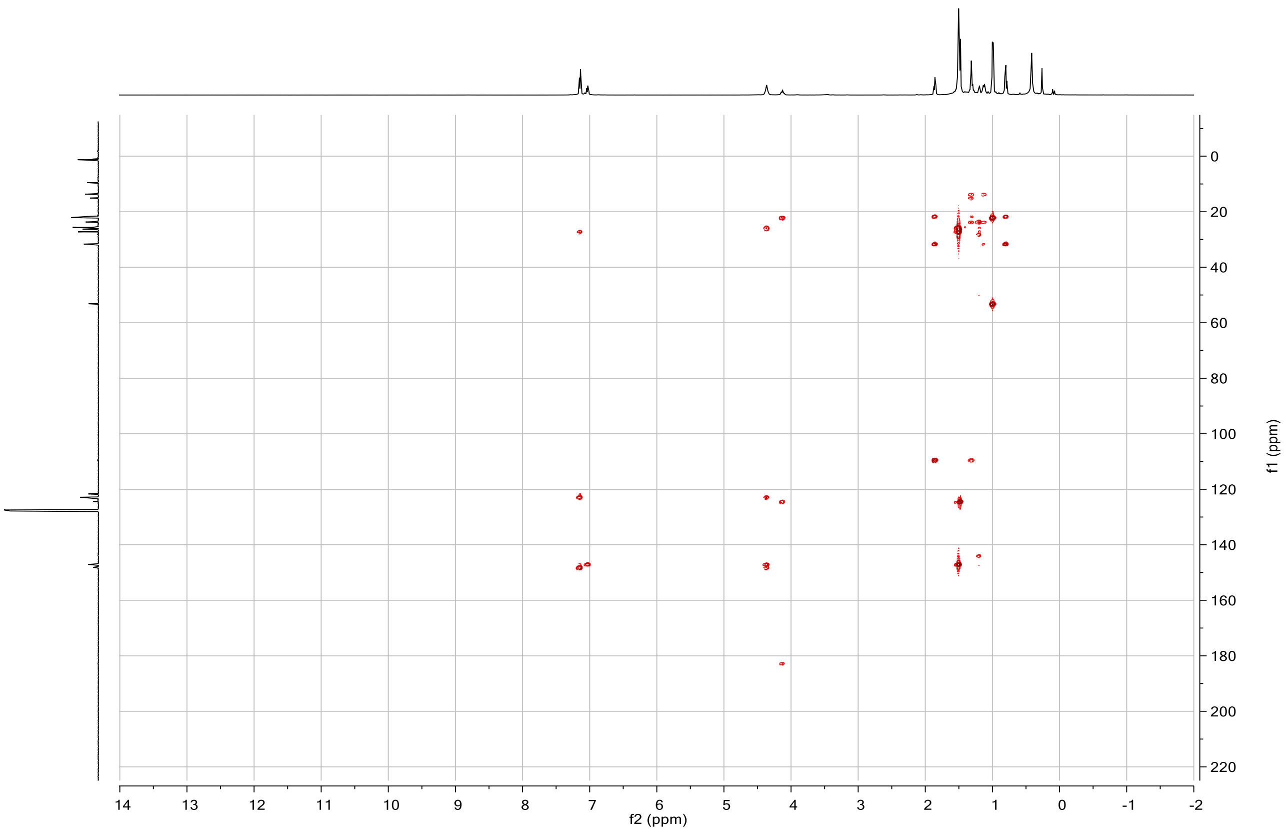

Synthesis of [(NHC^{iPr})Cu…(Me$_3$SiCC)$_2$-κ^2-C,C'-Al{SiN^{Dipp}}] (**8**)

In a J Young's tube, ethynyltrimethylsilane (6.1 mg, 9.2 μL, 0.075 mmol) was added via micropipette to a colourless solution of [(NHC^{iPr})Cu-Al{SiN^{Dipp}}] (**1**, 19 mg, 0.025 mmol) in C_6D_6. The colourless reaction mixture was then kept at 60°C for three days before full conversion of **8** and generation of corresponding alkene was confirmed by NMR spectroscopy. The colourless solution was then put under vacuum to remove all volatiles and give compound **8** as a white powder. Single crystals suitable for X-ray crystallography was obtained by slow evaporation a hexane solution at room temperature. Yield 21 mg, 84 %. Anal Calc'd for $C_{51}H_{89}AlCuN_4Si_4$ (**8**, 961.17) C, 63.73; H, 9.33; N, 5.83 %. Found: C, 63.40; H, 8.90; N, 5.89 %.^{1}H NMR (500 MHz, 298 K, Benzene-d_6) δ 7.12 (d, J = 7.5 Hz, 4H, *m*-C_6H_3), 7.04 (t, J = 7.5 Hz, 2H, *p*-C_6H_3), 4.31 (sept, J = 6.8 Hz, 4H, C*H*Me$_2$), 4.24 (sept, J = 7.0 Hz, 2H, NC*H*Me$_2$), 1.55, (s, 6H, NC*Me*), 1.54 (d, J = 6.8Hz, 12H, CH*Me*$_2$) *overlapping peaks, 1.46 (d, J = 6.8 Hz, 12H, CH*Me*$_2$), 1.36 (s, 4H, SiC*H*$_2$), 1.07 (d, J = 7.0 Hz, 12H, NCH*Me*$_2$), 0.43 (s, 12H, Si*Me*$_2$), -0.09 (s, 18H, Si*Me*$_3$). ^{13}C{^{1}H} NMR (126 MHz, 298 K, Benzene-d_6) δ 182.6 (Cu$C_{carbene}$), 148.2 (*i*-C_6H_3), 147.1 (*o*-C_6H_3), 125.5 (N*C*Me), 123.3 (*m*-C_6H_3), 122.2 (*p*-C_6H_3), 111.3 (AlC*C*SiMe$_3$), 53.6 (N*C*HMe$_2$), 27.5 (*C*HMe$_2$), 27.3 (CH*Me*$_2$), 26.1 (CH*Me*$_2$), 22.7 (NCH*Me*$_2$), 15.4 (Si*C*H$_2$), 10.0 (NC*Me*), 1.7 (Si*Me*$_2$), 1.3 (Si*Me*$_3$). ^{13}C resonance correlated to Al*C*CSiMe$_3$ was not observed.

Figure S21. ^{1}H NMR (500 MHz, 298 K, d_6-benzene) spectrum of **8**. *grease

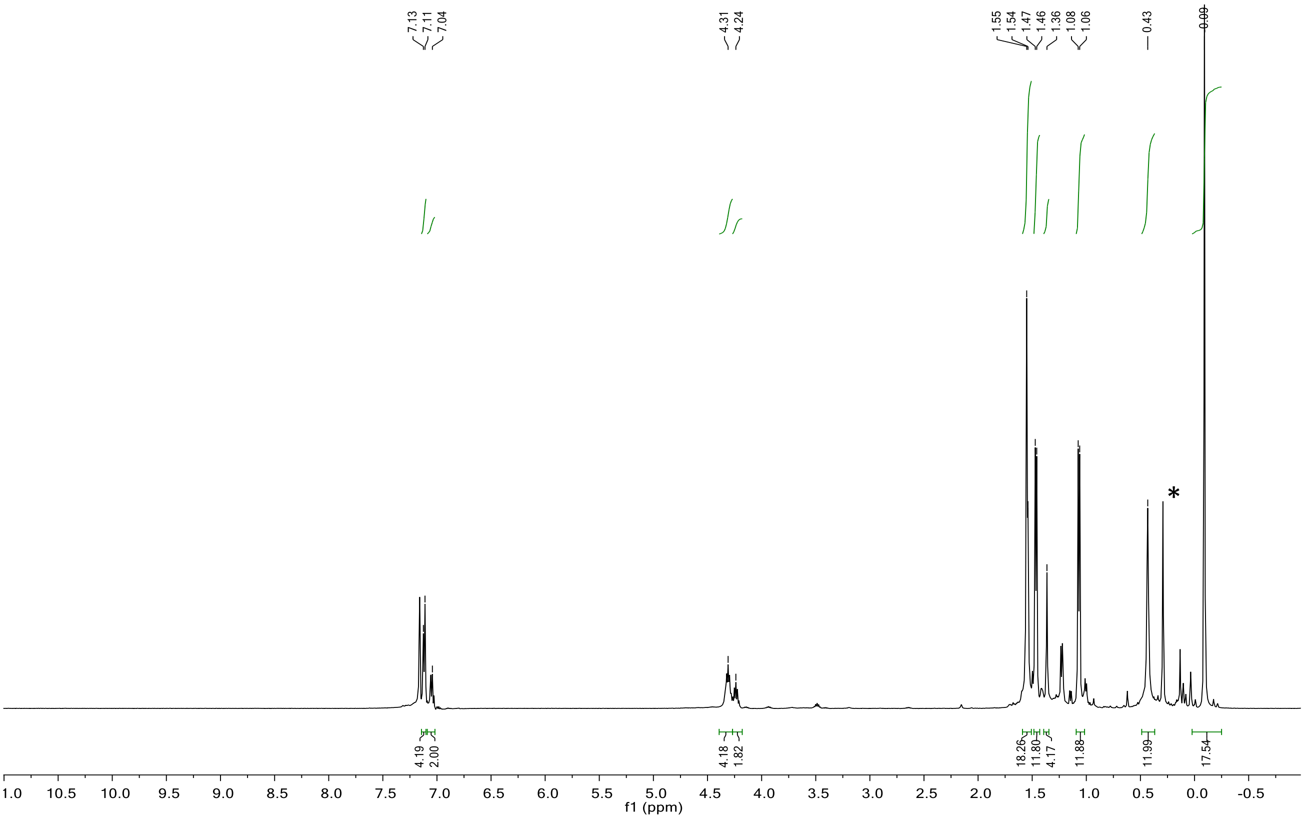

Figure S22. ^{13}C NMR (126 MHz, 298 K, d_6-benzene) spectrum of **8**. *grease

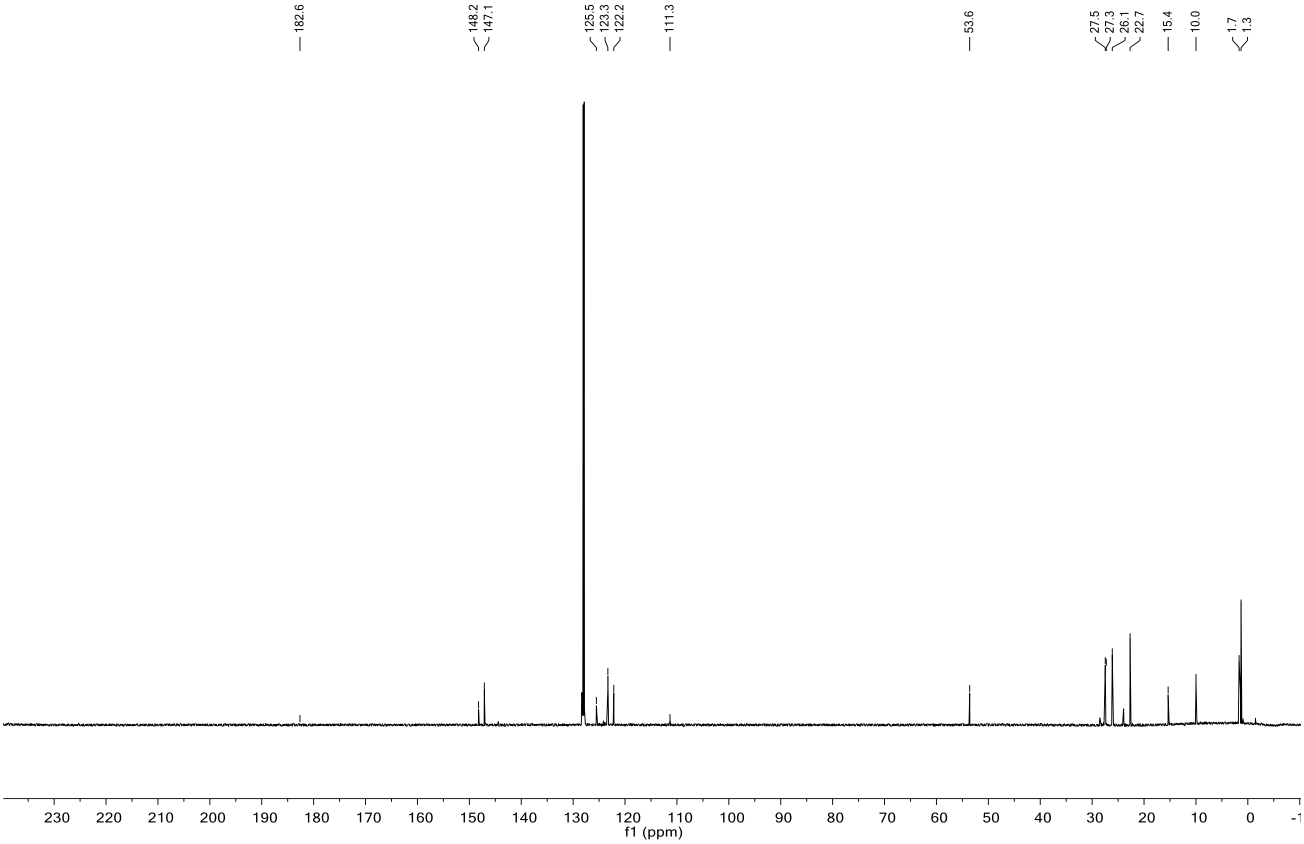

Figure S23. ^{1}H-^{1}H COSY spectrum of **8**.

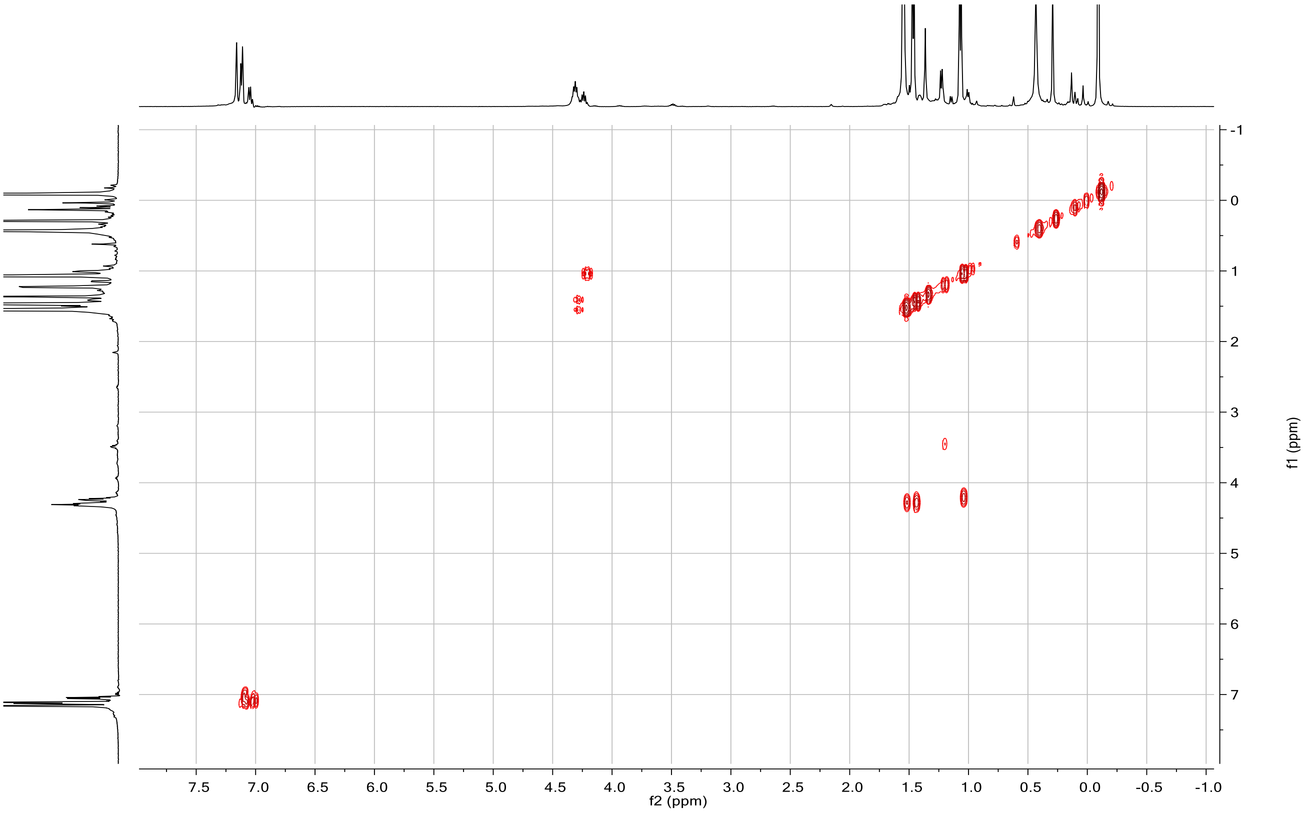

Figure S24. ^{1}H-^{13}C HSQC spectrum of **8**.

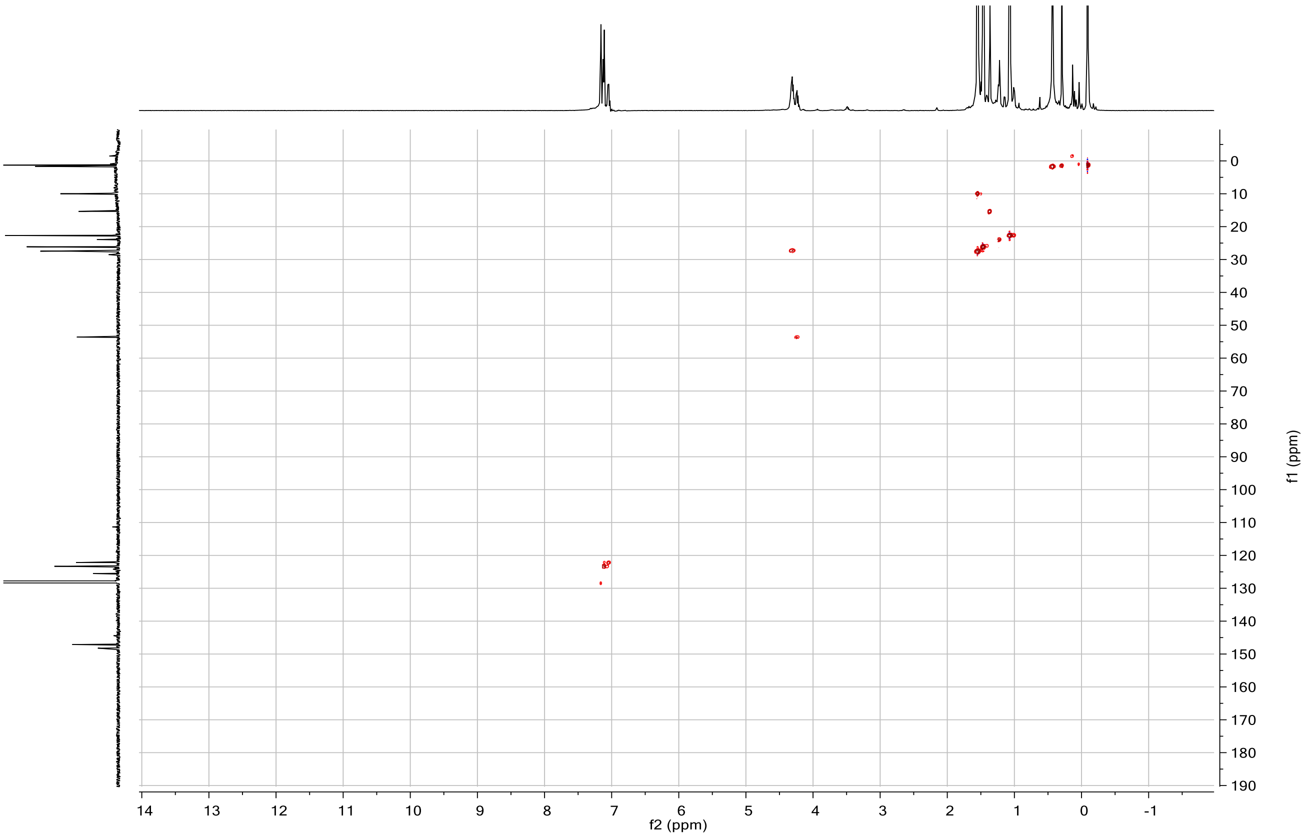

Figure S25. ^{1}H-^{13}C HMBC spectrum of **8**.

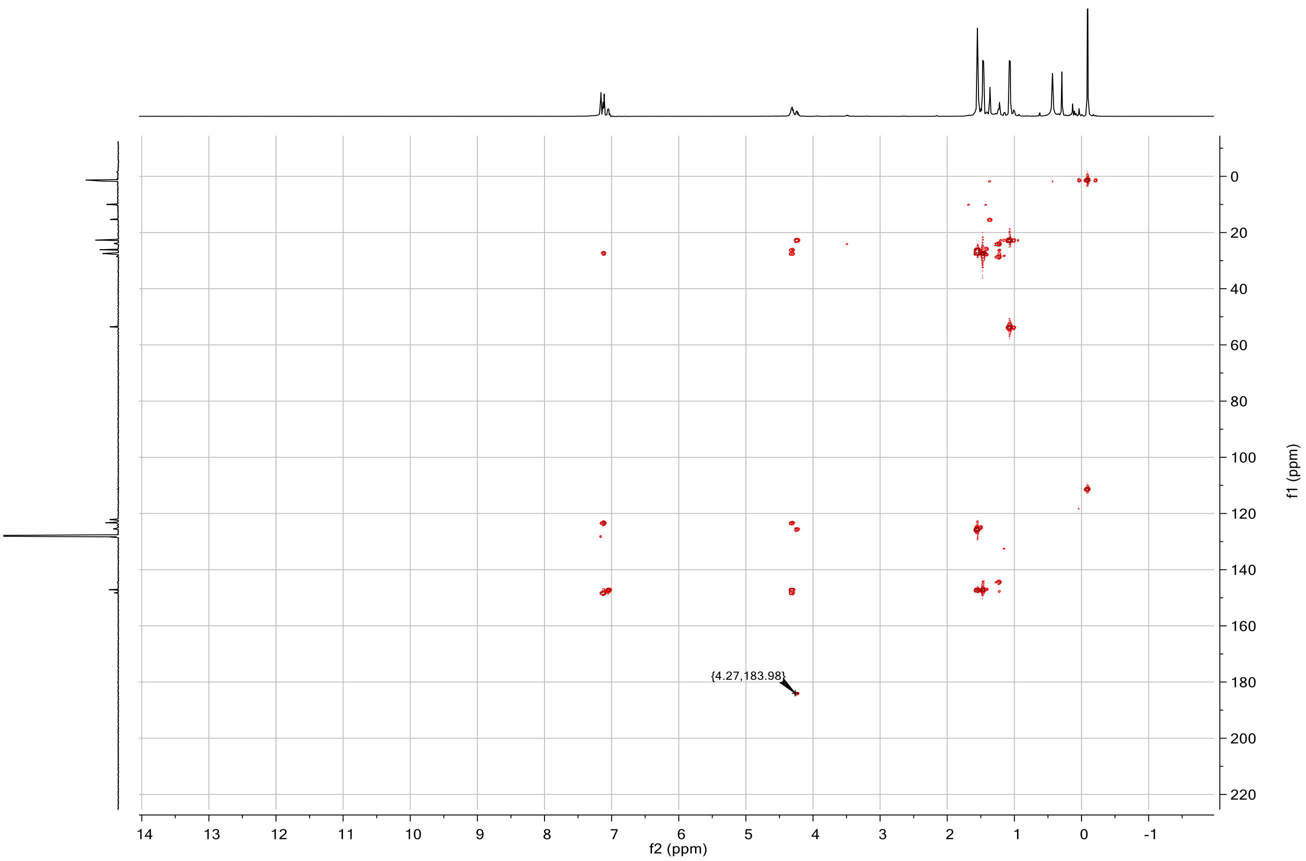

Synthesis of [(NHC^{iPr})Cu…(^{t}BuCC)$_2$-κ^2-C,C'-Al{SiN^{Dipp}}] (**9**)

In a J Young's tube, 3,3-dimethyl-1-butyne (6.1 mg, 9.2 μL, 0.075 mmol) was added via micropipette to a colourless solution of [(NHC^{iPr})Cu-Al{SiN^{Dipp}}] (**1**, 19 mg, 0.025 mmol) in C_6D_6. The colourless reaction mixture was then kept at 60°C overnight before full conversion of **9** and generation of 3,3-dimethyl-1-butene was confirmed by NMR spectroscopy. The colourless solution was then put under vacuum to remove all volatiles and give compound **9** as a pale-yellow waxy solid. Yield 19 mg, 76 %. No meaningful result of elemental analysis was obtained after several attempts. ^{1}H NMR (500 MHz, 298 K, Benzene-d_6) δ 7.21 (m, 2H, *p*-C_6H_3), 7.13 – 7.03 (m, 4H, *m*-C_6H_3), 4.40 (sept, *J* = 7.0 Hz, 4H, C*H*Me$_2$ on SiN^{Dipp}), 3.92 (sept, *J* = 6.5 Hz, 1H, NC*H*Me$_2$), 3.79 (sept, *J* = 6.5 Hz, 1H, NC*H*Me$_2$), 1.59 (d, *J* = 7.0 Hz, 12H, CH*Me*$_2$ on SiN^{Dipp}), 1.54 – 1.49 (m, 12H, CH*Me*$_2$ on SiN^{Dipp}), 1.39 (s, 4H, SiC*H*$_2$), 1.23 (d, *J* = 6.5 Hz, 12H, NC*H*Me$_2$), 1.07 (s, 3H, NC*Me*), 1.06 (s, 3H, NC*Me*), 0.97 (s, 18H, C*Me*$_3$), 0.60 (s, 6H, Si*Me*$_2$), 0.43 (s, 6H, Si*Me*$_2$). ^{13}C NMR (126 MHz, 298 K, Benzene-d_6) δ 167.3 ($C_{carbene}$), 148.8 (*i*-C_6H$_3$), 147.5 (*o*-C_6H$_3$), 145.9 (*o*-C_6H$_3$), 125.1 (NCMe), 124.0 (*m*-C_6H$_3$), 123.7 (*p*-C_6H$_3$), 122.1 (*m*-C_6H$_3$), 120.5 (AlC*C*Me$_3$), 53.8 (*C*Me$_3$), 50.4 (*C*Me$_3$), 31.8 (C*Me*$_3$), 28.2 (N*C*HMe$_2$), 27.3 (*C*HMe$_2$), 26.1 (CH*Me*$_2$), 25.1 (CH*Me*$_2$), 24.5 (CH*Me*$_2$), 22.6 (NCH*Me*$_2$), 15.1 (Si*C*H$_2$), 10.0 (NC*Me*), 9.0 (NC*Me*), 2.6 (Si*Me*$_2$), 0.1 (Si*Me*$_2$). ^{13}C resonance correlates to Al*C*CMe$_3$ was not observed.

Figure S26. ^{1}H NMR (500 MHz, 298 K, d_6-benzene) spectrum of **9**. *grease

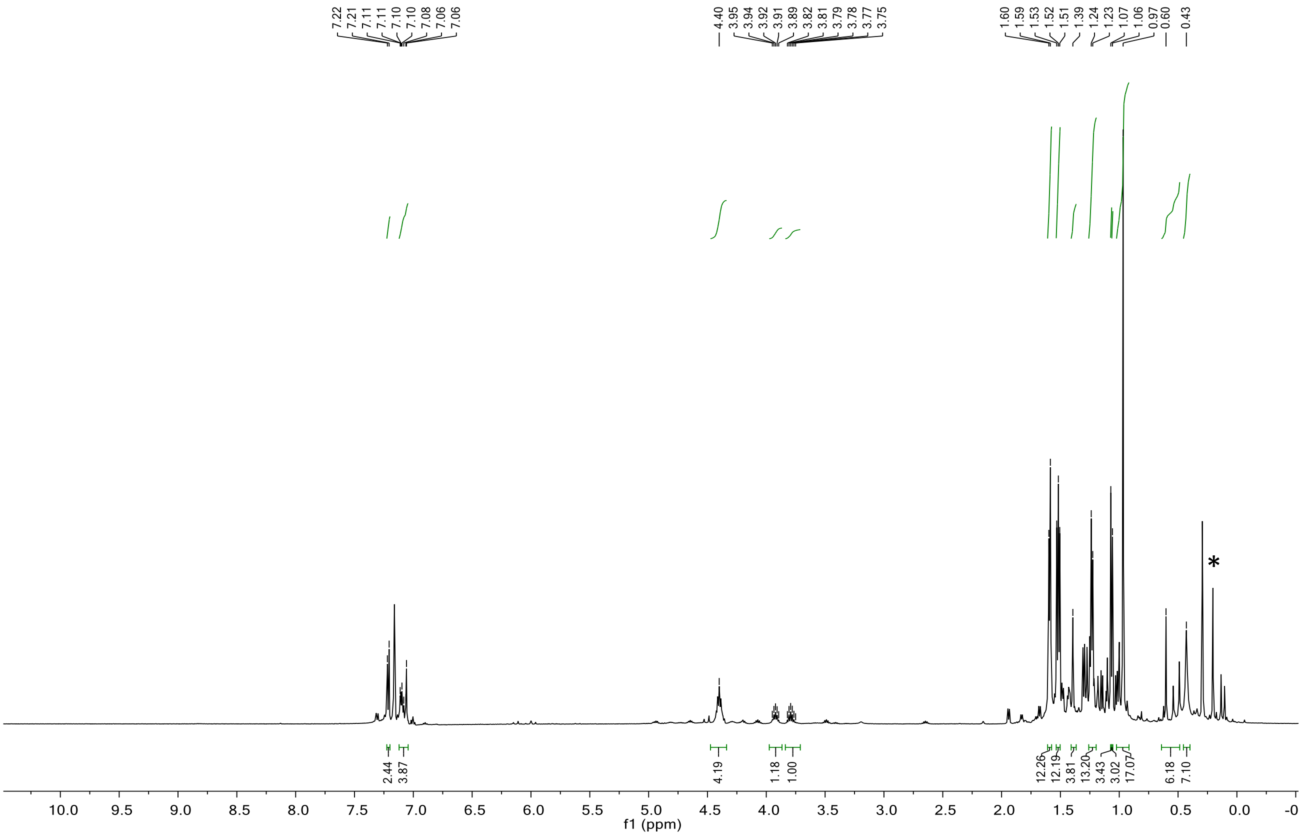

Figure S27. ^{13}C NMR (126 MHz, 298 K, d_6-benzene) spectrum of **9**. *grease

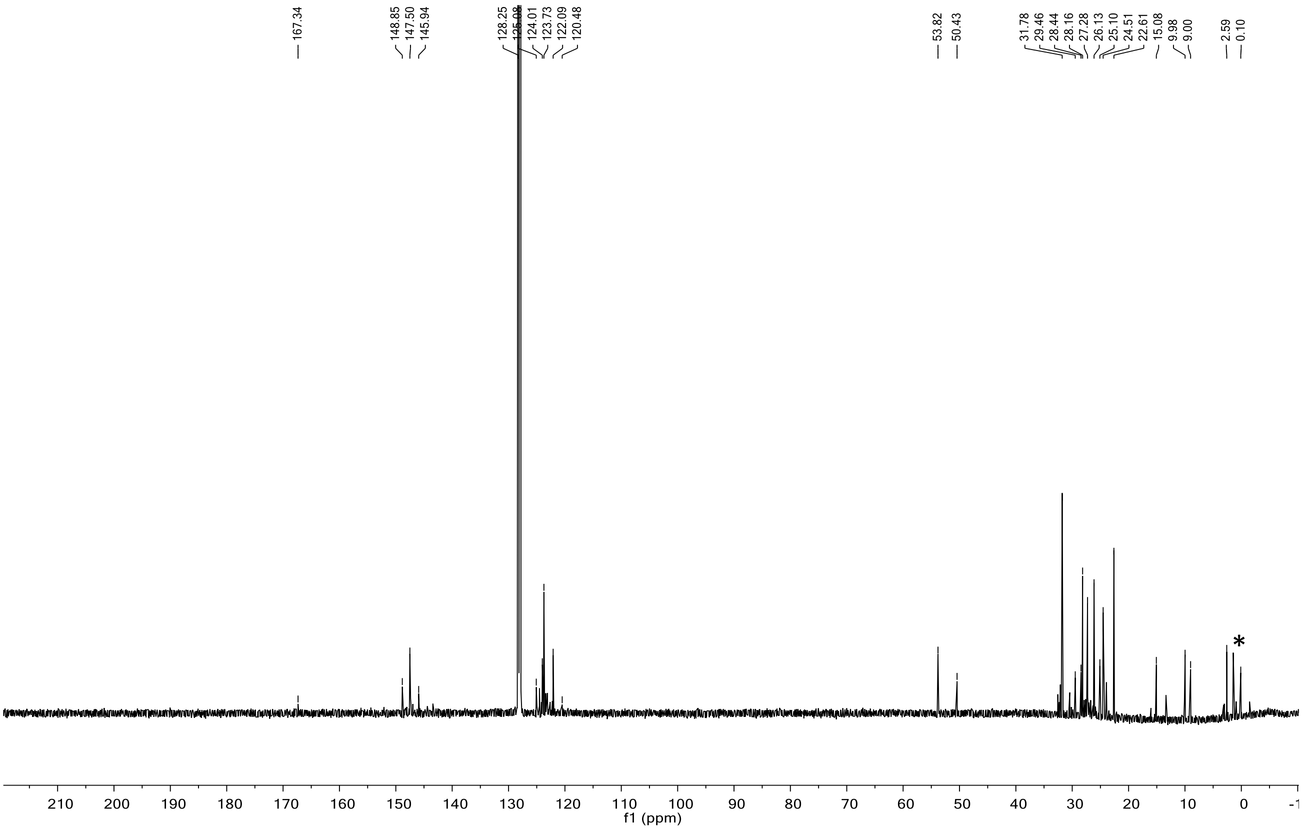

Figure S28. ^{1}H-^{1}H COSY spectrum of **9**.

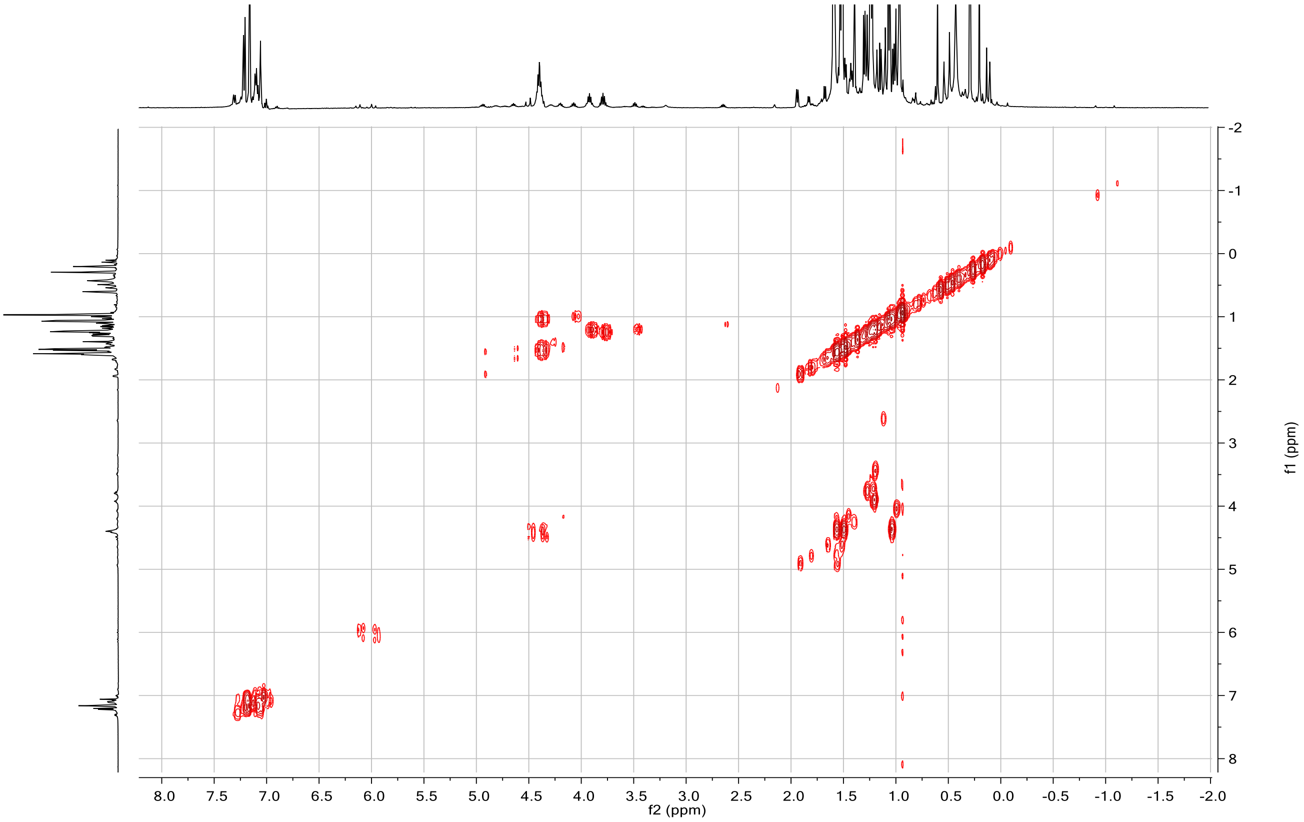

Figure S29. ^{1}H-^{13}C HSQC spectrum of **9**.

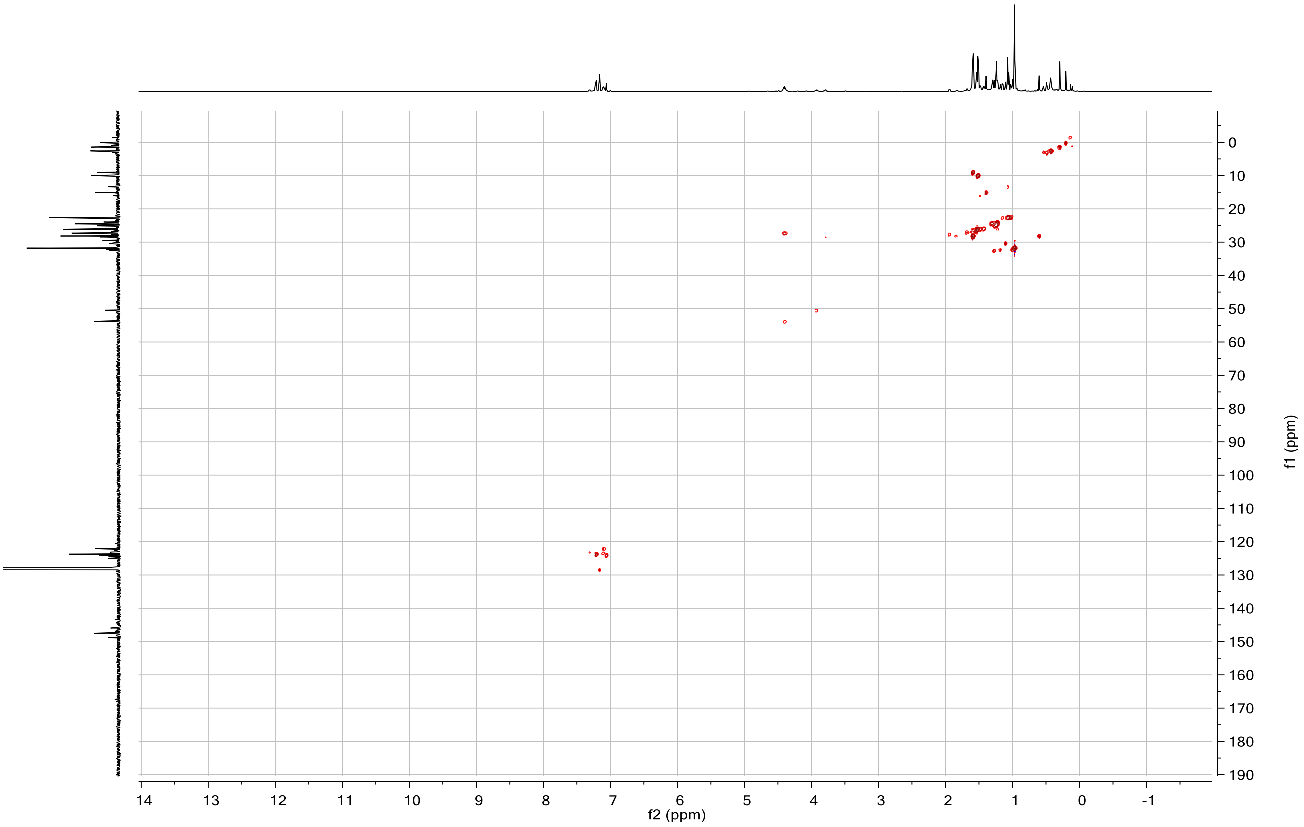

Figure S30. ^{1}H-^{13}C HMBC spectrum of **9**.

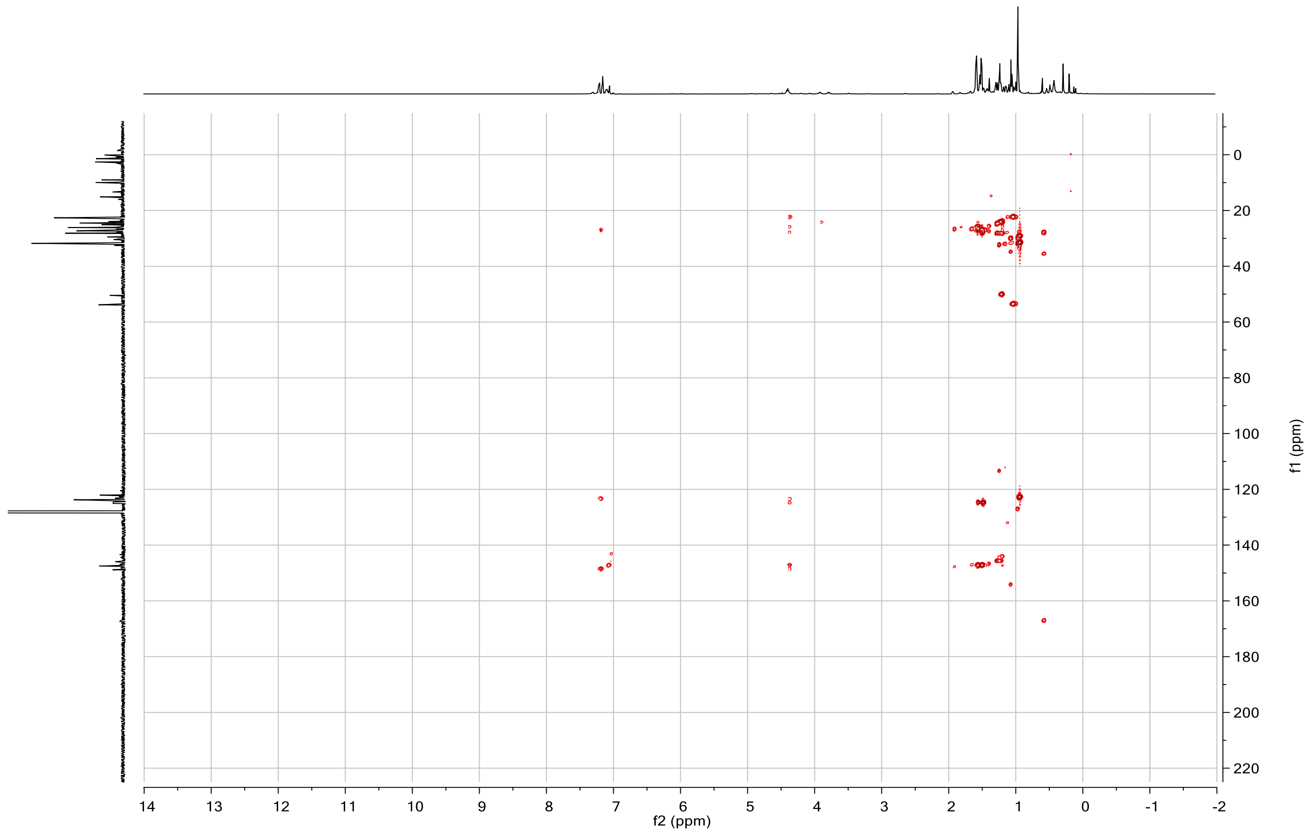

Heating reactions of [(NHC^{iPr})Cu-Al{SiN^{Dipp}}] with MesCCH; Isolation of **10** and **10a**

In a J Young's tube, 2-ethynyl-1,3,5-trimethylbenzene (7.2 mg, 7.8 mL, 0.05 mmol) was added via micropipette to a colourless solution of [(NHC^{iPr})Cu-Al{SiN^{Dipp}}] (**1**, 19 mg, 0.025 mmol) in C_6D_6. The tube was then immediately taken to a heating mantle and kept at 60°C to prevent side reaction previously observed at room temperature. Full consumption of A was observed by NMR spectroscopy after the reaction mixture was kept at 60°C for 3 days, along with the generation of related alkenes of 2-Ethynyl-1,3,5-trimethylbenzene. ^{1}H NMR spectrum of the crude reaction mixture indicates there are more than one Cu-Al involving species, despite bulk sample of each product was not obtained, two species were identified by mechanical separation of colourless single crystals and subsequent X-ray diffraction analysis. The products of the reaction, along with the mesityl-stryene, were found to be [(NHC^{iPr})Cu…(MesCC)$_2$-κ^2-C,C'-Al{SiN^{Dipp}}] (**10**) and [(NHC^{iPr})Cu(NHC^{iPr})][(MesCC)$_2$-κ^2-C,C'-Al{SiN^{Dipp}}] (**10a**), plausibly generated in the ratio of 4:1. (rough intepretation from the crude 1H NMR spectrum).

Figure S31. ^{1}H NMR (500 MHz, 298 K, d$_6$-benzene) spectrum of the reaction mixture *alkenes

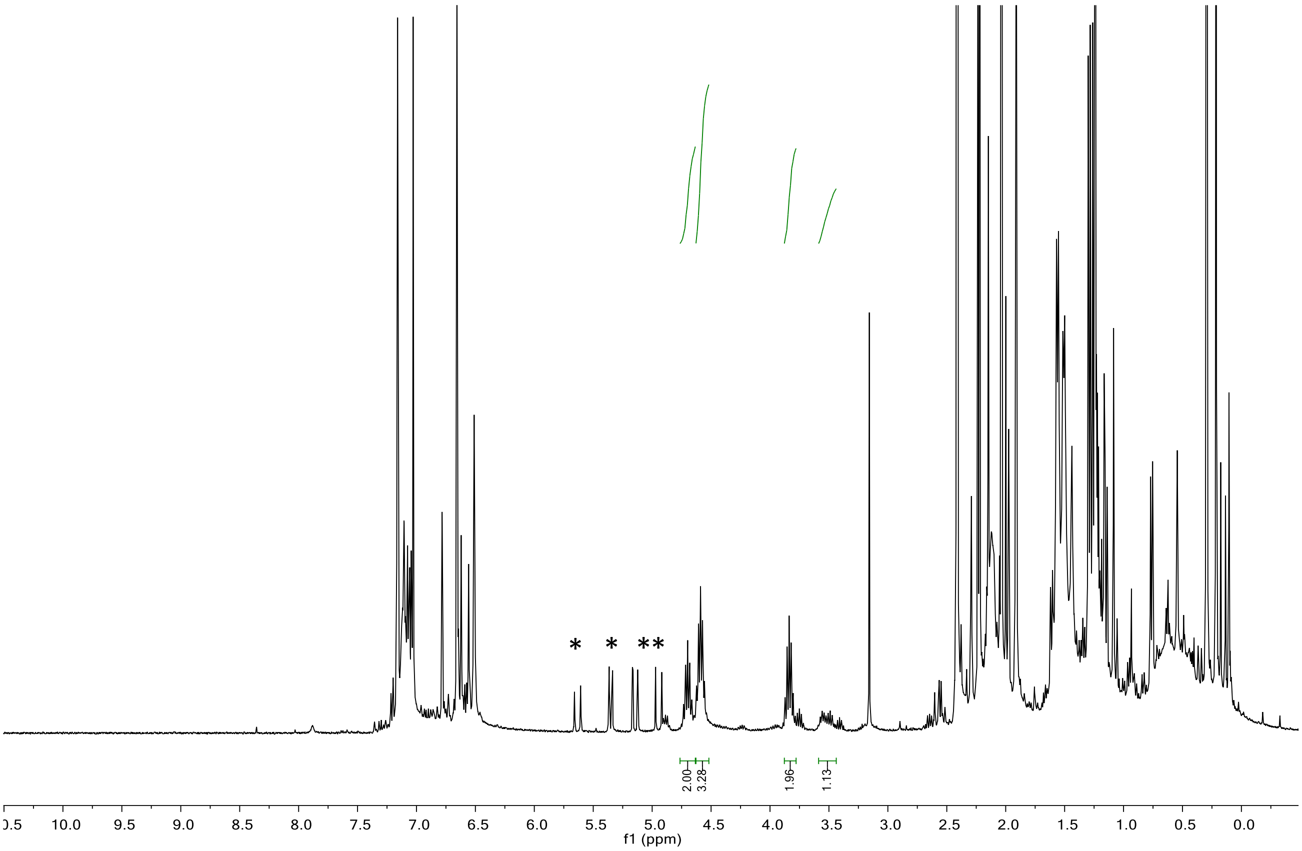

Room temperature reactions of [(NHC^{iPr})Cu-Al{SiN^{Dipp}}] with MesCCH; Isolation of **11**.

In a J Young's tube, 2-ethynyl-1,3,5-trimethylbenzene (7.2 mg, 7.8 mL, 0.05 mmol) was added via micropipette to a colourless solution of [(NHC^{iPr})Cu-Al{SiN^{Dipp}}] (**1**, 19 mg, 0.025 mmol) in C_6D_6. Upon addition of the acetylene, the reaction mixture transformed into a pale-yellow solution with significant amount of colourless crystalline solids precipitating within 15 mins, precluding in depth *in-situ* solution state characterisation of the reaction mixture. ^{1}H NMR spectrum shows two diagnostic doublets at 5.49 and 4.81 ppm (each integrated to 1H, J = 20.9Hz), alongside two septets at 4.56 and 4.46 ppm (each integrated to 2H), indicating some analogous reactivity to that between **1** with phenylacetylene or 1-hexyne taking place in the mixture. The identity of the colourless crystal was confirmed by X-ray diffraction analysis to be [(NHC^{iPr})Cu(NHC^{iPr})][(MesC^{H}C^H)(MesCC)-κ^2-C,C'-Al{SiN^{Dipp}}]. (**11**)

Figure S32. ^{1}H NMR (500 MHz, 298 K, d$_6$-benzene) spectrum of the reaction mixture

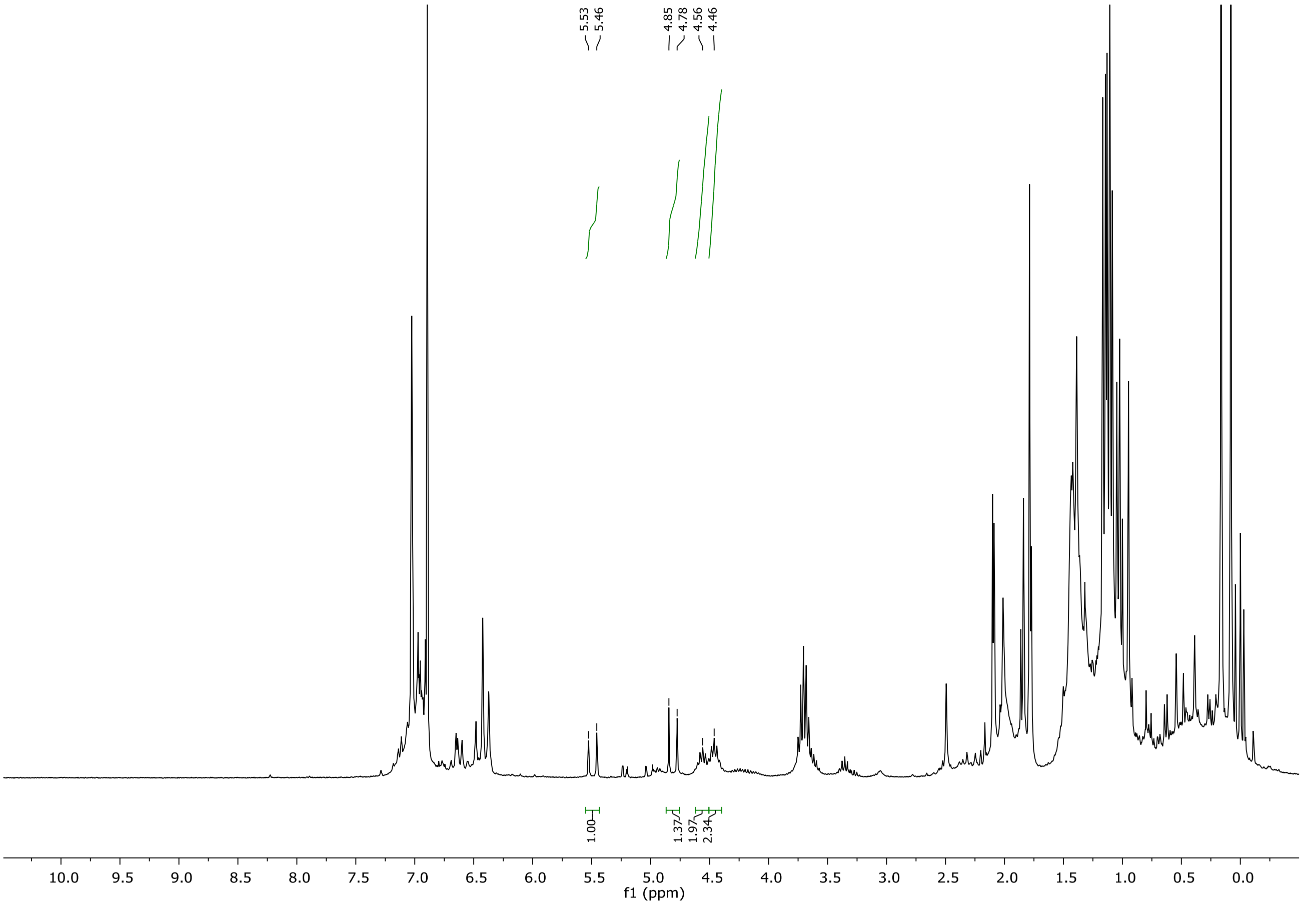

Synthesis of [(NHC^{iPr})Cu(H)(^{t}BuCC)Al{SiN^{Dipp}}] (**12**)

In a J Young's tube, 3,3-dimethyl-1-butyne (2.0 mg, 3.1 µl, 0.025 mmol) was added via micropipette to a colourless solution of [(NHC^{iPr})Cu-Al{SiN^{Dipp}}] (**1**, 19 mg, 0.025 mmol) in C_6D_6. Quantitative generation of **12** was observed by NMR spectroscopy in 2-3 hours at room temperature. The colourless solution was then put under vacuum to remove all volatiles and give compound **12** as a white crystalline solid. Yield 18.5 mg, 88 %. Single crystal suitable for X-ray crystallography was obtained by slow evaporation a concentrated benzene solution at room temperature. Anal Calc'd for $C_{54}H_{90}AlCuN_4Si_2$ (**12.**C_6H_6, 942.04): C, 68.85; H, 9.63; N, 5.95 %. Found: C, 68.90; H, 8.99; N, 5.45 %.^{1}H NMR (500 MHz, 298 K, Benzene-d_6) δ 7.14 (dd, J = 7.5, 1.8 Hz, 2H, m-C_6H_3), 7.09 (dd, J = 7.6, 1.8 Hz, 2H, m-C_6H_3), 7.00 (t, J = 7.5 Hz, 2H, p-C_6H_3), 4.29 (sept, J = 6.8 Hz, 2H, C*H*Me$_2$), 4.20 (sept, J = 6.8 Hz, 2H, C*H*Me$_2$), 4.07 (sept, J = 7.0 Hz, 2H, NC*H*Me$_2$), 1.59 (d, J = 6.8 Hz, 6H, CH*Me*$_2$), 1.52 (s, 6H, NC*Me*), 1.48 (d, J = 6.8 Hz, 6H, CH*Me*$_2$), 1.43 (d, J = 6.8 Hz, 6H, CH*Me*$_2$), 1.42 (d, J = 6.8 Hz, 6H, CH*Me*$_2$), 1.30-1.27 (br, 4H, SiC*H*$_2$), 1.02 (d, J = 7.0 Hz, 12H, NCH*Me*$_2$), 1.00 (s, 9H, C*Me*$_3$), 0.53-0.42 (s br, 6H, Si*Me*$_2$), 0.39 – 0.30 (s br, 6H, Si*Me*$_2$). ^{1}H resonance correlated to Al-*H* not observed. ^{13}C NMR (126 MHz, 298 K, Benzene-d_6) δ 181.5 (Cu$C_{carbene}$), 147.6 (i-C_6H$_3$), 147.4 (o-C_6H$_3$), 147.0 (o-C_6H$_3$), 144.4 (AlC*C*CMe$_3$), 125.0 (N*C*Me), 123.9 (m-C_6H$_3$), 123.3 (m-C_6H$_3$), 122.2 (p-C_6H$_3$), 53.7 (N*C*HMe$_2$), 50.4 (*C*Me$_3$), 32.1 (C*Me*$_3$), 29.9 (CH*Me*$_2$), 27.9 (CH*Me*$_2$), 26.0 (CH*Me*$_2$), 23.9 (CH*Me*$_2$), 22.6 (NCH*Me*$_2$), 15.2 (Si*C*H$_2$), 10.1 (NC*Me*), 0.9 (Si*Me*$_2$), -1.5 (Si*Me*$_2$). ^{13}C resonance correlated to Al-*C*C^{t}Bu not observed.

Figure S33. ^{1}H NMR (500 MHz, 298 K, d_6-benzene) spectrum of **12**. *grease

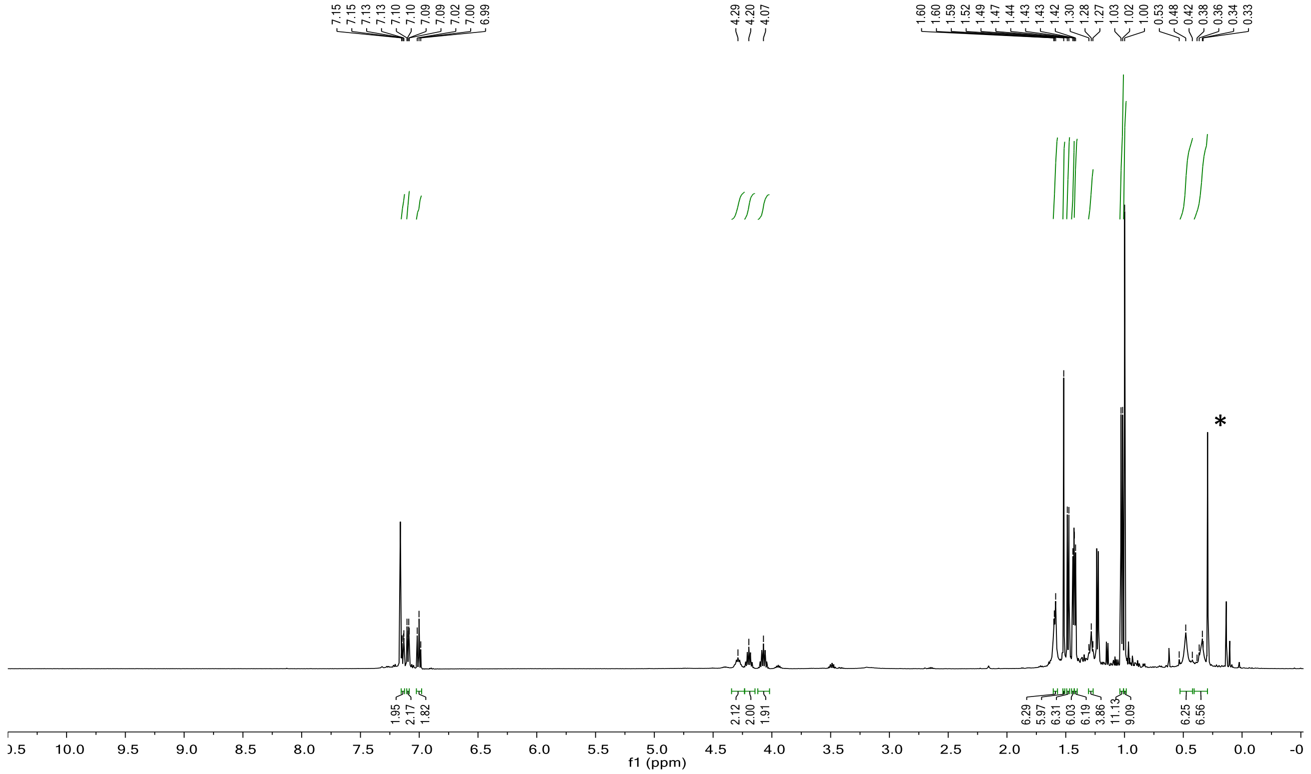

Figure S34. ^{13}C NMR (126 MHz, 298 K, d_6-benzene) spectrum of **12**. *grease

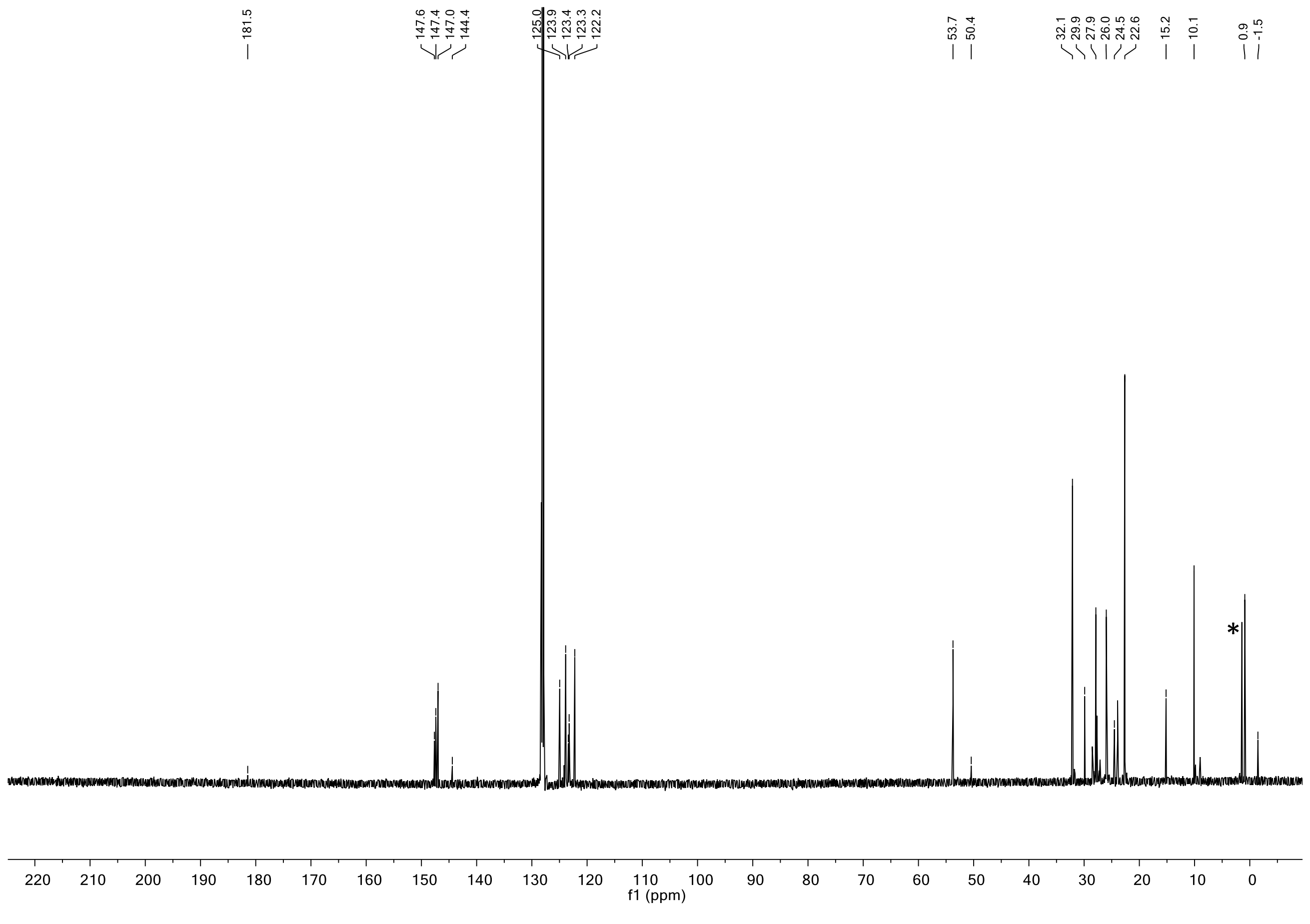

Figure S35. ^{1}H-^{1}H COSY spectrum of **12**.

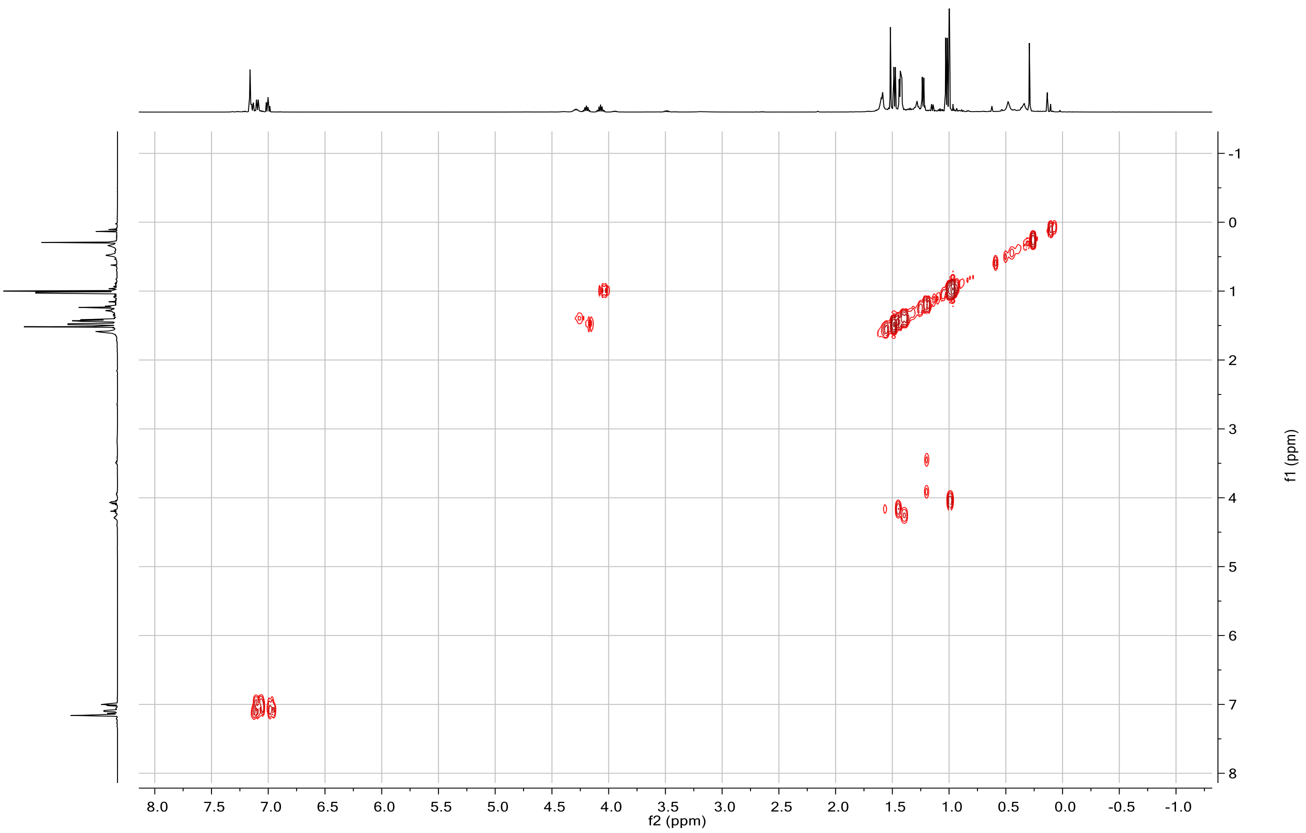

Figure S36. ^{1}H-^{13}C HSQC spectrum of **12**.

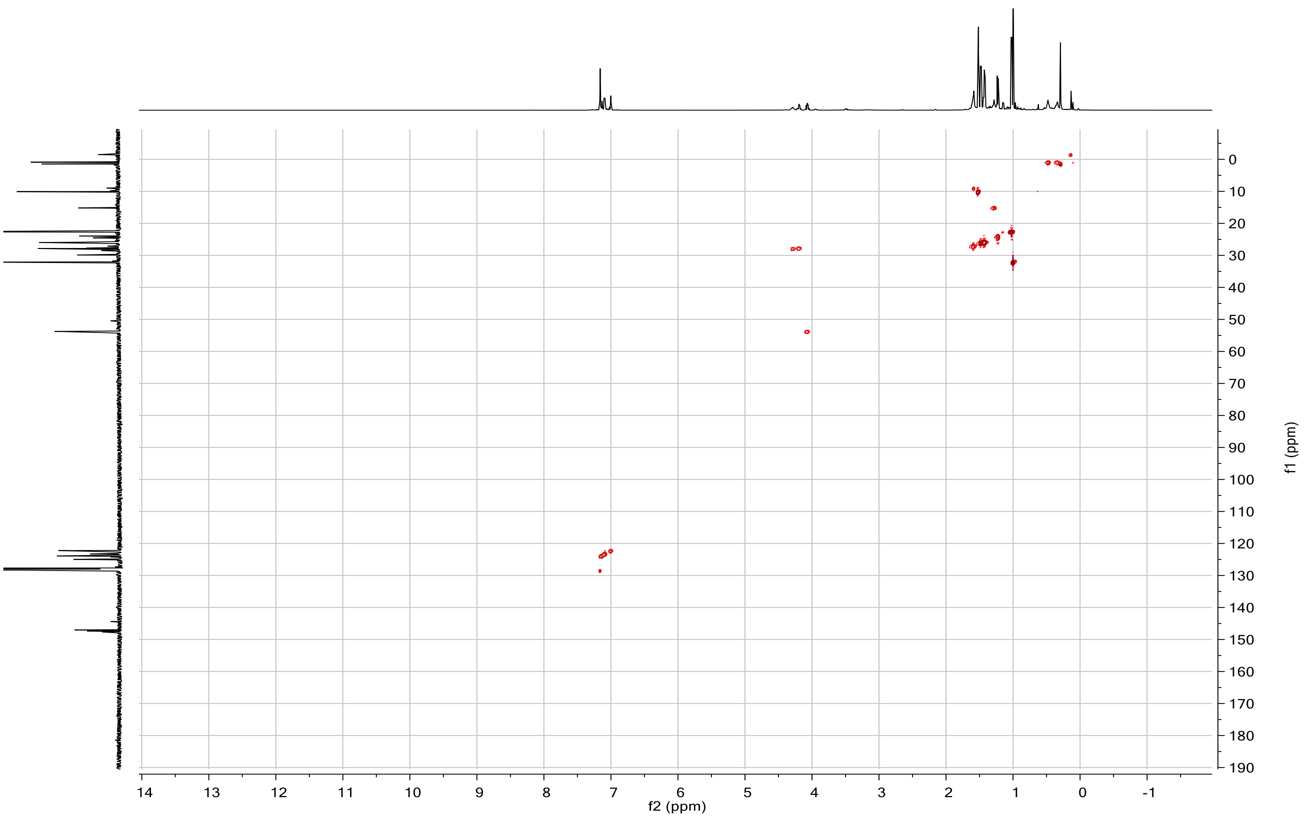

Figure S37. ^{1}H-^{13}C HMBC spectrum of **12**.

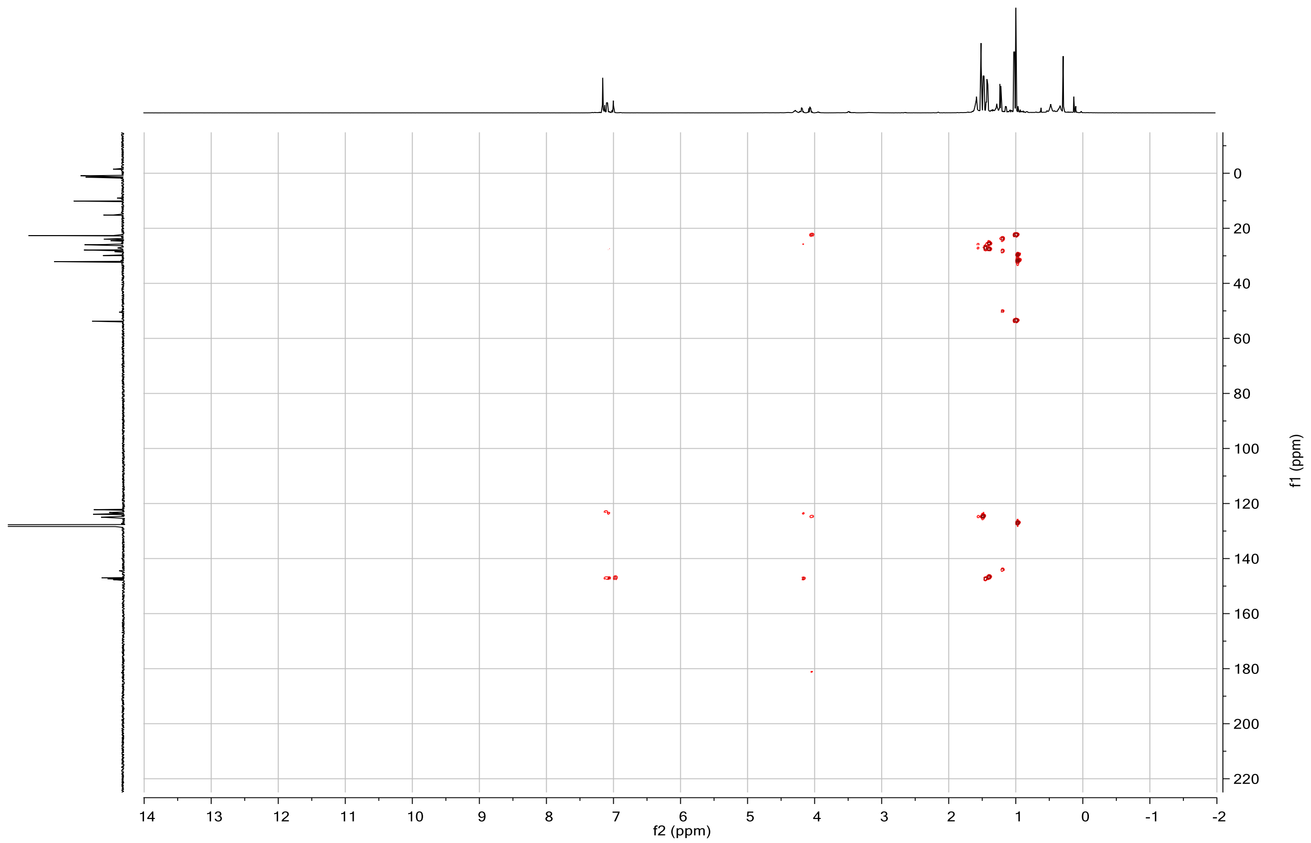

Synthesis of [(NHC^{iPr})Cu(H)(Me_3SiCC)Al{SiN^{Dipp}}] (**13**)

In a J Young's tube, ethynyltrimethylsilane (2.4 mg, 3.1 µl, 0.025 mmol) was added via micropipette to a colourless solution of [(NHC^{iPr})Cu-Al{SiN^{Dipp}}] (**1**, 19 mg, 0.025 mmol) in C_6D_6. Quantitative generation of **13** was observed by NMR spectroscopy overnight at room temperature. The colourless solution was then put under vacuum to remove all volatiles and give compound **13** as a white powder. Yield 19 mg, 89 %. Single crystal suitable for X-ray crystallography was obtained by slow evaporation a hexane solution at room temperature. Anal Calc'd for $C_{46}H_{80}AlCuN_4Si_3$ (**13**, 863.96) C, 63.95; H, 9.33; N, 6.49 %. Found: C, 64.38; H, 8.79; N, 6.28 %. ^{1}H NMR (500 MHz, 298 K, Benzene-*d*$_6$) δ 7.13 – 7.11 (m, 2H, *m*-C_6H_3), 7.09 – 7.06 (m, 2H, *m*-C_6H_3), 7.00 – 6.97 (m, 2H, *p*-C_6H_3), 4.29 (sept, J = 6.7 Hz, 2H, C*H*Me$_2$), 4.14 (sept, J = 6.7 Hz, 2H, C*H*Me$_2$), 3.94 (sept, J = 7.1 Hz, 2H, NC*H*Me$_2$), 1.50 (s, 6H, NC*Me*), 1.47 (d, J = 6.7 Hz, 12H, CH*Me*$_2$), 1.41 (d, J = 6.7 Hz, 12H, CH*Me*$_2$), 1.27 (s br, 4H, SiC*H*$_2$), 1.01 (d, J = 7.1 Hz, 12H, NCH*Me*$_2$), 0.47 (s br, 6H, Si*Me*$_2$), 0.34 (s br, 6H, Si*Me*$_2$), 0.04 (s, 9H, Si*Me*$_3$). ^{1}H resonance correlated to Al-*H* not observed. ^{13}C{^{1}H} NMR (126 MHz, 298 K, Benzene-*d*$_6$) δ 180.9 (Cu*C*$_{carbene}$), 147.7 (*i-C*$_6$H$_3$), 147.3 (Al*C*CSiMe$_3$), 146.9 (*o-C*$_6$H$_3$), 146.8 (*o-C*$_6$H$_3$), 124.8 (N*C*Me), 123.8 (*m-C*$_6$H$_3$), 123.26 (*m-C*$_6$H$_3$), 122.3 (*p-C*$_6$H$_3$), 118.1 (AlC*C*SiMe$_3$), 53.6 (N*C*HMe$_2$), 27.8 (*C*HMe$_2$), 27.6 (*C*HMe$_2$), 26.0 (CH*Me*$_2$), 25.6 (CH*Me*$_2$), 24.5 (CH*Me*$_2$), 22.6 (CH*Me*$_2$), 15.1(Si*C*H$_2$), 10.0 (NC*Me*), 1.2 (Si*Me*$_2$), 0.9 (Si*Me*$_3$), 0.8 (Si*Me*$_2$).

Figure S38. ^{1}H NMR (500 MHz, 298 K, d_6-benzene) spectrum of **13**. *grease

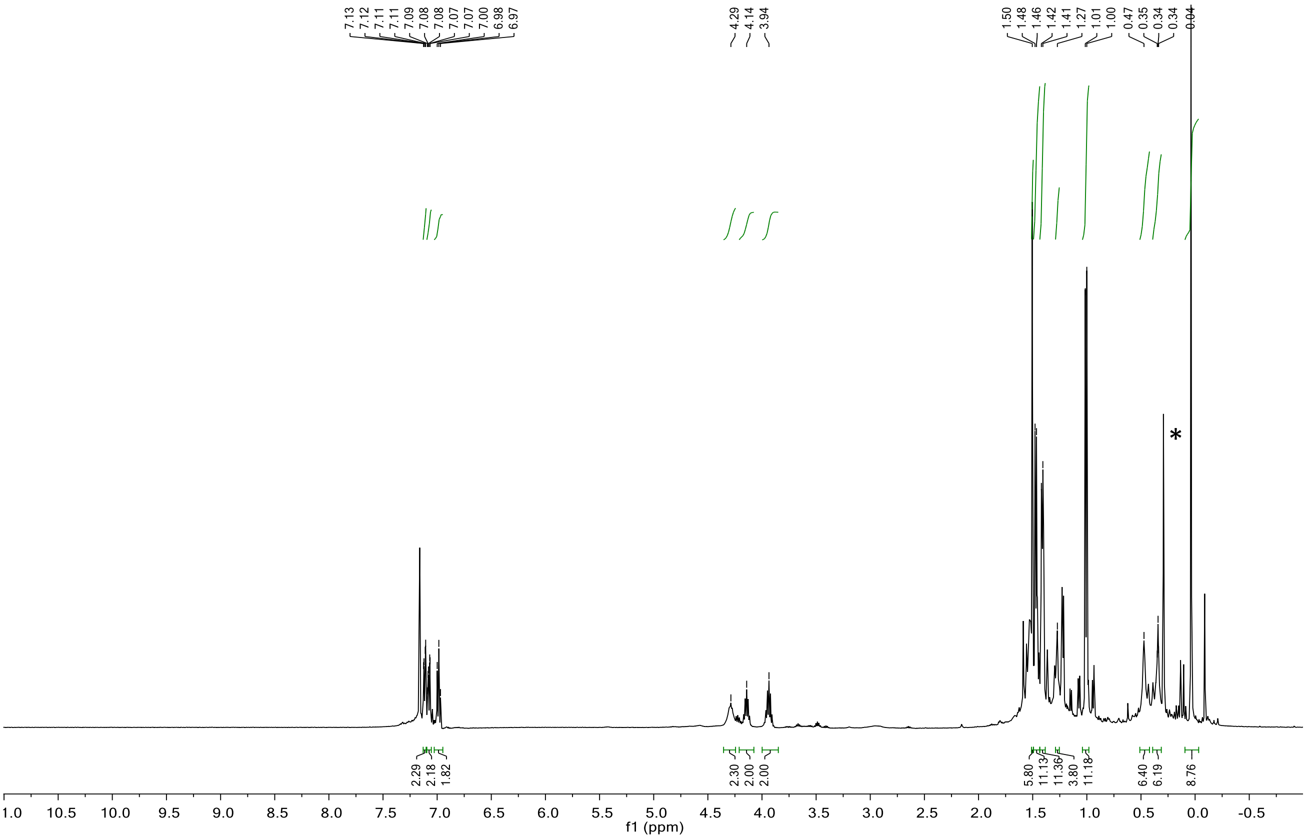

Figure S39. ^{13}C NMR (126 MHz, 298 K, d_6-benzene) spectrum of **13**. *grease

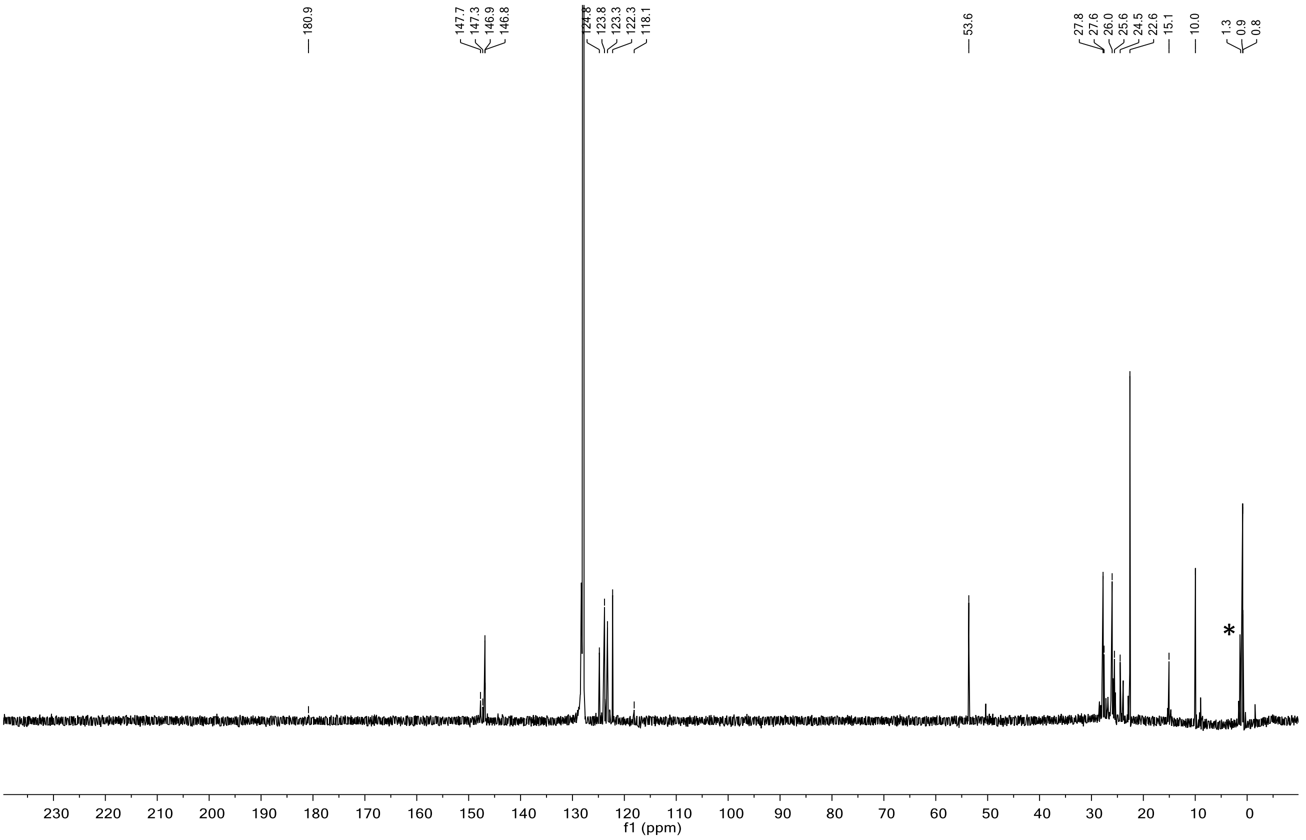

Figure S40. ^{1}H-^{1}H COSY spectrum of **13**.

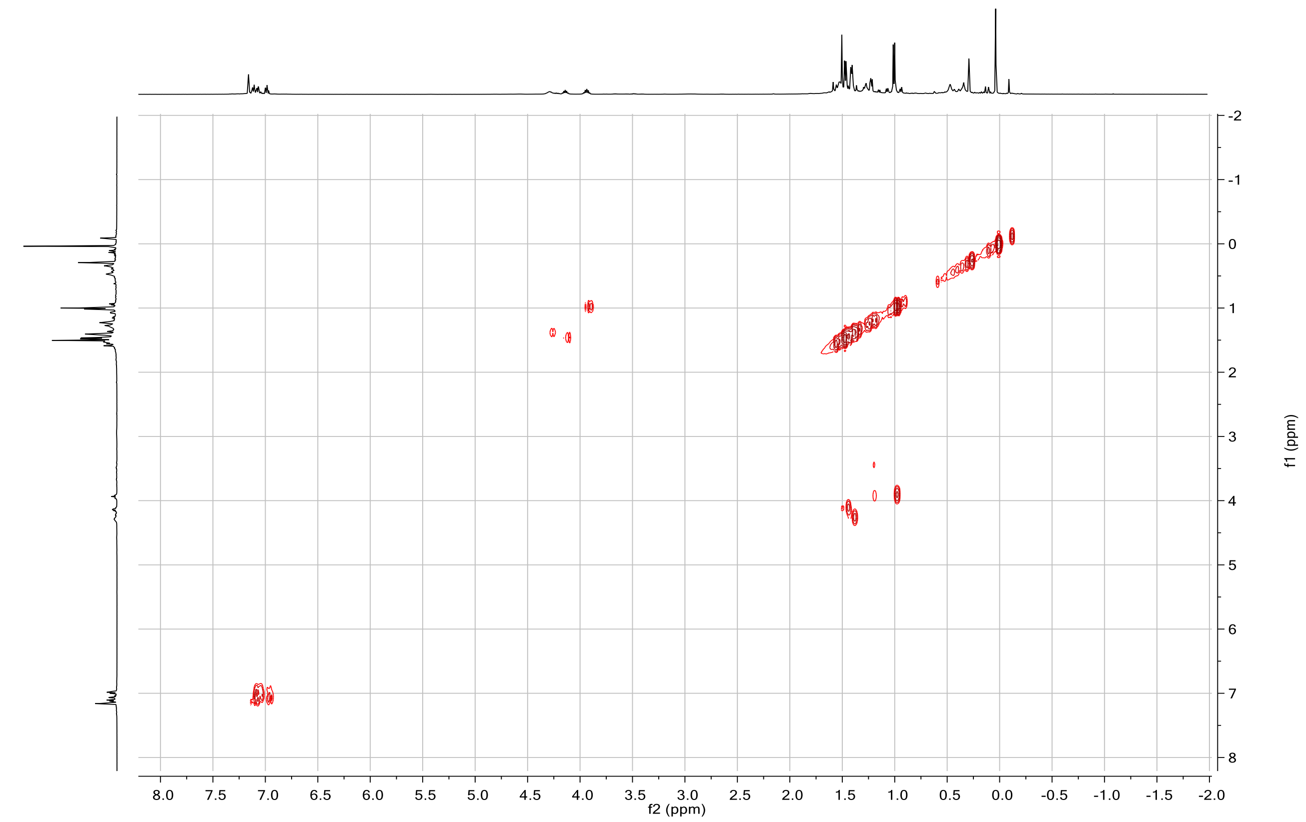

Figure S41. ^{1}H-^{13}C HSQC spectrum of **13**.

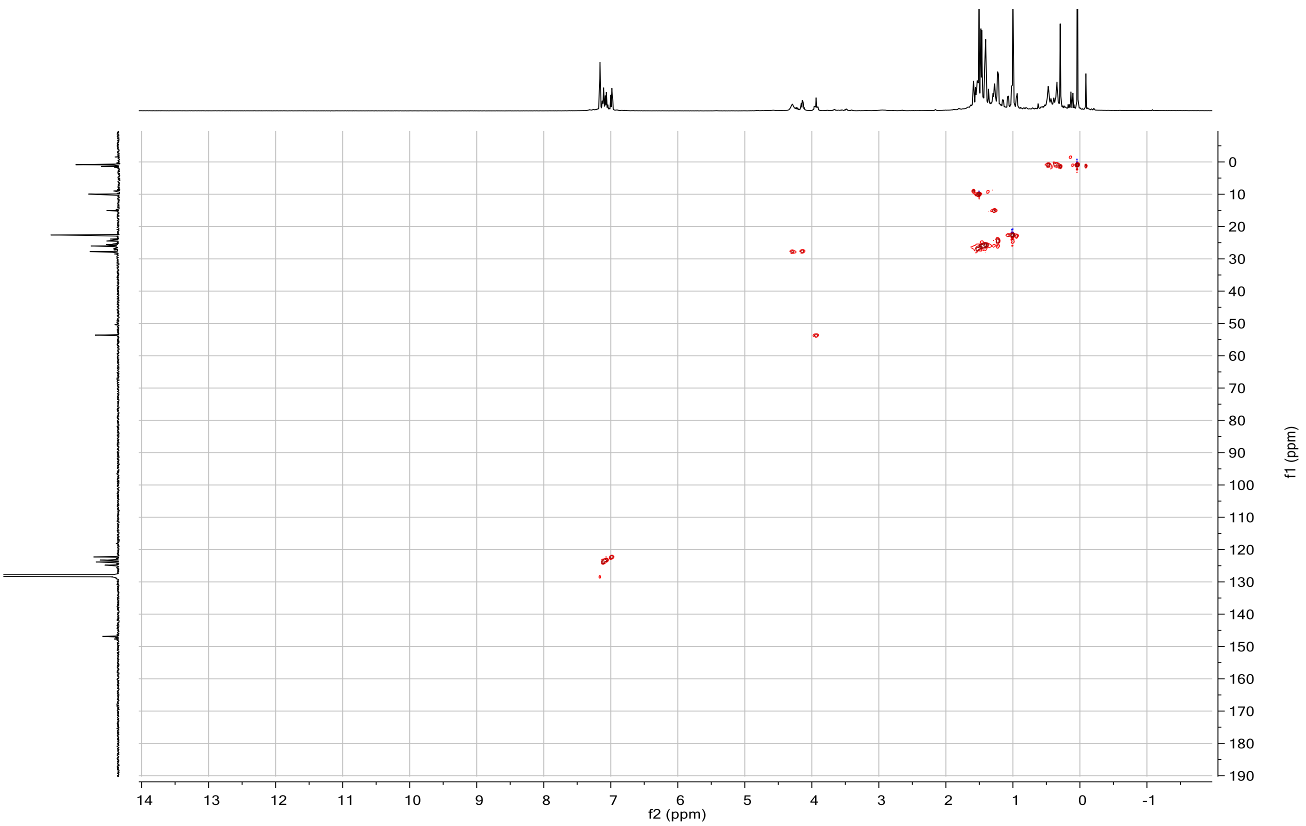

Figure S42. ^{1}H-^{13}C HMBC spectrum of **13**.

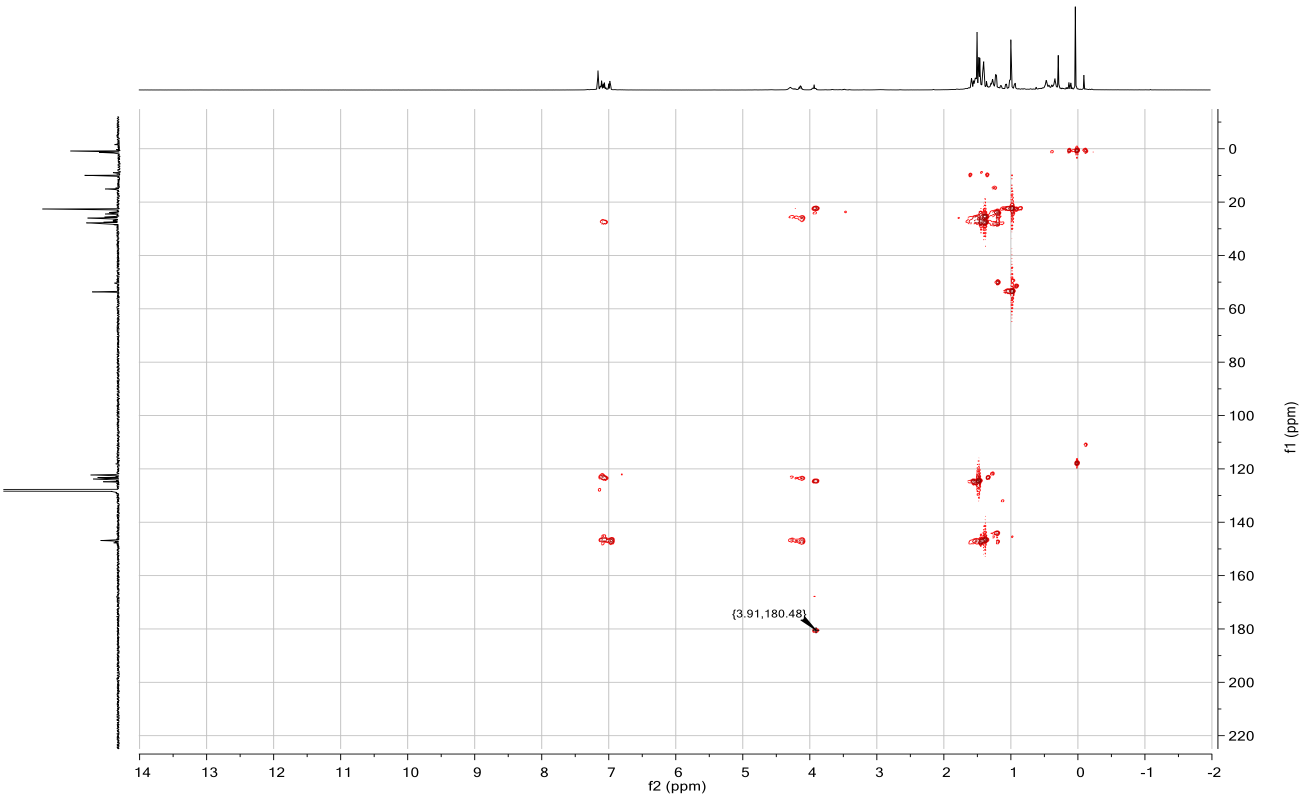

1.3. Generation of Alkenes from Corresponding Alkyne

Figure S43: ^{1}H NMR (500 MHz, d_6-benzene) spectrum of **1** with 3 equivalents of PhCCH (kept at 60°C overnight)

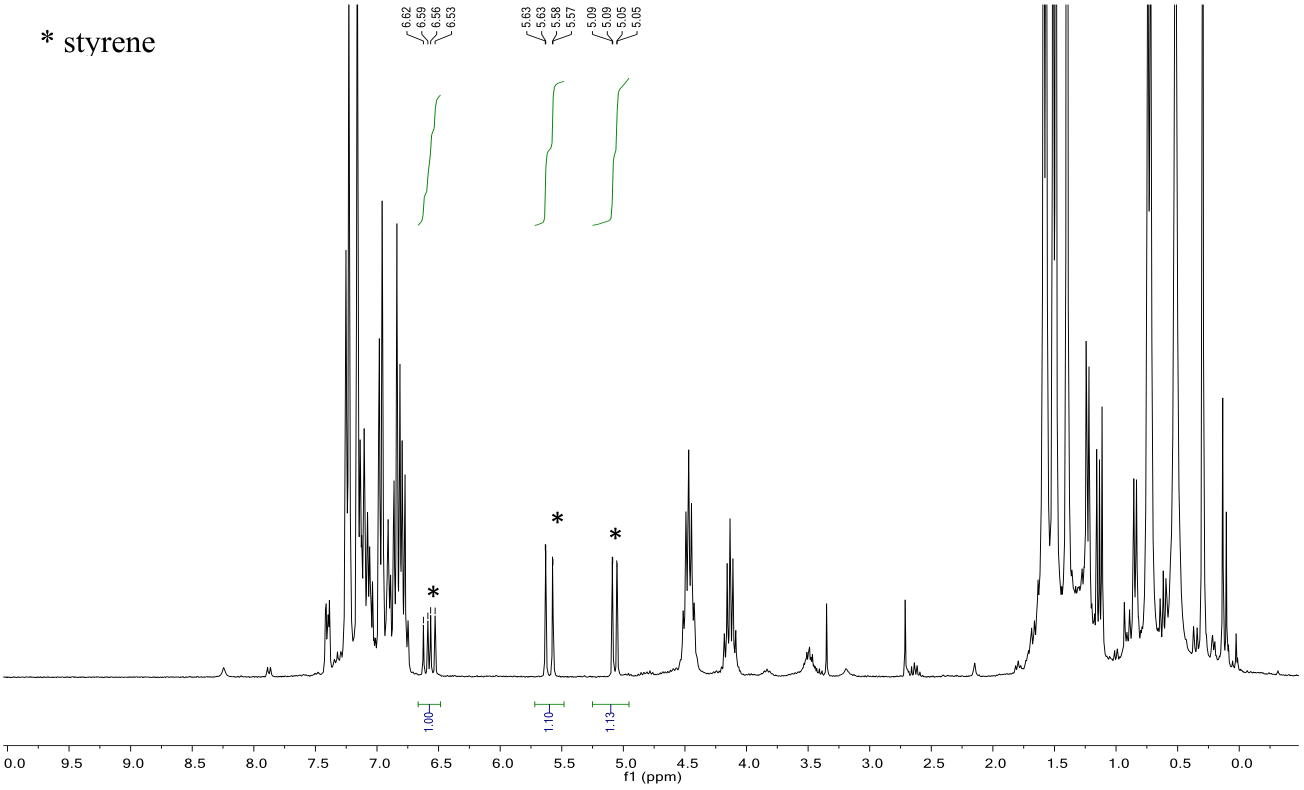

Figure S44: ^{1}H NMR (500 MHz, d$_6$-benzene) spectrum of **1** with 3 equivalents of ^{n}BuCCH (kept at 60°C overnight)

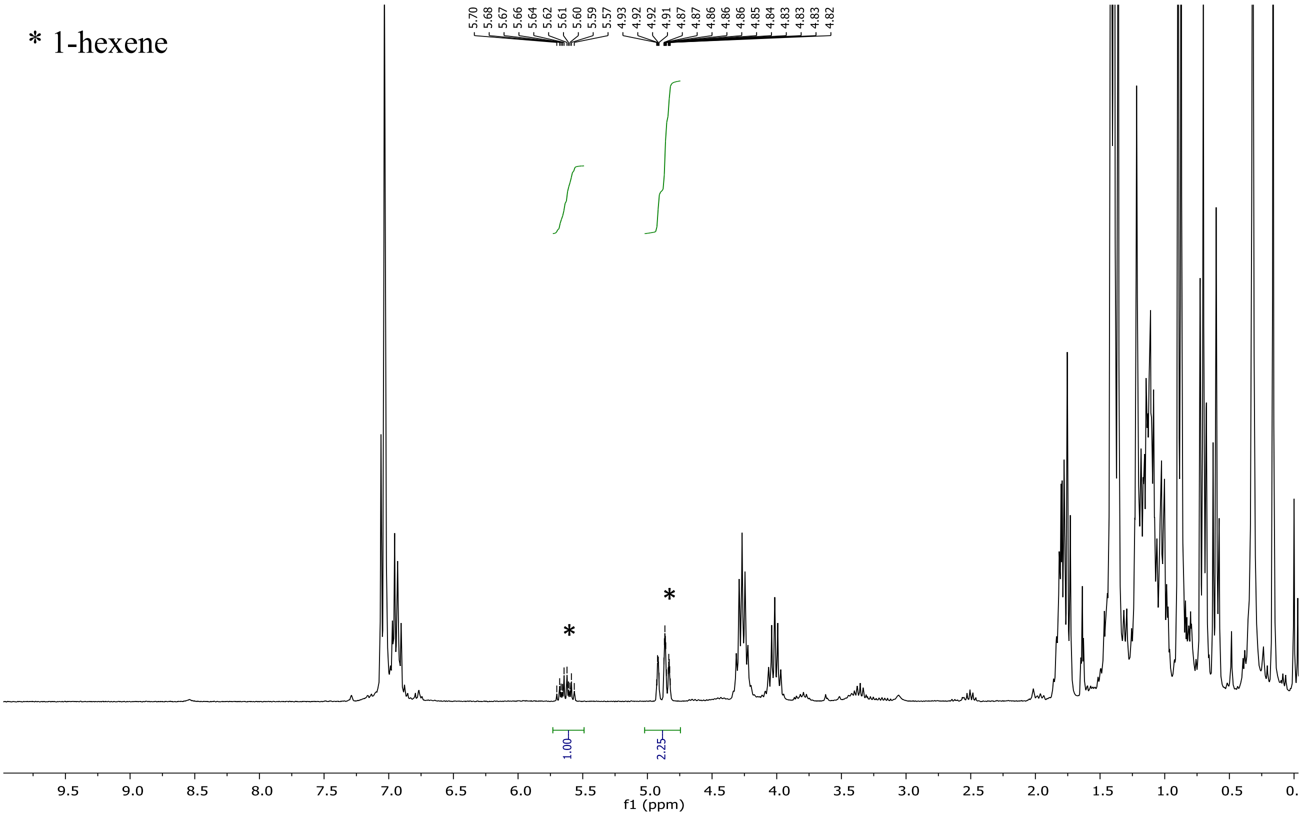

Figure S45: ^{1}H NMR (500 MHz, d$_6$-benzene) spectrum of **1** with 3 equivalents of MesCCH (kept at 60°C 3 days).

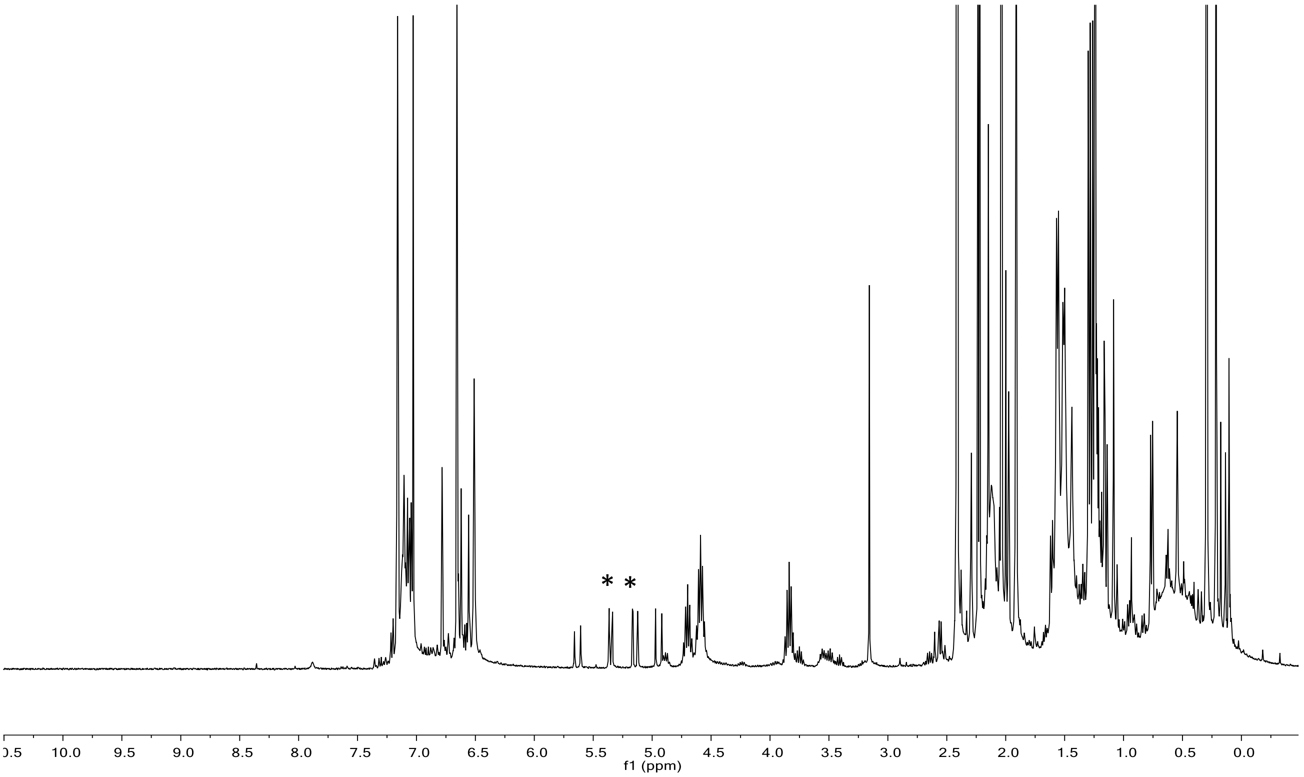

Figure S46: ^{1}H NMR (500 MHz, d$_6$-benzene) spectrum of **1** with 3 equivalents of ^{t}BuCCH (kept at 60°C overnight).

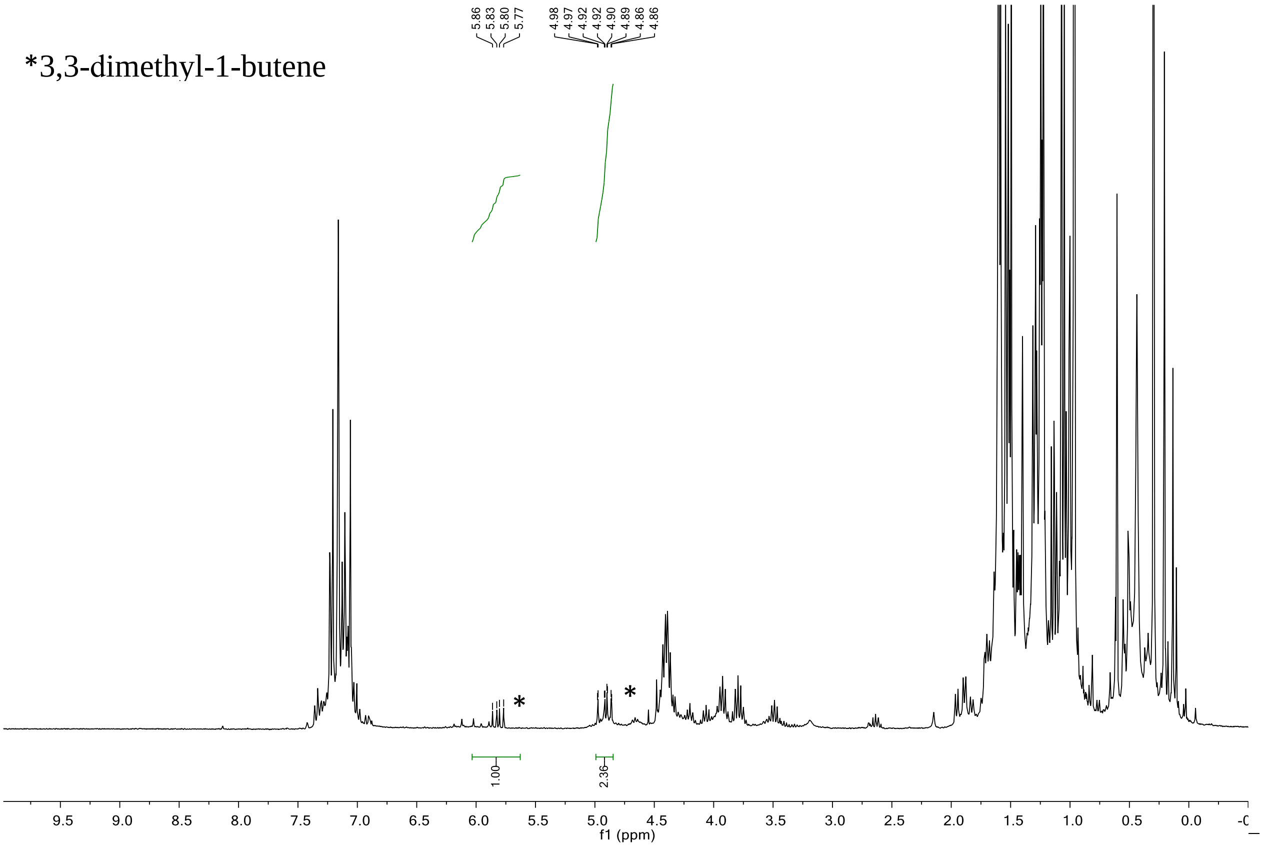

Figure S47: ^{1}H NMR (500 MHz, d_6-benzene) spectrum of **1** with 3 equivalents of Me_3SiCCH (kept at 60°C 3 days).

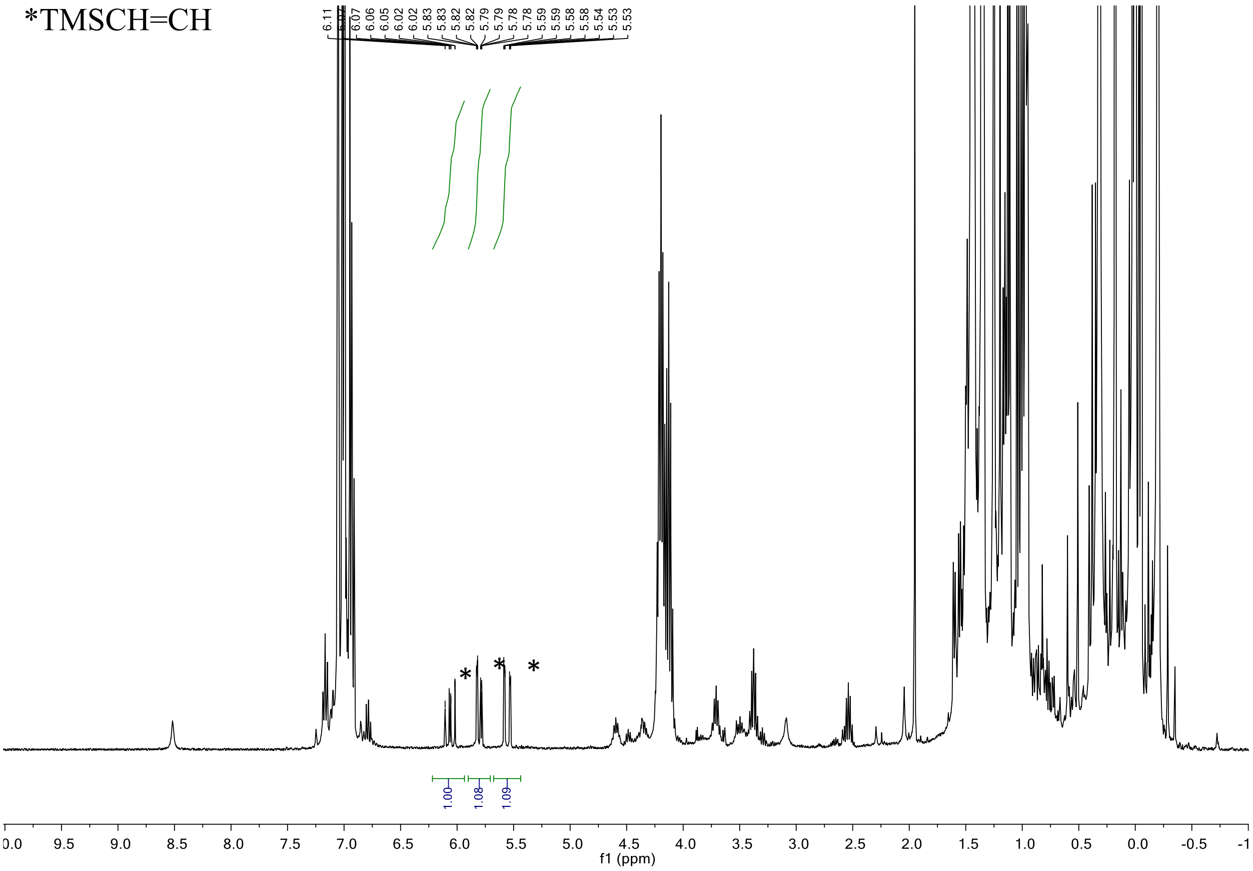

1.4. Single Crystal X-ray Diffraction Analysis

Single Crystal X–ray diffraction data were collected either on a SuperNova EosS2 diffractometer using CuKα (λ = 1.54184 Å) radiation (compounds **2a**, **3**, **5**, **7**, **10**, **10a**, **11**, **12**) or on a Rigaku Xcalibur diffractometer using Mo Kα (λ = 0.71073 Å) radiation (compounds **6**, **8** and **13**). The crystals were maintained at 150 K during data collection. Using Olex2,[2] the structures were solved with the olex2.solve[3] structure solution program or ShelXT and refined with the ShelXL[4] refinement package using Least Squares minimisation. Noteworthy points follow, and where disorder has been modelled, both distance and ADP restraints have been employed, on merit, in these regions to assist convergence. The hydrogen atoms attached to C31, C44 and C45 in the structure of **2a** were each located, and refined subject to being a distance of 0.98 Å from the relevant parent atom.

The asymmetric unit in **5** comprises one aluminium based complex and two crystallographically independent copper containing species. The metal centres in the latter are co-incident with inversion centres intrinsic to the space group and these symmetry elements serve to generate the remainder of transition metal complexes.

In the structure of **6**, the asymmetric unit contains two crystallographically (and chemically) distinct molecules, plus two regions of solvent. The mixed-metal complexes differ, most prominently, in the comparative Cu2 and Cu4 environments. Both solvent moieties were disordered in a manner that was accessible to modelling (disorder ratios of 55:45 and 75:25, for the moieties based on C1S and C1TS, respectively). There was evidence for some smearing of the electron density in the phenyl group based on C53 which was not modelled but this ring was treated as a rigid hexagon in the final least-squares cycles.

Structural refinement of **7** was unremarkable, with the exception of disorder modelling for C46-C49 for which each carbon was treated as being split over two sites in an 85:25 ratio. While there was some comparative smearing of electron density evident for C50-C53, this was not positionally divergent enough to lend itself to ready modelling.

In **8**, the $SiMe_3$ group based on Si3 was treated for 50:50 disorder over 2 proximate sites.

In the structure of **10**, the asymmetric unit comprises one molecule of benzene and half of the metal complex. Cu1, Al1 and C1, in the latter, are all located on a crystallographic 2-fold rotation axis, which serves to generate the remainder of the molecule.

The bridging hydride was located, and refined freely with a riding U_{iso} value, in the structure of **12**. The hydride was also located and refined in the structure of **13**, with a fixed U_{iso} value in this instance.

Figure S48: X-ray structure of **5**: Symm ops: ^{1} $2-x, -y, 2-z$; ^{2} $-x$ $2-y, 1-z$

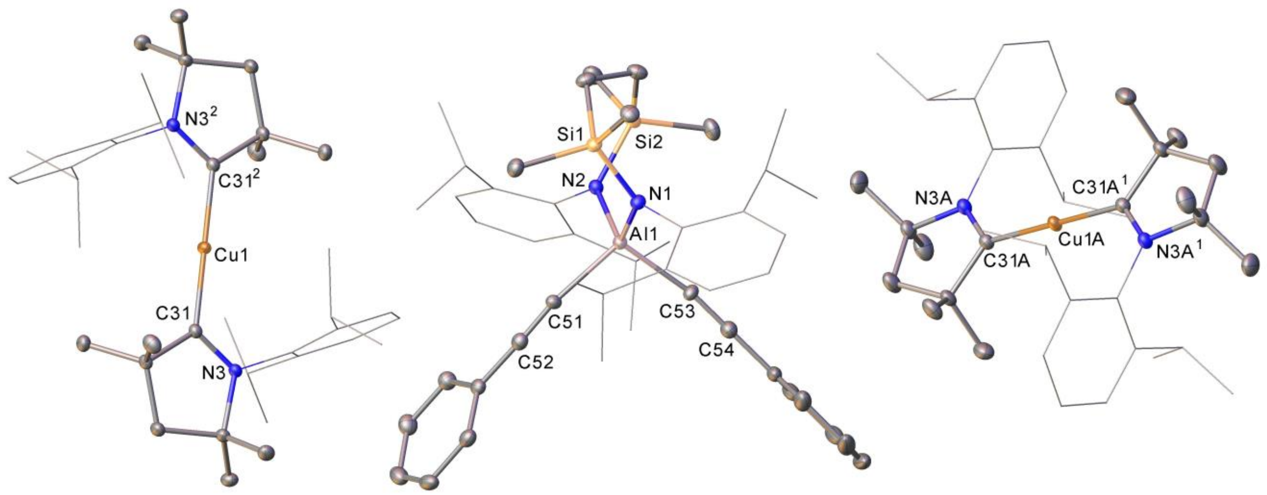

Figure S49: X-ray structure of **6**.

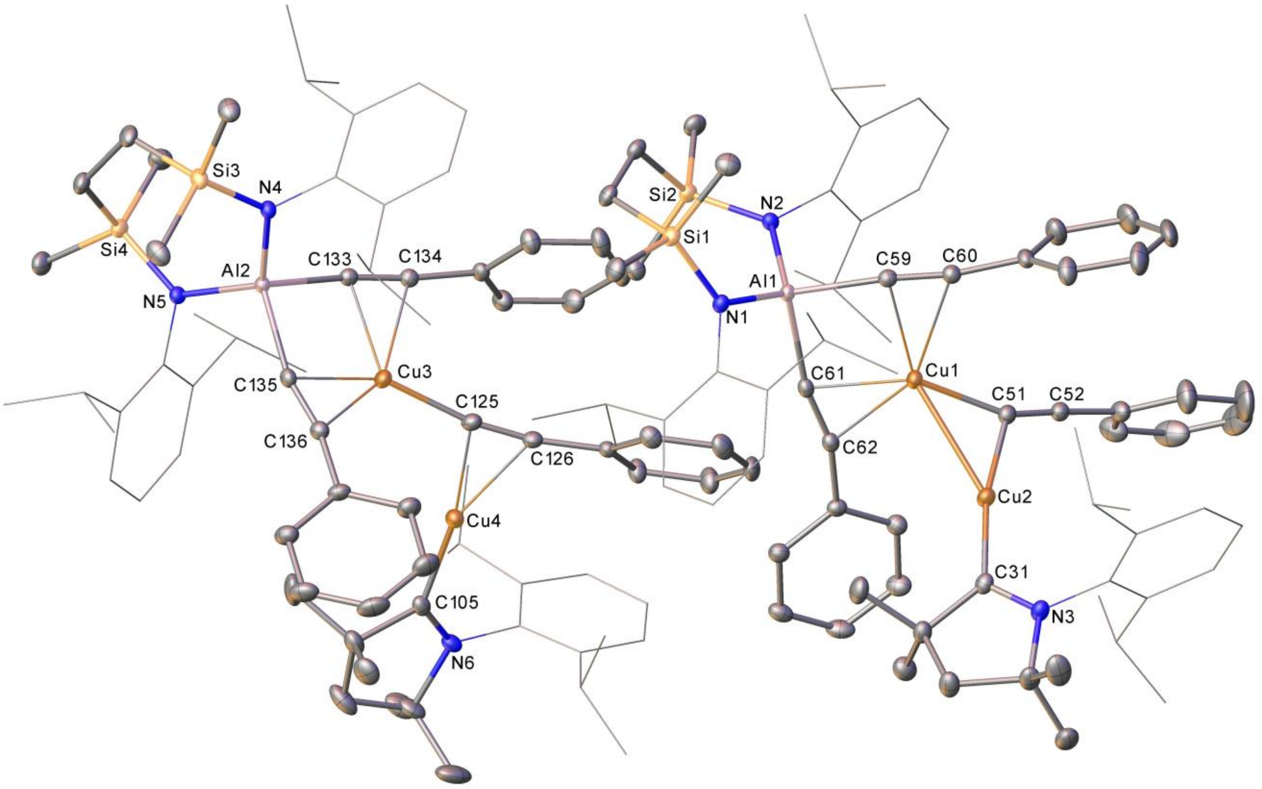

Figure S50: X-ray structure of **10a**.

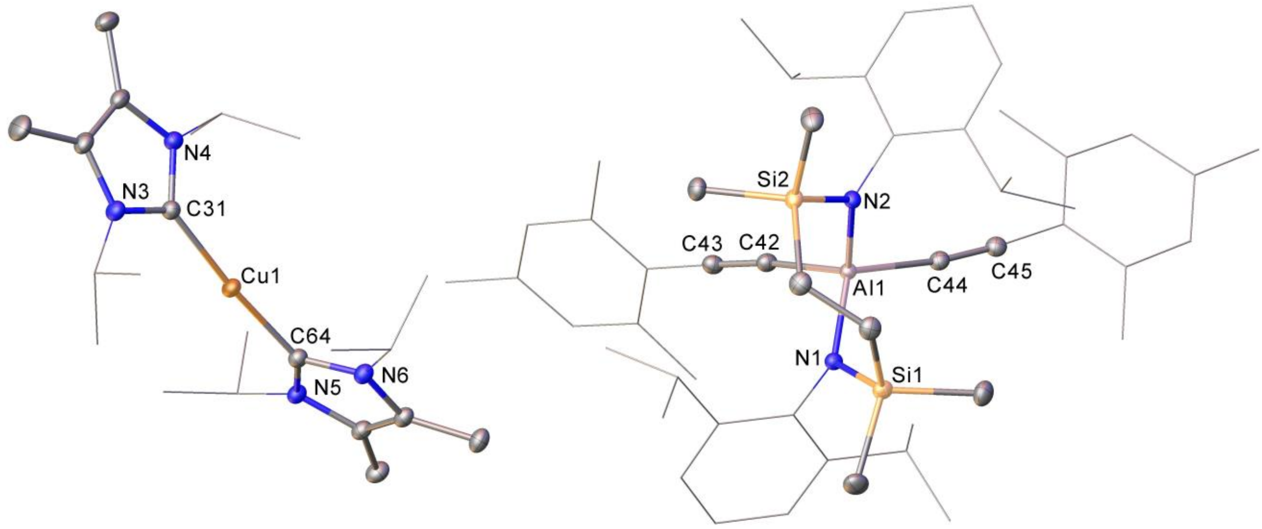

Figure S51: X-ray structure of **11**.

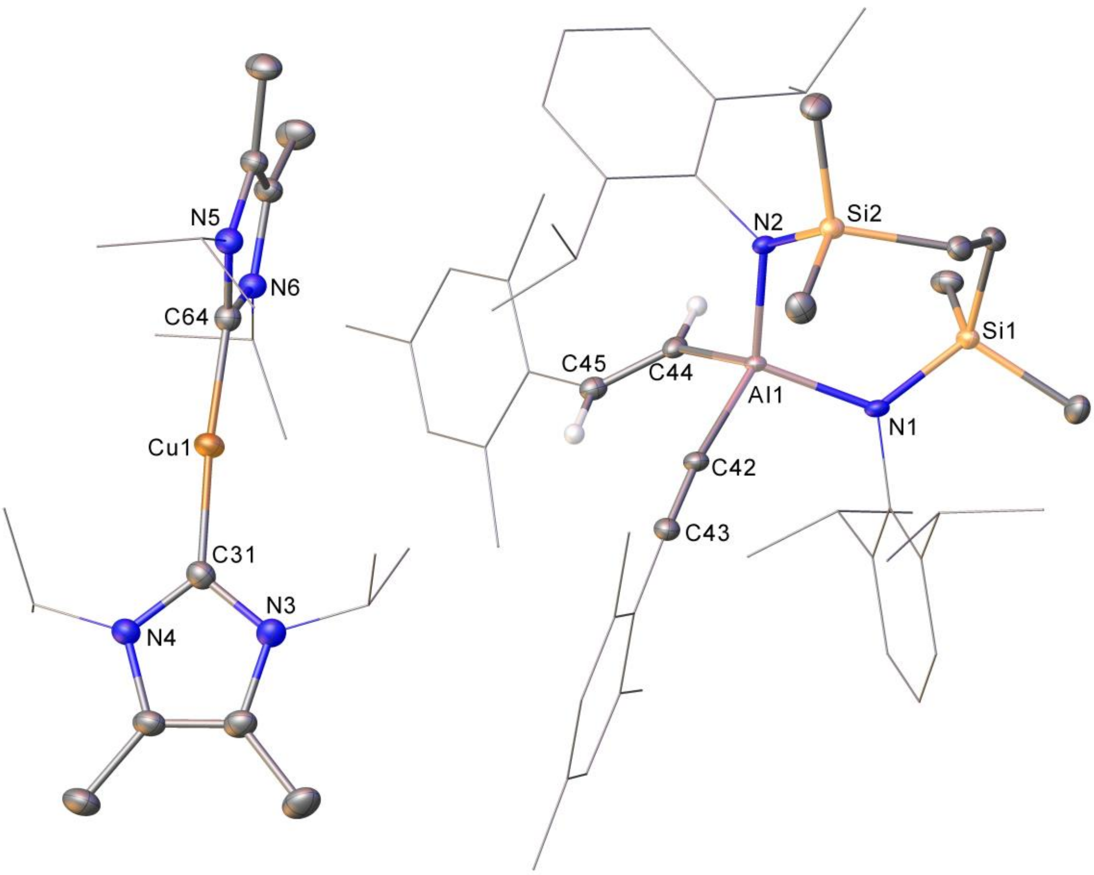

Table S1: Crystal data and structure refinement for compounds **2a**, **3, 5** and **6.**

Compound	**2a**	**3**	**5**	**6**
Empirical formula	$C_{57}H_{83}AlN_4Si_2$	$C_{57}H_{80}AlCuN_4Si_2$	$C_{86}H_{122}AlCuN_4Si_2$	$C_{160}H_{220}Al_2Cu_4N_6Si_4$
Formula weight	907.43	967.95	1358.57	2647.89
Crystal system	monoclinic	monoclinic	triclinic	triclinic
Space group	$P2_1/c$	*Cc*	*P*-1	*P*-1
a/Å	16.00380(10)	20.8885(2)	12.2250(2)	13.2732(2)
b/Å	13.50320(10)	13.8728(1)	12.4368(2)	17.1411(3)
c/Å	25.41530(10)	19.6118(2)	27.9562(4)	34.2337(6)
α/°	90	90	81.885(1)	97.978(1)
β/°	97.5190(10)	101.347(1)	86.337(1)	98.341(1)
γ/°	90	90	74.292(2)	96.908(2)
Volume/Å^3	5445.08(6)	5572.06(9)	4049.28(12)	7553.0(2)
Z	4	4	2	2
ρ_{calc} g/cm^3	1.107	1.154	1.114	1.164
μ/mm^{-1}	1.031	1.400	1.093	0.649
F(000)	1976.0	2080.0	1472.0	2840.0
Crystal size/mm^3	0.148 × 0.115 × 0.075	0.166 × 0.097 × 0.086	0.168 × 0.129 × 0.054	0.604 × 0.472 × 0.279
2θ range /°	7.428 to 145.89	7.698 to 145.962	7.446 to 145.826	6 to 58.258
Index ranges	-19 ≤ h ≤ 19, -16 ≤ k ≤ 16, -31 ≤ l ≤ 28	-25 ≤ h ≤ 22, -17 ≤ k ≤ 17, -24 ≤ l ≤ 24	-14 ≤ h ≤ 15, -15 ≤ k ≤ 15, -34 ≤ l ≤ 29	-18 ≤ h ≤ 17, -23 ≤ k ≤ 23, -46 ≤ l ≤ 46
Reflections collected	75392	29874	56874	128297
Independent reflections	10831 [R_{int} = 0.0409, R_{sigma} = 0.0246]	8301 [R_{int} = 0.0423, R_{sigma} = 0.0513]	16041 [R_{int} = 0.0327, R_{sigma} = 0.0372]	38945 [R_{int} = 0.0311, R_{sigma} = 0.0408]
Data/restraints/parameters	10831/3/607	8301/2/605	16041/0/878	38945/291/1741
Goodness-of-fit on F^2	1.012	1.031	1.025	1.050
Final R indexes [I>=2σ (I)]	R_1 = 0.0395, wR_2 = 0.1056	R_1 = 0.0371, wR_2 = 0.0903	R_1 = 0.0358, wR_2 = 0.0904	R_1 = 0.0425, wR_2 = 0.0943
Final R indexes [all data]	R_1 = 0.0452, wR_2 = 0.1104	R_1 = 0.0390, wR_2 = 0.0921	R_1 = 0.0413, wR_2 = 0.0947	R_1 = 0.0612, wR_2 = 0.1016
Largest diff. peak/hole / e Å^{-3}	0.56/-0.24	0.34/-0.36	0.53/-0.36	0.61/-0.36
Flack parameter	-	0.17(3)	-	-

Table S1: Crystal data and structure refinement for compounds **7**, **8**, **10** and **10a**.

Compound	**7**	**8**	**10**	**10a**
Empirical formula	$C_{53}H_{88}AlCuN_4Si_2$	$C_{51}H_{88}AlCuN_4Si_4$	$C_{75}H_{104}AlCuN_4Si_2$	$C_{74}H_{112}AlCuN_6Si_2$
Formula weight	927.97	960.13	1208.32	1232.39
Crystal system	monoclinic	monoclinic	orthorhombic	monoclinic
Space group	*Cc*	$P2_1/n$	*Pbcn*	$P2_1/c$
a/Å	21.4197(1)	12.6138(3)	15.0236(1)	12.40965(5)
b/Å	13.5245(1)	20.7585(4)	21.9618(1)	23.21981(10)
c/Å	19.6839(1)	22.1325(4)	21.1294(2)	25.09012(11)
α/°	90	90	90	90
β/°	103.151(1)	93.689(2)	90	92.1704(4)
γ/°	90	90	90	90
Volume/Å^3	5552.69(6)	5783.2(2)	6971.55(9)	7224.52(5)
Z	4	4	4	4
ρ_{calc} g/cm^3	1.110	1.103	1.151	1.133
μ/mm^{-1}	1.379	0.510	1.213	1.185
F(000)	2016.0	2080.0	2608.0	2672.0
Crystal size/mm^3	0.198 × 0.131 × 0.066	0.882 × 0.689 × 0.557	0.164 × 0.085 × 0.066	0.216 × 0.153 × 0.055
2θ range /°	7.79 to 145.704	5.872 to 60.604	7.128 to 145.926	7.052 to 146.126
Index ranges	-26 ≤ h ≤ 26, -16 ≤ k ≤ 16, -24 ≤ l ≤ 21	-17 ≤ h ≤ 17, -29 ≤ k ≤ 29, -29 ≤ l ≤ 31	-18 ≤ h ≤ 17, -27 ≤ k ≤ 26, -25 ≤ l ≤ 26	-14 ≤ h ≤ 15, -28 ≤ k ≤ 28, -30 ≤ l ≤ 29
Reflections collected	49961	98784	80334	97505
Independent reflections	9866 [R_{int} = 0.0313, R_{sigma} = 0.0265]	15956 [R_{int} = 0.0315, R_{sigma} = 0.0263]	6963 [R_{int} = 0.0557, R_{sigma} = 0.0251]	14393 [R_{int} = 0.0309, R_{sigma} = 0.0196]
Data/restraints/parameters	9866/69/601	15956/100/613	6963/0/388	14393/0/787
Goodness-of-fit on F^2	1.023	1.039	1.024	1.031
Final R indexes [I>=2σ (I)]	R_1 = 0.0273, wR_2 = 0.0729	R_1 = 0.0348, wR_2 = 0.0844	R_1 = 0.0351, wR_2 = 0.0917	R_1 = 0.0310, wR_2 = 0.0829
Final R indexes [all data]	R_1 = 0.0278, wR_2 = 0.0734	R_1 = 0.0509, wR_2 = 0.0920	R_1 = 0.0424, wR_2 = 0.0965	R_1 = 0.0346, wR_2 = 0.0857
Largest diff. peak/hole / e Å^{-3}	0.41/-0.25	0.50/-0.37	0.27/-0.51	0.36/-0.40
Flack parameter	0.000(6)	-	-	-

Table S1: Crystal data and structure refinement for compounds **11**, **12** and **13**.

Compound	**11**	**12**	**13**
Empirical formula	$C_{74}H_{114}AlCuN_6Si_2$	$C_{46}H_{80}AlCuN_4Si_3$	$C_{188}H_{320}Al_4Cu_4N_{16}Si_8$
Formula weight	1234.41	863.93	3391.38
Crystal system	monoclinic	Monoclinic	monoclinic
Space group	$P2_1/n$	$P2_1/n$	$P2_1/c$
a/Å	13.5126(1)	15.0696(4)	16.2074(3)
b/Å	23.3252(1)	16.5798(5)	12.7848(2)
c/Å	23.6673(2)	20.4317(4)	24.4265(5)
α/°	90	90	90
β/°	105.075(1)	91.457(2)	100.576(2)
γ/°	90	90	90
Volume/Å^3	7202.84(9)	5103.2(2)	4975.39(16)
Z	4	4	1
ρ_{calc} g/cm^3	1.138	1.124	1.132
μ/mm^{-1}	1.188	1.684	0.538
F(000)	2680.0	1872.0	1840.0
Crystal size/mm^3	0.2 × 0.125 × 0.075	0.095 × 0.062 × 0.03	0.412 × 0.35 × 0.169
2θ range /°	7.58 to 145.928	7.93 to 146.056	6 to 60.752
Index ranges	$-16 \le h \le 16, -28 \le k \le 28, -28 \le l \le 29$	$-18 \le h \le 16, -17 \le k \le 20, -23 \le l \le 25$	$-22 \le h \le 21, -14 \le k \le 18, -32 \le l \le 32$
Reflections collected	104118	28989	52870
Independent reflections	14348 [R_{int} = 0.0564, R_{sigma} = 0.0341]	10112 [R_{int} = 0.0562, R_{sigma} = 0.0631]	13342 [R_{int} = 0.0315, R_{sigma} = 0.0342]
Data/restraints/parameters	14348/0/787	10112/0/524	13342/0/520
Goodness-of-fit on F^2	1.022	1.018	1.030
Final R indexes [I>=2σ (I)]	R_1 = 0.0432, wR_2 = 0.1129	R_1 = 0.0480, wR_2 = 0.1087	R_1 = 0.0368, wR_2 = 0.0853
Final R indexes [all data]	R_1 = 0.0495, wR_2 = 0.1185	R_1 = 0.0710, wR_2 = 0.1207	R_1 = 0.0537, wR_2 = 0.0928
Largest diff. peak/hole / e Å^{-3}	0.96/-1.00	0.53/-0.42	0.47/-0.34

1.5. Computational Details

DFT calculations were performed with Gaussian 16 (C.01).[5] Cu, Al, and Si centres were described with the Stuttgart RECPs and associated basis sets,[6] while 6-31G** was employed for the remaining atoms (this basis set combination will herein, and throughout the manuscript is referred to as "BS1").[7,8] A polarization function was also added to Al (ζd = 0.190) and Si (ζd = 0.284).[9] Initial BP86 optimizations[10,11] were performed using the 'grid = ultrafine' option, with all stationary points being fully characterized via analytical frequency calculations as minima or transition states (all positive eigenvalues or one imaginary eigenvalue respectively). All energies were recomputed with a larger basis set featuring 6-311++G** basis sets on all atoms except Cu, for which cc-pVTZ-pp was employed (referred to as "BS2" throughout).[12,13] Corrections for the effect of benzene (ε = 2.3741) solvent were employed using the polarizable continuum model and BS1.[14] Single-point dispersion corrections to the BP86 results employed Grimme's D3 parameter set with Becke-Johnson damping as implemented in Gaussian.[14]

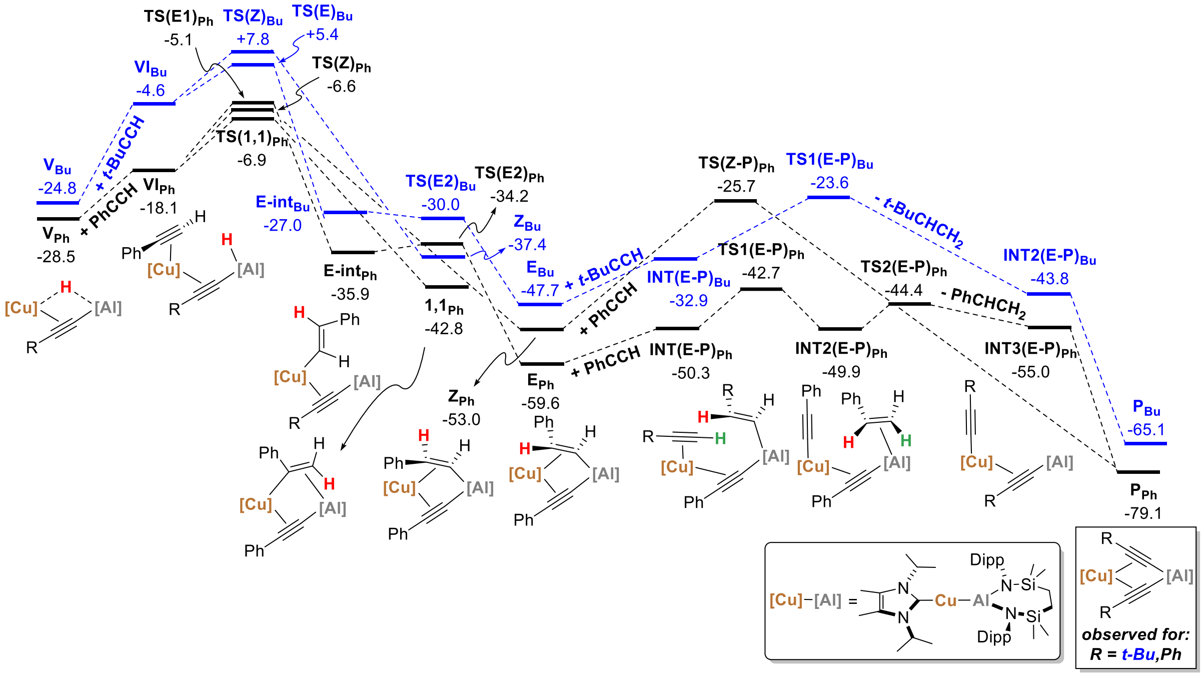

Figure S52. Full computed free energy profile (BP86-D3BJ,C_6H_6/BS2//BP86/BS1 level, energies quoted in kcal mol^{-1}) of onwards formation of **P_{Ph}** (black) and **P_{Bu}** (blue) from **V_{Ph}** and **V_{Bu}**, respectively, as well as alternative alkyne addition and H transfer processes to form **$1,1_{Ph}$**, **Z_{Ph}** and **Z_{Bu}**.

Breakdown of Energy Contributions

The following tables detail the evolution of the relative energies as the successive corrections to the initial SCF energy are included. Terms used are:

ΔE_{BS1} SCF energy computed with the BP86 functional with BS1

ΔH_{BS1} Enthalpy at 298.15 K with BS1

ΔG_{BS1} Free energy at 298.15 K and 1 atm with BS1

$\Delta G_{BS1/bnz}$ Free energy corrected for benzene solvent with BS1

$\Delta G_{BS1/bnz+D3BJ}$ Free energy corrected for benzene and dispersion effects with BS1

ΔE_{BS2} SCF energy computed with the BP86 functional with BS2

ΔG_{bnz} Free energy corrected for basis set (BS2), dispersion effects and benzene solvent

Table S2. Energies breakdown table (kcal/mol) of all stationary points in the free energy profiles in Figures 5, 6 and S52. In each case the final data used in the main article are highlighted in bold.

	ΔE_{BS1}	ΔH_{BS1}	ΔG_{BS1}	$\Delta G_{BS1/bnz}$	$\Delta G_{BS1/bnz+D3}$	ΔE_{BS2}	ΔG_{bnz}
**I**	0.0	0.0	0.0	0.0	0.0	0.0	0.0
**TS(I-III)$_{Ph}$**	21.7	22.4	38.8	39.8	15.8	25.9	20.0
**TS(I-II)$_{Ph}$**	6.1	6.9	20.7	21.6	7.2	8.8	10.0
**II$_{Ph}$**	-3.5	-1.8	13.1	13.4	-5.8	-2.7	-5.0
**TS(II-INT)$_{Ph}$**	-3.5	-2.3	14.8	16.0	-2.8	-2.8	-2.2
**INT$_{Ph}$**	-3.8	-2.0	13.3	14.4	-4.0	-2.9	-3.1
**TS(II-II)$_{Ph}$**	-1.5	-0.6	15.5	16.3	-1.3	0.7	0.9
**III$_{Ph}$**	-23.4	-21.4	-8.3	-8.5	-17.8	-20.8	-15.2
**TS(III-IV)$_{Ph}$**	1.7	0.3	12.8	12.3	5.6	3.7	7.6
**IV$_{Ph}$**	-13.0	-13.2	1.0	0.2	-17.2	-10.9	-15.2
**TS(IV-V)$_{Ph}$**	-10.1	-10.9	4.8	3.7	-10.2	-8.7	-8.8
**V$_{Ph}$**	-27.7	-27.6	-12.7	-12.0	-29.8	-26.4	-28.5
**VI$_{Ph}$**	-15.6	-14.7	11.4	12.2	-23.0	-10.6	-18.1
**TS(E)$_{Ph}$**	-2.2	-2.5	24.6	25.3	-9.7	2.3	-5.1
**E-INT1$_{Ph}$**	-42.9	-39.0	-13.5	-12.8	-39.3	-39.4	-35.9
**TS(E2)$_{Ph}$**	-41.9	-38.3	-10.8	-9.6	-38.3	-37.8	-34.2
**E$_{Ph}$**	-63.2	-58.8	-29.4	-27.9	-67.2	-55.7	-59.6
**INT(E-P)$_{Ph}$**	-55.8	-50.6	-11.1	-10.3	-59.9	-46.1	-50.3
**TS(E-P)$_{Ph}$**	-48.8	-46.3	-3.9	-1.9	-52.8	-38.7	-42.7
**INT2(E-P)$_{Ph}$**	-56.6	-50.4	-10.2	-8.4	-58.6	-48.0	-49.9
**TS2(E-P)$_{Ph}$**	-54.1	-48.2	-8.1	-6.3	-52.5	-45.9	-44.4
**INT2(E-P)$_{Ph}$**	-65.6	-60.5	-36.2	-35.3	-60.0	-60.6	-55.0
**P$_{Ph}$**	-85.7	-80.7	-52.2	-50.7	-87.6	-77.2	-79.1
**TS(1,1)$_{Ph}$**	-5.8	-5.6	22.5	24.0	-13.0	0.3	-6.9
**1,1$_{Ph}$**	-44.9	-41.2	-11.8	-10.2	-49.0	-38.7	-42.8

**TS(Z)$_{Ph}$**	-2.7	-2.9	24.9	26.4	-12.6	3.3	-6.6
**Z$_{Ph}$**	-56.0	-51.6	-22.3	-20.9	-60.6	-48.4	-53.0
**TS(Z-P)Ph**	-24.6	-23.5	20.4	22.4	-36.6	-13.8	-25.7
**TS(I-II)$_{Bu}$**	11.1	11.9	25.7	26.5	11.3	14.1	14.3
**II$_{Bu}$**	0.7	2.3	17.9	19.1	-0.9	2.4	0.8
**TS(II-III)$_{Bu}$**	3.5	4.3	22.1	23.1	4.4	6.7	7.6
**III)$_{Bu}$**	-18.2	-16.6	-2.7	-2.4	-13.6	-14.8	-10.2
**TS(I-III)$_{Bu}$**	25.4	25.9	44.2	44.9	19.8	30.1	24.5
**III$_{Bu}$**	-18.2	-16.6	-2.7	-2.4	-13.6	-14.8	-10.1
**TS(III-IV)$_{Bu}$**	4.6	3.0	17.1	17.0	7.9	7.2	10.6
**IV$_{Bu}$**	-11.2	-11.5	2.2	1.0	-13.1	-8.8	-10.7
**V$_{Bu}$**	-24.5	-24.4	-8.1	-7.4	-27.1	-22.2	-24.8
**VI$_{Bu}$**	-5.2	-4.2	23.7	23.5	-11.0	1.2	-4.6
**TS(E)$_{Bu}$**	3.9	3.7	34.3	34.8	-0.9	10.2	5.4
**E-int$_{Bu}$**	-36.7	-32.8	-3.9	-2.2	-33.3	-30.4	-27.0
**TS(E2)$_{Bu}$**	-35.7	-32.2	-0.4	1.3	-37.6	-28.0	-30.0
**E$_{Bu}$**	-51.3	-47.1	-16.5	-15.3	-57.1	-41.9	-47.7
**INT(E-P)$_{Bu}$**	-39.5	-34.6	7.9	8.6	-45.5	-26.8	-32.9
**TS1(E-P)$_{Bu}$**	-30.2	-27.9	18.1	19.8	-36.6	-17.2	-23.6
**INT2(E-P)$_{Bu}$**	-57.3	-52.2	-23.9	-22.1	-51.6	-49.4	-43.8
**P$_{Bu}$**	-73.1	-68.1	-37.7	-36.6	-75.2	-63.0	-65.1
**TS(Z)$_{Bu}$**	9.4	9.3	39.6	40.6	0.1	17.1	7.8
**Z$_{Bu}$**	-40.9	-36.6	-6.2	-5.1	-46.6	-31.8	-37.4

Cartesian Coordinates and Energies

PhCCH

SCF (BP86) Energy = -308.384259635
Enthalpy 0K = -308.278156
Enthalpy 298K = -308.270491
Free Energy 298K = -308.308773
Lowest Frequency = 139.6409 cm^{-1}
Second Frequency = 150.8438 cm^{-1}
SCF (BP86-D3BJ) Energy = -308.406625348
SCF (C6H6) Energy = -308.386067353
SCF (BS2) Energy = -308.463936022

```
C   0.59981  0.00003 -0.00005
C  -0.12011  1.22148 -0.00004
H   0.43363  2.16500 -0.00009
C   2.02973  0.00002 -0.00001
C  -0.12004 -1.22147 -0.00002
H   0.43374 -2.16496 -0.00002
C   3.25261 -0.00002  0.00005
C  -1.52033 -1.21573  0.00000
H  -2.06498 -2.16566 -0.00008
C  -2.22515 -0.00006  0.00004
H  -3.32000 -0.00003  0.00015
C  -1.52036  1.21573  0.00001
H  -2.06508  2.16562  0.00003
H   4.32575  0.00009  0.00010
```

HPhCCH$_2$

SCF (BP86) Energy = -309.639886019
Enthalpy 0K = -309.510361
Enthalpy 298K = -309.502438
Free Energy 298K = -309.541911
Lowest Frequency = 46.8166 cm^{-1}
Second Frequency = 200.8464 cm^{-1}
SCF (BP86-D3BJ) Energy = -309.664911102
SCF (C6H6) Energy = -309.641276383
SCF (BS2) Energy = -309.716310235

```
C   1.79075 -1.05182  0.00018
C   2.27665  0.26478  0.00015
C   0.40909 -1.28967 -0.00003
H   3.35471  0.45613  0.00025
H   0.03361 -2.31985 -0.00003
C   1.36717  1.33822 -0.00007
C  -0.52001 -0.22268 -0.00012
H   1.73781  2.36894 -0.00020
C  -0.01147  1.09908 -0.00024
H  -0.70392  1.94729 -0.00055
H   2.48838 -1.89608  0.00028
C  -1.96058 -0.53379 -0.00023
H  -2.19345 -1.60801 -0.00093
C  -2.99166  0.33698  0.00036
H  -2.85088  1.42295  0.00110
H  -4.02599 -0.01801  0.00008
```

***t*-BuCCH**

SCF (BP86) Energy = -234.586239247
Enthalpy 0K = -234.449586
Enthalpy 298K = -234.440796
Free Energy 298K = -234.480163
Lowest Frequency = 169.1734 cm^{-1}
Second Frequency = 169.3137 cm^{-1}
SCF (BP86-D3BJ) Energy = -234.606343592
SCF (C6H6) Energy = -234.587510303
SCF (BS2) Energy = -234.647660256

```
C  -1.17636  0.00006  0.00001
C  -2.39700  0.00004  0.00005
H  -3.47013 -0.00003  0.00005
C   0.29934  0.00001 -0.00003
C   0.81206  0.25834  1.44227
H   1.91626  0.26087  1.45420
H   0.45810  1.23236  1.81822
H   0.45868 -0.52496  2.13285
C   0.81200  1.11988 -0.94489
H   0.45916  2.10963 -0.61136
H   1.91621  1.12857 -0.95366
H   0.45743  0.95887 -1.97625
C   0.81192 -1.37828 -0.49741
H   1.91612 -1.39005 -0.50114
H   0.45782 -2.19082  0.15814
H   0.45854 -1.58466 -1.52107
```

***t*-BuHCCH$_2$**

SCF (BP86) Energy = -235.840752982
Enthalpy 0K = -235.680612
Enthalpy 298K = -235.671784
Free Energy 298K = -235.711379
Lowest Frequency = 102.8285 cm^{-1}
Second Frequency = 228.1389 cm^{-1}
SCF (BP86-D3BJ) Energy = -235.863887196
SCF (C6H6) Energy = -235.841261783
SCF (BS2) Energy = -235.898749957

```
C  -1.00872  0.67322  0.00002
H  -0.96247  1.77377  0.00004
C  -2.21645  0.08531  0.00002
H  -2.33995 -1.00265  0.00001
H  -3.13608  0.67941  0.00002
C   0.35479 -0.00249  0.00001
C   1.12695  0.46412  1.26230
H   2.14818  0.04272  1.27177
H   1.21630  1.56454  1.29236
H   0.61241  0.14140  2.18346
C   1.12670  0.46369 -1.26261
H   2.14792  0.04229 -1.27213
H   0.61197  0.14065 -2.18354
H   1.21603  1.56411 -1.29306
C   0.24977 -1.54020  0.00027
H  -0.28436 -1.90738  0.89369
H  -0.28455 -1.90766 -0.89293
H   1.25639 -1.99311  0.00025
```

TS(I-III)$_{Ph}$

SCF (BP86) Energy = -2339.39427809
Enthalpy 0K = -2338.281759
Enthalpy 298K = -2338.209426
Free Energy 298K = -2338.387728
Lowest Frequency = -260.1585 cm^{-1}
Second Frequency = 11.8749 cm^{-1}
SCF (BP86-D3BJ) Energy = -2339.77270026
SCF (C6H6) Energy = -2339.39854675
SCF (BS2) Energy = -3151.59032159

```
Cu -1.35951 -0.08342 -0.05529
Si  3.69122 -0.91434 -1.71796
Si  3.58617  2.18993  0.68223
Al  1.07692  0.06546  0.15184
N   2.28506 -1.22644 -0.65109
N   1.86149  1.81893  0.32516
N  -3.95163  1.32034 -0.60755
N  -4.28991 -0.82120 -0.70020
C   1.82002 -2.58713 -0.59420
C   2.26132 -3.47777  0.44522
C   1.77868 -4.80279  0.47923
H   2.13030 -5.47425  1.27122
C   0.87246 -5.28379 -0.47250
H   0.51140 -6.31723 -0.42591
C   0.45949 -4.42956 -1.50150
H  -0.22060 -4.80849 -2.27442
C   0.92291 -3.10233 -1.59203
C   3.29096 -3.06559  1.50107
H   3.41189 -1.97211  1.40793
C   2.84806 -3.40182  2.94342
H   1.84934 -3.00128  3.17536
H   3.56584 -2.98212  3.67131
H   2.82250 -4.49321  3.11321
C   4.66666 -3.72238  1.23646
H   4.59339 -4.82330  1.29711
H   5.40531 -3.39364  1.98966
H   5.05694 -3.46943  0.23973
C   0.51794 -2.28176 -2.81826
H   0.94545 -1.27405 -2.68731
```

C -1.00733 -2.11897 -2.95442
H -1.51310 -3.09559 -3.06020
H -1.25551 -1.51463 -3.84583
H -1.41918 -1.60073 -2.06797
C   1.10087 -2.89694 -4.11283
H   2.19536 -3.01404 -4.05498
H   0.86842 -2.26056 -4.98583
H   0.67158 -3.89670 -4.30616
C   4.51316 -2.55312 -2.27941
H   5.39357 -2.79959 -1.66291
H   4.86688 -2.43805 -3.31922
H   3.82854 -3.41525 -2.24724
C   3.27914 0.01005 -3.34487
H   2.77298 -0.65172 -4.06633
H   4.22317 0.34783 -3.81112
H   2.64473 0.89688 -3.19868
C   5.05685 0.10372 -0.85091
H   5.29119 -0.37468 0.11927
H   5.95847 -0.05593 -1.47915
C   4.80626 1.61611 -0.67530
H   4.49377 2.07139 -1.63475
H   5.75582 2.12753 -0.40941
C   4.22844 1.43552 2.32216
H   3.60556 1.71563 3.18800
H   5.24885 1.81461 2.51099
H   4.29860 0.33544 2.27819
C   3.83644 4.07990 0.77811
H   3.61699 4.55931 -0.19053
H   4.89072 4.29172 1.02979
H   3.19873 4.56611 1.53232
C   1.02221 2.98993 0.27789
C   0.48015 3.57062 1.47224
C -0.23904 4.78083 1.39810
H -0.62742 5.22375 2.32354
C -0.45016 5.43934 0.18129
H -0.98988 6.39239 0.14845
C   0.05033 4.86131 -0.99223
H -0.10570 5.36828 -1.95213
C   0.77582 3.65494 -0.96954
C   0.64748 2.91846 2.84500
H   1.26090 2.01482 2.69505
C -0.71765 2.46329 3.40578
H -1.38812 3.32376 3.58250
H -0.58722 1.93787 4.36974
H -1.21998 1.78010 2.69946
C   1.36548 3.82872 3.86640
H   2.36833 4.12516 3.51751
H   1.48399 3.30597 4.83259
H   0.79361 4.75368 4.06121
C   1.29454 3.08190 -2.28432
H   1.98592 2.27050 -2.00333
C   0.14669 2.45605 -3.10917
H -0.38250 1.68262 -2.52355
H   0.53443 1.98435 -4.03012
H -0.58894 3.22499 -3.40799
C   2.08128 4.10489 -3.13061
H   1.43630 4.92538 -3.49333
H   2.51506 3.61342 -4.01944
H   2.90666 4.55848 -2.55591
C -3.31367 0.11411 -0.43656
C -3.24184 2.60637 -0.37539
H -2.18794 2.29344 -0.24472
C -3.31243 3.54162 -1.59142
H -3.05755 3.00288 -2.51836
H -2.57377 4.34637 -1.45220
H -4.30383 4.00754 -1.71156
C -3.70052 3.27544 0.92987
H -3.63498 2.56955 1.77329
H -4.73551 3.65331 0.86851
H -3.03622 4.12827 1.14313
C -5.29586 1.14958 -0.96305
C -6.27221 2.25105 -1.24750
H -6.25022 3.03988 -0.47851
H -7.29600 1.84562 -1.26957
H -6.08972 2.73334 -2.22394
C -5.51160 -0.21274 -1.02561
C -6.77053 -0.92469 -1.42025
H -7.04767 -1.72546 -0.71570
H -6.69864 -1.37634 -2.42557
H -7.60785 -0.20986 -1.44578
C -4.03357 -2.27661 -0.55851
H -2.94994 -2.32171 -0.34947
C -4.77666 -2.86252 0.65273
H -4.55051 -2.29121 1.56553
H -4.44707 -3.90156 0.81461
H -5.86913 -2.87979 0.50328
C -4.31297 -3.05421 -1.85550
H -5.39154 -3.17474 -2.04659
H -3.87978 -4.06444 -1.76883
H -3.85166 -2.55998 -2.72429
C   0.24328 -0.92401 2.20824
C   1.40174 -0.45098 2.32818
H   2.30845 -0.31991 2.90122
C -0.92031 -1.70812 2.55431
C -1.83044 -1.22181 3.52750
C -1.04395 -3.04463 2.09400
C -2.81581 -2.06788 4.05411
C -2.02747 -3.88039 2.63722
C -2.90983 -3.40212 3.62290
H -1.73145 -0.19306 3.88328
H -0.34044 -3.42089 1.34553
H -3.50120 -1.68715 4.81883
H -2.09216 -4.91993 2.29872
H -3.66874 -4.06547 4.05085

TS(I-II)$_{Ph}$
SCF (BP86) Energy = -2339.41916970
Enthalpy 0K = -2338.307146
Enthalpy 298K = -2338.234110
Free Energy 298K = -2338.416624
Lowest Frequency = -28.4714 cm^{-1}
Second Frequency = 13.8854 cm^{-1}
SCF (BP86-D3BJ) Energy = -2339.78221749
SCF (C6H6) Energy = -2339.42349916
SCF (BS2) Energy = -3151.61758361

Cu 0.96066 -0.74376 0.16531
Si -2.83374 2.96325 -1.40331
Si -4.12269 0.71039 1.54089
Al -1.16594 0.40882 0.05919
N -1.38169 2.10510 -0.77740
N -2.80053 -0.24971 0.79225
N   1.92653 -3.38400 -0.85498
N   3.56282 -1.95924 -0.94964
C -0.11925 2.73134 -1.09365
C   0.45020 3.69070 -0.19437
C   1.69853 4.26813 -0.49732
H   2.12463 5.00773 0.19081
C   2.39569 3.93582 -1.66685
H   3.36145 4.40305 -1.88976
C   1.82733 3.02076 -2.55973
H   2.35558 2.77710 -3.48957
C   0.57906 2.41756 -2.30455
C -0.27236 4.10260 1.08988
H -1.31836 3.76603 0.98214
C   0.31596 3.37739 2.31992
H   0.29895 2.28262 2.18608
H -0.24976 3.62801 3.23555
H   1.36934 3.66838 2.48329
C -0.28271 5.62872 1.32152
H   0.72719 6.02021 1.53912
H -0.91916 5.87816 2.18868
H -0.66927 6.17511 0.44456
C -0.01130 1.48427 -3.36372
H -0.97511 1.12140 -2.96842
C   0.87235 0.24766 -3.63030
H   1.84878 0.53610 -4.06125
H   0.38285 -0.42978 -4.35339
H   1.05502 -0.31480 -2.69727
C -0.28717 2.23728 -4.68611
H -0.94037 3.11219 -4.52994
H -0.77575 1.57126 -5.41994
H   0.65058 2.60287 -5.14198
C -2.35362 4.70910 -2.00303

H -3.22771 5.17924 -2.48757
H -1.52910 4.68872 -2.73377
H -2.04200 5.35813 -1.16831
C -3.65089 2.07395 -2.88567
H -3.01204 2.11000 -3.78299
H -4.60596 2.57111 -3.13470
H -3.87137 1.01603 -2.67141
C -4.19130 3.19782 -0.07986
H -3.75157 3.70774 0.79838
H -4.88412 3.93991 -0.52929
C -4.97767 1.94306 0.35365
H -5.34290 1.38488 -0.52977
H -5.89506 2.24466 0.90221
C -3.44940 1.72403 3.01229
H -3.09623 1.07324 3.82901
H -4.22929 2.39058 3.42186
H -2.60100 2.35658 2.69869
C -5.52912 -0.44298 2.11761
H -6.08215 -0.83925 1.24878
H -6.24335 0.12737 2.73737
H -5.17850 -1.30660 2.70262
C -2.95945 -1.68089 0.83758
C -2.55562 -2.45077 1.97899
C -2.79122 -3.84118 1.99695
H -2.50425 -4.41619 2.88561
C -3.38760 -4.50398 0.91827
H -3.57056 -5.58338 0.95990
C -3.75460 -3.76134 -0.21060
H -4.22420 -4.26994 -1.06099
C -3.55955 -2.36820 -0.26957
C -1.87422 -1.82380 3.19796
H -1.65128 -0.77420 2.93243
C -0.54525 -2.53731 3.53670
H -0.71824 -3.57242 3.88067
H -0.01479 -2.01236 4.35191
H 0.12193 -2.58900 2.65948
C -2.78293 -1.82043 4.44953
H -3.71529 -1.25851 4.28305
H -2.25985 -1.36322 5.30873
H -3.06011 -2.85066 4.73697
C -3.99546 -1.61692 -1.52611
H -3.94149 -0.54372 -1.27648
C -3.01682 -1.87402 -2.69433
H -1.98558 -1.58742 -2.42037
H -3.30701 -1.29460 -3.58905
H -3.00459 -2.94343 -2.97227
C -5.44623 -1.93161 -1.94896
H -5.56545 -2.98118 -2.27243
H -5.74534 -1.29257 -2.79856
H -6.15543 -1.75506 -1.12262
C 2.25809 -2.08822 -0.53462
C 0.59097 -3.93448 -0.50294
H 0.05108 -3.04406 -0.12547
C -0.17739 -4.46095 -1.72382
H -0.16653 -3.72477 -2.54354
H -1.22647 -4.62769 -1.42976
H 0.22452 -5.41680 -2.09840
C 0.67909 -4.95766 0.64055
H 1.25780 -4.55187 1.48608
H 1.14413 -5.90612 0.32173
H -0.33984 -5.18166 0.99622
C 2.99718 -4.05387 -1.46270
C 2.97040 -5.47046 -1.95176
H 2.63811 -6.17749 -1.17354
H 3.98165 -5.77896 -2.25888
H 2.30640 -5.60109 -2.82400
C 4.03733 -3.14715 -1.52717
C 5.41286 -3.35852 -2.08445
H 6.18711 -3.39459 -1.29808
H 5.70219 -2.57051 -2.79914
H 5.45151 -4.31611 -2.62650
C 4.30020 -0.68105 -0.77448
H 3.59467 -0.06937 -0.18629
C 5.58326 -0.85750 0.05278
H 5.38659 -1.43304 0.97114
H 5.95697 0.13511 0.35106
H 6.38336 -1.35797 -0.51660
C 4.54894 0.03659 -2.11084
H 5.28631 -0.49063 -2.73954
H 4.94346 1.04598 -1.90893
H 3.61179 0.14427 -2.67698
C 2.77761 0.49519 2.39029
C 4.00863 1.20671 2.42490
C 1.69807 -0.10629 2.24910
H 0.79231 -0.50670 2.67711
C 5.41197 3.07666 1.70601
H 5.54613 3.97856 1.09936
C 4.19735 2.38203 1.64630
H 3.38686 2.73059 0.99798
C 5.06826 0.76179 3.26256
H 4.92312 -0.13852 3.86743
C 6.45256 2.62846 2.53925
H 7.39725 3.18012 2.58457
C 6.27353 1.47068 3.31742
H 7.07899 1.11907 3.97079

II_{Ph}

SCF (BP86) Energy = -2339.43439826
Enthalpy 0K = -2338.320602
Enthalpy 298K = -2338.247942
Free Energy 298K = -2338.428634
Lowest Frequency = 11.7563 cm^{-1}
Second Frequency = 15.7290 cm^{-1}
SCF (BP86-D3BJ) Energy = -2339.80501031
SCF (C6H6) Energy = -2339.43990402
SCF (BS2) Energy = -3151.63590605

Cu -1.22879 0.36112 0.16027
Si 3.21058 -2.45088 -1.30202
Si 3.96326 0.15400 1.53904
Al 1.09962 -0.22893 -0.03446
N 1.60261 -1.93474 -0.67083
N 2.49717 0.79216 0.71012
N -2.45970 2.53380 -1.44518
N -3.71030 0.74912 -1.38686
C 0.48994 -2.83206 -0.89093
C 0.10998 -3.77521 0.12072
C -0.98916 -4.62619 -0.10748
H -1.26910 -5.35124 0.66548
C -1.72030 -4.57960 -1.30030
H -2.56749 -5.25598 -1.45893
C -1.33845 -3.67342 -2.29557
H -1.89026 -3.65042 -3.24298
C -0.24528 -2.80100 -2.12102
C 0.89903 -3.93647 1.42340
H 1.56849 -3.06009 1.49761
C 0.00911 -3.98568 2.68480
H -0.67461 -3.12515 2.74542
H 0.63718 -3.99263 3.59332
H -0.60375 -4.90448 2.71297
C 1.78774 -5.20291 1.38535
H 1.16565 -6.11135 1.29333
H 2.37833 -5.29210 2.31478
H 2.48538 -5.19036 0.53470
C 0.15525 -1.89698 -3.28989
H 0.97632 -1.25212 -2.93318
C -0.98564 -0.96926 -3.75658
H -1.83659 -1.54551 -4.16378
H -0.63127 -0.30063 -4.56184
H -1.35140 -0.34486 -2.92487
C 0.68197 -2.72983 -4.48265
H 1.52438 -3.37802 -4.19082
H 1.02297 -2.06900 -5.30003
H -0.11123 -3.38198 -4.89047
C 3.15468 -4.25707 -1.93587
H 3.62725 -4.95233 -1.22210
H 3.71887 -4.32791 -2.88222
H 2.12977 -4.61355 -2.12385
C 3.81052 -1.39233 -2.77522
H 3.20544 -1.57905 -3.67746
H 4.85533 -1.66421 -3.01296
H 3.78679 -0.31138 -2.56930
C 4.56047 -2.36079 0.04653
H 4.20478 -2.91653 0.93506

H 5.40059 -2.96355 -0.35783
C 5.06949 -0.95785 0.44209
H 5.35664 -0.38043 -0.45767
H 6.00580 -1.05251 1.03197
C 3.47953 -0.86698 3.07873
H 2.98734 -0.24409 3.84356
H 4.37686 -1.31947 3.53746
H 2.79284 -1.69277 2.82259
C 5.09251 1.59960 2.05771
H 5.56108 2.06636 1.17476
H 5.90193 1.21860 2.70495
H 4.56012 2.39294 2.60414
C 2.35267 2.23114 0.68829
C 1.75104 2.94109 1.77903
C 1.67045 4.34830 1.72793
H 1.22483 4.88289 2.57520
C 2.14830 5.07771 0.63389
H 2.08177 6.17121 0.62126
C 2.72740 4.38657 -0.43699
H 3.11679 4.94855 -1.29421
C 2.84935 2.98363 -0.42689
C 1.19726 2.24031 3.02182
H 1.25889 1.15449 2.83743
C -0.28628 2.59225 3.26768
H -0.41643 3.66470 3.49896
H -0.67974 2.01638 4.12366
H -0.90364 2.35046 2.38687
C 2.02339 2.56167 4.28962
H 3.07859 2.26202 4.18369
H 1.60682 2.03383 5.16609
H 2.00532 3.64349 4.51298
C 3.52135 2.29917 -1.61545
H 3.68782 1.25222 -1.31112
C 2.59619 2.28661 -2.85304
H 1.63403 1.79258 -2.62753
H 3.06745 1.74941 -3.69538
H 2.37419 3.31501 -3.19005
C 4.89344 2.91166 -1.97012
H 4.79993 3.94640 -2.34525
H 5.38371 2.31994 -2.76323
H 5.56558 2.93437 -1.09580
C -2.50328 1.25074 -0.95637
C -1.33695 3.44589 -1.10637
H -0.58654 2.76107 -0.66677
C -0.70426 4.10582 -2.34036
H -0.49716 3.36257 -3.12699
H 0.25481 4.55518 -2.03613
H -1.33109 4.90683 -2.76409
C -1.75096 4.46142 -0.02900
H -2.19579 3.94931 0.83921
H -2.47835 5.19914 -0.40989
H -0.85655 5.00542 0.31530
C -3.62350 2.83790 -2.16700
C -3.90149 4.14219 -2.85035
H -3.72799 5.00631 -2.18807
H -4.95583 4.17955 -3.16584
H -3.28300 4.28586 -3.75387
C -4.41268 1.70560 -2.13487
C -5.73607 1.48199 -2.80138
H -6.48979 1.06158 -2.11566
H -5.65744 0.80153 -3.66758
H -6.13126 2.43985 -3.17394
C -4.19991 -0.58422 -0.94144
H -3.31962 -1.01455 -0.43261
C -5.32044 -0.44251 0.10199
H -5.00313 0.20958 0.93020
H -5.54901 -1.43283 0.52809
H -6.25040 -0.04068 -0.33517
C -4.57642 -1.50705 -2.11035
H -5.52486 -1.21643 -2.59048
H -4.70008 -2.53234 -1.72521
H -3.78123 -1.52728 -2.87122
C -1.80360 -0.49318 1.92345
C -2.91090 -0.76939 2.80000
C -0.53076 -0.63990 1.66750
H 0.32133 -1.04710 2.22870
C -4.57378 -2.34019 3.69243
H -4.98429 -3.35574 3.72899
C -3.47410 -2.07597 2.86616
H -3.03395 -2.87351 2.25920
C -3.51025 0.25427 3.58602
H -3.10087 1.26806 3.53659
C -5.14424 -1.32311 4.47769
H -6.00096 -1.53596 5.12499
C -4.60008 -0.02740 4.41824
H -5.03313 0.77623 5.02458

TS(II-INT)$_{Ph}$
SCF (BP86) Energy = -2339.43433110
Enthalpy 0K = -2338.320554
Enthalpy 298K = -2338.248759
Free Energy 298K = -2338.425881
Lowest Frequency = -5.0704 cm^{-1}
Second Frequency = 13.8268 cm^{-1}
SCF (BP86-D3BJ) Energy = -2339.80436868
SCF (C6H6) Energy = -2339.43841358
SCF (BS2) Energy = -3151.63610354

Cu 1.24456 -0.29002 0.17394
Si -3.36270 2.45583 -0.92502
Si -3.88210 -0.44198 1.66584
Al -1.10241 0.20438 0.02505
N -1.70549 1.93256 -0.44021
N -2.44210 -0.94047 0.70668
N 2.50315 -2.17497 -1.74587
N 3.67477 -0.35251 -1.50861
C -0.66433 2.91318 -0.66083
C -0.26995 3.79984 0.39544
C 0.72543 4.76586 0.14826
H 1.01399 5.44941 0.95519
C 1.34623 4.88097 -1.10041
H 2.11226 5.64530 -1.27158
C 0.96518 4.01358 -2.12998
H 1.43943 4.10619 -3.11459
C -0.02944 3.03349 -1.94125
C -0.90851 3.75813 1.78852
H -1.50747 2.83041 1.83520
C 0.13841 3.72383 2.92426
H 0.86442 2.90934 2.78170
H -0.35902 3.57673 3.89946
H 0.69824 4.67405 2.98834
C -1.86750 4.94772 2.03140
H -1.33529 5.90959 1.92224
H -2.28135 4.90700 3.05491
H -2.71314 4.95022 1.32708
C -0.41608 2.15820 -3.13786
H -1.20017 1.46348 -2.79174
C 0.76338 1.30236 -3.64801
H 1.58084 1.93618 -4.03816
H 0.43449 0.64761 -4.47544
H 1.16806 0.66667 -2.84335
C -0.99680 2.99508 -4.30176
H -1.87075 3.58954 -3.98864
H -1.31149 2.33784 -5.13227
H -0.24497 3.69824 -4.70275
C -3.38036 4.30169 -1.40817
H -3.26776 4.98255 -0.55089
H -4.34906 4.52477 -1.88994
H -2.58075 4.54579 -2.12548
C -4.03582 1.53889 -2.46045
H -3.48773 1.82816 -3.37199
H -5.09619 1.81363 -2.60827
H -3.98587 0.44366 -2.36889
C -4.62124 2.20306 0.48905
H -4.21860 2.66803 1.40874
H -5.49796 2.82238 0.20688
C -5.07399 0.75113 0.75965
H -5.40400 0.26560 -0.17882
H -5.97401 0.75554 1.41058
C -3.34333 0.42301 3.28100
H -2.79117 -0.26005 3.94712
H -4.22422 0.79603 3.83334
H -2.69522 1.29327 3.07544
C -4.95104 -1.96841 2.06867

H -5.46116 -2.33475 1.16159
H -5.72912 -1.69068 2.80125
H -4.37371 -2.80922 2.48197
C -2.26032 -2.36244 0.51301
C -1.59498 -3.17676 1.48838
C -1.47183 -4.56392 1.26418
H -0.97691 -5.17958 2.02451
C -1.96838 -5.17442 0.10758
H -1.86600 -6.25514 -0.04021
C -2.61380 -4.38197 -0.84880
H -3.02049 -4.85107 -1.75258
C -2.78066 -2.99589 -0.66395
C -1.01471 -2.61316 2.78759
H -1.09884 -1.51497 2.73114
C 0.48074 -2.96213 2.95018
H 0.63438 -4.05013 3.06532
H 0.89445 -2.47017 3.84769
H 1.06506 -2.62029 2.08025
C -1.79492 -3.09451 4.03378
H -2.85622 -2.80040 3.99879
H -1.35475 -2.66831 4.95303
H -1.75692 -4.19485 4.12551
C -3.53033 -2.20260 -1.73206
H -3.71714 -1.20625 -1.29747
C -2.66784 -2.00857 -2.99929
H -1.71768 -1.49711 -2.76224
H -3.20157 -1.40381 -3.75392
H -2.41895 -2.98136 -3.46000
C -4.89683 -2.82243 -2.09605
H -4.78703 -3.79979 -2.59887
H -5.44632 -2.15943 -2.78749
H -5.52271 -2.97732 -1.20105
C 2.51656 -0.96705 -1.09254
C 1.43424 -3.17337 -1.48564
H 0.68323 -2.58845 -0.92042
C 0.76112 -3.68217 -2.76890
H 0.48760 -2.84612 -3.43241
H -0.16477 -4.20882 -2.48650
H 1.39268 -4.38968 -3.33004
C 1.93817 -4.31029 -0.58190
H 2.41350 -3.90615 0.32621
H 2.66627 -4.96047 -1.09718
H 1.08117 -4.93082 -0.27338
C 3.63756 -2.32130 -2.55824
C 3.93382 -3.50884 -3.42271
H 3.83374 -4.46010 -2.87444
H 4.96999 -3.45221 -3.79112
H 3.27350 -3.56289 -4.30620
C 4.37640 -1.16406 -2.41301
C 5.64821 -0.78577 -3.10932
H 6.42221 -0.42523 -2.41227
H 5.49035 0.00128 -3.86774
H 6.06124 -1.66292 -3.63133
C 4.11844 0.94138 -0.92197
H 3.25585 1.23860 -0.30004
C 5.32379 0.74732 0.01324
H 5.11968 -0.03566 0.75969
H 5.51487 1.68588 0.55852
H 6.24371 0.48723 -0.53744
C 4.34349 2.03205 -1.97979
H 5.25401 1.85990 -2.57654
H 4.45920 3.00237 -1.46982
H 3.47912 2.11038 -2.65662
C 1.89893 0.25492 2.03285
C 3.07280 0.34561 2.86041
C 0.62315 0.44252 1.85459
H -0.23006 0.73333 2.47662
C 4.77996 1.69462 3.99811
H 5.17799 2.68113 4.26144
C 3.61985 1.61043 3.21783
H 3.12153 2.52184 2.87330
C 3.74810 -0.82247 3.31285
H 3.34993 -1.80352 3.03627
C 5.42557 0.53197 4.45343
H 6.32883 0.60327 5.06769
C 4.89630 -0.72450 4.10743
H 5.38772 -1.64012 4.45521

INT_{Ph}
SCF (BP86) Energy = -2339.43485699
Enthalpy 0K = -2338.320702
Enthalpy 298K = -2338.248177
Free Energy 298K = -2338.428412
Lowest Frequency = 12.5680 cm^{-1}
Second Frequency = 15.9793 cm^{-1}
SCF (BP86-D3BJ) Energy = -2339.80421328
SCF (C6H6) Energy = -2339.43896522
SCF (BS2) Energy = -3151.63623133

Cu 1.29220 -0.17414 0.13951
Si -3.58785 2.28874 -0.31361
Si -3.72764 -1.18616 1.55055
Al -1.08050 0.13168 0.10403
N -1.85791 1.84038 -0.05912
N -2.27438 -1.28014 0.49602
N 2.77102 -1.42472 -2.09814
N 3.57308 0.53216 -1.56845
C -0.93087 2.94555 -0.18034
C -0.50588 3.66635 0.98390
C 0.34354 4.78066 0.83312
H 0.65090 5.33828 1.72537
C 0.79612 5.19732 -0.42337
H 1.44501 6.07513 -0.51746
C 0.40117 4.47979 -1.55792
H 0.75238 4.79988 -2.54629
C -0.45277 3.36320 -1.46617
C -0.94309 3.27980 2.40065
H -1.44103 2.29645 2.32371
C 0.26149 3.14262 3.35846
H 1.02403 2.46451 2.94540
H -0.07079 2.74482 4.33456
H 0.73917 4.11928 3.55367
C -1.96118 4.27597 3.00356
H -1.54369 5.29840 3.03172
H -2.21424 3.98863 4.03989
H -2.89901 4.31160 2.42774
C -0.85309 2.65300 -2.76236
H -1.55272 1.84595 -2.48714
C 0.35760 1.99391 -3.45940
H 1.09376 2.75234 -3.78373
H 0.03241 1.44466 -4.36156
H 0.86409 1.28407 -2.78441
C -1.57737 3.60068 -3.74599
H -2.46185 4.07362 -3.28764
H -1.91143 3.04767 -4.64214
H -0.91014 4.41163 -4.08889
C -3.78566 4.18495 -0.37043
H -3.52889 4.68666 0.57460
H -4.83949 4.41926 -0.60448
H -3.15735 4.63205 -1.15775
C -4.33006 1.67864 -1.96668
H -3.88387 2.20207 -2.82753
H -5.41345 1.89848 -1.96978
H -4.21139 0.59680 -2.12872
C -4.69739 1.60278 1.08138
H -4.24720 1.85734 2.05935
H -5.63043 2.20077 1.02314
C -5.03744 0.09738 1.00246
H -5.38372 -0.17048 -0.01408
H -5.89953 -0.12651 1.66626
C -3.23612 -0.72253 3.33866
H -2.60725 -1.49487 3.81052
H -4.13726 -0.59089 3.96391
H -2.67956 0.23146 3.36809
C -4.65853 -2.84972 1.52431
H -5.17314 -2.98459 0.55748
H -5.42771 -2.85754 2.31636
H -4.00346 -3.72142 1.67135
C -1.94297 -2.58133 -0.04444
C -1.21447 -3.55680 0.71517
C -0.95249 -4.82098 0.14629
H -0.41142 -5.56485 0.74294
C -1.36814 -5.15381 -1.14731
H -1.15850 -6.14583 -1.56239

C -2.07037 -4.20139 -1.89467
H -2.41270 -4.45300 -2.90555
C -2.37418 -2.93178 -1.36697
C -0.70439 -3.30251 2.13593
H -0.89081 -2.23957 2.36190
C 0.81580 -3.54761 2.25597
H 1.07003 -4.61019 2.09204
H 1.17128 -3.27296 3.26408
H 1.37728 -2.94031 1.52844
C -1.44706 -4.16007 3.18805
H -2.53071 -3.96171 3.19919
H -1.05399 -3.95370 4.19968
H -1.30742 -5.23820 2.99109
C -3.17503 -1.95768 -2.22697
H -3.44949 -1.12183 -1.56206
C -2.31851 -1.38128 -3.37651
H -1.41448 -0.87798 -2.98867
H -2.89273 -0.64447 -3.96604
H -1.98786 -2.18109 -4.06343
C -4.48024 -2.57340 -2.77574
H -4.28210 -3.38404 -3.49944
H -5.07662 -1.80565 -3.29992
H -5.09995 -2.99428 -1.96603
C 2.56448 -0.35169 -1.26756
C 1.88977 -2.61901 -2.03477
H 1.06070 -2.28046 -1.38459
C 1.29105 -2.98937 -3.40016
H 0.87239 -2.10227 -3.90195
H 0.46963 -3.70544 -3.23632
H 2.02555 -3.46407 -4.07089
C 2.58518 -3.80227 -1.34257
H 3.00516 -3.49543 -0.37134
H 3.39707 -4.22764 -1.95735
H 1.84325 -4.59645 -1.15910
C 3.90707 -1.22751 -2.90012
C 4.41308 -2.18966 -3.93126
H 4.48886 -3.21851 -3.54243
H 5.42081 -1.88908 -4.25829
H 3.77264 -2.22036 -4.83058
C 4.41435 0.01176 -2.56457
C 5.60240 0.71550 -3.14653
H 6.27658 1.11340 -2.36992
H 5.31458 1.55729 -3.80073
H 6.18818 0.01168 -3.75829
C 3.72998 1.80101 -0.80762
H 2.77569 1.88105 -0.25681
C 4.86743 1.69722 0.22151
H 4.71247 0.83801 0.89241
H 4.88840 2.61199 0.83727
H 5.85403 1.59855 -0.26322
C 3.85288 3.03424 -1.71579
H 4.84927 3.12099 -2.17828
H 3.68522 3.93753 -1.10760
H 3.08791 3.02109 -2.50736
C 1.84903 -0.18579 2.06346
C 2.87921 -0.33824 3.07396
C 0.55571 -0.00033 1.92014
H -0.32079 0.01746 2.58119
C 3.79593 0.14874 5.29756
H 3.70400 0.65282 6.26615
C 2.78275 0.29662 4.34188
H 1.90355 0.90989 4.56226
C 4.02930 -1.12773 2.81221
H 4.11383 -1.61019 1.83247
C 4.91977 -0.65157 5.02628
H 5.70679 -0.77007 5.77842
C 5.02705 -1.29504 3.78117
H 5.89905 -1.92074 3.56088

TS(II_II)Ph
SCF (BP86) Energy = -2339.43117042
Enthalpy 0K = -2338.317863
Enthalpy 298K = -2338.246017
Free Energy 298K = -2338.424872
Lowest Frequency = -112.1346 cm^{-1}
Second Frequency = 13.0915 cm^{-1}
SCF (BP86-D3BJ) Energy = -2339.79935006
SCF (C6H6) Energy = -2339.43575771
SCF (BS2) Energy = -3151.63050939

Cu -1.54326 0.47873 0.39445
Si 3.00292 -2.82620 -0.47497
Si 4.08994 0.78291 0.82800
Al 1.08162 -0.26994 0.32303
N 1.41057 -2.06443 -0.06327
N 2.47345 0.96395 0.05951
N -2.88416 1.57752 -1.99217
N -3.86676 -0.19951 -1.19263
C 0.30916 -3.00335 0.03480
C -0.09278 -3.54000 1.30266
C -1.10332 -4.52244 1.33916
H -1.39454 -4.94464 2.30723
C -1.73479 -4.97981 0.17833
H -2.50560 -5.75643 0.23410
C -1.36248 -4.43364 -1.05440
H -1.85480 -4.78321 -1.96968
C -0.35511 -3.45443 -1.15307
C 0.52830 -3.10880 2.63626
H 1.10841 -2.18824 2.44654
C -0.54844 -2.77638 3.69215
H -1.26930 -2.03706 3.31028
H -0.07572 -2.35949 4.59854
H -1.10513 -3.67713 4.00666
C 1.50082 -4.16630 3.21021
H 0.99467 -5.13970 3.33980
H 1.87289 -3.84757 4.20031
H 2.37536 -4.32454 2.56001
C 0.00113 -2.92971 -2.54694
H 0.84637 -2.23217 -2.42676
C -1.16411 -2.13582 -3.17739
H -2.04483 -2.78335 -3.34206
H -0.86742 -1.72666 -4.15998
H -1.47243 -1.29777 -2.53069
C 0.44046 -4.06284 -3.50256
H 1.27213 -4.65583 -3.08698
H 0.76740 -3.64707 -4.47248
H -0.39044 -4.76112 -3.70826
C 2.88329 -4.71975 -0.28858
H 2.66478 -5.05356 0.73643
H 3.84975 -5.15985 -0.59253
H 2.10110 -5.13880 -0.94210
C 3.61533 -2.56817 -2.26726
H 2.97983 -3.09337 -2.99726
H 4.63078 -2.99916 -2.34264
H 3.68220 -1.51280 -2.56914
C 4.38819 -2.18103 0.67565
H 4.02879 -2.19211 1.72212
H 5.16915 -2.96802 0.62915
C 5.01041 -0.81193 0.31111
H 5.22277 -0.75874 -0.77338
H 6.00006 -0.70895 0.80426
C 3.93724 0.71394 2.73232
H 3.57783 1.66694 3.15350
H 4.92040 0.49318 3.18518
H 3.24182 -0.07900 3.06037
C 5.22325 2.22360 0.31514
H 5.49194 2.14979 -0.75203
H 6.15781 2.18444 0.90150
H 4.76061 3.21018 0.47079
C 2.21205 2.20669 -0.63827
C 1.80883 3.39678 0.05451
C 1.61718 4.59007 -0.67290
H 1.32195 5.49524 -0.12927
C 1.80577 4.65404 -2.05737
H 1.66483 5.59728 -2.59660
C 2.19245 3.49362 -2.73721
H 2.35521 3.53039 -3.82114
C 2.40141 2.27789 -2.05796
C 1.57940 3.45165 1.56619
H 1.72817 2.43071 1.95427
C 0.13562 3.88025 1.91071
H -0.06479 4.91711 1.58576
H -0.03233 3.83216 3.00034
H -0.60207 3.21744 1.43024

```
C   2.58641  4.38321  2.28050
H   3.63067  4.07040  2.11630
H   2.40010  4.38645  3.36921
H   2.49107  5.42483  1.92529
C   2.84529  1.06299 -2.86646
H   3.04448  0.26695 -2.13092
C   1.72740  0.56522 -3.80870
H   0.80699  0.32678 -3.24796
H   2.04428 -0.34622 -4.34628
H   1.47247  1.32982 -4.56460
C   4.14626  1.31950 -3.65844
H   4.00417  2.08566 -4.44137
H   4.48240  0.39470 -4.16052
H   4.96044  1.66538 -2.99963
C  -2.72851  0.56781 -1.07094
C  -1.87160  2.65657 -2.12327
H  -1.00642  2.25508 -1.56376
C  -1.42822  2.88890 -3.57575
H  -1.20973  1.93492 -4.08212
H  -0.50155  3.48453 -3.56317
H  -2.17752  3.44105 -4.16531
C  -2.32984  3.94501 -1.42062
H  -2.62366  3.73618 -0.37931
H  -3.18146  4.42000 -1.93770
H  -1.49423  4.66368 -1.40494
C  -4.11492  1.46634 -2.65712
C  -4.60924  2.39696 -3.72245
H  -4.52415  3.45526 -3.42453
H  -5.67372  2.19965 -3.92396
H  -4.06670  2.27507 -4.67653
C  -4.73520  0.34070 -2.15102
C  -6.05853 -0.24220 -2.54430
H  -6.69836 -0.45608 -1.67190
H  -5.95155 -1.18022 -3.11674
H  -6.60360  0.47058 -3.18254
C  -4.12396 -1.33432 -0.26337
H  -3.13293 -1.51723  0.18949
C  -5.08222 -0.91124  0.86185
H  -4.67162 -0.04543  1.40509
H  -5.19764 -1.74115  1.57886
H  -6.08442 -0.65475  0.47725
C  -4.56407 -2.61871 -0.98122
H  -5.61721 -2.58604 -1.30337
H  -4.45251 -3.46455 -0.28414
H  -3.92646 -2.82455 -1.85500
C  -1.33038  0.47126  2.31351
C  -1.76919  0.96778  3.61651
C  -0.04461  0.30664  1.89632
H   0.81329  0.70876  2.49376
C  -1.48905  1.19833  6.04619
H  -0.91273  0.98786  6.95448
C  -1.03636  0.72342  4.80878
H  -0.10839  0.14408  4.74994
C  -2.97424  1.70831  3.72439
H  -3.55083  1.88728  2.80951
C  -2.67605  1.94839  6.12705
H  -3.02885  2.31944  7.09532
C  -3.41200  2.20861  4.95736
H  -4.33875  2.79097  5.01193
```

III$_{Ph}$

SCF (BP86) Energy = -2339.46606117
Enthalpy 0K = -2338.351869
Enthalpy 298K = -2338.279212
Free Energy 298K = -2338.462693
Lowest Frequency = 10.6029 cm^{-1}
Second Frequency = 13.9752 cm^{-1}
SCF (BP86-D3BJ) Energy = -2339.82100094
SCF (C6H6) Energy = -2339.47229551
SCF (BS2) Energy = -3151.66478097

```
Cu -2.23491 -0.55044  0.42408
Si  4.02557  0.01270 -2.19559
Al  1.68356  0.28980  0.11105
Si  2.96704  3.27081 -0.24036
N  -5.02494  0.35068 -0.13999
N   3.08624 -0.55153 -0.76955
N  -4.32713 -1.12430 -1.57570
N   1.58360  2.13526 -0.00194
C  -3.91475 -0.37305 -0.50112
H   1.30142 -1.41327  1.97914
C   1.70447 -2.92516 -1.98867
H   1.58478 -1.85200 -2.21638
C   2.71172 -3.03195 -0.84220
C   0.30870  2.75602  0.28999
C   4.30263 -2.07602  0.78191
C   3.94084  2.95382 -1.85510
H   3.23602  2.96389 -2.70832
H   4.55192  3.87297 -1.97628
C  -1.09782 -2.11163  2.48171
C   4.85635  1.71026 -1.91401
H   5.49302  1.64884 -1.01101
H   5.56597  1.80847 -2.76217
C   3.36541 -1.88965 -0.28293
C   4.21382  3.16209  1.19961
H   3.77982  3.53393  2.14166
H   5.10908  3.76971  0.97615
H   4.54967  2.12558  1.37122
C   3.90457 -4.49177  0.72642
H   4.10926 -5.49447  1.11679
C  -3.40787 -2.11272 -2.19815
H  -2.44352 -1.89031 -1.70302
C  -0.06578  3.09685  1.62681
C   5.42138 -1.22602 -2.56651
H   6.17511 -1.23153 -1.76150
H   5.92931 -0.94168 -3.50463
H   5.04881 -2.25675 -2.67604
C  -4.99165  1.23186  1.05725
H  -3.91094  1.30337  1.27998
C   4.54650 -3.37394  1.27007
H   5.25688 -3.50895  2.09380
C  -2.20182 -1.88019  3.34117
H  -2.75604 -0.93931  3.23353
C   0.81720  2.79428  2.83869
H   1.70537  2.25377  2.46769
C  -6.12185  0.04538 -0.95986
C  -0.71060 -1.10240  1.44398
C   0.31660 -3.47331 -1.58894
H   0.36089 -4.54920 -1.34261
H  -0.39303 -3.35755 -2.42946
H  -0.08314 -2.93526 -0.71355
C  -5.67675 -0.88978 -1.87491
C   2.21337 -3.63189 -3.26634
H   3.18418 -3.22911 -3.60061
H   1.49106 -3.51008 -4.09386
H   2.34524 -4.71556 -3.09756
C   2.99862 -4.31064 -0.32390
H   2.49482 -5.18360 -0.75558
C   5.02969 -0.89721  1.43057
H   4.84225 -0.01761  0.79003
C  -0.39691 -3.33546  2.63815
H   0.44704 -3.55011  1.97338
C   0.63180 -0.84480  1.29225
C  -0.57574  3.07483 -0.78954
C  -0.77924 -4.27348  3.60905
H  -0.22623 -5.21583  3.69902
C  -5.68743  0.56398  2.25525
H  -5.54619  1.18293  3.15718
H  -5.25613 -0.43149  2.44805
H  -6.77320  0.44963  2.09488
C  -0.22758  2.69304 -2.22858
H   0.86498  2.53755 -2.25867
C  -1.86366 -4.01316  4.46349
H  -2.15957 -4.74565  5.22239
C  -1.29092  3.75900  1.84465
H  -1.56945  4.03227  2.86926
C   2.91070  0.18770 -3.73661
H   2.56325 -0.79236 -4.10212
H   3.46357  0.67787 -4.55790
H   2.01979  0.80195 -3.52086
C   4.45216 -0.59134  2.83233
H   4.59309 -1.45035  3.51217
H   3.36837 -0.38401  2.79109
H   4.95137  0.28452  3.28414
```

C -6.43520 -1.51705 -3.00494
H -6.12283 -1.12444 -3.98898
H -7.51041 -1.30543 -2.89662
H -6.31929 -2.61302 -3.03251
C -0.89172 1.34916 -2.60798
H -0.62858 0.54890 -1.89081
H -0.58543 1.02302 -3.61869
H -1.99240 1.44080 -2.58891
C 2.30049 5.05023 -0.35178
H 1.68272 5.19491 -1.25372
H 3.14906 5.75467 -0.40492
H 1.68310 5.32146 0.51913
C 0.10344 1.87897 3.85859
H -0.78906 2.37208 4.28469
H 0.77984 1.63889 4.69831
H -0.21264 0.93593 3.38598
C -2.56992 -2.80523 4.32858
H -3.41418 -2.58656 4.99289
C -3.79573 -3.55379 -1.82853
H -4.74869 -3.86534 -2.28867
H -3.87982 -3.66224 -0.73534
H -3.01245 -4.24490 -2.18149
C 1.30182 4.09047 3.52980
H 1.83865 4.75647 2.83314
H 1.98100 3.85218 4.36774
H 0.45262 4.66240 3.94491
C -5.50852 2.64963 0.77172
H -6.60301 2.68777 0.65292
H -5.03477 3.06832 -0.13018
H -5.24676 3.29924 1.62275
C 6.55803 -1.10293 1.50852
H 7.04891 -0.19389 1.89871
H 6.98867 -1.32590 0.51760
H 6.82735 -1.93490 2.18298
C -0.57718 3.77968 -3.26578
H -1.66785 3.92307 -3.36718
H -0.19517 3.49382 -4.26149
H -0.13542 4.75429 -2.99741
C -3.23160 -1.89176 -3.70816
H -2.39097 -2.51000 -4.06478
H -2.99602 -0.83808 -3.92851
H -4.12248 -2.18450 -4.28707
C -1.78801 3.73551 -0.51829
H -2.45393 3.99514 -1.34896
C -7.48566 0.65707 -0.85065
H -7.87291 0.63526 0.18161
H -8.19667 0.09570 -1.47676
H -7.50666 1.70696 -1.19246
C -2.14620 4.08984 0.78872
H -3.07858 4.63198 0.97968

TS(III-IV)$_{Ph}$
SCF (BP86) Energy = -2339.42609115
Enthalpy 0K = -2338.317417
Enthalpy 298K = -2338.244571
Free Energy 298K = -2338.429075
Lowest Frequency = -581.8128 cm^{-1}
Second Frequency = 8.7433 cm^{-1}
SCF (BP86-D3BJ) Energy = -2339.77682576
SCF (C6H6) Energy = -2339.43283547
SCF (BS2) Energy = -3151.62576198

Cu -2.46529 -0.53660 0.42642
Si 3.74179 -0.49269 -2.45192
Al 1.75097 0.21448 0.04827
Si 3.58813 2.86482 -0.33428
N -4.98252 0.81140 -0.35074
N 2.80364 -0.92007 -0.98620
N -4.95453 -1.27803 -0.95815
N 2.01080 2.05099 -0.05791
C -4.18092 -0.30174 -0.38021
H 1.55980 -0.49044 1.60985
C 0.91902 -2.92612 -2.15796
H 0.97184 -1.83536 -2.31691
C 1.95282 -3.27152 -1.08390
C 0.88846 2.89662 0.27098
C 3.77095 -2.69613 0.47933
C 4.38103 2.38457 -2.00807
H 3.65461 2.60247 -2.81444
H 5.19335 3.12965 -2.14329
C -0.92670 -1.64178 2.76709
C 4.95711 0.95794 -2.16274
H 5.60318 0.70138 -1.30132
H 5.62871 0.92316 -3.04649
C 2.84035 -2.29286 -0.53242
C 4.84896 2.45341 1.03854
H 4.54050 2.87885 2.00752
H 5.84128 2.86853 0.78627
H 4.96874 1.36535 1.17517
C 2.93413 -4.99685 0.35551
H 2.97241 -6.03791 0.69429
C -4.43056 -2.66085 -1.12216
H -3.36717 -2.55230 -0.83845
C 0.61864 3.27869 1.62369
C 4.81128 -1.97638 -2.98457
H 5.64391 -2.14220 -2.28096
H 5.24573 -1.77928 -3.98031
H 4.23610 -2.91385 -3.03940
C -4.49566 2.08068 0.25643
H -3.41338 1.89698 0.38621
C 3.79339 -4.03873 0.90435
H 4.50947 -4.33796 1.67914
C -2.03985 -1.54087 3.63562
H -2.88537 -0.91093 3.33488
C 1.46255 2.78374 2.79996
H 2.22008 2.09470 2.38856
C -6.24788 0.53687 -0.88679
C -0.89664 -0.92168 1.48371
C -0.51823 -3.26315 -1.70183
H -0.64822 -4.34872 -1.54024
H -1.24439 -2.95190 -2.47551
H -0.76231 -2.74240 -0.76099
C -6.22937 -0.79043 -1.27557
C 1.22364 -3.62689 -3.50215
H 2.22363 -3.36532 -3.88562
H 0.48040 -3.34151 -4.26883
H 1.18695 -4.72602 -3.39547
C 2.02463 -4.60411 -0.63167
H 1.34655 -5.34937 -1.06463
C 4.75482 -1.71617 1.11996
H 4.58802 -0.73849 0.63445
C 0.15953 -2.46467 3.15382
H 1.01550 -2.56790 2.47925
C 0.05663 -0.39388 0.75580
C 0.03871 3.38777 -0.77128
C 0.13177 -3.14868 4.37711
H 0.97680 -3.78731 4.65676
C -5.10739 2.30667 1.64838
H -4.62440 3.17793 2.12041
H -4.93962 1.42962 2.29414
H -6.19169 2.50565 1.60207
C 0.23812 2.96372 -2.22582
H 1.22133 2.46450 -2.27357
C -0.97113 -3.02446 5.23933
H -0.98806 -3.56139 6.19386
C -0.45724 4.14836 1.89250
H -0.65017 4.44680 2.92984
C 2.60430 -0.00157 -3.90624
H 2.02866 -0.86533 -4.27765
H 3.20367 0.39218 -4.74672
H 1.88382 0.78115 -3.61486
C 4.49387 -1.54691 2.63374
H 4.65415 -2.49607 3.17628
H 3.45844 -1.21695 2.82124
H 5.18024 -0.79670 3.06603
C -7.31624 -1.57619 -1.94399
H -7.10668 -1.75618 -3.01327
H -8.26604 -1.02208 -1.88739
H -7.48066 -2.55628 -1.46722
C -0.83190 1.92680 -2.64218
H -0.83789 1.06058 -1.95748
H -0.65029 1.55761 -3.66784
H -1.84130 2.37732 -2.61907
C 3.32440 4.75022 -0.37802

H 2.71026 5.05329 -1.24226
H 4.30167 5.25713 -0.46122
H 2.82199 5.12089 0.52969
C 0.61660 1.98941 3.81999
H -0.15808 2.62600 4.28505
H 1.25701 1.60056 4.63136
H 0.11505 1.13439 3.34039
C -2.05668 -2.21444 4.86462
H -2.92123 -2.11170 5.52986
C -5.08228 -3.63609 -0.12843
H -6.14967 -3.80901 -0.34598
H -4.99040 -3.25906 0.90279
H -4.56909 -4.61042 -0.18351
C 2.20178 3.94444 3.50516
H 2.84590 4.50690 2.80821
H 2.83753 3.55954 4.32248
H 1.48902 4.66229 3.94972
C -4.65573 3.28684 -0.68026
H -5.69834 3.63559 -0.75179
H -4.28706 3.05432 -1.69246
H -4.04934 4.11663 -0.28440
C 6.22520 -2.12163 0.87018
H 6.91289 -1.36807 1.29382
H 6.44532 -2.21796 -0.20626
H 6.46195 -3.09069 1.34474
C 0.25936 4.14872 -3.21413
H -0.71632 4.66502 -3.26146
H 0.49047 3.79465 -4.23436
H 1.01853 4.89818 -2.93333
C -4.48317 -3.14199 -2.58013
H -3.88694 -4.06428 -2.67399
H -4.05314 -2.38896 -3.26030
H -5.50743 -3.37541 -2.91272
C -1.02161 4.25659 -0.45112
H -1.65264 4.64598 -1.25891
C -7.36582 1.52576 -1.02025
H -7.54068 2.08689 -0.08762
H -8.30167 0.99981 -1.26524
H -7.18325 2.26132 -1.82317
C -1.27183 4.64840 0.87043
H -2.08350 5.34869 1.10024

IV_{Ph}
SCF (BP86) Energy = -2339.44954105
Enthalpy 0K = -2338.339016
Enthalpy 298K = -2338.266138
Free Energy 298K = -2338.447969
Lowest Frequency = 7.5166 cm^{-1}
Second Frequency = 13.3122 cm^{-1}
SCF (BP86-D3BJ) Energy = -2339.81743728
SCF (C6H6) Energy = -2339.45671607
SCF (BS2) Energy = -3151.64900747

Cu -1.80162 -0.23659 0.13004
Si 4.32896 0.56045 -1.38688
Al 1.78973 0.50547 0.70511
Si 2.53284 3.61591 0.45892
N -4.71789 0.05137 0.62417
N 3.16719 -0.29845 -0.33905
N -4.29064 -0.53707 -1.42690
N 1.33856 2.29869 0.25016
C -3.68436 -0.16668 -0.25222
H 2.07066 0.36010 2.29110
C 2.18727 -2.23506 -2.39322
H 2.09157 -1.13677 -2.35576
C 2.85130 -2.66481 -1.08354
C -0.04046 2.65563 0.15895
C 3.99120 -2.21024 1.05502
C 3.98874 3.45786 -0.76931
H 3.59391 3.53082 -1.80060
H 4.57872 4.38651 -0.61672
C -0.86348 -3.08769 1.14294
C 4.91148 2.22305 -0.62629
H 5.18951 2.05578 0.43205
H 5.87242 2.41953 -1.14698
C 3.33738 -1.71145 -0.12406
C 3.30625 3.61913 2.20789
H 2.59816 3.98456 2.96968
H 4.19729 4.27174 2.23339
H 3.61883 2.60460 2.50839
C 3.64023 -4.52394 0.30676
H 3.76217 -5.60064 0.46950
C -3.48265 -0.92607 -2.61490
H -2.45712 -0.64069 -2.31812
C -0.84121 2.87836 1.33248
C 5.89969 -0.49886 -1.62447
H 6.55404 -0.44887 -0.73841
H 6.47579 -0.12085 -2.48730
H 5.66628 -1.56029 -1.80693
C -4.45434 0.42254 2.04159
H -3.38000 0.67807 2.03270
C 4.11729 -3.60217 1.24473
H 4.62315 -3.96712 2.14681
C -2.20054 -3.55470 1.11637
H -2.99397 -2.87119 0.79615
C -0.26554 2.72934 2.74290
H 0.78603 2.41635 2.63060
C -5.95607 -0.18959 0.01390
C -0.50716 -1.74131 0.77629
C 0.77012 -2.82479 -2.56713
H 0.78912 -3.92848 -2.61343
H 0.32027 -2.46331 -3.51039
H 0.11452 -2.53097 -1.73230
C -5.68493 -0.56151 -1.29001
C 3.06124 -2.61191 -3.61264
H 4.07868 -2.19492 -3.53295
H 2.61024 -2.23967 -4.55073
H 3.16101 -3.70868 -3.70384
C 3.01646 -4.04364 -0.85014
H 2.64724 -4.75662 -1.59730
C 4.61517 -1.29399 2.11161
H 4.39273 -0.25585 1.81160
C 0.17081 -3.97241 1.54539
H 1.20754 -3.62339 1.55158
C 0.10660 -0.65704 0.53645
C -0.65595 2.83529 -1.12777
C -0.14204 -5.28509 1.91918
H 0.66533 -5.95791 2.22579
C -4.65803 -0.77447 2.98478
H -4.34707 -0.49238 4.00433
H -4.04668 -1.63496 2.66920
H -5.71351 -1.09222 3.03344
C 0.14797 2.62851 -2.40898
H 1.19969 2.53337 -2.08905
C -1.47140 -5.74082 1.89633
H -1.70587 -6.76922 2.18998
C -2.18135 3.29432 1.19127
H -2.76692 3.50126 2.09567
C 3.68534 0.98945 -3.13925
H 3.58209 0.09553 -3.77463
H 4.40302 1.66964 -3.63374
H 2.70919 1.49944 -3.10982
C 4.00924 -1.52008 3.51433
H 4.20092 -2.54619 3.87796
H 2.92150 -1.34777 3.50492
H 4.45785 -0.82179 4.24369
C -6.65881 -0.89050 -2.38050
H -6.74341 -0.07964 -3.12528
H -7.66096 -1.04390 -1.95101
H -6.39003 -1.81293 -2.92043
C -0.24284 1.31058 -3.11300
H -0.11118 0.44747 -2.43700
H 0.38107 1.13834 -4.00835
H -1.29881 1.34099 -3.43937
C 1.70753 5.30439 0.13777
H 1.39277 5.40549 -0.91448
H 2.42375 6.11592 0.35520
H 0.81598 5.45928 0.76710
C -0.97835 1.62487 3.55375
H -2.04387 1.87193 3.72328
H -0.50589 1.50701 4.54496
H -0.91873 0.65225 3.03634
C -2.49820 -4.87148 1.49113
H -3.53616 -5.22047 1.46711

C -3.50280 -2.44715 -2.83739
H -4.49760 -2.81334 -3.14229
H -3.19628 -2.98047 -1.92338
H -2.79105 -2.70809 -3.63771
C -0.29447 4.06476 3.52097
H 0.23940 4.86391 2.98018
H 0.18154 3.94641 4.51064
H -1.32955 4.41361 3.69068
C -5.22724 1.67515 2.48171
H -6.29552 1.47539 2.66101
H -5.13234 2.47964 1.73574
H -4.79903 2.03911 3.43029
C 6.15180 -1.46373 2.17104
H 6.59290 -0.74528 2.88502
H 6.62096 -1.30194 1.18701
H 6.43052 -2.47899 2.50676
C 0.04288 3.81609 -3.38928
H -0.97828 3.92745 -3.79730
H 0.72264 3.66712 -4.24671
H 0.31171 4.76879 -2.90283
C -3.85048 -0.11928 -3.86944
H -3.07758 -0.28322 -4.63800
H -3.88749 0.95974 -3.64904
H -4.81557 -0.42640 -4.30284
C -2.00402 3.23532 -1.21335
H -2.45197 3.38872 -2.20280
C -7.29323 -0.03346 0.67226
H -7.33311 -0.52151 1.65978
H -8.07211 -0.49733 0.04735
H -7.57244 1.02565 0.81108
C -2.76958 3.47899 -0.06617
H -3.80641 3.82371 -0.15324

TS(IV-V)$_{Ph}$
SCF (BP86) Energy = -2339.44491055
Enthalpy 0K = -2338.334570
Enthalpy 298K = -2338.262485
Free Energy 298K = -2338.441922
Lowest Frequency = -7.0331 cm^{-1}
Second Frequency = 13.2723 cm^{-1}
SCF (BP86-D3BJ) Energy = -2339.80717069
SCF (C6H6) Energy = -2339.45256520
SCF (BS2) Energy = -3151.64547846

Cu 1.92998 0.14526 -0.06012
Si -4.75178 -0.25733 -0.08422
Al -1.55890 -0.45898 0.68557
Si -2.63970 -3.50093 0.95009
N 4.40252 0.76476 1.35628
N -3.16280 0.48987 0.25018
N 4.85923 -0.38897 -0.43236
N -1.52073 -2.30927 0.22295
C 3.81487 0.11321 0.29990
H -1.11248 -0.27667 2.23258
C -3.19312 2.15678 -2.25135
H -3.19115 1.05957 -2.14489
C -3.06501 2.74254 -0.84272
C -0.32654 -2.79812 -0.39371
C -3.03012 2.58172 1.61712
C -4.45636 -3.15360 0.48144
H -4.57328 -3.25119 -0.61393
H -5.01605 -4.00949 0.91457
C 1.04390 2.84943 -1.14849
C -5.06224 -1.82029 0.98136
H -4.77510 -1.61681 2.03085
H -6.16970 -1.89860 0.99734
C -3.08538 1.92365 0.33946
C -2.61227 -3.49283 2.86757
H -1.75268 -4.04645 3.27940
H -3.53204 -3.96331 3.25914
H -2.56529 -2.46296 3.26137
C -2.89066 4.77516 0.52090
H -2.82255 5.86670 0.59075
C 4.62016 -1.11977 -1.70687
H 3.53337 -1.31871 -1.68470
C 0.80951 -3.20915 0.38582
C -6.14726 0.97121 0.35357
H -6.31139 1.01066 1.44358
H -7.09256 0.64575 -0.11525
H -5.92991 1.99575 0.01145
C 3.57913 1.49592 2.36000
H 2.54658 1.21184 2.08379
C -2.91935 3.98616 1.67570
H -2.88285 4.47143 2.65877
C 1.98208 2.96982 -2.20465
H 2.32836 2.06353 -2.71120
C 0.80635 -3.13771 1.91454
H -0.18569 -2.76512 2.21516
C 5.79893 0.68353 1.28386
C 0.56117 1.56190 -0.71561
C -2.01598 2.53467 -3.17622
H -1.93585 3.62806 -3.31227
H -2.15677 2.08640 -4.17611
H -1.05733 2.17363 -2.77440
C 6.09007 -0.04562 0.14561
C -4.52426 2.58970 -2.91140
H -5.39725 2.33437 -2.28854
H -4.65019 2.10410 -3.89624
H -4.54551 3.68228 -3.07570
C -2.97361 4.14298 -0.72483
H -2.96816 4.75130 -1.63730
C -3.15457 1.82976 2.94558
H -3.20136 0.75382 2.70786
C 0.56785 4.02242 -0.50794
H -0.17937 3.93477 0.28588
C -0.04246 0.53288 -0.28941
C -0.24304 -2.89846 -1.82274
C 1.03433 5.27862 -0.91699
H 0.65350 6.17826 -0.42269
C 3.70161 3.01906 2.19196
H 2.97976 3.51584 2.86103
H 3.47020 3.31892 1.15769
H 4.70646 3.39171 2.45217
C -1.40302 -2.46647 -2.71599
H -2.19721 -2.11636 -2.03463
C 1.97415 5.38922 -1.95612
H 2.33372 6.37496 -2.26917
C 1.94939 -3.72826 -0.26274
H 2.79713 -4.06736 0.34635
C -5.04694 -0.82745 -1.89059
H -5.23844 0.01362 -2.57460
H -5.93484 -1.48588 -1.91642
H -4.19606 -1.40338 -2.29050
C -1.93391 2.05409 3.86449
H -1.82571 3.11843 4.14334
H -1.00724 1.72250 3.36936
H -2.04443 1.47565 4.79945
C 7.43793 -0.43416 -0.38198
H 7.66531 -1.50137 -0.21351
H 8.21888 0.15039 0.12865
H 7.53767 -0.23764 -1.46200
C -1.00766 -1.28847 -3.63327
H -0.64287 -0.43272 -3.04166
H -1.87442 -0.95030 -4.22867
H -0.21035 -1.57946 -4.34219
C -2.22500 -5.25414 0.32560
H -2.43566 -5.35343 -0.75261
H -2.84364 -5.99597 0.86033
H -1.16620 -5.51697 0.48055
C 1.83766 -2.12387 2.45109
H 2.86910 -2.39438 2.16025
H 1.79242 -2.06777 3.55403
H 1.62300 -1.11221 2.05602
C 2.44336 4.23130 -2.60057
H 3.16503 4.31262 -3.42018
C 4.92946 -0.23048 -2.92262
H 6.00729 -0.01575 -3.01942
H 4.39092 0.72813 -2.85163
H 4.60617 -0.74190 -3.84438
C 1.03105 -4.51901 2.57019
H 0.28631 -5.25725 2.23004
H 0.95633 -4.44287 3.66980
H 2.03190 -4.92411 2.33458
C 3.83064 1.01181 3.79601

H 4.80309 1.34656 4.19131
H 3.77919 -0.08690 3.85752
H 3.04769 1.42506 4.45270
C -4.45397 2.21742 3.69050
H -4.56785 1.61551 4.61001
H -5.34587 2.05801 3.06298
H -4.44511 3.28169 3.98801
C -1.96563 -3.63981 -3.54858
H -1.21503 -4.02963 -4.26008
H -2.84188 -3.31348 -4.13668
H -2.28037 -4.47865 -2.90520
C 5.33614 -2.47741 -1.75620
H 4.94605 -3.04923 -2.61333
H 5.13878 -3.06197 -0.84373
H 6.42462 -2.38054 -1.89231
C 0.91799 -3.42080 -2.42206
H 0.95543 -3.51341 -3.51458
C 6.75375 1.25087 2.28986
H 6.52610 2.29930 2.54127
H 7.77821 1.22634 1.88728
H 6.75915 0.67480 3.23187
C 2.01046 -3.84891 -1.65680
H 2.88802 -4.29321 -2.13930

V_{Ph}
SCF (BP86) Energy = -2339.47302367
Enthalpy 0K = -2338.361628
Enthalpy 298K = -2338.289057
Free Energy 298K = -2338.469842
Lowest Frequency = 9.7888 cm^{-1}
Second Frequency = 14.1242 cm^{-1}
SCF (BP86-D3BJ) Energy = -2339.84146110
SCF (C6H6) Energy = -2339.47781242
SCF (BS2) Energy = -3151.67375291

Cu 1.32102 -0.00859 0.10103
Si -3.87271 1.55819 -1.20501
Al -1.30896 -0.06841 -0.08488
Si -3.94192 -1.53886 1.26422
N 3.56408 1.55167 1.24075
N -2.22747 1.51466 -0.49126
N 4.05567 -0.56847 1.21201
N -2.29769 -1.52891 0.55650
C 3.06180 0.32385 0.88915
H -0.02875 0.38549 1.04675
C -0.65694 2.70530 -2.76578
H -1.07281 1.68695 -2.67689
C -0.82089 3.37875 -1.40034
C -1.51793 -2.74325 0.58628
C -1.61882 3.46801 0.93100
C -5.23534 -0.74894 0.09859
H -5.17661 -1.25679 -0.88327
H -6.21099 -1.06158 0.52727
C 2.27835 -1.00033 -2.85299
C -5.21376 0.78593 -0.07735
H -5.17162 1.29175 0.90654
H -6.16928 1.12256 -0.53279
C -1.54816 2.77339 -0.32162
C -4.06751 -0.62114 2.93771
H -3.55531 -1.17046 3.74424
H -5.13030 -0.52059 3.22395
H -3.63747 0.39269 2.89603
C -0.32042 5.32964 0.00611
H 0.12820 6.32269 0.12231
C 3.89586 -2.02701 0.95993
H 2.84084 -2.10950 0.64267
C -0.69976 -3.06298 1.71992
C -4.42793 3.36460 -1.45774
H -4.66257 3.83982 -0.49016
H -5.34557 3.38435 -2.07155
H -3.66901 3.98870 -1.95350
C 2.76649 2.78978 1.02622
H 1.79040 2.40098 0.68117
C -1.00173 4.72612 1.07036
H -1.07912 5.25205 2.02989
C 2.08330 -2.01612 -3.82418
H 1.15225 -2.58971 -3.81086
C -0.74212 -2.23776 3.00840
H -1.33951 -1.33580 2.79362
C 4.85752 1.43762 1.77158
C 1.23833 -0.71868 -1.89081
C 0.82981 2.57245 -3.16313
H 1.29636 3.55992 -3.33192
H 0.92891 1.99477 -4.09863
H 1.40419 2.04879 -2.38230
C 5.16958 0.09201 1.75402
C -1.42224 3.44900 -3.88552
H -2.50436 3.50151 -3.68574
H -1.28121 2.93758 -4.85463
H -1.05334 4.48451 -3.99887
C -0.23462 4.64792 -1.21356
H 0.29431 5.11464 -2.05332
C -2.36418 2.88512 2.12988
H -2.81759 1.94170 1.78124
C 3.48886 -0.26410 -2.87523
H 3.64349 0.51388 -2.12131
C 0.05940 -0.62596 -1.42301
C -1.53234 -3.64275 -0.53033
C 4.46651 -0.52768 -3.84422
H 5.39254 0.05724 -3.85213
C 3.34780 3.66271 -0.09690
H 2.62107 4.45460 -0.34006
H 3.51907 3.06838 -1.00889
H 4.29673 4.14572 0.19162
C -2.47271 -3.44708 -1.72231
H -3.08480 -2.55574 -1.49853
C 4.26279 -1.53664 -4.80085
H 5.02878 -1.74289 -5.55553
C 0.11805 -4.21064 1.68231
H 0.73451 -4.45269 2.55679
C -3.91241 0.61447 -2.86376
H -3.31633 1.12688 -3.63649
H -4.94781 0.52141 -3.23736
H -3.50549 -0.40548 -2.75208
C -1.40211 2.54942 3.29178
H -0.91204 3.46033 3.68117
H -0.61466 1.85093 2.96242
H -1.94957 2.08132 4.12955
C 6.41983 -0.57362 2.24376
H 6.26354 -1.11487 3.19352
H 7.19784 0.18425 2.42536
H 6.82788 -1.29211 1.51424
C -1.71767 -3.18002 -3.04330
H -1.08919 -2.27898 -2.96338
H -2.42959 -3.03613 -3.87590
H -1.06732 -4.03468 -3.30668
C -4.50839 -3.33591 1.55900
H -4.85006 -3.80611 0.62234
H -5.35656 -3.34392 2.26572
H -3.70854 -3.96727 1.97806
C 0.64853 -1.77151 3.48528
H 1.28908 -2.62835 3.76228
H 0.55207 -1.13206 4.38112
H 1.16297 -1.18694 2.70352
C 3.06936 -2.27892 -4.78339
H 2.90292 -3.06728 -5.52523
C 4.78150 -2.51321 -0.19900
H 5.85328 -2.49960 0.06246
H 4.62824 -1.90047 -1.10048
H 4.51439 -3.55457 -0.44449
C -1.44236 -3.02662 4.14118
H -2.45755 -3.34559 3.85330
H -1.52279 -2.41125 5.05560
H -0.87080 -3.93649 4.39914
C 2.52100 3.56456 2.32938
H 3.42353 4.08595 2.68830
H 2.15678 2.89385 3.12409
H 1.74278 4.32166 2.14127
C -3.50485 3.80529 2.61845
H -4.06516 3.32447 3.43999
H -4.21715 4.03354 1.80777
H -3.11707 4.76663 3.00069
C -3.42095 -4.65577 -1.90264
H -2.86332 -5.56197 -2.20033

H -4.16288 -4.45040 -2.69475
H -3.96476 -4.89432 -0.97426
C   4.07650 -2.86778 2.23427
H   3.69018 -3.88295 2.04663
H   3.51167 -2.44072 3.07722
H   5.13472 -2.96514 2.52614
C -0.68972 -4.77083 -0.52016
H -0.69866 -5.44846 -1.38242
C   5.68671 2.57610 2.28324
H   5.73506 3.41440 1.56936
H   6.71925 2.23642 2.45949
H   5.30609 2.97544 3.23990
C   0.14395 -5.05646 0.56738
H   0.78716 -5.94350 0.55615

VI$_{Ph}$

SCF (BP86) Energy = -2647.83787465
Enthalpy 0K = -2646.620103
Enthalpy 298K = -2646.538890
Free Energy 298K = -2646.740068
Lowest Frequency = 11.5071 cm^{-1}
Second Frequency = 13.6260 cm^{-1}
SCF (BP86-D3BJ) Energy = -2648.25642866
SCF (C6H6) Energy = -2647.84444456
SCF (BS2) Energy = -3460.11250129

Cu 1.90691 0.18164 -0.09730
Si -3.95446 -0.84191 2.17014
Al -1.76749 0.16961 -0.10113
Si -4.00014 2.60825 0.19931
N   4.67126 -1.01753 -0.03956
N -2.91539 -1.10326 0.74538
N   4.74299 1.12254 -0.41724
N -2.32995 2.00979 -0.03402
C   3.87212 0.08942 -0.17650
H -1.52358 -0.34811 -1.60927
C -1.03861 -3.19919 1.79461
H -1.10496 -2.12690 2.04470
C -2.02391 -3.44556 0.65182
C -1.33763 2.90573 -0.55332
C -3.75722 -2.74393 -0.94458
C -4.71617 2.03012 1.87776
H -3.97501 2.23670 2.67322
H -5.55637 2.73001 2.07129
C   1.62889 -0.17489 3.01083
C -5.22835 0.57288 1.96212
H -5.87120 0.33650 1.09267
H -5.88943 0.46080 2.84794
C -2.89699 -2.41981 0.15734
C -5.27304 2.06720 -1.12668
H -5.12353 2.60343 -2.07757
H -6.28606 2.31869 -0.76079
H -5.25598 0.98796 -1.34085
C -2.89393 -5.03806 -1.00012
H -2.90410 -6.04501 -1.43255
C   4.23929 2.50359 -0.64447
H   3.15847 2.41188 -0.43323
C -1.23780 3.18262 -1.96138
C -4.98522 -2.40465 2.53859
H -5.75828 -2.56348 1.76809
H -5.49716 -2.29319 3.51060
H -4.37028 -3.31770 2.57632
C   4.07952 -2.35428 0.24321
H   3.00646 -2.12244 0.37171
C -3.73461 -4.03724 -1.50011
H -4.40308 -4.26395 -2.33968
C   2.99003 0.14858 3.23258
H   3.58111 0.53060 2.39527
C -2.23170 2.61938 -2.97952
H -2.89024 1.92407 -2.43402
C   6.02621 -0.68847 -0.20133
C   0.98745 -0.00155 1.72338
C   0.41504 -3.49306 1.35961
H   0.56382 -4.56754 1.14746
H   1.12126 -3.20620 2.15941
H   0.67319 -2.92768 0.44881
C   6.07196 0.67132 -0.44278
C -1.38208 -4.01915 3.05904
H -2.39158 -3.78689 3.43622
H -0.65889 -3.80900 3.86814
H -1.34638 -5.10444 2.85406
C -2.04783 -4.72952 0.07024
H -1.38414 -5.50535 0.47154
C -4.71492 -1.71915 -1.54496
H -4.58844 -0.80022 -0.94872
C   0.86543 -0.66273 4.10301
H -0.18972 -0.90038 3.94845
C   0.06780 0.11031 0.84885
C -0.39844 3.54276 0.33371
C   1.45050 -0.82416 5.36543
H   0.84216 -1.20045 6.19469
C   4.58916 -2.95306 1.56368
H   3.97119 -3.83001 1.81732
H   4.49962 -2.22733 2.38770
H   5.63476 -3.29519 1.49545
C -0.47207 3.35612 1.85129
H -0.96455 2.38359 2.02374
C   2.80279 -0.50334 5.57161
H   3.25542 -0.62890 6.56063
C -0.22627 4.04422 -2.43694
H -0.17057 4.25244 -3.51225
C -2.92985 -0.41078 3.72731
H -2.37709 -1.29393 4.09076
H -3.58530 -0.06563 4.54684
H -2.20141 0.39204 3.52125
C -4.35621 -1.39081 -3.01135
H -4.46156 -2.28042 -3.65882
H -3.31747 -1.03040 -3.08785
H -5.02648 -0.60805 -3.41025
C   7.27695 1.53576 -0.65873
H   7.16892 2.19605 -1.53462
H   8.16226 0.90472 -0.83345
H   7.49910 2.17410 0.21467
C   0.90976 3.33511 2.53656
H   1.60333 2.64054 2.03590
H   0.80673 3.01176 3.58651
H   1.37545 4.33775 2.55383
C -4.05043 4.52771 0.16023
H -4.16993 4.96142 1.16723
H -4.91718 4.85557 -0.43993
H -3.14477 4.96654 -0.28881
C -1.55433 1.81582 -4.11013
H -0.85995 2.44068 -4.70143
H -2.31730 1.42436 -4.80608
H -0.99364 0.96067 -3.70256
C   3.56742 -0.01384 4.49941
H   4.61998 0.24924 4.65159
C   4.82148 3.51649 0.35369
H   5.88087 3.74606 0.15574
H   4.72452 3.15365 1.38973
H   4.25775 4.46013 0.27191
C -3.09852 3.75213 -3.57930
H -3.60689 4.33910 -2.79652
H -3.86859 3.33744 -4.25439
H -2.48202 4.45357 -4.17048
C   4.21521 -3.31300 -0.95002
H   5.25754 -3.63367 -1.11488
H   3.83575 -2.85245 -1.87575
H   3.61860 -4.21877 -0.75265
C -6.19167 -2.15946 -1.43320
H -6.86208 -1.36843 -1.81458
H -6.47547 -2.37241 -0.38869
H -6.38768 -3.07289 -2.02330
C -1.34072 4.44908 2.51747
H -0.92024 5.45310 2.32590
H -1.37573 4.29962 3.61175
H -2.37223 4.43872 2.13892
C   4.39266 2.93442 -2.11192
H   3.84221 3.87633 -2.27078
H   3.96658 2.17334 -2.78455
H   5.44495 3.10871 -2.39272
C   0.59198 4.39491 -0.19538
H   1.28883 4.88852 0.49130
C   7.17157 -1.64871 -0.09299

H 7.35044 -1.97387 0.94701
H 8.09649 -1.16730 -0.44686
H 7.01996 -2.55400 -0.70291
C 0.69185 4.64921 -1.57038
H 1.45620 5.33307 -1.95737
C 1.41048 -1.52903 -3.22376
C 0.37892 -2.44337 -2.87744
H -0.40629 -2.11655 -2.18696
C 1.40069 -0.22103 -2.66100
C 2.41510 -1.92213 -4.14783
H 3.19982 -1.21014 -4.42199
C 1.35939 0.87636 -2.08164
C 2.38451 -3.20515 -4.70749
H 3.15610 -3.50259 -5.42525
C 1.36663 -4.10905 -4.35147
H 1.35030 -5.11168 -4.79171
C 0.36819 -3.72568 -3.43846
H -0.42997 -4.42072 -3.15922
H 1.08864 1.92461 -1.98933

TS(E)$_{Ph}$
SCF (BP86) Energy = -2647.81661593
Enthalpy 0K = -2646.600107
Enthalpy 298K = -2646.519573
Free Energy 298K = -2646.719110
Lowest Frequency = -411.4563 cm^{-1}
Second Frequency = 8.7198 cm^{-1}
SCF (BP86-D3BJ) Energy = -2648.23487911
SCF (C6H6) Energy = -2647.82316581
SCF (BS2) Energy = -3460.09183382

Cu 1.98684 -0.13358 -0.16803
Si -3.68951 0.13363 2.46758
Al -1.48477 0.29816 0.05296
Si -3.44817 2.95142 -0.27022
N 4.60337 -1.59105 -0.39835
N -2.81840 -0.59169 1.08214
N 4.85720 0.54384 -0.72416
N -1.85587 2.11861 -0.38251
C 3.91711 -0.40327 -0.41049
H -1.09580 -0.70167 -1.22939
C -1.05435 -2.67383 2.34580
H -0.95983 -1.57590 2.36915
C -2.24668 -3.00688 1.44886
C -0.77629 2.87855 -0.96864
C -4.15584 -2.41711 0.01400
C -4.20008 2.88511 1.49202
H -3.43089 3.22729 2.21061
H -4.96223 3.69295 1.47832
C 2.02565 0.06189 3.02991
C -4.85215 1.57054 1.97552
H -5.57458 1.19646 1.22516
H -5.45583 1.76582 2.88709
C -3.06362 -1.99439 0.84375
C -4.77307 2.26964 -1.46577
H -4.55080 2.54333 -2.50947
H -5.75264 2.71149 -1.20564
H -4.87563 1.17635 -1.41919
C -3.60087 -4.77213 0.41275
H -3.81021 -5.83587 0.25501
C 4.48440 1.98368 -0.78217
H 3.38273 1.96070 -0.68719
C -0.61796 2.96157 -2.39395
C -4.77109 -1.17184 3.33779
H -5.59291 -1.52110 2.69172
H -5.21745 -0.73172 4.24687
H -4.19039 -2.05936 3.63597
C 3.88627 -2.87036 -0.14588
H 2.87570 -2.52875 0.14367
C -4.39873 -3.79067 -0.18502
H -5.24265 -4.09232 -0.81745
C 3.41430 -0.21071 3.09308
H 3.95750 -0.37288 2.15767
C -1.60202 2.31396 -3.37251
H -2.27591 1.67344 -2.77839
C 5.96108 -1.39510 -0.70140
C 1.30465 0.12643 1.77808
C 0.26005 -3.24445 1.76638
H 0.24716 -4.34958 1.75052
H 1.12142 -2.92383 2.37898
H 0.41893 -2.89129 0.73355
C 6.12323 -0.03764 -0.90318
C -1.25357 -3.16821 3.79679
H -2.15778 -2.73344 4.25493
H -0.38732 -2.88775 4.42242
H -1.35641 -4.26757 3.83952
C -2.53375 -4.36770 1.22044
H -1.90240 -5.12809 1.69578
C -5.07638 -1.41727 -0.68151
H -4.76690 -0.42045 -0.32728
C 1.32553 0.28276 4.24534
H 0.25572 0.50404 4.20948
C 0.34296 0.24368 0.95279
C 0.14147 3.60726 -0.13597
C 1.99637 0.22409 5.47320
H 1.43754 0.39717 6.39911
C 4.47216 -3.65566 1.03781
H 3.77871 -4.47088 1.30161
H 4.58601 -3.01029 1.92373
H 5.44592 -4.11446 0.80195
C 0.01136 3.64562 1.38942
H -0.59571 2.77038 1.67879
C 3.37370 -0.05122 5.52255
H 3.89359 -0.09487 6.48516
C 0.43011 3.73190 -2.93612
H 0.52972 3.79715 -4.02618
C -2.45451 0.82521 3.75119
H -1.91090 -0.00201 4.23841
H -2.97613 1.40053 4.53657
H -1.71455 1.49425 3.27936
C -4.89651 -1.45744 -2.21626
H -5.16846 -2.44865 -2.62193
H -3.85245 -1.25324 -2.50558
H -5.54221 -0.70761 -2.70743
C 7.37263 0.70818 -1.26150
H 7.33854 1.11587 -2.28680
H 8.24022 0.03296 -1.20469
H 7.57043 1.54926 -0.57612
C 1.36855 3.55807 2.11854
H 1.97297 2.71560 1.74850
H 1.21170 3.41359 3.20159
H 1.95677 4.48653 2.00115
C -3.28132 4.81638 -0.70025
H -3.41201 5.44956 0.19360
H -4.07553 5.09745 -1.41389
H -2.31184 5.07324 -1.15463
C -0.91569 1.42537 -4.43220
H -0.25711 2.01686 -5.09436
H -1.67462 0.94344 -5.07369
H -0.31101 0.63626 -3.96223
C 4.07784 -0.26604 4.32638
H 5.15273 -0.47624 4.35318
C 5.04165 2.76250 0.42006
H 6.13969 2.86208 0.38196
H 4.76393 2.27200 1.36670
H 4.61338 3.77809 0.42381
C -2.45138 3.39266 -4.08845
H -2.96998 4.05491 -3.37724
H -3.21019 2.92278 -4.74011
H -1.81349 4.03054 -4.72698
C 3.74714 -3.70636 -1.42809
H 4.71768 -4.08647 -1.78903
H 3.27709 -3.11302 -2.22820
H 3.10154 -4.57726 -1.22751
C -6.56345 -1.61351 -0.31252
H -7.18276 -0.82762 -0.78047
H -6.72440 -1.56843 0.77768
H -6.94655 -2.58798 -0.66495
C -0.73005 4.91710 1.86617
H -0.17966 5.82561 1.56101
H -0.81355 4.92696 2.96807
H -1.74402 4.98500 1.44636
C 4.82198 2.62934 -2.13482
H 4.27722 3.58365 -2.21501

H	4.49973	1.98986	-2.97279
H	5.89760	2.84284	-2.24592
C	1.16728	4.36806	-0.73269
H	1.84367	4.94202	-0.08878
C	6.99794	-2.47546	-0.76341
H	7.22632	-2.89698	0.23114
H	7.93636	-2.06931	-1.17120
H	6.69522	-3.31088	-1.41645
C	1.32301	4.43637	-2.12244
H	2.11109	5.05612	-2.56609
C	-0.24132	-2.30676	-2.94545
C	-1.40478	-2.95229	-2.47176
H	-1.87241	-2.59762	-1.54964
C	0.40741	-1.18454	-2.31111
C	0.37898	-2.79887	-4.13068
H	1.28476	-2.30747	-4.49982
C	1.40061	-0.41440	-2.11054
C	-0.17435	-3.87869	-4.82477
H	0.30588	-4.23085	-5.74413
C	-1.33493	-4.51221	-4.34385
H	-1.75892	-5.36575	-4.88317
C	-1.93862	-4.04744	-3.16563
H	-2.83292	-4.53588	-2.76686
H	2.17958	0.02928	-2.72632

E-INT1$_{Ph}$

SCF (BP86) Energy = -2647.88140373
Enthalpy 0K = -2646.658345
Enthalpy 298K = -2646.577658
Free Energy 298K = -2646.779877
Lowest Frequency = 8.6472 cm^{-1}
Second Frequency = 11.5935 cm^{-1}
SCF (BP86-D3BJ) Energy = -2648.28613415
SCF (C6H6) Energy = -2647.88795625
SCF (BS2) Energy = -3460.15837300

Cu	1.73377	0.26920	-0.13079
Si	-4.26979	-1.55719	1.71151
Al	-1.92403	0.02051	0.11124
Si	-4.51163	0.06003	-1.90720
N	4.35803	1.00523	1.16071
N	-2.55470	-1.32398	1.19421
N	4.48061	1.14334	-1.00451
N	-3.01082	0.75929	-1.17951
C	3.60329	0.89345	0.02021
H	3.13518	-2.47046	0.41275
C	-1.24079	-0.74276	3.83813
H	-1.92765	-0.10861	3.25055
C	-0.93964	-1.98567	2.99804
C	-2.60649	2.06020	-1.68356
C	-1.29285	-3.46399	1.05253
C	-5.79694	-0.47276	-0.59604
H	-6.02668	0.39407	0.05262
H	-6.72079	-0.63928	-1.18903
C	0.99667	3.04064	1.21712
C	-5.49383	-1.72776	0.25238
H	-5.16887	-2.56752	-0.39068
H	-6.42744	-2.08279	0.73747
C	-1.58316	-2.25109	1.75103
C	-4.12414	-1.46427	-2.98263
H	-3.54604	-1.19213	-3.88061
H	-5.06327	-1.94020	-3.31714
H	-3.54513	-2.21999	-2.42685
C	0.30634	-4.08725	2.79663
H	1.04119	-4.79366	3.19738
C	4.04485	1.04020	-2.42117
H	2.95675	0.87878	-2.32843
C	-1.77167	2.17239	-2.83643
C	-4.40574	-3.16652	2.71495
H	-4.23023	-4.04932	2.07812
H	-5.42300	-3.24865	3.13623
H	-3.68432	-3.21010	3.54563
C	3.76141	0.75039	2.49662
H	2.68501	0.65827	2.26802
C	-0.33949	-4.35177	1.58378
H	-0.09936	-5.26917	1.03664
C	1.95106	3.88088	0.59200
H	2.43764	3.52799	-0.32178
C	-1.27116	0.95230	-3.61054
H	-1.64409	0.05569	-3.08637
C	5.69616	1.31025	0.85808
C	0.65565	1.74697	0.65637
C	0.03061	0.08450	4.12669
H	0.75785	-0.49078	4.72725
H	-0.21998	0.99532	4.69988
H	0.52019	0.38589	3.18671
C	5.77320	1.40038	-0.51670
C	-1.94599	-1.10784	5.16554
H	-2.88620	-1.65787	4.99448
H	-2.18403	-0.19766	5.74514
H	-1.30004	-1.74514	5.79538
C	-0.00507	-2.91675	3.49482
H	0.48531	-2.71654	4.45493
C	-1.99201	-3.81240	-0.26230
H	-2.90308	-3.19030	-0.31294
C	0.38463	3.49315	2.41457
H	-0.34709	2.85067	2.91168
C	-0.17608	0.80777	0.36538
C	-3.06709	3.24683	-1.03207
C	0.71715	4.74045	2.96045
H	0.23720	5.06840	3.88897
C	3.93764	1.93294	3.46247
H	3.28931	1.77532	4.34015
H	3.63836	2.87985	2.98569
H	4.97218	2.02684	3.83085
C	-3.98291	3.18064	0.19131
H	-4.44446	2.17727	0.19187
C	1.65447	5.56956	2.32154
H	1.90845	6.54578	2.74754
C	-1.39507	3.45354	-3.28701
H	-0.75302	3.53758	-4.17182
C	-4.85882	-0.08507	2.77225
H	-4.31263	-0.02910	3.72791
H	-5.93488	-0.18241	3.00215
H	-4.71659	0.87494	2.24745
C	-1.11113	-3.44243	-1.47547
H	-0.16406	-4.00781	-1.46391
H	-0.84022	-2.37127	-1.46953
H	-1.63280	-3.66144	-2.42479
C	6.96309	1.75116	-1.35740
H	7.11496	1.04469	-2.18981
H	7.87480	1.72832	-0.74012
H	6.88836	2.76473	-1.79039
C	-3.16503	3.31103	1.49567
H	-2.39821	2.51840	1.56868
H	-3.81621	3.24205	2.38566
H	-2.63056	4.27624	1.53093
C	-5.35350	1.38634	-2.97997
H	-5.72244	2.22601	-2.36792
H	-6.21907	0.93866	-3.49910
H	-4.67548	1.80398	-3.74086
C	0.27079	0.87575	-3.62189
H	0.71218	1.74369	-4.14429
H	0.61075	-0.03826	-4.14057
H	0.66640	0.85362	-2.59185
C	2.26541	5.13446	1.13218
H	2.99623	5.77423	0.62560
C	4.27121	2.34340	-3.20439
H	5.33615	2.52866	-3.41945
H	3.86707	3.21238	-2.65930
H	3.74736	2.27645	-4.17235
C	-1.82093	0.92336	-5.05537
H	-2.92374	0.93692	-5.07526
H	-1.48092	0.01414	-5.58267
H	-1.46914	1.79551	-5.63486
C	4.23282	-0.59151	3.08151
H	5.30184	-0.57818	3.35336
H	4.06319	-1.40940	2.36332
H	3.65880	-0.81335	3.99646
C	-2.43073	-5.28985	-0.34733
H	-3.03992	-5.45377	-1.25354
H	-3.03195	-5.58868	0.52844
H	-1.56697	-5.97437	-0.41068
C	-5.12385	4.21924	0.16292

H -4.74776 5.25211 0.26801
H -5.82105 4.04210 1.00050
H -5.69801 4.16698 -0.77765
C   4.63425 -0.20199 -3.10833
H   4.18910 -0.30691 -4.11216
H   4.39157 -1.10534 -2.52709
H   5.72768 -0.13573 -3.23699
C -2.65340 4.50220 -1.51564
H -2.99624 5.41047 -1.00820
C   6.78466 1.52665 1.86490
H   6.66842 2.47808 2.41384
H   7.76107 1.56015 1.35660
H   6.83418 0.71675 2.61127
C -1.82090 4.61483 -2.63501
H -1.51323 5.60084 -2.99950
C   2.86724 -3.83489 -1.23980
C   3.55730 -4.86198 -0.54789
H   3.89636 -4.66717 0.47718
C   2.63544 -2.53668 -0.56880
C   2.44657 -4.11772 -2.56493
H   1.92313 -3.34145 -3.13417
C   1.89492 -1.47660 -0.99873
C   2.69502 -5.36184 -3.15765
H   2.35819 -5.54850 -4.18402
C   3.37654 -6.36865 -2.44985
H   3.57102 -7.34022 -2.91639
C   3.80765 -6.10826 -1.13865
H   4.34211 -6.88035 -0.57319
H   1.40664 -1.62039 -1.98398

TS(E2)$_{Ph}$
SCF (BP86) Energy = -2647.88540071
Enthalpy 0K = -2646.661614
Enthalpy 298K = -2646.582066
Free Energy 298K = -2646.779031
Lowest Frequency = -4.9996 cm^{-1}
Second Frequency = 12.8080 cm^{-1}
SCF (BP86-D3BJ) Energy = -2648.29573228
SCF (C6H6) Energy = -2647.89078853
SCF (BS2) Energy = -3460.16065729

Cu -1.55492 -0.26268 -0.07474
Si 4.05142 0.75846 2.21371
Al 1.73814 -0.22809 0.19658
Si 4.06622 0.96379 -1.74209
N -4.20887 0.13012 1.21737
N   2.30837 0.61127 1.73860
N -4.46422 -0.86706 -0.69726
N   2.77233 -0.22672 -1.32604
C -3.50726 -0.38585 0.15818
H -3.21289 2.04616 -1.14077
C   1.32141 -1.34619 3.81174
H   1.90617 -1.55408 2.89850
C   0.92088 0.13152 3.77327
C   2.53187 -1.30055 -2.27763
C   0.93133 2.40391 2.80072
C   5.40885 1.12949 -0.38849
H   5.85668 0.13132 -0.22046
H   6.20495 1.71793 -0.89170
C -1.39793 -3.43687 0.42181
C   5.04673 1.79924 0.95710
H   4.51437 2.75409 0.78906
H   5.97649 2.07421 1.49720
C   1.36708 1.03901 2.75860
C   3.31768 2.69240 -2.03366
H   2.67133 2.70471 -2.92627
H   4.11548 3.44197 -2.18068
H   2.70949 3.01912 -1.17259
C -0.30613 1.95092 4.87034
H -0.92759 2.30883 5.69880
C -4.07821 -1.47019 -1.99973
H -2.97872 -1.53740 -1.91756
C   1.67750 -1.10952 -3.40874
C   4.20193 1.58160 3.92377
H   3.82339 2.61421 3.94761
H   5.26959 1.60162 4.20611
H   3.65765 1.01719 4.69725
C -3.49375 0.78622 2.34569
H -2.43079 0.60524 2.10123
C   0.10941 2.82915 3.86334
H -0.20190 3.87906 3.90326
C -2.61135 -3.66943 1.11400
H -3.14344 -2.81425 1.54112
C   0.92258 0.19383 -3.67477
H   1.07289 0.84823 -2.79894
C -5.59350 -0.01563 1.02847
C -0.83725 -2.10792 0.28710
C   0.08896 -2.27761 3.80672
H -0.50893 -2.15891 4.72813
H   0.40464 -3.33424 3.75225
H -0.56099 -2.07125 2.94312
C -5.75641 -0.64733 -0.18866
C   2.21126 -1.67360 5.03427
H   3.12929 -1.06405 5.05812
H   2.50878 -2.73735 5.02224
H   1.66846 -1.49120 5.97895
C   0.09236 0.61147 4.80804
H -0.23020 -0.08324 5.59241
C   1.32406 3.43808 1.74069
H   1.79677 2.88308 0.91020
C -0.71909 -4.54422 -0.15122
H   0.21174 -4.36749 -0.69862
C   0.17100 -1.32298 0.17828
C   3.17560 -2.56663 -2.10102
C -1.24084 -5.83798 -0.02643
H -0.70703 -6.68087 -0.47860
C -3.78995 0.12049 3.69808
H -3.06424 0.49358 4.43895
H -3.67740 -0.97442 3.63571
H -4.80164 0.35109 4.07073
C   4.10268 -2.84549 -0.91969
H   4.26978 -1.87617 -0.41970
C -2.43753 -6.05898 0.67739
H -2.83929 -7.07275 0.77675
C   1.50390 -2.16853 -4.32305
H   0.85643 -2.01313 -5.19362
C   4.89977 -0.94339 2.37082
H   4.43975 -1.54584 3.17075
H   5.96403 -0.79712 2.63021
H   4.86584 -1.53424 1.44235
C   0.09632 4.19094 1.18298
H -0.35315 4.85201 1.94560
H -0.67894 3.49444 0.82842
H   0.39238 4.83165 0.33463
C -7.03175 -1.06608 -0.85489
H -7.07750 -0.75124 -1.91058
H -7.88980 -0.60571 -0.34034
H -7.18256 -2.15999 -0.82771
C   3.43989 -3.79884 0.10109
H   2.47442 -3.40036 0.45976
H   4.09318 -3.95838 0.97741
H   3.24113 -4.78535 -0.35477
C   4.98817 0.37312 -3.29775
H   5.58966 -0.52570 -3.08118
H   5.67664 1.16556 -3.63977
H   4.31017 0.12379 -4.12816
C -0.59592 -0.04765 -3.81851
H -0.82581 -0.65118 -4.71526
H -1.12844 0.91371 -3.90763
H -0.99384 -0.56882 -2.93252
C -3.11704 -4.96961 1.24953
H -4.04954 -5.13337 1.80069
C -4.62465 -2.89529 -2.17948
H -5.71060 -2.91116 -2.36706
H -4.40287 -3.51643 -1.29687
H -4.13411 -3.35785 -3.05206
C   1.45165 0.93091 -4.92752
H   2.52356 1.17575 -4.84669
H   0.89690 1.87332 -5.08155
H   1.32066 0.31345 -5.83434
C -3.70553 2.30761 2.35372
H -4.73813 2.58979 2.62111
H -3.45843 2.73313 1.36858
H -3.02797 2.75617 3.09866

```
C   2.35332  4.46708  2.26600
H   2.57474  5.21824  1.48708
H   3.30560  3.99954  2.56168
H   1.95562  5.00545  3.14501
C   5.48215 -3.38765 -1.35328
H   5.40253 -4.38498 -1.82071
H   6.14885 -3.48781 -0.47851
H   5.97044 -2.71774 -2.08104
C  -4.40520 -0.53447 -3.17492
H  -3.98955 -0.95593 -4.10527
H  -3.95342  0.45729 -3.01570
H  -5.49164 -0.41119 -3.32162
C   2.94940 -3.59854 -3.03216
H   3.44274 -4.56624 -2.88408
C  -6.64766  0.41064  2.00437
H  -6.66472 -0.22320  2.90879
H  -7.64186  0.33703  1.53639
H  -6.51720  1.45465  2.33359
C   2.12594 -3.40795 -4.14623
H   1.97140 -4.21612 -4.86931
C  -2.02300  3.61859 -2.02710
C  -3.18651  4.36325 -2.34238
H  -4.16215  3.97453 -2.02402
C  -2.15989  2.34467 -1.28653
C  -0.77824  4.14275 -2.45801
H   0.13952  3.58462 -2.24342
C  -1.18446  1.53090 -0.78599
C  -0.70424  5.35420 -3.15633
H   0.27214  5.73418 -3.47792
C  -1.87156  6.08125 -3.45265
H  -1.81055  7.02717 -4.00117
C  -3.11571  5.57616 -3.04149
H  -4.03464  6.12915 -3.26699
H  -0.15608  1.91203 -0.95867
```

E$_{Ph}$

```
SCF (BP86) Energy = -2647.91378909
Enthalpy 0K = -2646.689251
Enthalpy 298K = -2646.609315
Free Energy 298K = -2646.805168
Lowest Frequency = 14.4255 cm-1
Second Frequency = 16.8134 cm-1
SCF (BP86-D3BJ) Energy =  -2648.33882086
SCF (C6H6) Energy =  -2647.91910097
SCF (BS2) Energy =  -3460.18425945
```

```
Si  4.14030  0.67395  1.71480
Al  1.47815 -0.12644 -0.01182
Si  4.00190 -1.77296 -1.37448
N   2.29485 -1.64343 -0.83389
N   2.65503  1.12346  0.81725
C   1.44585 -2.77261 -1.11662
C   2.70536  3.42268 -0.15677
C   2.28325  2.51476  0.87128
C   1.24704 -3.80452 -0.13549
C   0.81527 -2.92883 -2.39928
C   5.25804 -1.53233  0.04965
H   4.98522 -2.23035  0.86442
H   6.20950 -1.92924 -0.36420
C  -0.43287  2.04035 -1.00025
H  -0.22571  2.54839 -0.04493
C   0.99174  2.16703  3.10891
H   1.23518  1.12076  2.85774
C   1.49835  3.03822  1.95563
C   3.56727  2.96525 -1.33133
H   3.74412  1.88797 -1.17578
C   0.09590 -0.75221  1.32208
C   1.00752 -1.92525 -3.53938
H   1.52929 -1.05069 -3.11374
C  -1.20488  2.87114 -1.95004
C   0.03355 -4.07346 -2.66065
H  -0.40937 -4.19580 -3.65688
C   4.97419  2.22345  2.45499
H   5.47914  2.81348  1.67193
H   5.74266  1.90932  3.18337
H   4.26980  2.89620  2.96735
C  -1.76852 -1.35982  3.12923
C   0.13518  0.80553 -1.22723
H  -0.05136  0.41240 -2.24044
C  -0.95076 -0.96945  2.00553
C   1.16872  4.40921  1.97941
H   0.58110  4.79751  2.82014
C   1.94228 -3.77281  1.22610
H   2.33246 -2.74851  1.35512
C  -3.18286 -1.40709  3.06672
H  -3.67586 -1.13121  2.13029
C  -1.96184  5.10480 -2.62238
H  -1.99301  6.18477 -2.44160
C  -1.27951  4.26788 -1.72741
H  -0.76374  4.69166 -0.85750
C   2.33740  4.78022 -0.09239
H   2.66918  5.45714 -0.88890
C   1.57522  5.28543  0.96683
H   1.31302  6.34848  1.00990
C   3.75784 -0.54987  3.13218
H   3.17313 -0.06259  3.93028
H   4.69376 -0.92924  3.58013
H   3.17926 -1.41972  2.77862
C   2.85018  3.14614 -2.68771
H   1.88492  2.61551 -2.70466
H   3.47430  2.75619 -3.51182
H   2.65171  4.21253 -2.89793
C   4.44540 -0.52596 -2.75326
H   3.92917 -0.77093 -3.69594
H   5.53285 -0.56244 -2.94805
H   4.18840  0.51160 -2.49098
C  -1.85557  2.33795 -3.08906
H  -1.81926  1.25861 -3.26947
C   0.44689 -4.92131 -0.44492
H   0.31842 -5.70612  0.30945
C   5.48683 -0.11052  0.60364
H   5.69615  0.59827 -0.22081
H   6.40137 -0.09714  1.23399
C  -0.54123  2.26617  3.27574
H  -0.85280  3.28132  3.58172
H  -0.89194  1.56196  4.05006
H  -1.06328  2.01629  2.33655
C   4.36976 -3.51826 -2.08146
H   4.89846 -4.14635 -1.34491
H   5.03201 -3.42309 -2.96009
H   3.46553 -4.06279 -2.39452
C  -1.13324 -1.70566  4.35208
H  -0.04186 -1.66789  4.40912
C   4.93999  3.67483 -1.35680
H   4.82894  4.76218 -1.51861
H   5.56520  3.27925 -2.17717
H   5.48995  3.53490 -0.41133
C  -2.60228  4.56101 -3.74810
H  -3.14050  5.21191 -4.44527
C   1.67465  2.51880  4.45155
H   2.76737  2.38596  4.40688
H   1.28780  1.87639  5.26316
H   1.47795  3.56860  4.73518
C  -0.33353 -1.44886 -4.14209
H  -0.84634 -2.26312 -4.68488
H  -0.16519 -0.63029 -4.86442
H  -1.02382 -1.08515 -3.36211
C  -3.93479 -1.79628  4.18396
H  -5.02744 -1.83011  4.11491
C   0.99245 -4.08530  2.40075
H   0.65721 -5.13824  2.38835
H   0.09968 -3.44298  2.37980
H   1.51284 -3.92664  3.36225
C  -2.54802  3.17301 -3.97513
H  -3.04899  2.74184 -4.84893
C  -0.16109 -5.06927 -1.69708
H  -0.75633 -5.96033 -1.92678
C   1.88590 -2.50733 -4.67297
H   2.87293 -2.82929 -4.30695
H   2.04267 -1.75726 -5.46919
H   1.39970 -3.38671 -5.13297
C  -1.89263 -2.08886  5.46410
H  -1.38468 -2.35166  6.39807
C  -3.29534 -2.13851  5.38678
```

H -3.88499 -2.43980 6.25866
C 3.14269 -4.74793 1.26531
H 3.65643 -4.69355 2.24205
H 3.87896 -4.52442 0.47832
H 2.80453 -5.78995 1.11977
N -4.33324 0.86868 -0.06803
N -4.03366 -1.13858 -0.85048
C -5.60326 0.38612 -0.41980
C -5.41223 -0.88863 -0.91716
C -3.35835 -0.06577 -0.31909
Cu -1.46486 -0.04185 0.16256
C -3.99606 2.17573 0.55701
H -2.89190 2.18372 0.52218
C -6.43942 -1.83346 -1.46330
H -6.40119 -1.90556 -2.56457
H -7.44830 -1.48333 -1.19426
H -6.32762 -2.85263 -1.05881
C -6.88671 1.15104 -0.30294
H -7.00348 1.62866 0.68361
H -7.74028 0.46847 -0.43841
H -6.97534 1.94190 -1.06858
C -3.31367 -2.38698 -1.22689
H -2.24933 -2.09939 -1.14250
C -4.42802 2.22688 2.03226
H -5.52570 2.23255 2.14366
H -4.04274 3.15133 2.49341
H -4.02290 1.36993 2.59347
C -4.50070 3.37227 -0.26349
H -4.22783 3.26970 -1.32544
H -4.02321 4.28982 0.11660
H -5.59089 3.50933 -0.18020
C -3.56675 -3.52511 -0.22563
H -3.36748 -3.19490 0.80588
H -2.87968 -4.35603 -0.45468
H -4.59988 -3.90920 -0.28096
C -3.57500 -2.80274 -2.68234
H -4.57281 -3.24912 -2.82108
H -2.82531 -3.55883 -2.96499
H -3.46992 -1.94697 -3.36860

INT(E-P)$_{Ph}$
SCF (BP86) Energy = -2956.28626824
Enthalpy 0K = -2954.955219
Enthalpy 298K = -2954.866735
Free Energy 298K = -2955.084743
Lowest Frequency = 4.8412 cm^{-1}
Second Frequency = 9.7038 cm^{-1}
SCF (BP86-D3BJ) Energy = -2956.75028442
SCF (C6H6) Energy = -2956.29447745
SCF (BS2) Energy = -3768.63298786

Si -4.29311 -1.84772 1.23971
Al -1.81581 0.18668 0.35916
Si -3.42060 1.60614 2.87634
N -1.90313 1.13158 2.03728
N -3.29542 -0.99542 0.02213
C -0.69155 1.68766 2.58244
C -4.70678 -0.44531 -1.97988
C -3.69562 -1.24400 -1.34602
C 0.12517 0.91992 3.48555
C -0.28881 3.03866 2.29637
C -4.49730 0.12290 3.43138
H -3.86026 -0.55373 4.03285
H -5.20013 0.57989 4.16080
C -1.58148 1.01438 -2.55718
H -1.62944 -0.05854 -2.79914
C -2.01798 -3.22457 -1.54042
H -1.77228 -2.84989 -0.53325
C -3.11491 -2.31718 -2.10144
C -5.39455 0.72053 -1.27040
H -4.91519 0.80700 -0.28154
C -0.07093 -0.94594 0.44976
C -1.10109 3.97675 1.39818
H -1.92365 3.38073 0.96854
C -1.51053 1.87334 -3.75906
C 0.86847 3.56960 2.90206
H 1.14393 4.61044 2.69240
C -5.57325 -2.98773 0.40186
H -6.29628 -2.42663 -0.21113
H -6.13597 -3.53015 1.18242
H -5.10076 -3.73251 -0.25767
C 1.03505 -3.36582 0.79273
C -1.62200 1.39336 -1.24514
H -1.61898 2.48661 -1.08368
C 0.65255 -1.99194 0.53417
C -3.54836 -2.56188 -3.42068
H -3.10133 -3.39377 -3.97872
C -0.26239 -0.49342 3.92391
H -0.96950 -0.87374 3.16678
C 2.17833 -3.96455 0.20820
H 2.81469 -3.35704 -0.44070
C -1.69260 2.06086 -6.20047
H -1.88549 1.58936 -7.17056
C -1.74168 1.29152 -5.02967
H -1.98641 0.22410 -5.08161
C -5.10075 -0.72963 -3.30134
H -5.87509 -0.10830 -3.76700
C -4.53553 -1.78122 -4.02959
H -4.86313 -1.99186 -5.05368
C -3.23526 -2.95514 2.38185
H -2.82322 -3.80597 1.81400
H -3.84980 -3.36144 3.20507
H -2.39881 -2.39619 2.83396
C -5.20064 2.05847 -2.01939
H -4.13398 2.27656 -2.18178
H -5.64417 2.89050 -1.44298
H -5.69564 2.04249 -3.00695
C -4.52981 2.75983 1.82667
H -4.16478 3.79942 1.86017
H -5.56023 2.75528 2.22632
H -4.57916 2.45858 0.76924
C -1.21457 3.25780 -3.70786
H -1.01615 3.72899 -2.73922
C 1.27640 1.49711 4.05591
H 1.87081 0.90749 4.76341
C -5.29046 -0.67595 2.37651
H -5.89062 0.00401 1.74330
H -6.03038 -1.33234 2.88154
C -0.73650 -3.15502 -2.40250
H -0.91927 -3.52912 -3.42616
H 0.06251 -3.77350 -1.95717
H -0.36429 -2.11976 -2.48013
C -3.04841 2.54879 4.50661
H -3.07147 1.87565 5.37995
H -3.83225 3.31104 4.66397
H -2.07286 3.05880 4.50664
C 0.22580 -4.15721 1.64851
H -0.65694 -3.70807 2.10855
C -6.90308 0.46330 -1.04885
H -7.43480 0.35528 -2.01142
H -7.36435 1.30873 -0.50718
H -7.08572 -0.45298 -0.46366
C -1.40081 3.43294 -6.13031
H -1.35897 4.03684 -7.04316
C -2.47446 -4.69560 -1.40770
H -3.35586 -4.79313 -0.75273
H -1.66478 -5.31299 -0.97910
H -2.74183 -5.12556 -2.38992
C -0.26391 4.55415 0.23470
H 0.54197 5.21350 0.60480
H -0.90192 5.16355 -0.43081
H 0.20722 3.75929 -0.36396
C 2.49495 -5.30649 0.46197
H 3.38120 -5.74986 -0.00489
C 0.93637 -1.46056 4.00284
H 1.62380 -1.19596 4.82707
H 1.50811 -1.46982 3.06134
H 0.58662 -2.48930 4.19720
C -1.16138 4.02589 -4.87720
H -0.92843 5.09476 -4.81372
C 1.65609 2.81568 3.77813
H 2.53702 3.25626 4.25954
C -1.71979 5.14690 2.20024
H -2.35353 4.79423 3.02863

H -2.33824 5.78323 1.54174
H -0.93123 5.78750 2.63506
C 0.54841 -5.49766 1.89673
H -0.09285 -6.08959 2.55835
C 1.68210 -6.08090 1.30618
H 1.92995 -7.12905 1.50265
C -0.98751 -0.47953 5.29034
H -1.28270 -1.50259 5.58622
H -1.89367 0.14336 5.26585
H -0.32528 -0.07666 6.07813
N 4.32981 -1.18714 -1.72751
N 4.81207 -1.05748 0.38949
C 5.70208 -1.46277 -1.61592
C 6.00805 -1.38012 -0.27110
C 3.77328 -0.93863 -0.49689
Cu 1.89145 -0.50945 -0.13776
C 3.49196 -1.17487 -2.95807
H 2.51402 -0.82285 -2.58212
C 7.34419 -1.54419 0.38707
H 7.76401 -0.58087 0.72652
H 8.06113 -1.97852 -0.32696
H 7.30399 -2.21634 1.25953
C 6.62628 -1.74119 -2.76221
H 6.22994 -2.50895 -3.44655
H 7.59131 -2.11073 -2.38196
H 6.83613 -0.83719 -3.36094
C 4.58636 -0.89884 1.85265
H 3.53128 -0.57303 1.90369
C 3.29575 -2.58402 -3.54109
H 4.22918 -3.00236 -3.95326
H 2.56203 -2.53686 -4.36254
H 2.90498 -3.27765 -2.78012
C 3.98759 -0.15703 -3.99648
H 4.16717 0.82318 -3.52764
H 3.21427 -0.02800 -4.77136
H 4.91127 -0.48415 -4.50073
C 4.70184 -2.23925 2.59683
H 4.06768 -3.00655 2.12540
H 4.36086 -2.10598 3.63666
H 5.74011 -2.61005 2.63305
C 5.45110 0.21309 2.46570
H 6.51220 -0.07404 2.54476
H 5.08901 0.42194 3.48580
H 5.37573 1.14358 1.88048
C 1.52499 1.42760 -0.97914
C 2.50529 2.17149 -0.79469
H 0.47505 1.27520 -1.28018
C 3.66337 2.95837 -0.54678
C 3.95824 3.40656 0.76992
C 4.51928 3.33947 -1.61514
C 5.08856 4.20014 1.00113
C 5.64273 4.13657 -1.36908
C 5.93342 4.56564 -0.06204
H 3.28064 3.13536 1.58627
H 4.27625 3.01829 -2.63239
H 5.30719 4.54476 2.01719
H 6.29250 4.42949 -2.20017
H 6.81228 5.19097 0.12610

TS(E-P)$_{Ph}$
SCF (BP86) Energy = -2956.27516013
Enthalpy 0K = -2954.947130
Enthalpy 298K = -2954.859858
Free Energy 298K = -2955.073226
Lowest Frequency = -723.4943 cm^{-1}
Second Frequency = 6.9962 cm^{-1}
SCF (BP86-D3BJ) Energy = -2956.74107316
SCF (C6H6) Energy = -2956.28157242
SCF (BS2) Energy = -3768.62122810

Si -4.15821 -2.26331 0.30017
Al -1.58450 -0.24301 0.45632
Si -3.25305 0.06550 3.30785
N -1.72969 0.01131 2.34686
N -3.14648 -0.95272 -0.39713
C -0.51673 0.30069 3.07940
C -4.53903 0.41088 -1.96679
C -3.51889 -0.57465 -1.73851
C 0.31853 -0.76656 3.56003
C -0.13984 1.65144 3.39512
C -4.32253 -1.51528 3.14695
H -3.67998 -2.39070 3.36100
H -5.01143 -1.44376 4.01554
C -1.32604 1.79265 -1.80378
H -1.15319 0.95449 -2.49464
C -1.85632 -2.29953 -2.76339
H -1.63201 -2.42224 -1.69156
C -2.92328 -1.20834 -2.88270
C -5.26447 1.11414 -0.81936
H -4.86358 0.68191 0.11184
C 0.01746 -1.42044 0.04635
C -0.99623 2.86613 3.02234
H -1.81573 2.49978 2.38066
C -1.70718 3.03563 -2.47614
C 1.03379 1.89468 4.13699
H 1.29662 2.93024 4.38483
C -5.44865 -2.87614 -0.96214
H -6.19706 -2.10460 -1.20281
H -5.98149 -3.74374 -0.53394
H -4.99045 -3.19149 -1.91247
C 1.26384 -3.71468 -0.53965
C -1.09931 1.58238 -0.45496
H -1.29007 2.45495 0.19015
C 0.82271 -2.36194 -0.26133
C -3.33923 -0.84302 -4.17926
H -2.88421 -1.34560 -5.04156
C -0.05335 -2.24023 3.37723
H -0.74884 -2.29012 2.52211
C 2.56117 -4.00246 -1.02959
H 3.25358 -3.17113 -1.18861
C -2.40816 4.16481 -4.53772
H -2.68513 4.11245 -5.59605
C -2.05827 2.99626 -3.85032
H -2.07778 2.02778 -4.36155
C -4.91337 0.74177 -3.28303
H -5.69233 1.49782 -3.43725
C -4.32613 0.12522 -4.39274
H -4.64050 0.38788 -5.40890
C -3.09112 -3.76870 0.78923
H -2.72362 -4.27818 -0.11739
H -3.68390 -4.49628 1.37168
H -2.22098 -3.47688 1.40089
C -4.98695 2.63351 -0.80039
H -3.91076 2.84786 -0.71056
H -5.50629 3.11197 0.04934
H -5.34468 3.11833 -1.72607
C -4.37593 1.54836 2.86195
H -4.01783 2.47915 3.33062
H -5.40073 1.36195 3.23176
H -4.43736 1.72164 1.77719
C -1.70455 4.29435 -1.81998
H -1.41013 4.35233 -0.76733
C 1.48213 -0.46458 4.29544
H 2.09747 -1.28850 4.67420
C -5.13647 -1.74077 1.85629
H -5.74122 -0.84644 1.61462
H -5.87316 -2.55686 2.01305
C -0.54264 -1.90099 -3.47385
H -0.69225 -1.77602 -4.56177
H 0.22465 -2.68094 -3.32493
H -0.14005 -0.95521 -3.07334
C -2.88292 0.20007 5.18617
H -2.99115 -0.77654 5.68709
H -3.61579 0.88426 5.64944
H -1.87403 0.57935 5.40953
C 0.37435 -4.79853 -0.31739
H -0.62455 -4.59259 0.07400
C -6.78989 0.86433 -0.84332
H -7.25551 1.28899 -1.75069
H -7.27055 1.34205 0.02905
H -7.03384 -0.21047 -0.81691
C -2.40355 5.40189 -3.87133
H -2.67114 6.31785 -4.40882
C -2.34492 -3.66126 -3.30991

H -3.25098 -4.01107 -2.78833
H -1.56170 -4.42924 -3.18010
H -2.58383 -3.60300 -4.38715
C -0.20905 3.93810 2.23673
H 0.57181 4.40670 2.86180
H -0.88708 4.74789 1.91016
H 0.28963 3.51528 1.35107
C 2.95134 -5.32200 -1.29616
H 3.96025 -5.52159 -1.67382
C 1.15677 -3.14552 3.06873
H 1.81373 -3.26647 3.94928
H 1.75933 -2.74626 2.23799
H 0.81456 -4.15537 2.78374
C -2.04725 5.46081 -2.51091
H -2.03199 6.42442 -1.99064
C 1.85106 0.85369 4.58674
H 2.74738 1.06457 5.18130
C -1.62115 3.52607 4.27569
H -2.22109 2.81747 4.86705
H -2.27252 4.37035 3.98537
H -0.83396 3.92777 4.93893
C 0.77135 -6.11458 -0.58582
H 0.06847 -6.93517 -0.40605
C 2.05946 -6.38492 -1.07841
H 2.36606 -7.41532 -1.28638
C -0.78879 -2.79226 4.62143
H -1.06063 -3.85308 4.47332
H -1.70949 -2.23063 4.83714
H -0.14146 -2.73017 5.51493
N 4.34458 -0.67092 -2.01221
N 4.82231 -0.73599 0.10711
C 5.74624 -0.62024 -1.92037
C 6.04927 -0.66812 -0.57372
C 3.76928 -0.73530 -0.76835
Cu 1.86244 -0.60725 -0.32579
C 3.49316 -0.56460 -3.22550
H 2.48011 -0.76191 -2.82869
C 7.39790 -0.63925 0.07868
H 7.57407 0.29796 0.63457
H 8.18511 -0.72102 -0.68661
H 7.54181 -1.47574 0.78307
C 6.68660 -0.55007 -3.08507
H 6.70335 -1.48566 -3.67160
H 7.71161 -0.36933 -2.72627
H 6.43668 0.27140 -3.77744
C 4.58078 -0.82210 1.57206
H 3.48125 -0.73431 1.64715
C 3.80586 -1.64436 -4.27325
H 4.76152 -1.46718 -4.79235
H 3.01089 -1.64158 -5.03708
H 3.83001 -2.64636 -3.81502
C 3.50152 0.86433 -3.79233
H 3.22472 1.58485 -3.00643
H 2.76462 0.94082 -4.60943
H 4.48618 1.14460 -4.20312
C 4.98718 -2.19402 2.13395
H 4.51282 -3.00760 1.56200
H 4.65202 -2.27226 3.18124
H 6.08010 -2.34431 2.12156
C 5.18922 0.35937 2.34231
H 6.28341 0.27737 2.44927
H 4.75340 0.38085 3.35445
H 4.94674 1.31530 1.85122
C 1.60057 1.35782 -0.17135
C 2.24756 2.42524 -0.03633
H 0.21070 1.26174 -0.29326
C 3.02187 3.60349 0.14830
C 3.46490 3.97712 1.44918
C 3.38010 4.43066 -0.95239
C 4.24224 5.12645 1.63185
C 4.15446 5.57951 -0.75624
C 4.59143 5.93195 0.53323
H 3.17676 3.35084 2.29905
H 3.03382 4.15749 -1.95389
H 4.57518 5.40015 2.63887
H 4.41909 6.20679 -1.61427
H 5.19742 6.83179 0.68142

INT2(E-P)$_{Ph}$
SCF (BP86) Energy = -2956.28760809
Enthalpy 0K = -2954.954559
Enthalpy 298K = -2954.866385
Free Energy 298K = -2955.083338
Lowest Frequency = 7.6525 cm^{-1}
Second Frequency = 12.1926 cm^{-1}
SCF (BP86-D3BJ) Energy = -2956.75240609
SCF (C6H6) Energy = -2956.29424798
SCF (BS2) Energy = -3768.63592102

Si -4.23079 -2.01995 -0.89676
Al -1.60375 -0.55179 0.29860
Si -3.32671 -1.62339 2.88451
N -1.77020 -1.28392 2.02876
N -3.14288 -0.58191 -0.79644
C -0.58250 -1.58324 2.80869
C -4.49864 1.42261 -1.43811
C -3.46734 0.46819 -1.73741
C 0.10573 -2.83193 2.63915
C -0.11059 -0.67529 3.81678
C -4.49115 -2.80878 1.92921
H -3.91392 -3.71883 1.67860
H -5.21401 -3.13334 2.70755
C -0.89377 2.37206 -0.40587
H -0.09665 1.97554 -1.04961
C -1.75651 -0.45924 -3.47282
H -1.54061 -1.12206 -2.61918
C -2.82505 0.53822 -3.01901
C -5.28137 1.40796 -0.12270
H -4.89387 0.56070 0.46560
C 0.12515 -1.03002 -0.55506
C -0.75825 0.68454 4.09952
H -1.56941 0.81625 3.36379
C -1.49478 3.62865 -0.84818
C 0.98953 -1.03767 4.61962
H 1.33143 -0.33881 5.39172
C -5.45461 -1.84036 -2.34555
H -6.12977 -0.97800 -2.23872
H -6.07257 -2.75438 -2.39847
H -4.93401 -1.72721 -3.30958
C 1.31991 -2.75448 -2.18722
C -1.12327 1.68027 0.75858
H -1.84457 2.02830 1.50520
C 0.97573 -1.69487 -1.25207
C -3.21179 1.53658 -3.93587
H -2.72455 1.56913 -4.91779
C -0.32369 -3.87985 1.61061
H -0.95250 -3.35801 0.86846
C 2.65166 -3.01783 -2.58611
H 3.44995 -2.40082 -2.16540
C -1.85449 5.26384 -2.63609
H -1.75549 5.55028 -3.68807
C -1.36690 4.02530 -2.20256
H -0.89569 3.33951 -2.91335
C -4.83639 2.40469 -2.38787
H -5.62268 3.12884 -2.14425
C -4.20556 2.47162 -3.63389
H -4.49116 3.23789 -4.36241
C -3.24812 -3.62166 -1.23051
H -2.88551 -3.63481 -2.27226
H -3.90534 -4.49960 -1.09629
H -2.38219 -3.75300 -0.56156
C -5.06391 2.69620 0.69966
H -3.99857 2.85513 0.92636
H -5.61622 2.64572 1.65489
H -5.41756 3.58824 0.15325
C -4.29727 -0.02721 3.28261
H -3.86222 0.49755 4.14869
H -5.34516 -0.27340 3.53184
H -4.31155 0.67652 2.43651
C -2.09648 4.53170 0.06530
H -2.13847 4.27338 1.12767
C 1.19630 -3.14396 3.47487
H 1.69411 -4.11257 3.35424
C -5.25852 -2.30343 0.68930

H -5.82051 -1.38141 0.92694
H -6.02983 -3.04728 0.39831
C -0.43526 0.23831 -3.86577
H -0.57173 0.90722 -4.73493
H 0.32639 -0.51325 -4.13659
H -0.03230 0.83402 -3.03038
C -3.00677 -2.52049 4.53985
H -2.78635 -3.59035 4.39554
H -3.92104 -2.44873 5.15590
H -2.17260 -2.08936 5.11298
C 0.28781 -3.56624 -2.72677
H -0.74303 -3.38430 -2.41401
C -6.79788 1.19099 -0.33410
H -7.24447 2.01498 -0.91846
H -7.31917 1.15529 0.63918
H -7.01447 0.25071 -0.86585
C -2.46263 6.13937 -1.72138
H -2.83302 7.11376 -2.05702
C -2.24438 -1.33710 -4.64931
H -3.15289 -1.90510 -4.38943
H -1.46165 -2.06143 -4.93619
H -2.47968 -0.72457 -5.53809
C 0.24845 1.84791 3.93986
H 1.01283 1.81828 4.73702
H -0.26866 2.82040 4.02508
H 0.77946 1.81902 2.97496
C 2.94084 -4.04848 -3.49136
H 3.98032 -4.23697 -3.78182
C 0.87000 -4.51435 0.86556
H 1.44888 -5.18863 1.52201
H 1.55338 -3.74730 0.47015
H 0.51157 -5.12024 0.01581
C -2.57688 5.77137 -0.36836
H -3.02508 6.46292 0.35237
C 1.64166 -2.26407 4.46705
H 2.47813 -2.53659 5.12043
C -1.38552 0.75533 5.51283
H -2.15178 -0.01926 5.67483
H -1.85748 1.74124 5.67429
H -0.61448 0.62810 6.29347
C 0.58084 -4.59200 -3.63396
H -0.23286 -5.20586 -4.03513
C 1.90883 -4.83857 -4.02339
H 2.13626 -5.64217 -4.73164
C -1.17339 -5.00300 2.24994
H -1.46698 -5.74931 1.48987
H -2.09164 -4.61405 2.71576
H -0.59663 -5.52772 3.03279
N 4.66589 0.10647 -1.81652
N 4.87791 -0.96801 0.06044
C 6.04748 -0.02132 -1.58697
C 6.18125 -0.70395 -0.39427
C 3.94263 -0.46076 -0.80062
Cu 2.01545 -0.23225 -0.39882
C 3.97451 0.84678 -2.90284
H 2.91247 0.59397 -2.72943
C 7.43867 -1.13922 0.29439
H 7.44847 -0.86104 1.36125
H 8.30921 -0.65674 -0.17703
H 7.59600 -2.23094 0.23349
C 7.12490 0.46452 -2.50796
H 7.19316 -0.13599 -3.43286
H 8.10235 0.40013 -2.00470
H 6.98006 1.51658 -2.80476
C 4.45454 -1.57128 1.35254
H 3.35766 -1.65349 1.24117
C 4.35611 0.34284 -4.30376
H 5.38196 0.62488 -4.59054
H 3.67317 0.79100 -5.04454
H 4.25634 -0.75282 -4.37046
C 4.12642 2.36645 -2.72498
H 3.78945 2.66394 -1.71883
H 3.50481 2.88802 -3.47265
H 5.16796 2.70079 -2.86764
C 5.01272 -2.98724 1.56004
H 4.84076 -3.61721 0.67188
H 4.48905 -3.45061 2.41215
H 6.09018 -2.98867 1.79208
C 4.72384 -0.61812 2.52713
H 5.80197 -0.49270 2.72666
H 4.25168 -1.02559 3.43621
H 4.27494 0.36628 2.31831
C 2.16199 1.43766 0.53242
C 2.48848 2.54717 1.00181
H -0.25918 1.09433 1.12304
C 2.82834 3.82284 1.54876
C 3.45209 3.93437 2.82198
C 2.55477 5.02333 0.83696
C 3.78434 5.18662 3.35312
C 2.89300 6.27125 1.37447
C 3.50947 6.36300 2.63447
H 3.66684 3.02015 3.38400
H 2.06914 4.95590 -0.14161
H 4.26319 5.24550 4.33709
H 2.67149 7.18140 0.80568
H 3.77174 7.34064 3.05235

TS2(E-P)$_{Ph}$

SCF (BP86) Energy = -2956.28351390
Enthalpy 0K = -2954.950760
Enthalpy 298K = -2954.862891
Free Energy 298K = -2955.079964
Lowest Frequency = -28.5763 cm^{-1}
Second Frequency = 9.2527 cm^{-1}
SCF (BP86-D3BJ) Energy = -2956.74211890
SCF (C6H6) Energy = -2956.29012861
SCF (BS2) Energy = -3768.63259723

Si -3.70980 -2.89551 -0.77897
Al -1.42647 -0.94223 0.22934
Si -3.19674 -1.58306 2.87910
N -1.62267 -1.19582 2.05842
N -2.88369 -1.28965 -0.89344
C -0.47728 -1.18320 2.95448
C -4.52991 0.30519 -1.90940
C -3.31637 -0.45609 -1.99863
C 0.31606 -2.36132 3.14950
C -0.17288 -0.01162 3.72232
C -4.05555 -3.13367 2.14377
H -3.30269 -3.94501 2.13067
H -4.77458 -3.42583 2.93798
C -2.01299 2.58696 -1.16077
H -2.13934 2.22338 -2.18889
C -1.29837 -1.22647 -3.45675
H -1.06211 -1.74785 -2.51472
C -2.57184 -0.41280 -3.22317
C -5.41525 0.32862 -0.66107
H -4.92910 -0.31610 0.08789
C 0.41283 -1.19421 -0.40854
C -0.94814 1.30242 3.59090
H -1.68113 1.17036 2.77566
C -2.71319 3.84578 -0.86335
C 0.87629 -0.05376 4.66197
H 1.09232 0.84396 5.25236
C -4.79683 -3.19615 -2.31114
H -5.55781 -2.41728 -2.46668
H -5.31250 -4.16618 -2.19788
H -4.18515 -3.24385 -3.22674
C 1.98186 -3.01304 -1.55181
C -1.20927 1.87497 -0.32755
H -0.97898 2.18740 0.69403
C 1.40797 -1.85318 -0.88878
C -3.03104 0.38725 -4.28955
H -2.45168 0.40955 -5.21990
C 0.08290 -3.66384 2.38140
H -0.61048 -3.42837 1.55518
C 3.37616 -3.25336 -1.58800
H 4.04055 -2.53278 -1.10378
C -4.23665 5.60905 -1.63090
H -4.88444 6.02242 -2.41141
C -3.55586 4.40503 -1.85516
H -3.67870 3.86948 -2.80306
C -4.94906 1.07546 -3.01218
H -5.88049 1.64769 -2.93095

C -4.21190 1.13024 -4.19923
H -4.55834 1.73343 -5.04550
C -2.43096 -4.31314 -0.75107
H -2.01756 -4.46896 -1.76178
H -2.91985 -5.25473 -0.44293
H -1.58624 -4.14342 -0.06345
C -5.53119 1.74704 -0.05991
H -4.54567 2.16213 0.20107
H -6.15166 1.72778 0.85335
H -6.00424 2.44890 -0.76875
C -4.38387 -0.09335 2.85213
H -4.17263 0.58234 3.69718
H -5.43316 -0.42601 2.94220
H -4.29172 0.49405 1.92742
C -2.56499 4.54566 0.36003
H -1.89689 4.15233 1.13236
C   1.35135 -2.34732 4.10590
H   1.93686 -3.26034 4.26342
C -4.79275 -3.06737 0.78696
H -5.54727 -2.25987 0.79487
H -5.37004 -4.00338 0.63381
C -0.08988 -0.33027 -3.80867
H -0.24044 0.19231 -4.77041
H   0.82326 -0.94441 -3.89903
H   0.09170 0.43177 -3.03307
C -2.89708 -2.02783 4.70891
H -2.40991 -3.00692 4.83721
H -3.87787 -2.07023 5.21593
H -2.27627 -1.28309 5.22959
C   1.12354 -3.94940 -2.18459
H   0.04470 -3.77839 -2.15574
C -6.83316 -0.22936 -0.92798
H -7.37117 0.38260 -1.67364
H -7.43039 -0.21622 0.00122
H -6.81405 -1.26626 -1.30061
C -4.08780 6.28367 -0.40823
H -4.61683 7.22579 -0.22990
C -1.49697 -2.29758 -4.55481
H -2.31996 -2.98937 -4.30806
H -0.57608 -2.89389 -4.68178
H -1.73511 -1.83525 -5.52928
C -0.01380 2.47749 3.22379
H   0.68934 2.69891 4.04667
H -0.60139 3.39817 3.05328
H   0.58634 2.27088 2.32249
C   3.89201 -4.38859 -2.22797
H   4.97412 -4.55919 -2.23702
C   1.38562 -4.22513 1.77097
H   2.06118 -4.61784 2.55191
H   1.92794 -3.45407 1.20403
H   1.16122 -5.06004 1.08469
C -3.24778 5.74637 0.58429
H -3.11745 6.27339 1.53537
C   1.63433 -1.21023 4.86922
H   2.42920 -1.22815 5.62324
C -1.72537 1.65421 4.88196
H -2.43317 0.86328 5.17782
H -2.29773 2.58871 4.74350
H -1.03294 1.81251 5.72806
C   1.64449 -5.07693 -2.83147
H   0.96280 -5.78516 -3.31459
C   3.03129 -5.30378 -2.85644
H   3.43673 -6.18726 -3.36051
C -0.57708 -4.75483 3.25690
H -0.71316 -5.68574 2.67776
H -1.56346 -4.44621 3.63683
H   0.05815 -4.99204 4.12906
N   4.83256 0.27053 -1.67983
N   5.00591 -0.23069 0.42839
C   6.19015 0.42252 -1.34607
C   6.29956 0.10485 -0.00662
C   4.09945 -0.11579 -0.59114
Cu 2.13569 -0.08828 -0.36890
C   4.15523 0.58303 -2.96534
H   3.12362 0.22884 -2.78739
C   7.53092 0.06717 0.84606
H   7.39828 0.61253 1.79522
H   8.36993 0.54011 0.31201
H   7.84013 -0.96370 1.09514
C   7.27592 0.81170 -2.30245
H   7.51078 0.00889 -3.02416
H   8.20099 1.03163 -1.74683
H   7.02047 1.71472 -2.88118
C   4.54247 -0.53752 1.80840
H   3.48588 -0.82537 1.65669
C   4.73107 -0.21335 -4.14677
H   5.73271 0.13642 -4.44474
H   4.06787 -0.09048 -5.01914
H   4.78512 -1.28837 -3.90926
C   4.08864 2.09971 -3.20724
H   3.62329 2.60010 -2.34289
H   3.47280 2.30153 -4.09994
H   5.08518 2.53866 -3.38425
C   5.27095 -1.73873 2.42997
H   5.28959 -2.59638 1.73742
H   4.72735 -2.04722 3.33789
H   6.30559 -1.50087 2.72605
C   4.55075 0.71890 2.69259
H   5.57390 1.07408 2.90479
H   4.06287 0.48601 3.65346
H   3.97855 1.52300 2.20226
C   1.96147 1.76560 0.07601
C   2.07438 2.99886 0.22601
H -0.55392 1.09798 -0.74474
C   2.14925 4.41666 0.39684
C   3.13599 5.00709 1.23268
C   1.23611 5.28102 -0.26723
C   3.20379 6.39641 1.39461
C   1.31307 6.66955 -0.10241
C   2.29537 7.23681 0.72796
H   3.84589 4.35436 1.75077
H   0.46610 4.84019 -0.90745
H   3.97268 6.82786 2.04557
H   0.59677 7.31418 -0.62374
H   2.35189 8.32311 0.85528

INT2(E-P)$_{Ph}$

SCF (BP86) Energy = -2646.66192283
Enthalpy 0K = -2645.460543
Enthalpy 298K = -2645.379925
Free Energy 298K = -2645.582936
Lowest Frequency = 6.4462 cm^{-1}
Second Frequency = 12.1534 cm^{-1}
SCF (BP86-D3BJ) Energy = -2647.06108760
SCF (C6H6) Energy = -2646.66853118
SCF (BS2) Energy = -3458.93966499

Si 4.10531 1.58850 1.90891
Al 1.88264 0.01677 0.18448
Si 4.40751 0.35696 -1.84883
N   3.01229 -0.55273 -1.14638
N   2.39851 1.31754 1.37403
C   2.72157 -1.85946 -1.70767
C   1.02922 3.39637 1.48854
C   1.37060 2.11382 2.02280
C   3.29581 -3.02974 -1.11825
C   1.87611 -1.99460 -2.85230
C   5.67691 0.89072 -0.52153
H   6.01273 -0.01130 0.02500
H   6.55925 1.21057 -1.11488
C   1.08527 0.32951 3.89429
H   1.77201 -0.21246 3.22055
C   0.71776 1.64749 3.20701
C   1.73015 3.97956 0.25985
H   2.58334 3.31630 0.03500
C   0.24408 -0.97057 0.39334
C   1.22424 -0.79644 -3.54449
H   1.46763 0.10112 -2.95052
C   1.62618 -3.28168 -3.36969
H   0.98096 -3.38173 -4.25014
C   4.16955 3.05383 3.11861
H   4.02456 4.01414 2.59813
H   5.16202 3.07803 3.60190
H   3.40476 2.98474 3.90773

C -0.98406 -3.20530 1.13752
C -0.60920 -1.86993 0.71458
C -0.27490 2.45382 3.80088
H -0.76433 2.10243 4.71697
C   4.20354 -2.95207 0.11001
H   4.47621 -1.88980 0.23575
C -2.08201 -3.43506 2.00114
H -2.67300 -2.57961 2.34027
C   0.02204 4.15420 2.11495
H -0.24513 5.13081 1.69598
C -0.63336 3.69455 3.26253
H -1.40328 4.30738 3.74444
C   4.79258 0.04046 2.78488
H   4.25282 -0.16320 3.72397
H   5.85945 0.18618 3.03196
H   4.71887 -0.86024 2.15339
C   0.80427 3.98840 -0.97574
H   0.38121 2.99298 -1.18747
H   1.35048 4.34082 -1.86930
H -0.05309 4.66690 -0.82247
C   3.80697 1.91910 -2.75660
H   3.23840 1.66413 -3.66568
H   4.66638 2.54389 -3.05847
H   3.15297 2.53542 -2.11714
C   3.00375 -4.29367 -1.66752
H   3.44351 -5.18788 -1.21105
C   5.28564 2.01425 0.46512
H   4.87673 2.88728 -0.07789
H   6.19645 2.39396 0.97422
C -0.14000 -0.57580 4.14439
H -0.84189 -0.11889 4.86483
H   0.17838 -1.54506 4.56693
H -0.68360 -0.77343 3.20751
C   5.34194 -0.77725 -3.05577
H   5.83153 -1.61054 -2.52474
H   6.12681 -0.19616 -3.57076
H   4.68165 -1.21462 -3.82090
C -0.23400 -4.31728 0.67435
H   0.60481 -4.14780 -0.00797
C   2.28876 5.39817 0.51266
H   1.47814 6.13111 0.67143
H   2.86965 5.74055 -0.36180
H   2.94686 5.43567 1.39719
C   1.82786 0.58123 5.22869
H   2.74142 1.18196 5.08784
H   2.11703 -0.37510 5.70039
H   1.18175 1.12422 5.94155
C -0.31399 -0.92236 -3.58606
H -0.63166 -1.78240 -4.20337
H -0.76269 -0.01096 -4.01579
H -0.72833 -1.04335 -2.57197
C -2.40454 -4.73561 2.41119
H -3.24871 -4.89540 3.09074
C   3.45025 -3.39720 1.38496
H   3.13558 -4.45301 1.30795
H   2.53749 -2.79713 1.54880
H   4.09077 -3.29921 2.27955
C   2.17747 -4.42864 -2.78945
H   1.96924 -5.41854 -3.20975
C   1.77949 -0.58856 -4.97302
H   2.87297 -0.44449 -4.97536
H   1.31755 0.29833 -5.44177
H   1.55880 -1.45924 -5.61631
C -0.57482 -5.61547 1.07692
H   0.00991 -6.46442 0.70667
C -1.65508 -5.83168 1.94944
H -1.91440 -6.84791 2.26439
C   5.51324 -3.75316 -0.05260
H   6.17575 -3.58391 0.81437
H   6.05995 -3.45764 -0.96399
H   5.32747 -4.84004 -0.11327
N -4.39262 -0.18469 1.23890
N -4.37221 -1.62026 -0.39304
C -5.72405 -0.59611 1.05613
C -5.71172 -1.50678 0.01844
C -3.56037 -0.80201 0.34395
Cu -1.67078 -0.33019 0.02272
C -3.86984 0.86763 2.15152
H -2.77570 0.79909 2.01010
C -6.86108 -2.27485 -0.55942
H -6.89363 -2.21573 -1.66004
H -7.81088 -1.86345 -0.18326
H -6.83500 -3.34432 -0.28413
C -6.88851 -0.14234 1.88266
H -6.86549 -0.55679 2.90644
H -7.82918 -0.47579 1.41708
H -6.93800 0.95559 1.96954
C -3.82393 -2.38428 -1.54347
H -2.73301 -2.26808 -1.41483
C -4.17722 0.57699 3.62866
H -5.23899 0.73274 3.87979
H -3.58500 1.26395 4.25523
H -3.89910 -0.45470 3.90061
C -4.29798 2.27061 1.69515
H -4.00229 2.42805 0.64571
H -3.77999 3.02230 2.31378
H -5.38410 2.43440 1.79995
C -4.14708 -3.88465 -1.47374
H -3.89336 -4.29605 -0.48350
H -3.54133 -4.41497 -2.22718
H -5.20531 -4.10106 -1.69202
C -4.20998 -1.72467 -2.87772
H -5.29313 -1.79085 -3.07707
H -3.68627 -2.23259 -3.70480
H -3.91288 -0.66381 -2.87732
C -1.84743 1.29358 -0.96577
C -2.21785 2.24637 -1.67931
C -2.62164 3.35830 -2.47961
C -2.70937 3.25565 -3.89518
C -2.95499 4.60711 -1.88500
C -3.11061 4.34837 -4.67331
C -3.35493 5.69530 -2.67054
C -3.43612 5.57506 -4.06867
H -2.45645 2.30187 -4.36927
H -2.89165 4.70234 -0.79638
H -3.16950 4.24263 -5.76255
H -3.60577 6.64658 -2.18754
H -3.74895 6.42779 -4.68036

P_{Ph}

SCF (BP86) Energy = -2646.69404035
Enthalpy 0K = -2645.492058
Enthalpy 298K = -2645.412147
Free Energy 298K = -2645.608280
Lowest Frequency = 16.3582 cm^{-1}
Second Frequency = 17.5552 cm^{-1}
SCF (BP86-D3BJ) Energy = -2647.11270110
SCF (C6H6) Energy = -2646.69989499
SCF (BS2) Energy = -3458.96621035

Cu -1.42963 0.01380 0.05826
Si 4.17156 -1.57946 -1.38529
Al 1.61170 -0.07642 0.03465
Si 4.23190 0.90885 1.69007
N -4.03653 -1.18045 -0.71856
N   2.47090 -1.52975 -0.81236
N -4.34677 0.78397 0.16599
N   2.69936 1.24441 0.82790
C -3.36597 -0.10875 -0.18231
C   0.15885 -0.76305 1.24721
C   1.10220 -1.75597 -3.47206
H   1.63614 -0.89193 -3.04108
C   0.96345 -2.79828 -2.35904
C -0.90571 -0.98596 1.89732
C   2.20695 2.60184 0.84823
C -1.70160 -1.40609 3.02826
C   1.51121 -3.74057 -0.14559
C   5.59222 0.17927 0.55688
H   5.74878 0.88644 -0.28059
H   6.51792 0.24714 1.16717
C -1.65955 2.29636 -2.30178
C   5.42675 -1.26142 0.02464
H   5.19414 -1.96027 0.85124
H   6.39321 -1.61772 -0.39101

C 1.64530 -2.67683 -1.10097
C 3.94924 -0.32011 3.12593
H 3.33547 0.13233 3.92267
H 4.91067 -0.63152 3.57201
H 3.43050 -1.23141 2.78194
C -1.03277 -1.70163 4.24722
H 0.05474 -1.59620 4.29058
C 0.04691 -4.97239 -1.68085
H -0.54881 -5.86316 -1.91104
C -4.00368 2.07705 0.81968
H -2.90196 2.10346 0.74082
C 1.39285 3.08412 1.92656
C 4.60161 -3.30620 -2.09078
H 5.04034 -3.96460 -1.32298
H 5.35446 -3.18989 -2.89031
H 3.73009 -3.82713 -2.51727
C -3.29561 -2.38190 -1.19792
H -2.23781 -2.06788 -1.12739
C 0.71303 -4.85896 -0.45508
H 0.62792 -5.66878 0.27909
C -2.73895 1.81863 -3.08438
H -2.95079 0.74585 -3.09798
C -3.82332 -1.96200 4.11754
H -4.91275 -2.06139 4.06235
C 0.97935 2.20397 3.10893
H 1.29511 1.17322 2.87373
C -3.15046 -2.25564 5.31442
H -3.70995 -2.58473 6.19605
C -5.42451 -0.97029 -0.69489
C -0.80745 1.41137 -1.54639
C -0.26073 -1.25466 -3.99520
H -0.82821 -2.06091 -4.49449
H -0.11452 -0.44887 -4.73611
H -0.87457 -0.85075 -3.17468
C -5.62160 0.27658 -0.13292
C 1.93451 -2.30514 -4.65568
H 2.93268 -2.64547 -4.33720
H 2.06773 -1.52952 -5.43139
H 1.42654 -3.16575 -5.12776
C 0.18261 -3.94323 -2.61999
H -0.30762 -4.03786 -3.59690
C 2.25050 -3.73346 1.19397
H 2.69725 -2.72961 1.30217
C -3.11063 -1.53791 2.98665
H -3.63567 -1.30080 2.05828
C -1.75252 -2.12035 5.37239
H -1.21695 -2.34314 6.30129
C -1.38814 3.68949 -2.28360
H -0.55492 4.05894 -1.67738
C 0.21847 0.87585 -1.04698
C 2.53766 3.51013 -0.21047
C -2.17603 4.57024 -3.03653
H -1.95396 5.64246 -3.01627
C -3.59047 -2.70742 -2.66969
H -2.84750 -3.44283 -3.01781
H -3.50099 -1.81172 -3.30546
H -4.59121 -3.14627 -2.81294
C 3.42160 3.09489 -1.38415
H 3.71834 2.05100 -1.18832
C -3.24084 4.08646 -3.81634
H -3.85071 4.77887 -4.40592
C 0.94850 4.42207 1.91892
H 0.33956 4.78245 2.75678
C 4.53475 -0.32686 -2.78132
H 4.02328 -0.61050 -3.71558
H 5.62084 -0.30883 -2.98594
H 4.22558 0.69852 -2.52841
C 1.31351 -3.98117 2.39509
H 0.90454 -5.00774 2.38745
H 0.46666 -3.27940 2.39407
H 1.86753 -3.86104 3.34348
C -6.91538 0.99869 0.09130
H -6.99067 1.42052 1.10687
H -7.75825 0.30160 -0.03655
H -7.06298 1.82607 -0.62496
C 2.65628 3.13259 -2.72584
H 1.76259 2.48831 -2.69246
H 3.30084 2.78635 -3.55377
H 2.32673 4.15894 -2.96922
C 4.97730 2.52348 2.37964
H 5.42971 3.12486 1.57321
H 5.77505 2.28167 3.10384
H 4.23456 3.15951 2.88507
C -0.55207 2.19291 3.30949
H -0.93016 3.18695 3.60967
H -0.83418 1.47681 4.10094
H -1.06933 1.89366 2.38275
C -3.51728 2.70820 -3.83743
H -4.34202 2.32335 -4.44691
C -4.55899 3.29203 0.06061
H -5.64754 3.40606 0.19016
H -4.32862 3.23002 -1.01453
H -4.08398 4.20415 0.45762
C 1.66719 2.63505 4.42570
H 2.76544 2.58145 4.35494
H 1.34902 1.98589 5.26156
H 1.40098 3.67436 4.69054
C -3.48150 -3.59301 -0.27024
H -4.49705 -4.01979 -0.33461
H -3.27615 -3.32496 0.77775
H -2.76317 -4.37524 -0.56557
C 3.39693 -4.77144 1.21646
H 3.93953 -4.73025 2.17801
H 4.12238 -4.60004 0.40649
H 3.00143 -5.79647 1.09803
C 4.70858 3.94513 -1.47671
H 4.47957 5.00421 -1.69266
H 5.35983 3.57487 -2.28857
H 5.28465 3.91607 -0.53667
C -4.37302 2.07443 2.31231
H -3.96954 2.98358 2.78778
H -3.94204 1.19987 2.82472
H -5.46486 2.07329 2.47090
C 2.05420 4.83282 -0.17920
H 2.32089 5.51396 -0.99662
C -6.45228 -1.92494 -1.22220
H -6.46840 -1.95802 -2.32597
H -7.45540 -1.61394 -0.89098
H -6.29206 -2.95379 -0.86086
C 1.26536 5.30042 0.87762
H 0.91107 6.33714 0.89479

TS(1,1)$_{Ph}$
SCF (BP86) Energy = -2647.82234971
Enthalpy 0K = -2646.604752
Enthalpy 298K = -2646.524536
Free Energy 298K = -2646.722522
Lowest Frequency = -523.2578 cm^{-1}
Second Frequency = 11.1792 cm^{-1}
SCF (BP86-D3BJ) Energy = -2648.24372376
SCF (C6H6) Energy = -2647.82765950
SCF (BS2) Energy = -3460.09509483

Cu -1.73362 -0.23841 -0.09446
Si 3.78201 1.37928 2.10249
Al 1.71930 -0.09779 0.06667
Si 4.41782 -2.01070 0.12845
N -4.68117 0.54871 0.00431
N 2.66562 1.40891 0.70121
N -4.34372 -1.46032 -0.75660
N 2.65605 -1.74224 -0.09053
C -3.69024 -0.36251 -0.25496
H 0.95344 0.21091 -1.44704
C 0.60460 3.19939 1.99363
H 0.85225 2.15026 2.22649
C 1.42085 3.59020 0.75977
C 1.85010 -2.83006 -0.58877
C 2.97526 3.11780 -1.08954
C 5.01442 -1.32985 1.81471
H 4.31578 -1.66124 2.60633
H 5.96086 -1.87528 2.01279
C -1.76375 -0.40720 3.08177
C 5.26956 0.19303 1.89677
H 5.86842 0.53322 1.03085

H 5.89565 0.41946 2.78622
C 2.34673 2.69399 0.12764
C 5.54839 -1.19686 -1.18280
H 5.42077 -1.64363 -2.18120
H 6.59904 -1.36062 -0.87824
H 5.39751 -0.11063 -1.27299
C 1.86138 5.29644 -0.94917
H 1.69714 6.30455 -1.34596
C -3.58230 -2.66337 -1.19051
H -2.55672 -2.44530 -0.83918
C 1.77855 -3.11985 -1.99554
C 4.55936 3.10080 2.35883
H 5.28077 3.32284 1.55409
H 5.10775 3.12256 3.31689
H 3.81794 3.91407 2.36639
C -4.36031 1.88685 0.57013
H -3.27486 1.81527 0.75806
C 2.72142 4.40494 -1.60005
H 3.22363 4.71686 -2.52355
C -3.08435 -0.90821 3.17282
H -3.58126 -1.24492 2.25844
C 2.62037 -2.37295 -3.03488
H 3.09217 -1.52071 -2.51740
C -5.94240 0.03807 -0.34498
C -1.03470 -0.35605 1.83115
C -0.91036 3.26134 1.69346
H -1.24395 4.29880 1.51036
H -1.48921 2.86355 2.54612
H -1.15597 2.66502 0.79904
C -5.72708 -1.23750 -0.82969
C 0.92293 4.06864 3.23132
H 1.98333 4.00166 3.52342
H 0.31174 3.75123 4.09560
H 0.69776 5.13341 3.04068
C 1.21300 4.87458 0.21657
H 0.52038 5.55750 0.72338
C 3.93951 2.21421 -1.85207
H 3.94279 1.24762 -1.32132
C -1.11913 0.02684 4.26971
H -0.09413 0.40440 4.20902
C 0.00025 -0.30310 1.09244
C 1.09369 -3.64946 0.31936
C -1.78224 -0.03669 5.50167
H -1.26914 0.30302 6.40766
C -5.05440 2.13509 1.91902
H -4.62811 3.04354 2.37585
H -4.88500 1.29635 2.61290
H -6.13881 2.30031 1.81061
C 1.16028 -3.46056 1.83763
H 1.50126 -2.42588 2.01582
C -3.09338 -0.53691 5.57982
H -3.60699 -0.58829 6.54542
C 0.95597 -4.17177 -2.44914
H 0.91438 -4.38552 -3.52363
C 2.88671 0.81285 3.69723
H 2.17855 1.57494 4.06226
H 3.61478 0.61295 4.50350
H 2.32189 -0.11927 3.52008
C 3.47284 1.95591 -3.30111
H 3.46425 2.88469 -3.89948
H 2.45550 1.53163 -3.32093
H 4.15273 1.24500 -3.80354
C -6.73847 -2.23164 -1.31407
H -6.45183 -2.68533 -2.27693
H -7.70939 -1.73398 -1.46343
H -6.89982 -3.05326 -0.59383
C -0.20340 -3.64823 2.53498
H -0.99403 -3.05835 2.04606
H -0.13887 -3.32325 3.58762
H -0.51718 -4.70800 2.54462
C 4.82638 -3.88375 0.06019
H 5.07393 -4.28179 1.05870
H 5.71012 -4.04370 -0.58171
H 3.99968 -4.48735 -0.34683
C 1.79704 -1.80868 -4.21400
H 1.31079 -2.61203 -4.79634
H 2.45881 -1.25924 -4.90636
H 1.01337 -1.11381 -3.87358
C -3.73841 -0.97490 4.41102
H -4.75689 -1.37484 4.46410
C -4.04961 -3.94660 -0.48617
H -5.01801 -4.31061 -0.86576
H -4.12976 -3.79736 0.60309
H -3.30340 -4.73770 -0.66537
C 3.73712 -3.29466 -3.58312
H 4.36058 -3.71291 -2.77640
H 4.39502 -2.74230 -4.27817
H 3.30316 -4.14497 -4.13967
C -4.58979 3.01731 -0.44529
H -5.66118 3.19405 -0.63961
H -4.08775 2.80080 -1.40090
H -4.16974 3.95332 -0.04155
C 5.38110 2.77069 -1.83214
H 6.07466 2.08230 -2.34767
H 5.74532 2.91291 -0.80044
H 5.43938 3.74856 -2.34312
C 2.19101 -4.41641 2.48369
H 1.91912 -5.47015 2.29121
H 2.22052 -4.26958 3.57844
H 3.20483 -4.25506 2.09014
C -3.52806 -2.78222 -2.72109
H -2.85275 -3.61008 -2.99421
H -3.12308 -1.85265 -3.15323
H -4.51699 -2.99343 -3.16222
C 0.29337 -4.69158 -0.19073
H -0.26916 -5.31876 0.50966
C -7.24634 0.75720 -0.17761
H -7.55036 0.83842 0.88104
H -8.04378 0.20924 -0.70346
H -7.22003 1.77676 -0.59519
C 0.21087 -4.95662 -1.56313
H -0.40881 -5.78050 -1.93502
C -1.73142 1.62221 -2.81414
C -0.96030 2.73585 -2.38991
H -0.11116 2.58040 -1.71584
C -1.40643 0.27247 -2.39253
C -2.81407 1.84465 -3.70189
H -3.40416 0.98451 -4.03464
C -0.46573 -0.43627 -1.85907
C -3.11129 3.13644 -4.15906
H -3.94372 3.29044 -4.85428
C -2.34318 4.23097 -3.72515
H -2.57749 5.24049 -4.07986
C -1.27043 4.02611 -2.83912
H -0.65916 4.86810 -2.49860
H -0.14894 -1.47638 -1.80710

$1,1_{Ph}$

SCF (BP86) Energy = -2647.88456029
Enthalpy 0K = -2646.661133
Enthalpy 298K = -2646.581118
Free Energy 298K = -2646.777076
Lowest Frequency = 10.6893 cm^{-1}
Second Frequency = 15.5103 cm^{-1}
SCF (BP86-D3BJ) Energy = -2648.30895512
SCF (C6H6) Energy = -2647.88972898
SCF (BS2) Energy = -3460.15716635

Cu -1.74975 0.25633 -0.22410
Si 3.98142 0.56169 1.97357
Al 1.54701 -0.21958 0.01935
Si 4.04587 -2.28423 -0.71673
N -4.67836 0.89163 0.22314
N 2.73901 1.03674 0.75550
N -4.38296 -0.53646 -1.38854
N 2.30771 -1.90059 -0.45737
C -3.70092 0.20037 -0.44971
H 1.32616 0.98335 -2.62591
C 0.79893 2.63927 2.39751
H 0.91301 1.54343 2.34034
C 1.73811 3.25293 1.35589
C 1.36044 -2.97462 -0.66795
C 3.46987 3.13398 -0.39378
C 5.14086 -1.91174 0.80890

```
H  4.71878 -2.48109  1.65999
H  6.09886 -2.42216  0.57211
C -2.18095 -1.20018  2.58911
C  5.41906 -0.45248  1.22252
H  5.82822  0.12032  0.36883
H  6.21597 -0.42846  1.99546
C  2.63045  2.46375  0.55766
C  4.77131 -1.37205 -2.23111
H  4.38954 -1.80544 -3.17074
H  5.87180 -1.47179 -2.24049
H  4.53205 -0.29735 -2.23683
C  2.55185  5.30430  0.28282
H  2.52858  6.39523  0.18706
C -3.64574 -1.39466 -2.35202
H -2.60318 -1.32874 -1.98872
C  0.81069 -3.24356 -1.96944
C  4.79311  2.10476  2.74456
H  5.39033  2.67166  2.01264
H  5.46986  1.77895  3.55453
H  4.05796  2.80269  3.17377
C -4.32259  1.83394  1.31622
H -3.24454  1.64185  1.45890
C  3.41446  4.53769 -0.50752
H  4.07072  5.03702 -1.23059
C -3.30470 -2.02042  2.31617
H -3.50381 -2.30980  1.28043
C  1.33225 -2.58080 -3.25199
H  1.97874 -1.73830 -2.95010
C -5.95359  0.60444 -0.29174
C -1.29273 -0.75869  1.54071
C -0.67870  2.97202  2.09512
H -0.87532  4.05630  2.17633
H -1.33896  2.45624  2.81589
H -0.95872  2.65300  1.07707
C -5.76640 -0.30464 -1.31398
C  1.14759  3.08277  3.83750
H  2.17097  2.79020  4.12369
H  0.45067  2.62501  4.56283
H  1.07058  4.17912  3.95024
C  1.72023  4.65296  1.19909
H  1.03877  5.24644  1.82003
C  4.44200  2.37805 -1.29981
H  4.31943  1.30863 -1.06055
C -1.93281 -0.81919  3.93429
H -1.06348 -0.19238  4.15463
C -0.20953 -0.49544  0.92635
C  0.95920 -3.81529  0.42484
C -2.78211 -1.24553  4.96406
H -2.57370 -0.94097  5.99540
C -5.03629  1.50759  2.63768
H -4.56577  2.08835  3.44824
H -4.93983  0.43878  2.88644
H -6.10453  1.77856  2.61893
C  1.65644 -3.77705  1.78704
H  2.41777 -2.98038  1.73082
C -3.88771 -2.06559  4.68188
H -4.54603 -2.40162  5.48963
C -0.18527 -4.23285 -2.11002
H -0.59437 -4.43419 -3.10737
C  3.17884 -0.44087  3.38353
H  2.52141  0.20538  3.98935
H  3.95122 -0.86095  4.05230
H  2.56868 -1.27853  3.00945
C  4.11516  2.57879 -2.79738
H  4.24846  3.63276 -3.10045
H  3.07517  2.29654 -3.03071
H  4.78448  1.96588 -3.42713
C -6.80384 -0.97283 -2.16432
H -6.57083 -0.91374 -3.24034
H -7.77949 -0.48448 -2.01485
H -6.93150 -2.04021 -1.91041
C  0.70686 -3.44857  2.95721
H  0.23690 -2.46338  2.82907
H  1.26051 -3.44874  3.91325
H -0.10064 -4.19739  3.04320
C  4.29198 -4.15962 -1.01625
H  4.62032 -4.66031 -0.08994
H  5.08280 -4.31471 -1.77095
H  3.38090 -4.66890 -1.36648
C  0.21412 -2.03375 -4.16886
H -0.40265 -2.85156 -4.58296
H  0.65359 -1.49431 -5.02627
H -0.45395 -1.33564 -3.63922
C -4.14020 -2.45354  3.35362
H -4.99643 -3.09740  3.12449
C -4.07579 -2.86804 -2.28357
H -5.06527 -3.04109 -2.73751
H -4.09555 -3.22657 -1.24183
H -3.34223 -3.48036 -2.83260
C  2.20403 -3.56953 -4.06574
H  3.05551 -3.94904 -3.48006
H  2.60206 -3.08032 -4.97314
H  1.60679 -4.44119 -4.38822
C -4.48622  3.29993  0.88159
H -5.54250  3.57055  0.71327
H -3.91758  3.50217 -0.04008
H -4.09691  3.96028  1.67437
C  5.91602  2.76187 -1.03632
H  6.59126  2.17094 -1.68067
H  6.20696  2.58373  0.01183
H  6.09773  3.82932 -1.25540
C  2.38170 -5.11489  2.07189
H  1.65767 -5.93922  2.20200
H  2.97166 -5.04269  3.00311
H  3.06179 -5.39806  1.25313
C -3.68310 -0.81514 -3.77568
H -3.00833 -1.39744 -4.42462
H -3.34046  0.23195 -3.77928
H -4.69148 -0.85783 -4.22054
C -0.04564 -4.78166  0.22951
H -0.35303 -5.40413  1.07817
C -7.24287  1.16787  0.22416
H -7.52665  0.74192  1.20286
H -8.05838  0.93996 -0.48006
H -7.20651  2.26388  0.33755
C -0.63863 -4.98405 -1.02097
H -1.41597 -5.74473 -1.15316
C -0.78874  2.58289 -1.90711
C  0.22842  3.56253 -2.04175
H  1.25030  3.32297 -1.73491
C -0.53683  1.19230 -1.47369
C -2.11476  2.95632 -2.25268
H -2.91006  2.21408 -2.12800
C  0.63070  0.55428 -1.88565
C -2.40201  4.23019 -2.76292
H -3.42907  4.48274 -3.04986
C -1.37991  5.18646 -2.88784
H -1.60556  6.19003 -3.26430
C -0.06894  4.84858 -2.51053
H  0.73291  5.59138 -2.57818
H  0.60415 -0.54998 -1.90223
```

$TS(Z)_{Ph}$

SCF (BP86) Energy = -2647.81743667
Enthalpy 0K = -2646.600516
Enthalpy 298K = -2646.520189
Free Energy 298K = -2646.718656
Lowest Frequency = -557.6960 cm^{-1}
Second Frequency = 12.2297 cm^{-1}
SCF (BP86-D3BJ) Energy = -2648.24208227
SCF (C6H6) Energy = -2647.82275374
SCF (BS2) Energy = -3460.09023606

```
Cu  1.87521  0.15671 -0.04608
Si -3.81025 -1.03575  2.13600
Al -1.59533  0.17117  0.07297
Si -4.05936  2.39489  0.17795
N   4.61015 -1.04699 -0.46321
N  -2.72988 -1.20555  0.71373
N   4.65813  1.12588 -0.53294
N  -2.33799  1.91701 -0.04869
C   3.81609  0.06301 -0.32586
H  -0.86038 -0.25556 -1.44524
C  -0.81077 -3.22764  1.86143
```

```
H  -0.91741 -2.15757  2.10527
C  -1.76768 -3.52445  0.70557
C  -1.42859  2.95612 -0.47484
C  -3.44552 -2.89246 -0.98138
C  -4.76305  1.76580  1.84232
H  -4.05459  2.02332  2.65261
H  -5.65049  2.41011  2.01626
C   1.85604 -0.12657  3.11497
C  -5.17930  0.28040  1.91992
H  -5.79547  0.00216  1.04379
H  -5.83867  0.12051  2.79944
C  -2.63749 -2.52987  0.14628
C  -5.27063  1.78274 -1.17044
H  -5.10518  2.29006 -2.13371
H  -6.29932  2.02157 -0.84199
H  -5.22311  0.69782 -1.34591
C  -2.55820 -5.17604 -0.92975
H  -2.53952 -6.19560 -1.33064
C   4.13925  2.52011 -0.50935
H   3.09476  2.38661 -0.17432
C  -1.34722  3.35079 -1.85399
C  -4.73484 -2.66983  2.46184
H  -5.45918 -2.87676  1.65602
H  -5.29750 -2.59041  3.40863
H  -4.06492 -3.54046  2.53029
C   4.05480 -2.41017 -0.24699
H   2.97990 -2.21074 -0.08859
C  -3.38566 -4.20098 -1.49789
H  -4.01383 -4.45997 -2.35860
C   3.26588 -0.04048  3.20979
H   3.83926  0.14444  2.29646
C  -2.23984  2.74729 -2.94215
H  -2.79395  1.91256 -2.48075
C   5.93475 -0.68870 -0.76314
C   1.14957  0.02987  1.86015
C   0.65618 -3.46336  1.43615
H   0.85079 -4.53284  1.23630
H   1.34724 -3.13448  2.23199
H   0.89081 -2.89913  0.51836
C   5.96404  0.69169 -0.81001
C  -1.12533 -4.05159  3.13058
H  -2.14370 -3.85879  3.50590
H  -0.41313 -3.80294  3.93784
H  -1.04269 -5.13622  2.93664
C  -1.75639 -4.82499  0.16154
H  -1.09690 -5.57915  0.60774
C  -4.37862 -1.88892 -1.65057
H  -4.31029 -0.96739 -1.04962
C   1.11029 -0.35917  4.30044
H   0.01995 -0.41863  4.23871
C   0.13875  0.16977  1.09774
C  -0.59870  3.63542  0.48337
C   1.76109 -0.50523  5.53183
H   1.16890 -0.68467  6.43552
C   4.60308 -3.05663  1.03609
H   4.04624 -3.98669  1.23705
H   4.47171 -2.38822  1.90191
H   5.67012 -3.32201  0.95008
C  -0.67646  3.34819  1.98610
H  -1.10973  2.33947  2.09955
C   3.16178 -0.42010  5.61266
H   3.66610 -0.53350  6.57788
C  -0.44844  4.36775 -2.23543
H  -0.39805  4.66086 -3.29055
C  -2.81739 -0.53483  3.69165
H  -2.16063 -1.35578  4.02465
H  -3.49343 -0.27860  4.52662
H  -2.18598  0.34801  3.48943
C  -3.92034 -1.55497 -3.08778
H  -3.96328 -2.44615 -3.73968
H  -2.88312 -1.18061 -3.09986
H  -4.57092 -0.78174 -3.53406
C   7.13433  1.58831 -1.07914
H   6.92563  2.32287 -1.87442
H   7.99587  0.98826 -1.41012
H   7.45016  2.14931 -0.18213
C   0.69975  3.36204  2.68418
H   1.42927  2.72770  2.15662
H   0.60530  2.98207  3.71599
H   1.11441  4.38401  2.75505
C  -4.23520  4.30580  0.18540
H  -4.39785  4.69333  1.20516
H  -5.11725  4.59063 -0.41445
H  -3.35889  4.82374 -0.23459
C  -1.44702  2.17927 -4.14039
H  -0.89548  2.97278 -4.67642
H  -2.13887  1.71554 -4.86563
H  -0.71935  1.41774 -3.82279
C   3.90923 -0.18498  4.44676
H   5.00088 -0.11138  4.50099
C   4.85468  3.39827  0.52931
H   5.87917  3.66588  0.22392
H   4.89628  2.89971  1.51144
H   4.29110  4.33798  0.64944
C  -3.25958  3.79350 -3.45437
H  -3.85923  4.22483 -2.63677
H  -3.95028  3.33927 -4.18765
H  -2.74192  4.62886 -3.95967
C   4.18713 -3.30871 -1.48524
H   5.22677 -3.62864 -1.66389
H   3.80416 -2.80198 -2.38503
H   3.58404 -4.21837 -1.33068
C  -5.85123 -2.35461 -1.64131
H  -6.50695 -1.57428 -2.06733
H  -6.20154 -2.57280 -0.61811
H  -5.99272 -3.26991 -2.24363
C  -1.61438  4.34765  2.70372
H  -1.24917  5.38321  2.57965
H  -1.65561  4.13062  3.78639
H  -2.63921  4.30455  2.30755
C   4.09657  3.13617 -1.91609
H   3.54836  4.09192 -1.87435
H   3.55673  2.46489 -2.60294
H   5.10327  3.34181 -2.31745
C   0.28550  4.64153  0.04340
H   0.90574  5.16141  0.78193
C   7.05869 -1.65021 -1.00367
H   7.19502 -2.35880 -0.16963
H   8.00400 -1.09745 -1.11628
H   6.91296 -2.24309 -1.92325
C   0.37219  5.01205 -1.30409
H   1.05583  5.80802 -1.62040
C   0.75308 -1.33840 -2.81629
C  -0.01413 -2.49167 -2.54246
H  -0.76777 -2.46231 -1.74993
C   0.60340 -0.07859 -2.08393
C   1.70526 -1.38687 -3.87009
H   2.28574 -0.48453 -4.08378
C   1.24528  1.03710 -1.94285
C   1.87903 -2.55737 -4.61797
H   2.60732 -2.57296 -5.43635
C   1.11507 -3.70348 -4.32942
H   1.24873 -4.61676 -4.91920
C   0.17269 -3.66427 -3.28965
H  -0.43596 -4.54274 -3.05433
H   0.94176  2.02503 -1.59404
```

Z_{Ph}

SCF (BP86) Energy = -2647.90230884
Enthalpy 0K = -2646.677639
Enthalpy 298K = -2646.597829
Free Energy 298K = -2646.793912
Lowest Frequency = 9.6988 cm^{-1}
Second Frequency = 14.8811 cm^{-1}
SCF (BP86-D3BJ) Energy = -2648.32796050
SCF (C6H6) Energy = -2647.90779511
SCF (BS2) Energy = -3460.17273959

```
Cu  1.58966  0.05008 -0.11811
Si -3.98266 -1.00445  1.78119
Al -1.50294  0.13092 -0.00984
Si -4.12892  2.10041 -0.61724
N   4.50416 -0.80982 -0.32432
N  -2.62747 -1.25417  0.62484
```

N 4.15903 1.25070 -0.93196
N -2.37313 1.81086 -0.36875
C 3.51568 0.12826 -0.47108
H 1.47736 0.00413 -2.89179
C -0.84544 -2.86751 2.44765
H -1.04284 -1.78202 2.45568
C -1.56910 -3.44752 1.22921
C -1.51654 2.96004 -0.53202
C -2.93567 -3.26944 -0.81292
C -5.20539 1.53988 0.86103
H -4.80462 2.00802 1.78043
H -6.18106 2.03984 0.68221
C 1.99755 0.59094 3.05884
C -5.42429 0.02842 1.06803
H -5.75606 -0.45106 0.12700
H -6.25530 -0.13780 1.78599
C -2.35938 -2.64268 0.34074
C -4.83527 1.23333 -2.16859
H -4.42860 1.66257 -3.09828
H -5.93244 1.36713 -2.19034
H -4.63276 0.15125 -2.18199
C -2.01878 -5.43804 -0.13616
H -1.90750 -6.51486 -0.30589
C 3.40225 2.50771 -1.18714
H 2.35186 2.20178 -1.02304
C -1.08838 3.40294 -1.83448
C -4.78666 -2.67495 2.23150
H -4.07020 -3.42694 2.59431
H -5.30195 -3.11046 1.35882
H -5.54218 -2.50830 3.01976
C 4.19972 -2.16792 0.20567
H 3.09661 -2.16936 0.25159
C -2.75195 -4.64800 -1.02863
H -3.20648 -5.11196 -1.91208
C 3.37247 0.91729 2.97501
H 3.82039 1.05681 1.98740
C -1.54807 2.73425 -3.13630
H -2.03546 1.78321 -2.85938
C 5.75702 -0.28420 -0.68233
C 1.14541 0.46575 1.89660
C 0.68331 -3.06467 2.33166
H 0.95412 -4.13594 2.35260
H 1.20375 -2.56691 3.16858
H 1.06637 -2.63670 1.39034
C 5.53737 1.02386 -1.06862
C -1.34117 -3.46507 3.78459
H -2.41643 -3.28574 3.94527
H -0.79268 -3.01886 4.63376
H -1.17632 -4.55697 3.82277
C -1.42687 -4.82746 0.97515
H -0.83907 -5.43626 1.67301
C -3.75307 -2.47492 -1.82698
H -3.82578 -1.45208 -1.42139
C 1.42432 0.40303 4.34495
H 0.36096 0.15714 4.41685
C 0.03930 0.41088 1.27361
C -1.09172 3.72078 0.61269
C 2.20511 0.53773 5.49909
H 1.74461 0.38867 6.48149
C 4.73571 -2.35768 1.63439
H 4.34900 -3.30663 2.04134
H 4.40399 -1.54087 2.29446
H 5.83731 -2.41059 1.66348
C -1.58000 3.39817 2.02631
H -1.96237 2.36324 1.99918
C 3.56849 0.86639 5.40232
H 4.17518 0.97410 6.30728
C -0.26188 4.54048 -1.94926
H 0.03498 4.87960 -2.94920
C -3.38693 -0.14475 3.38081
H -2.72298 -0.80400 3.96436
H -4.24720 0.12260 4.02020
H -2.83146 0.78304 3.16322
C -3.03834 -2.39588 -3.19469
H -2.92891 -3.39763 -3.64792
H -2.03020 -1.96116 -3.09337
H -3.61522 -1.76997 -3.89915
C 6.52999 2.02308 -1.58008
H 6.42460 2.20136 -2.66490
H 7.55340 1.65464 -1.40870
H 6.44365 2.99795 -1.07300
C -0.47069 3.48104 3.09476
H 0.41250 2.89047 2.81015
H -0.84493 3.09730 4.06028
H -0.14646 4.52291 3.27004
C -4.50542 3.97389 -0.81337
H -4.98040 4.37442 0.09841
H -5.22184 4.12245 -1.64045
H -3.61408 4.58552 -1.02115
C -0.38536 2.41657 -4.10499
H 0.07141 3.33835 -4.50798
H -0.75613 1.83354 -4.96623
H 0.41260 1.83049 -3.62144
C 4.14600 1.05704 4.13636
H 5.20684 1.31627 4.05106
C 3.72568 3.60382 -0.15977
H 4.73651 4.02416 -0.29573
H 3.63776 3.21837 0.86831
H 2.99634 4.42216 -0.27556
C -2.57974 3.61275 -3.88563
H -3.45739 3.84551 -3.26458
H -2.93107 3.10453 -4.80188
H -2.12416 4.57264 -4.18932
C 4.62833 -3.28769 -0.75371
H 5.72203 -3.42065 -0.78319
H 4.25600 -3.10131 -1.77307
H 4.18911 -4.23733 -0.40749
C -5.18798 -3.02202 -1.99116
H -5.76907 -2.38303 -2.67985
H -5.72236 -3.05652 -1.02651
H -5.18893 -4.04464 -2.40944
C -2.74923 4.32540 2.43547
H -2.42045 5.38038 2.45392
H -3.11450 4.06782 3.44605
H -3.59575 4.25291 1.73644
C 3.52621 2.98685 -2.64127
H 2.76669 3.76526 -2.81948
H 3.34191 2.16237 -3.34922
H 4.51433 3.42447 -2.85842
C -0.26348 4.84664 0.43917
H 0.03913 5.42240 1.32110
C 7.04918 -1.04314 -0.67629
H 7.21610 -1.58265 0.27017
H 7.89122 -0.34573 -0.80768
H 7.10521 -1.78187 -1.49517
C 0.15938 5.26372 -0.82834
H 0.78377 6.15730 -0.94246
C 1.18136 -2.06656 -2.45673
C 2.06217 -2.38241 -3.52171
H 2.49272 -1.56434 -4.11241
C 0.87719 -0.63664 -2.22142
C 0.62813 -3.13277 -1.71176
H -0.06921 -2.92321 -0.89577
C -0.12464 -0.01825 -1.48438
C 0.94897 -4.46201 -2.02367
H 0.49768 -5.26518 -1.43366
C 1.81458 -4.75948 -3.09045
H 2.05006 -5.80057 -3.33619
C 2.36986 -3.71139 -3.84387
H 3.03998 -3.92867 -4.68296
H -0.06896 1.07148 -1.69738

TS(Z-P)$_{Ph}$

SCF (BP86) Energy = -2956.23659084
Enthalpy 0K = -2954.910771
Enthalpy 298K = -2954.823523
Free Energy 298K = -2955.034558
Lowest Frequency = -870.5779 cm^{-1}
Second Frequency = 11.9700 cm^{-1}
SCF (BP86-D3BJ) Energy = -2956.71547481
SCF (C6H6) Energy = -2956.24290349
SCF (BS2) Energy = -3768.58141851

Cu -1.23194 0.68126 -0.18891

Si	4.64102	-1.28385	-1.13348
Al	1.72588	-0.22875	0.14230
Si	3.79245	-0.71915	2.65210
N	-3.53631	1.89354	-1.71479
N	2.83893	-1.16610	-1.07285
N	-4.20522	1.21821	0.23918
N	2.61146	0.28598	1.74960
C	-3.09127	1.28050	-0.56530
H	-1.88830	-2.08340	0.85590
C	2.35595	0.21428	-3.67572
H	2.68858	0.62907	-2.70874
C	2.06299	-1.27103	-3.44566
C	2.39268	1.60354	2.31476
C	1.88717	-3.28026	-2.02250
C	5.42534	-1.08325	1.72047
H	5.90178	-0.11435	1.47700
H	6.07895	-1.53530	2.49652
C	-0.42852	3.83717	-0.53729
C	5.38705	-1.99572	0.47765
H	4.86323	-2.94304	0.70743
H	6.41962	-2.29344	0.19837
C	2.23817	-1.89513	-2.16563
C	3.02618	-2.40105	3.14234
H	2.30388	-2.27180	3.96710
H	3.80871	-3.09664	3.49407
H	2.50153	-2.88809	2.30302
C	1.32411	-3.40099	-4.40842
H	0.99816	-3.98797	-5.27412
C	-4.15321	0.64049	1.60706
H	-3.15054	0.18038	1.64830
C	1.42506	1.80955	3.35170
C	5.17376	-2.46387	-2.54044
H	4.91380	-2.05900	-3.53197
H	4.73066	-3.46853	-2.47626
H	6.27302	-2.56898	-2.49837
C	-2.64983	2.10201	-2.89199
H	-1.65272	1.83800	-2.49894
C	1.44532	-4.00040	-3.14943
H	1.19882	-5.06258	-3.03626
C	-1.53098	4.33066	0.20223
H	-2.07480	3.64365	0.85608
C	0.51810	0.68760	3.84911
H	0.82800	-0.22562	3.31629
C	-4.90016	2.20772	-1.63171
C	0.01013	2.46844	-0.42560
C	1.08269	0.97971	-4.10057
H	0.69220	0.60905	-5.06578
H	1.29848	2.05700	-4.21793
H	0.28824	0.87046	-3.34413
C	-5.32453	1.78552	-0.38900
C	3.47851	0.44295	-4.71314
H	4.41895	-0.04764	-4.41421
H	3.68303	1.52218	-4.83212
H	3.19592	0.04766	-5.70541
C	1.61786	-2.03951	-4.54104
H	1.51360	-1.55794	-5.52116
C	1.99497	-4.02397	-0.68754
H	2.22639	-3.26382	0.07936
C	0.27645	4.73054	-1.38523
H	1.13144	4.35741	-1.95590
C	0.76723	1.45771	-0.38692
C	3.16361	2.73308	1.88825
C	-0.11469	6.07147	-1.48567
H	0.44160	6.74775	-2.14329
C	-2.60600	3.56740	-3.35727
H	-1.73472	3.70009	-4.01917
H	-2.49373	4.25933	-2.50871
H	-3.50064	3.84798	-3.93629
C	4.23936	2.62433	0.81244
H	4.24905	1.57258	0.48478
C	-1.21016	6.55013	-0.74644
H	-1.51119	7.59987	-0.82543
C	1.25667	3.08808	3.91802
H	0.51319	3.22182	4.71351
C	5.55321	0.35372	-1.54020
H	4.93302	1.04060	-2.13736
H	6.45198	0.11333	-2.13583
H	5.88960	0.89432	-0.64255
C	0.67141	-4.71990	-0.29815
H	0.43263	-5.54856	-0.98890
H	-0.17347	-4.01555	-0.30673
H	0.75035	-5.14976	0.71586
C	-6.70290	1.87725	0.19387
H	-7.20535	0.89566	0.24512
H	-7.32640	2.53355	-0.43250
H	-6.70510	2.30471	1.20995
C	3.91953	3.49815	-0.41987
H	2.94995	3.21101	-0.85856
H	4.69979	3.38127	-1.19281
H	3.86782	4.56922	-0.15407
C	4.36281	0.17023	4.23949
H	4.94372	1.07675	4.00135
H	5.01438	-0.50725	4.81937
H	3.52825	0.48112	4.88559
C	-0.95006	0.98150	3.47876
H	-1.31274	1.88919	3.99372
H	-1.60262	0.13651	3.76076
H	-1.05232	1.14787	2.38987
C	-1.91352	5.67449	0.09863
H	-2.76376	6.04139	0.68344
C	-4.22846	1.73440	2.68538
H	-5.21034	2.23642	2.69947
H	-3.44795	2.49613	2.53250
H	-4.07009	1.28266	3.67811
C	0.62755	0.42654	5.36793
H	1.65367	0.16006	5.66945
H	-0.03805	-0.40419	5.66513
H	0.32883	1.31346	5.95485
C	-2.98836	1.13180	-4.03466
H	-3.95829	1.36698	-4.50464
H	-3.00831	0.09024	-3.68138
H	-2.21454	1.21302	-4.81628
C	3.13521	-5.06966	-0.68029
H	3.16762	-5.59669	0.29011
H	4.12309	-4.61290	-0.84798
H	2.97820	-5.82943	-1.46726
C	5.64088	2.96950	1.36558
H	5.69224	4.02047	1.70215
H	6.41438	2.83411	0.58788
H	5.90732	2.33323	2.22654
C	-5.19562	-0.46978	1.81812
H	-4.92029	-1.06399	2.70398
H	-5.22631	-1.15366	0.95530
H	-6.20829	-0.07024	1.98721
C	2.95335	3.99446	2.47898
H	3.55476	4.84512	2.13631
C	-5.70518	2.88737	-2.69814
H	-5.42212	3.94627	-2.82726
H	-6.77267	2.86110	-2.43076
H	-5.60566	2.39398	-3.67878
C	2.01130	4.18550	3.49328
H	1.86853	5.17257	3.94643
C	-2.01867	-1.99367	-1.33112
C	-3.34691	-2.47731	-1.23300
H	-3.82113	-2.52518	-0.24698
C	-1.30688	-1.60265	-0.09359
C	-1.40351	-1.97600	-2.60410
H	-0.36817	-1.63925	-2.69866
C	-0.09782	-1.16665	0.29114
C	-2.09974	-2.42747	-3.73467
H	-1.59053	-2.42563	-4.70346
C	-3.42122	-2.89356	-3.62657
H	-3.95663	-3.24847	-4.51366
C	-4.04388	-2.91517	-2.36697
H	-5.06905	-3.28764	-2.26484
H	-0.18392	-1.55492	1.64032
C	-1.82123	-2.75877	2.36448
C	-0.66814	-2.28311	2.63883
H	0.09845	-2.36450	3.40687
C	-3.00677	-3.50799	2.69505
C	-3.70033	-3.24980	3.90932
C	-3.50358	-4.52696	1.84069
C	-4.84274	-3.98572	4.25002
C	-4.63918	-5.26573	2.19667

```
C  -5.31818 -4.99748  3.39781
H  -3.31697 -2.47545  4.58146
H  -2.97981 -4.73488  0.90272
H  -5.35952 -3.77427  5.19255
H  -4.99866 -6.05606  1.52894
H  -6.20874 -5.57357  3.66903
```

TS(I-II)$_{Bu}$

SCF (BP86) Energy = -2265.61309437
Enthalpy 0K = -2264.470528
Enthalpy 298K = -2264.396458
Free Energy 298K = -2264.580033
Lowest Frequency = -85.8570 cm^{-1}
Second Frequency = 11.8584 cm^{-1}
SCF (BP86-D3BJ) Energy = -2265.97508653
SCF (C6H6) Energy = -2265.61716629
SCF (BS2) Energy = -3077.79282014

```
Cu  1.29949 -0.08776  0.38018
Si -3.66925  2.22595 -0.38403
Si -3.87011 -1.27056  1.36669
Al -1.09754  0.12098  0.22052
N  -1.95381  1.81071 -0.00851
N  -2.36467 -1.30036  0.39193
N   3.06907 -1.28016 -1.72313
N   3.95334  0.57212 -1.01222
C  -1.05269  2.93738 -0.05542
C  -0.65575  3.60737  1.15036
C   0.13651  4.76999  1.06599
H   0.41661  5.29278  1.98689
C   0.56958  5.27864 -0.16359
H   1.16925  6.19461 -0.20539
C   0.23646  4.59176 -1.33586
H   0.59447  4.96942 -2.30124
C  -0.55496  3.42683 -1.30859
C  -1.07299  3.10021  2.53464
H  -1.26684  2.01390  2.42571
C   0.02067  3.30078  3.60836
H   1.01060  2.95761  3.26553
H  -0.24130  2.74699  4.52736
H   0.11670  4.36302  3.89572
C  -2.38668  3.74294  3.03659
H  -2.28014  4.84109  3.09623
H  -2.64101  3.37222  4.04597
H  -3.23330  3.51977  2.37115
C  -0.84283  2.71307 -2.63196
H  -1.56811  1.91183 -2.41507
C   0.42977  2.03655 -3.19141
H   1.20681  2.78499 -3.43206
H   0.19971  1.48402 -4.12077
H   0.85216  1.32563 -2.45955
C  -1.46369  3.64689 -3.69405
H  -2.37729  4.14078 -3.32232
H  -1.72863  3.07699 -4.60249
H  -0.75973  4.44137 -4.00035
C  -3.91710  4.12022 -0.37926
H  -4.96036  4.33990 -0.66882
H  -3.25355  4.61243 -1.10886
H  -3.73082  4.58833  0.59947
C  -4.26828  1.66298 -2.11221
H  -3.82668  2.27492 -2.91513
H  -5.36545  1.78743 -2.16559
H  -4.04249  0.60759 -2.32689
C  -4.89570  1.47130  0.87508
H  -4.56246  1.70975  1.90227
H  -5.83085  2.05116  0.72825
C  -5.18978 -0.03867  0.73520
H  -5.43569 -0.29338 -0.31339
H  -6.09911 -0.29975  1.31697
C  -3.48780 -0.78208  3.17549
H  -2.87556 -1.54447  3.68460
H  -4.42063 -0.65400  3.75330
H  -2.93856  0.17499  3.21924
C  -4.72178 -2.97649  1.32090
H  -5.15885 -3.16631  0.32572
H  -5.54338 -3.00100  2.05827
H  -4.03634 -3.80916  1.54158
C  -1.95820 -2.58023 -0.13361
C  -1.19659 -3.51223  0.64621
C  -0.84131 -4.75850  0.08963
H  -0.27481 -5.46951  0.70317
C  -1.20047 -5.11569 -1.21469
H  -0.92105 -6.09352 -1.62240
C  -1.93704 -4.20609 -1.98292
H  -2.23390 -4.47742 -3.00316
C  -2.32820 -2.95532 -1.46792
C  -0.74857 -3.21691  2.07824
H  -1.03361 -2.17385  2.29865
C   0.78379 -3.32065  2.22374
H   1.14444 -4.34624  2.02571
H   1.08800 -3.04790  3.24908
H   1.29096 -2.62988  1.52825
C  -1.43707 -4.13922  3.11101
H  -2.53451 -4.04270  3.08400
H  -1.10038 -3.89433  4.13455
H  -1.19142 -5.20026  2.92467
C  -3.15161 -2.02467 -2.35470
H  -3.47969 -1.19733 -1.70355
C  -2.29177 -1.41951 -3.48670
H  -1.42826 -0.86524 -3.07878
H  -2.88467 -0.72100 -4.10395
H  -1.89895 -2.20902 -4.15266
C  -4.41171 -2.70463 -2.93232
H  -4.15680 -3.51466 -3.63898
H  -5.02649 -1.97081 -3.48316
H  -5.03561 -3.14323 -2.13528
C   2.91383 -0.29954 -0.77066
C   2.13546 -2.43610 -1.77237
H   1.34374 -2.13937 -1.05694
C   1.47315 -2.60979 -3.14687
H   1.08615 -1.64856 -3.52135
H   0.62030 -3.29946 -3.03805
H   2.15838 -3.03511 -3.89847
C   2.79042 -3.72612 -1.25457
H   3.25397 -3.56176 -0.26857
H   3.56194 -4.11104 -1.94369
H   2.01420 -4.50129 -1.14553
C   4.17513 -1.02924 -2.54777
C   4.62590 -1.89782 -3.68339
H   4.72142 -2.95576 -3.38720
H   5.61594 -1.56942 -4.03608
H   3.93688 -1.85596 -4.54507
C   4.73140  0.15445 -2.10455
C   5.92537  0.87716 -2.65205
H   6.79989  0.82730 -1.97955
H   5.71564  1.94270 -2.84559
H   6.22336  0.42794 -3.61211
C   4.10262  1.81630 -0.21366
H   3.42358  1.64300  0.64033
C   5.52802  2.00774  0.33346
H   5.92885  1.07439  0.76161
H   5.50530  2.76902  1.13113
H   6.22966  2.36717 -0.43602
C   3.60090  3.04848 -0.98098
H   4.22294  3.26226 -1.86749
H   3.63498  3.93290 -0.32352
H   2.55762  2.91250 -1.30177
C   2.63315 -0.24673  2.95372
C   1.53884  0.24603  2.64148
H   0.62051  0.76352  2.85427
C   3.91539 -0.83296  3.39208
C   3.64584 -1.72452  4.63825
H   3.18762 -1.13957  5.45248
H   2.97230 -2.56004  4.38964
H   4.59960 -2.14376  5.00473
C   4.88249  0.31330  3.79824
H   5.83932 -0.11188  4.14931
H   5.09077  0.98011  2.94717
H   4.45297  0.91974  4.61256
C   4.55603 -1.69675  2.27461
H   3.89363 -2.53474  2.00360
H   4.73674 -1.10146  1.36598
H   5.51682 -2.11081  2.62873
```

II$_{Bu}$
SCF (BP86) Energy = -2265.62975510
Enthalpy 0K = -2264.485145
Enthalpy 298K = -2264.411641
Free Energy 298K = -2264.592417
Lowest Frequency = 12.7154 cm^{-1}
Second Frequency = 15.3565 cm^{-1}
SCF (BP86-D3BJ) Energy = -2265.99947866
SCF (C6H6) Energy = -2265.63320952
SCF (BS2) Energy = -3077.81150290

```
Cu  1.36791 -0.35230  0.63757
Si -2.92502  2.65256 -1.09430
Si -3.94421 -0.48630  1.03038
Al -0.89158  0.23260  0.02666
N  -1.36966  2.00040 -0.44702
N  -2.37344 -0.92704  0.27190
N   2.87156 -2.01567 -1.30216
N   3.99589 -0.24050 -0.72342
C  -0.29460  2.96963 -0.45588
C  -0.03862  3.78005  0.69908
C   0.97038  4.76225  0.64139
H   1.15145  5.38974  1.52194
C   1.73676  4.96431 -0.51141
H   2.50796  5.74206 -0.53677
C   1.49708  4.16395 -1.63406
H   2.08910  4.32203 -2.54387
C   0.49746  3.17130 -1.63411
C  -0.83036  3.63350  2.00384
H  -1.44681  2.72199  1.90235
C   0.09753  3.45745  3.22600
H   0.80017  2.62529  3.06917
H  -0.49772  3.24644  4.13266
H   0.68249  4.37277  3.42702
C  -1.78601  4.82227  2.26242
H  -1.23038  5.77670  2.29099
H  -2.29404  4.70183  3.23606
H  -2.56422  4.90897  1.48848
C   0.27858  2.36422 -2.91744
H  -0.57002  1.68466 -2.72996
C   1.50060  1.48784 -3.26926
H   2.38954  2.10778 -3.48772
H   1.29428  0.88098 -4.16968
H   1.74842  0.80515 -2.43977
C  -0.08558  3.26631 -4.11906
H  -0.97316  3.88791 -3.91497
H  -0.29705  2.65316 -5.01345
H   0.74391  3.94914 -4.37613
C  -2.79271  4.53387 -1.38655
H  -2.57193  5.11510 -0.47942
H  -3.75494  4.89060 -1.79559
H  -2.00640  4.77038 -2.12166
C  -3.44046  1.96082 -2.80030
H  -2.71862  2.24234 -3.58390
H  -4.41613  2.40271 -3.07417
H  -3.55561  0.86745 -2.82062
C  -4.37325  2.32642  0.10778
H  -4.07562  2.65051  1.12289
H  -5.17163  3.03150 -0.20349
C  -4.92867  0.88537  0.12785
H  -5.14199  0.53783 -0.90116
H  -5.91219  0.86979  0.64412
C  -3.67586  0.12475  2.82134
H  -3.24580 -0.65961  3.46549
H  -4.63469  0.44300  3.26811
H  -2.99913  0.99747  2.85214
C  -5.12263 -1.98601  1.01352
H  -5.50814 -2.15814 -0.00601
H  -5.98867 -1.78329  1.66777
H  -4.64988 -2.92179  1.34681
C  -2.20758 -2.31692 -0.09370
C  -1.78146 -3.30466  0.85554
C  -1.67167 -4.65210  0.45204
H  -1.36344 -5.40069  1.19147
C  -1.95472 -5.06090 -0.85541
H  -1.86795 -6.11486 -1.14144
C  -2.36671 -4.10006 -1.78569
H  -2.60601 -4.40803 -2.81052
C  -2.50896 -2.74518 -1.42940
C  -1.45096 -2.97410  2.31288
H  -1.47500 -1.87589  2.41133
C  -0.03350 -3.44586  2.70049
H   0.06778 -4.54326  2.62540
H   0.18825 -3.16655  3.74564
H   0.73175 -2.97988  2.05903
C  -2.48114 -3.57591  3.29805
H  -3.50228 -3.20852  3.10896
H  -2.21595 -3.31715  4.33893
H  -2.50442 -4.67812  3.22534
C  -3.00583 -1.76312 -2.48625
H  -3.20893 -0.82186 -1.94966
C  -1.92329 -1.47229 -3.54905
H  -1.00672 -1.06648 -3.08526
H  -2.28450 -0.73607 -4.28887
H  -1.64492 -2.39207 -4.09428
C  -4.31837 -2.21977 -3.15937
H  -4.17514 -3.13068 -3.76764
H  -4.69813 -1.43228 -3.83439
H  -5.10125 -2.43770 -2.41349
C   2.79923 -0.89040 -0.51563
C   1.78192 -3.02444 -1.29800
H   0.95854 -2.49531 -0.78069
C   1.29177 -3.38540 -2.70825
H   1.12802 -2.48066 -3.31532
H   0.32722 -3.90992 -2.61554
H   1.98782 -4.05143 -3.24318
C   2.16250 -4.25818 -0.46347
H   2.50410 -3.96138  0.54101
H   2.95950 -4.85338 -0.94220
H   1.27562 -4.90279 -0.34970
C   4.09246 -2.07422 -1.99124
C   4.49710 -3.16108 -2.94037
H   4.34070 -4.16562 -2.51366
H   5.56909 -3.07093 -3.17544
H   3.94527 -3.11602 -3.89570
C   4.80074 -0.94536 -1.63373
C   6.13212 -0.49122 -2.15066
H   6.85210 -0.26555 -1.34693
H   6.04733  0.40971 -2.78347
H   6.57698 -1.28315 -2.77290
C   4.36146  0.97405  0.05031
H   3.45633  1.16068  0.65656
C   5.52531  0.69452  1.01727
H   5.32198 -0.19732  1.63124
H   5.65289  1.55695  1.69275
H   6.48368  0.54787  0.49249
C   4.60926  2.20058 -0.83938
H   5.53933  2.11313 -1.42498
H   4.70081  3.09425 -0.20033
H   3.76448  2.36762 -1.52384
C   1.51950 -0.14509  2.63321
C   0.32103  0.14309  2.19196
H  -0.65692  0.40668  2.61375
C   2.26417 -0.25546  3.94479
C   3.18477  0.98161  4.10897
H   3.89644  1.05798  3.27162
H   2.59999  1.91522  4.14887
H   3.76321  0.89882  5.04694
C   1.27019 -0.30403  5.13396
H   1.80964 -0.38517  6.09538
H   0.65166  0.60904  5.16533
H   0.59375 -1.17111  5.04721
C   3.14171 -1.53101  3.94832
H   3.72132 -1.59876  4.88692
H   2.52330 -2.43938  3.85998
H   3.84585 -1.51974  3.09934
```

TS(II-III)$_{Bu}$
SCF (BP86) Energy = -2265.62518328
Enthalpy 0K = -2264.481085
Enthalpy 298K = -2264.408541
Free Energy 298K = -2264.585769
Lowest Frequency = -140.5798 cm^{-1}
Second Frequency = 18.3386 cm^{-1}

SCF (BP86-D3BJ) Energy = -2265.99276069
SCF (C6H6) Energy = -2265.62889450
SCF (BS2) Energy = -3077.80464524

Cu 1.59167 -0.21821 0.82799
Si -2.99949 2.31452 -1.53773
Si -4.08863 -0.84871 0.58142
Al -1.06959 0.26775 0.23683
N -1.49875 1.88047 -0.62229
N -2.35615 -1.10405 0.16216
N 3.22946 -1.94489 -0.93112
N 4.13011 0.00621 -0.55381
C -0.46912 2.90403 -0.58159
C -0.42458 3.85808 0.48724
C 0.63643 4.78357 0.53796
H 0.67615 5.49808 1.36833
C 1.62325 4.82887 -0.45249
H 2.43350 5.56423 -0.39623
C 1.53395 3.94972 -1.53717
H 2.27186 4.01395 -2.34549
C 0.50364 2.99282 -1.62976
C -1.51621 3.96238 1.55687
H -2.29586 3.22450 1.29684
C -0.99430 3.62431 2.96957
H -0.54244 2.62217 2.99276
H -1.81550 3.66377 3.70796
H -0.22648 4.35227 3.28905
C -2.16214 5.36885 1.58230
H -1.44676 6.12759 1.94650
H -3.02825 5.38064 2.26756
H -2.50436 5.69168 0.58664
C 0.41606 2.14703 -2.90318
H -0.42466 1.44573 -2.77055
C 1.68016 1.30525 -3.17059
H 2.56069 1.94685 -3.35733
H 1.53992 0.67707 -4.06865
H 1.90967 0.64670 -2.31719
C 0.11243 3.03994 -4.13034
H -0.79377 3.65041 -3.98193
H -0.03136 2.42376 -5.03621
H 0.94761 3.73506 -4.32990
C -3.08308 4.19907 -1.81463
H -3.46156 4.72442 -0.92376
H -3.78461 4.40103 -2.64317
H -2.10806 4.63802 -2.07920
C -3.14359 1.54546 -3.28150
H -2.42830 1.99866 -3.98579
H -4.16039 1.75045 -3.66402
H -2.99542 0.45608 -3.29959
C -4.58486 1.83678 -0.58459
H -4.51386 2.22400 0.44988
H -5.37422 2.45021 -1.06755
C -5.00240 0.35053 -0.59341
H -4.95634 -0.06175 -1.61951
H -6.06717 0.25710 -0.29213
C -4.23571 -0.12388 2.34219
H -3.89688 -0.84160 3.10724
H -5.28430 0.14154 2.56619
H -3.63376 0.79499 2.45746
C -5.05057 -2.48775 0.45551
H -5.16963 -2.78862 -0.59900
H -6.06005 -2.35135 0.88155
H -4.56420 -3.32262 0.98155
C -1.96863 -2.47369 -0.10135
C -1.63703 -3.38093 0.95877
C -1.33965 -4.72524 0.65173
H -1.10634 -5.41398 1.47229
C -1.34987 -5.20611 -0.66173
H -1.12929 -6.25839 -0.87209
C -1.66083 -4.32042 -1.69962
H -1.68281 -4.68530 -2.73354
C -1.97343 -2.97094 -1.44631
C -1.59971 -2.96922 2.43226
H -1.78708 -1.88300 2.47309
C -0.21155 -3.23132 3.05503
H 0.04234 -4.30634 3.04345
H -0.19534 -2.90108 4.10870
H 0.57600 -2.68094 2.51543
C -2.68774 -3.67873 3.27246
H -3.70432 -3.46195 2.90664
H -2.63150 -3.35336 4.32659
H -2.55459 -4.77528 3.25664
C -2.33341 -2.07441 -2.62709
H -2.66304 -1.11792 -2.18972
C -1.10608 -1.78843 -3.52069
H -0.28754 -1.32488 -2.94287
H -1.37086 -1.10261 -4.34518
H -0.71593 -2.71908 -3.97049
C -3.49750 -2.63778 -3.47131
H -3.22065 -3.58165 -3.97394
H -3.78631 -1.91886 -4.25861
H -4.38708 -2.84010 -2.85137
C 2.96923 -0.71678 -0.36499
C 2.23200 -3.04298 -0.87689
H 1.30407 -2.52268 -0.57477
C 1.98513 -3.69605 -2.24550
H 1.85052 -2.93507 -3.03100
H 1.05759 -4.28705 -2.18408
H 2.79913 -4.37540 -2.54487
C 2.58128 -4.06283 0.21962
H 2.73643 -3.55624 1.18574
H 3.49078 -4.63873 -0.02453
H 1.74545 -4.77259 0.33198
C 4.53773 -2.00208 -1.43690
C 5.15213 -3.18659 -2.11894
H 5.01198 -4.11780 -1.54527
H 6.23698 -3.03136 -2.22743
H 4.74185 -3.35398 -3.13044
C 5.10512 -0.76620 -1.20042
C 6.47213 -0.28739 -1.58512
H 7.00969 0.17693 -0.74155
H 6.44255 0.44974 -2.40677
H 7.08002 -1.13782 -1.93108
C 4.30079 1.35241 0.05479
H 3.27083 1.62840 0.34512
C 5.14248 1.26534 1.33842
H 4.68971 0.54667 2.03994
H 5.17360 2.25334 1.82770
H 6.18263 0.95873 1.13380
C 4.82207 2.40669 -0.93330
H 5.90120 2.30614 -1.13222
H 4.65474 3.40662 -0.50112
H 4.27748 2.35940 -1.88894
C 1.11377 0.33724 2.60981
C -0.10769 0.30063 2.02830
H -1.06194 0.20433 2.61238
C 1.46389 0.41683 4.08740
C 2.20655 1.75968 4.32322
H 3.09581 1.82873 3.67503
H 1.55759 2.62283 4.10179
H 2.53341 1.83534 5.37682
C 0.23014 0.34698 5.01940
H 0.53167 0.40848 6.08049
H -0.47100 1.17684 4.82369
H -0.32112 -0.59965 4.88066
C 2.43710 -0.73647 4.43677
H 2.78351 -0.64723 5.48310
H 1.95200 -1.71976 4.31973
H 3.31644 -0.71337 3.77070

III$_{Bu}$
SCF (BP86) Energy = -2265.65978926
Enthalpy 0K = -2264.515317
Enthalpy 298K = -2264.441832
Free Energy 298K = -2264.625192
Lowest Frequency = 8.9551 cm^{-1}
Second Frequency = 16.5029 cm^{-1}
SCF (BP86-D3BJ) Energy = -2266.01550161
SCF (C6H6) Energy = -2265.66466563
SCF (BS2) Energy = -3077.83887587

Cu 2.21377 0.67212 0.83659
Si -3.85173 0.49955 -2.16655
Si -2.79603 -3.10500 -0.99252

Al -1.57686 -0.27098 0.11110
N -2.97591 0.72463 -0.61154
N -1.44674 -2.04115 -0.43355
N 5.01516 -0.08916 0.15114
N 4.32011 1.58551 -1.04691
C -3.32393 1.90226 0.16161
C -4.29467 1.80476 1.20821
C -4.60405 2.94523 1.97400
H -5.34032 2.86074 2.78183
C -3.99750 4.18072 1.72345
H -4.25287 5.05797 2.32757
C -3.06261 4.27994 0.68772
H -2.58644 5.24657 0.48458
C -2.71008 3.16689 -0.10055
C -4.99953 0.48768 1.53674
H -4.72975 -0.22361 0.73653
C -4.50167 -0.09865 2.87825
H -3.40909 -0.25647 2.87350
H -4.98726 -1.06812 3.09020
H -4.72976 0.58406 3.71608
C -6.53819 0.62199 1.54550
H -6.88686 1.28533 2.35675
H -7.01043 -0.36405 1.70205
H -6.91526 1.03471 0.59448
C -1.67329 3.36324 -1.20826
H -1.51677 2.37966 -1.68317
C -0.31299 3.82641 -0.64264
H -0.39616 4.80816 -0.14260
H 0.42257 3.93612 -1.46157
H 0.07677 3.09888 0.08848
C -2.16664 4.34728 -2.29387
H -3.11435 4.01622 -2.75054
H -1.41706 4.44591 -3.10003
H -2.33569 5.35477 -1.87367
C -5.26417 1.76882 -2.29422
H -6.05196 1.56390 -1.55025
H -5.72240 1.71117 -3.29711
H -4.91942 2.80199 -2.13181
C -2.68397 0.70740 -3.66448
H -2.35066 1.75165 -3.78023
H -3.19964 0.41253 -4.59597
H -1.78481 0.07547 -3.56667
C -4.65882 -1.22440 -2.32814
H -5.32881 -1.38607 -1.46239
H -5.33467 -1.12730 -3.20355
C -3.71871 -2.43360 -2.52632
H -2.98432 -2.23369 -3.32985
H -4.30322 -3.30810 -2.88212
C -4.09852 -3.36620 0.37755
H -3.69357 -3.95678 1.21504
H -4.97208 -3.90833 -0.02700
H -4.46169 -2.40865 0.78681
C -2.08747 -4.79828 -1.49902
H -1.42375 -4.71310 -2.37558
H -2.91887 -5.47383 -1.76686
H -1.50780 -5.27091 -0.69057
C -0.17653 -2.71322 -0.25989
C 0.14896 -3.39013 0.95610
C 1.36984 -4.08919 1.04376
H 1.61126 -4.61805 1.97339
C 2.26641 -4.14001 -0.02825
H 3.19510 -4.71510 0.05306
C 1.95557 -3.45957 -1.21273
H 2.65358 -3.50057 -2.05643
C 0.75048 -2.74648 -1.35002
C -0.78253 -3.40516 2.16907
H -1.67336 -2.81095 1.90210
C -0.12727 -2.73608 3.39760
H 0.77364 -3.28769 3.72250
H -0.83118 -2.71909 4.24906
H 0.16381 -1.69987 3.16250
C -1.25033 -4.83650 2.52037
H -1.74602 -5.33097 1.66779
H -1.96271 -4.81455 3.36432
H -0.39983 -5.47334 2.82289
C 0.44918 -2.00461 -2.65202
H -0.64714 -1.88133 -2.69855
C 1.06727 -0.58812 -2.61909
H 0.73390 -0.01628 -1.73197
H 0.79616 -0.01169 -3.52238
H 2.16848 -0.64748 -2.55700
C 0.89343 -2.75718 -3.92239
H 1.99307 -2.82608 -4.00308
H 0.53654 -2.22797 -4.82326
H 0.49077 -3.78394 -3.94758
C 3.90359 0.67946 -0.09959
C 4.98082 -1.14349 1.19912
H 3.90168 -1.22530 1.42711
C 5.46279 -2.50935 0.68880
H 4.96364 -2.77367 -0.25635
H 5.20035 -3.27851 1.43344
H 6.55448 -2.54821 0.54469
C 5.70773 -0.68423 2.47431
H 5.30827 0.28131 2.82391
H 6.79493 -0.57735 2.31906
H 5.55610 -1.42861 3.27374
C 6.11319 0.33449 -0.61239
C 7.47393 -0.29345 -0.60080
H 7.86268 -0.43383 0.42141
H 8.18770 0.35338 -1.13455
H 7.48787 -1.27831 -1.10031
C 5.67055 1.39797 -1.37571
C 6.43196 2.18747 -2.39686
H 6.31992 3.27541 -2.25784
H 6.11930 1.95019 -3.42931
H 7.50638 1.95817 -2.32145
C 3.40542 2.65971 -1.51341
H 2.43881 2.36604 -1.06243
C 3.79605 4.02651 -0.92696
H 3.87764 3.96807 0.17019
H 3.01641 4.76650 -1.17282
H 4.75163 4.40097 -1.33143
C 3.23629 2.67939 -3.04036
H 4.12872 3.06252 -3.56122
H 2.39522 3.34426 -3.29854
H 3.00459 1.67371 -3.42587
C 0.64332 0.90446 1.92675
C -0.64765 0.56961 1.60334
H -1.43058 0.87839 2.33210
C 0.93220 1.63677 3.27529
C 1.55603 3.02089 2.96042
H 2.45478 2.90706 2.32529
H 0.84035 3.66417 2.42005
H 1.85396 3.54131 3.89056
C -0.30086 1.85883 4.18048
H -0.00054 2.36825 5.11426
H -1.06385 2.48672 3.68880
H -0.77752 0.90313 4.46075
C 1.97307 0.80814 4.07049
H 2.26893 1.33088 4.99992
H 1.57022 -0.18184 4.34628
H 2.88168 0.64154 3.46229

TS(I-III)$_{Bu}$

SCF (BP86) Energy = -2265.59027108
Enthalpy 0K = -2264.446778
Enthalpy 298K = -2264.374017
Free Energy 298K = -2264.550541
Lowest Frequency = -228.6282 cm^{-1}
Second Frequency = 12.2951 cm^{-1}
SCF (BP86-D3BJ) Energy = -2265.96821032
SCF (C6H6) Energy = -2265.59438757
SCF (BS2) Energy = -3077.76728717

Cu 1.33975 0.28842 0.17900
Si -3.65065 0.90911 -1.61112
Si -3.32484 -2.57787 0.25288
Al -1.09157 -0.10810 0.30956
N -2.24844 1.20632 -0.54122
N -1.65108 -1.94461 0.08408
N 4.13994 -0.67768 -0.35343
N 4.15374 1.48774 -0.19445
C -1.77060 2.55984 -0.42041
C -2.26743 3.44151 0.60137

```
C  -1.68203  4.71429  0.76667
H  -2.06285  5.37296  1.55672
C  -0.64953  5.16772 -0.06030
H  -0.20991  6.16016  0.08984
C  -0.22948  4.35242 -1.11811
H   0.52234  4.72939 -1.82276
C  -0.78077  3.07326 -1.32876
C  -3.48217  3.10685  1.47530
H  -3.81708  2.09572  1.18319
C  -3.17162  3.09461  2.98780
H  -2.40563  2.34621  3.23510
H  -4.08281  2.85815  3.56683
H  -2.81424  4.08301  3.32980
C  -4.63741  4.10990  1.22961
H  -4.37959  5.10942  1.62385
H  -5.55389  3.77827  1.75015
H  -4.86970  4.22770  0.16101
C  -0.40399  2.32596 -2.60973
H  -0.84283  1.31695 -2.53444
C   1.11215  2.16099 -2.82172
H   1.61775  3.13783 -2.92861
H   1.31192  1.58506 -3.74297
H   1.57255  1.61729 -1.97631
C  -1.02231  3.04240 -3.83561
H  -2.11186  3.17236 -3.72947
H  -0.83156  2.47159 -4.76261
H  -0.58203  4.04789 -3.96421
C  -4.60189  2.52915 -1.96619
H  -5.35350  2.73440 -1.18743
H  -5.13854  2.42401 -2.92544
H  -3.94048  3.40743 -2.04204
C  -3.25842  0.20390 -3.34942
H  -2.74445  0.94033 -3.98721
H  -4.21671 -0.04733 -3.84116
H  -2.64953 -0.71179 -3.32881
C  -4.91009 -0.30131 -0.83332
H  -5.08832 -0.01217  0.22035
H  -5.86205 -0.07801 -1.35892
C  -4.58955 -1.80584 -0.95518
H  -4.27938 -2.05344 -1.98806
H  -5.50854 -2.40450 -0.77829
C  -4.10549 -2.34122  1.99093
H  -3.43724 -2.63645  2.81519
H  -5.01314 -2.96792  2.05590
H  -4.42804 -1.29911  2.15857
C  -3.36461 -4.44057 -0.16766
H  -3.07713 -4.61063 -1.21888
H  -4.39457 -4.81680 -0.03515
H  -2.69279 -5.05129  0.45398
C  -0.66546 -2.97393 -0.12810
C  -0.17545 -3.77899  0.95526
C   0.67927 -4.86783  0.68738
H   1.01973 -5.49145  1.52326
C   1.08844 -5.18322 -0.61342
H   1.73086 -6.05063 -0.80239
C   0.65289 -4.37207 -1.66859
H   0.96545 -4.60555 -2.69399
C  -0.20537 -3.27682 -1.45316
C  -0.53420 -3.49813  2.41607
H  -1.16362 -2.59265  2.42285
C   0.73440 -3.19189  3.24173
H   1.38915 -4.07846  3.32290
H   0.46750 -2.88236  4.26860
H   1.31780 -2.38318  2.77427
C  -1.31957 -4.65093  3.08199
H  -2.26970 -4.85927  2.56493
H  -1.55424 -4.40244  4.13290
H  -0.73051 -5.58576  3.08805
C  -0.64659 -2.45093 -2.65695
H  -1.34389 -1.69259 -2.26426
C   0.54120 -1.70808 -3.30646
H   1.05134 -1.06240 -2.56959
H   0.19317 -1.06835 -4.13720
H   1.28367 -2.41645 -3.71754
C  -1.39961 -3.29587 -3.70820
H  -0.74893 -4.07502 -4.14450
H  -1.75512 -2.65889 -4.53779
H  -2.27604 -3.80184 -3.26923
C   3.30994  0.39954 -0.15437
C   3.60656 -2.06716 -0.36205
H   2.51023 -1.91742 -0.33580
C   3.95236 -2.81638 -1.65717
H   3.71990 -2.20314 -2.54275
H   3.33540 -3.72725 -1.70390
H   5.01093 -3.11960 -1.70134
C   4.00479 -2.83905  0.90575
H   3.75272 -2.26313  1.81055
H   5.08113 -3.08071  0.93253
H   3.44067 -3.78523  0.93460
C   5.47548 -0.27994 -0.50207
C   6.63622 -1.19389 -0.75580
H   6.64125 -2.06324 -0.07888
H   7.58071 -0.65023 -0.59654
H   6.65023 -1.57778 -1.79105
C   5.48518  1.09758 -0.40107
C   6.65844  2.02216 -0.52644
H   6.69407  2.77283  0.27982
H   6.66518  2.56526 -1.48802
H   7.59412  1.44380 -0.47373
C   3.64852  2.86794  0.02067
H   2.55188  2.73549  0.01689
C   4.06636  3.41513  1.39608
H   3.80227  2.70722  2.19668
H   3.54006  4.36474  1.58833
H   5.14927  3.61725  1.45102
C   4.01194  3.82225 -1.12893
H   5.06852  4.13324 -1.10078
H   3.39703  4.73311 -1.04198
H   3.80229  3.36323 -2.10754
C  -0.53323  0.47585  2.53555
C  -1.70854  0.03045  2.41171
H  -2.66892 -0.22262  2.84202
C   0.53344  1.19439  3.32175
C   0.77193  2.59586  2.71982
H   1.53265  3.13273  3.31296
H   1.12368  2.50407  1.67735
H  -0.15205  3.19346  2.70050
C  -0.03217  1.33107  4.76570
H   0.71479  1.84051  5.39980
H  -0.96002  1.92410  4.78225
H  -0.24169  0.34146  5.20422
C   1.86076  0.40942  3.38611
H   2.25079  0.21768  2.36889
H   2.60993  0.99778  3.94575
H   1.72973 -0.55775  3.89621
```

III_{Bu}

SCF (BP86) Energy = -2265.65978921
Enthalpy 0K = -2264.515316
Enthalpy 298K = -2264.441832
Free Energy 298K = -2264.625184
Lowest Frequency = 8.9740 cm^{-1}
Second Frequency = 16.4997 cm^{-1}
SCF (BP86-D3BJ) Energy = -2266.01549617
SCF (C6H6) Energy = -2265.66466621
SCF (BS2) Energy = -3077.83887609

```
Cu  2.21407  0.67185  0.83667
Si -3.85182  0.49974 -2.16641
Si -2.79651 -3.10498 -0.99231
Al -1.57693 -0.27095  0.11096
N  -2.97594  0.72489 -0.61142
N  -1.44708 -2.04114 -0.43368
N   5.01543 -0.08938  0.15107
N   4.32046  1.58556 -1.04664
C  -3.32395  1.90251  0.16172
C  -4.29470  1.80504  1.20831
C  -4.60413  2.94555  1.97402
H  -5.34043  2.86108  2.78184
C  -3.99760  4.18104  1.72343
H  -4.25302  5.05832  2.32749
C  -3.06265  4.28022  0.68775
H  -2.58647  5.24683  0.48459
C  -2.71007  3.16712 -0.10045
```

C -4.99955 0.48798 1.53692
H -4.72961 -0.22342 0.73685
C -4.50191 -0.09814 2.87859
H -3.40932 -0.25588 2.87405
H -4.98746 -1.06763 3.09057
H -4.73021 0.58464 3.71630
C -6.53823 0.62223 1.54537
H -6.88704 1.28577 2.35641
H -7.01047 -0.36378 1.70208
H -6.91515 1.03472 0.59420
C -1.67315 3.36341 -1.20804
H -1.51669 2.37982 -1.68297
C -0.31286 3.82637 -0.64220
H -0.39600 4.80806 -0.14206
H 0.42281 3.93609 -1.46103
H 0.07673 3.09870 0.08887
C -2.16619 4.34756 -2.29368
H -3.11385 4.01663 -2.75056
H -1.41645 4.44615 -3.09969
H -2.33521 5.35505 -1.87346
C -5.26404 1.76923 -2.29419
H -6.05144 1.56506 -1.54961
H -5.72286 1.71099 -3.29677
H -4.91894 2.80243 -2.13272
C -2.68406 0.70728 -3.66438
H -2.34957 1.75122 -3.77947
H -3.20022 0.41363 -4.59598
H -1.78561 0.07425 -3.56715
C -4.65921 -1.22410 -2.32777
H -5.32911 -1.38556 -1.46191
H -5.33518 -1.12695 -3.20309
C -3.71938 -2.43351 -2.52598
H -2.98507 -2.23384 -3.32963
H -4.30415 -3.30790 -2.88163
C -4.09882 -3.36622 0.37791
H -3.69361 -3.95624 1.21567
H -4.97210 -3.90896 -0.02644
H -4.46250 -2.40868 0.78673
C -2.08801 -4.79824 -1.49897
H -1.42321 -4.71279 -2.37469
H -2.91933 -5.47334 -1.76815
H -1.50944 -5.27154 -0.69012
C -0.17683 -2.71321 -0.26028
C 0.14878 -3.39043 0.95551
C 1.36969 -4.08946 1.04289
H 1.61121 -4.61855 1.97237
C 2.26620 -4.13994 -0.02919
H 3.19493 -4.71499 0.05192
C 1.95526 -3.45917 -1.21345
H 2.65323 -3.49988 -2.05720
C 0.75011 -2.74613 -1.35048
C -0.78258 -3.40571 2.16857
H -1.67345 -2.81149 1.90179
C -0.12722 -2.73675 3.39712
H 0.77374 -3.28837 3.72187
H -0.83105 -2.71987 4.24865
H 0.16382 -1.70052 3.16209
C -1.25025 -4.83712 2.51972
H -1.74599 -5.33153 1.66714
H -1.96255 -4.81534 3.36375
H -0.39968 -5.47395 2.82208
C 0.44865 -2.00393 -2.65225
H -0.64770 -1.88090 -2.69873
C 1.06640 -0.58731 -2.61891
H 0.73289 -0.01579 -1.73163
H 0.79517 -0.01068 -3.52204
H 2.16762 -0.64642 -2.55681
C 0.89306 -2.75603 -3.92284
H 1.99272 -2.82468 -4.00356
H 0.53606 -2.22664 -4.82356
H 0.49061 -3.78287 -3.94834
C 3.90391 0.67938 -0.09946
C 4.98102 -1.14391 1.19885
H 3.90189 -1.22564 1.42689
C 5.46282 -2.50972 0.68823
H 4.96363 -2.77377 -0.25697
H 5.20028 -3.27901 1.43271
H 6.55450 -2.54869 0.54411
C 5.70804 -0.68496 2.47409
H 5.30867 0.28053 2.82390
H 6.79524 -0.57813 2.31881
H 5.55639 -1.42950 3.27338
C 6.11345 0.33429 -0.61246
C 7.47414 -0.29376 -0.60098
H 7.86291 -0.43423 0.42121
H 8.18794 0.35305 -1.13472
H 7.48799 -1.27858 -1.10055
C 5.67087 1.39797 -1.37554
C 6.43232 2.18761 -2.39655
H 6.32034 3.27553 -2.25734
H 6.11965 1.95053 -3.42905
H 7.50672 1.95824 -2.32118
C 3.40587 2.65997 -1.51287
H 2.43923 2.36629 -1.06196
C 3.79666 4.02658 -0.92607
H 3.87820 3.96786 0.17108
H 3.01714 4.76673 -1.17178
H 4.75232 4.40100 -1.33041
C 3.23675 2.68006 -3.03981
H 4.12922 3.06324 -3.56058
H 2.39575 3.34509 -3.29782
H 3.00494 1.67451 -3.42559
C 0.64353 0.90409 1.92674
C -0.64743 0.56936 1.60317
H -1.43039 0.87808 2.33193
C 0.93238 1.63617 3.27541
C 1.55643 3.02024 2.96083
H 1.85432 3.54048 3.89109
H 2.45525 2.90639 2.32579
H 0.84092 3.66372 2.42047
C -0.30074 1.85827 4.18051
H -0.00046 2.36756 5.11438
H -1.06361 2.48628 3.68882
H -0.77753 0.90260 4.46066
C 1.97306 0.80727 4.07058
H 2.88171 0.64064 3.46243
H 2.26892 1.32985 5.00010
H 1.57006 -0.18268 4.34620

TS(III-IV)$_{Bu}$
SCF (BP86) Energy = -2265.62351600
Enthalpy 0K = -2264.484144
Enthalpy 298K = -2264.410535
Free Energy 298K = -2264.593691
Lowest Frequency = -657.2197 cm^{-1}
Second Frequency = 12.2099 cm^{-1}
SCF (BP86-D3BJ) Energy = -2265.97578276
SCF (C6H6) Energy = -2265.62907146
SCF (BS2) Energy = -3077.80382199

Cu -2.32649 -0.85328 0.86767
Si 3.74646 -0.67382 -2.16681
Al 1.63973 0.31036 0.15463
Si 3.06981 3.02686 -0.90137
N -5.04239 0.13018 0.30934
N 2.85551 -0.87294 -0.62500
N -4.51673 -1.49200 -1.04138
N 1.62190 2.08407 -0.40586
C -4.02153 -0.73016 -0.01258
H 1.59142 -0.04277 1.84881
C 1.33407 -3.36789 -1.26159
H 1.22961 -2.35324 -1.68285
C 2.36624 -3.27788 -0.13540
C 0.38564 2.82226 -0.30389
C 4.05980 -2.08332 1.19975
C 3.94612 2.28903 -2.43391
H 3.20502 2.20242 -3.25175
H 4.64069 3.09241 -2.75863
C 4.72765 0.96678 -2.26234
H 5.39740 1.01969 -1.38254
H 5.40483 0.82011 -3.13030
C 3.09233 -2.07597 0.14314
C 4.35437 3.13457 0.50622
H 3.98392 3.75354 1.33990

H 5.29246 3.58771 0.13859
H 4.60194 2.14033 0.91461
C 3.54503 -4.43168 1.67896
H 3.71850 -5.33757 2.26998
C -3.70567 -2.59398 -1.62199
H -2.72486 -2.45700 -1.13080
C 0.04206 3.52851 0.89237
C 5.04283 -2.05903 -2.34794
H 5.90053 -1.89151 -1.67542
H 5.42590 -2.07178 -3.38346
H 4.63550 -3.05608 -2.11912
C -4.89856 1.08218 1.44419
H -3.83311 0.96777 1.72020
C 4.25809 -3.25886 1.94968
H 4.99724 -3.25295 2.75969
C 0.91050 3.47558 2.15049
H 1.76524 2.81432 1.92678
C -6.16464 -0.09004 -0.50112
C -0.72236 -0.94774 1.94409
C -0.05315 -3.80649 -0.74509
H -0.02368 -4.81952 -0.30494
H -0.77616 -3.83584 -1.58192
H -0.42850 -3.10910 0.02228
C -5.83043 -1.12087 -1.36012
C 1.79850 -4.31704 -2.39103
H 2.76359 -4.00432 -2.82218
H 1.05488 -4.34553 -3.20850
H 1.92151 -5.34939 -2.01706
C 2.61196 -4.43092 0.63688
H 2.05689 -5.34964 0.41207
C 4.90632 -0.85609 1.54144
H 4.62214 -0.06648 0.82380
C 0.11667 -0.35855 1.12660
C -0.50141 2.88880 -1.42508
C -5.74775 0.64968 2.65110
H -5.49934 1.28364 3.51838
H -5.53913 -0.39847 2.92022
H -6.82975 0.75340 2.46376
C -0.21574 2.11568 -2.71110
H 0.81619 1.73438 -2.62224
C -1.13859 4.29681 0.92525
H -1.38643 4.84890 1.83994
C 2.57959 -0.72873 -3.67944
H 2.15594 -1.73622 -3.82455
H 3.13051 -0.45850 -4.59825
H 1.73869 -0.02267 -3.57487
C 4.61684 -0.33095 2.96550
H 4.88233 -1.08214 3.73099
H 3.54822 -0.08568 3.08322
H 5.20789 0.57940 3.17286
C -6.65745 -1.71938 -2.45703
H -6.30709 -1.41506 -3.45919
H -7.70228 -1.38518 -2.36198
H -6.66298 -2.82123 -2.42677
C -1.15733 0.89472 -2.83479
H -1.08679 0.24686 -1.94351
H -0.90635 0.29168 -3.72660
H -2.20945 1.22138 -2.92702
C 2.54303 4.79084 -1.38970
H 1.90300 4.78716 -2.28769
H 3.44147 5.39160 -1.61563
H 1.98276 5.29898 -0.58896
C 0.14125 2.85297 3.33702
H -0.72184 3.47796 3.63080
H 0.79915 2.75856 4.21950
H -0.23323 1.85112 3.07170
C -4.24521 -3.97291 -1.20756
H -5.21747 -4.20360 -1.67457
H -4.35894 -4.03306 -0.11316
H -3.53180 -4.75278 -1.52153
C 1.46529 4.86465 2.53957
H 2.05576 5.31471 1.72386
H 2.11638 4.78719 3.42865
H 0.65039 5.56886 2.78633
C -5.12166 2.54258 1.02771
H -6.17504 2.75857 0.78643
H -4.48834 2.80669 0.16639
H -4.83347 3.19820 1.86580
C 6.41794 -1.12989 1.36935
H 7.00216 -0.21131 1.55680
H 6.65640 -1.48558 0.35305
H 6.77132 -1.89817 2.08010
C -0.28968 2.99094 -3.98010
H -1.31036 3.37474 -4.15762
H -0.00088 2.40450 -4.87039
H 0.38522 3.86084 -3.91210
C -3.50125 -2.44580 -3.13719
H -2.73118 -3.16332 -3.46585
H -3.15109 -1.43238 -3.39054
H -4.41697 -2.66069 -3.71137
C -1.67368 3.66377 -1.34039
H -2.33992 3.71952 -2.20975
C -7.44126 0.69276 -0.44630
H -7.83172 0.79021 0.57981
H -8.21567 0.18658 -1.04338
H -7.32545 1.71139 -0.85688
C -1.99305 4.37974 -0.18009
H -2.89381 5.00255 -0.14077
C -0.67571 -1.50338 3.36903
C -0.94726 -3.02786 3.28827
H -0.16280 -3.53614 2.70332
H -0.96385 -3.46783 4.30240
H -1.92088 -3.22676 2.80568
C -1.79455 -0.84035 4.21001
H -1.81525 -1.26720 5.22937
H -1.63619 0.24814 4.29690
H -2.78303 -1.00749 3.74460
C 0.68811 -1.26820 4.04848
H 0.67837 -1.69461 5.06775
H 1.50667 -1.74480 3.48423
H 0.91908 -0.19280 4.13130

IV_{Bu}
SCF (BP86) Energy = -2265.64860749
Enthalpy 0K = -2264.507754
Enthalpy 298K = -2264.433706
Free Energy 298K = -2264.617409
Lowest Frequency = 7.1646 cm^{-1}
Second Frequency = 18.0949 cm^{-1}
SCF (BP86-D3BJ) Energy = -2266.00902988
SCF (C6H6) Energy = -2265.65583773
SCF (BS2) Energy = -3077.82934187

Cu -1.89305 -0.62126 0.42103
Si 3.89761 -0.00571 -1.90657
Al 1.73456 0.49778 0.56036
Si 2.59262 3.41597 -0.41001
N -4.81033 -0.08300 0.44966
N 2.97210 -0.52716 -0.47024
N -4.24094 -1.38653 -1.19854
N 1.29797 2.20026 -0.16636
C -3.72310 -0.67082 -0.14717
H 2.23353 0.60501 2.09572
C 1.75671 -2.96336 -1.68678
H 1.59158 -1.89789 -1.92101
C 2.67218 -3.00806 -0.46136
C -0.04371 2.69880 -0.14722
C 4.16190 -1.96565 1.20299
C 3.73259 2.97784 -1.88191
H 3.11056 2.88401 -2.79271
H 4.34828 3.88836 -2.04190
C 4.65376 1.74628 -1.72346
H 5.20140 1.78756 -0.76225
H 5.44271 1.76997 -2.50499
C 3.26701 -1.82136 0.08823
C 3.70914 3.57221 1.13285
H 3.17262 4.04923 1.96983
H 4.60033 4.18449 0.90593
H 4.05536 2.58600 1.48551
C 3.81933 -4.39775 1.20093
H 4.03480 -5.38424 1.62623
C -3.36893 -2.24925 -2.04223
H -2.35184 -1.96839 -1.71279
C -0.64173 3.20367 1.05709

```
C   5.38719 -1.16919 -2.19098
H   6.23264 -0.90358 -1.53468
H   5.73485 -1.07374 -3.23482
H   5.14372 -2.22734 -2.00546
C  -4.66148  0.71228  1.70025
H  -3.56831  0.85933  1.77123
C   4.40746 -3.24714  1.73592
H   5.09463 -3.34237  2.58529
C   0.09046  3.17858  2.40072
H   1.07432  2.70979  2.22790
C  -5.99618 -0.43078 -0.21000
C  -0.46832 -1.49693  1.69483
C   0.37744 -3.60486 -1.42248
H   0.46501 -4.67845 -1.17460
H  -0.25738 -3.52953 -2.32564
H  -0.13688 -3.09879 -0.58931
C  -5.63425 -1.25694 -1.25859
C   2.41693 -3.64147 -2.91035
H   3.39342 -3.19140 -3.15150
H   1.77147 -3.55635 -3.80399
H   2.58617 -4.71730 -2.72271
C   2.96250 -4.26621  0.10208
H   2.51189 -5.16408 -0.33913
C   4.91759 -0.78474  1.81917
H   4.56220  0.12645  1.30795
C   0.03011 -0.61191  0.93956
C  -0.81080  2.75232 -1.35908
C  -5.12480 -0.09583  2.92398
H  -4.87488  0.45621  3.84509
H  -4.62258 -1.07604  2.96154
H  -6.21495 -0.26486  2.92251
C  -0.24921  2.20090 -2.66616
H   0.80575  1.95362 -2.46026
C  -1.93075  3.77161  1.00824
H  -2.36155  4.18529  1.92884
C   2.88679  0.00504 -3.53310
H   2.66884 -1.01596 -3.88620
H   3.46504  0.51627 -4.32434
H   1.92849  0.53646 -3.42270
C   4.63101 -0.62282  3.32802
H   4.96429 -1.50629  3.90251
H   3.55470 -0.47065  3.50610
H   5.17018  0.25416  3.72947
C  -6.51832 -1.87034 -2.30151
H  -6.41250 -1.37453 -3.28239
H  -7.57271 -1.77425 -1.99974
H  -6.31659 -2.94404 -2.44670
C  -0.96964  0.89440 -3.06544
H  -0.89012  0.14231 -2.25968
H  -0.53391  0.46150 -3.98408
H  -2.04336  1.08274 -3.25151
C   1.83488  5.12321 -0.79424
H   1.29648  5.12243 -1.75661
H   2.63973  5.87648 -0.85710
H   1.12199  5.44793 -0.01931
C  -0.65290  2.30871  3.43892
H  -1.65424  2.71941  3.66780
H  -0.08588  2.26648  4.38607
H  -0.77552  1.27761  3.06832
C  -3.57273 -3.73773 -1.71503
H  -4.56587 -4.10344 -2.02543
H  -3.45340 -3.91991 -0.63511
H  -2.81478 -4.33483 -2.24800
C   0.32610  4.59841  2.96443
H   0.89310  5.22864  2.25910
H   0.89567  4.54951  3.90967
H  -0.62764  5.11446  3.17821
C  -5.31358  2.09897  1.61235
H  -6.41323  2.05584  1.65881
H  -5.00378  2.61877  0.69322
H  -4.97049  2.70273  2.46831
C   6.44212 -0.90739  1.58612
H   6.96697 -0.01801  1.97874
H   6.68673 -1.00537  0.51606
H   6.85559 -1.79275  2.10215
C  -0.29152  3.21672 -3.82745
H  -1.32751  3.47900 -4.10915
H   0.19705  2.79730 -4.72487
H   0.22826  4.15323 -3.56465
C  -3.48506 -1.93460 -3.54107
H  -2.66461 -2.44205 -4.07447
H  -3.39103 -0.85347 -3.72813
H  -4.43138 -2.29277 -3.97664
C  -2.09738  3.32385 -1.35565
H  -2.65903  3.37921 -2.29638
C  -7.36741  0.04992  0.15635
H  -7.58017 -0.06678  1.23155
H  -8.12449 -0.53275 -0.39087
H  -7.52125  1.11218 -0.10174
C  -2.65823  3.85078 -0.18617
H  -3.63943  4.33875 -0.21033
C  -0.71229 -2.52524  2.74584
C  -1.63043 -3.65642  2.22024
H  -1.17969 -4.15830  1.34865
H  -1.78430 -4.40971  3.01262
H  -2.61715 -3.25962  1.92345
C  -1.36307 -1.85599  3.98426
H  -1.51223 -2.60744  4.77967
H  -0.72231 -1.05160  4.37956
H  -2.34493 -1.42127  3.73074
C   0.67390 -3.10917  3.13568
H   0.54033 -3.86022  3.93472
H   1.16457 -3.58983  2.27466
H   1.34335 -2.31712  3.50632
```

V$_{Bu}$

SCF (BP86) Energy = -2265.66982162
Enthalpy 0K = -2264.527603
Enthalpy 298K = -2264.454202
Free Energy 298K = -2264.633859
Lowest Frequency = 15.1349 cm^{-1}
Second Frequency = 18.4728 cm^{-1}
SCF (BP86-D3BJ) Energy = -2266.03900020
SCF (C6H6) Energy = -2265.67408047
SCF (BS2) Energy = -3077.85070970

```
Cu -1.46714 -0.08352  0.30081
Al  1.18246 -0.08186  0.13308
Si  3.84528  1.54647  0.95630
Si  3.69247 -1.69665 -1.31369
N   2.16895  1.50802  0.31998
N   2.08013 -1.59580 -0.54197
N  -3.72910  1.48118 -0.77177
C   1.24589 -2.77339 -0.52744
N  -4.32874 -0.59689 -0.53814
C  -3.26263  0.25561 -0.36346
C   0.00947 -0.52855  1.67976
C   1.53825  2.77498  0.05455
C   0.40652  5.34491 -0.47418
H  -0.00704  6.34056 -0.66987
C   1.28794 -3.68652  0.57848
C  -1.08096 -0.65523  2.31544
C  -5.24158 -2.51749  0.80338
H  -6.26324 -2.56272  0.39306
H  -5.24662 -1.86039  1.68762
H  -4.97766 -3.53516  1.13623
C  -3.21653 -0.08550  3.51661
H  -3.85659 -0.36492  4.37233
H  -3.79737 -0.19702  2.58654
H  -2.94728  0.97840  3.62019
C   1.01901  4.61249 -1.49830
H   1.07803  5.03963 -2.50688
C   0.88370  3.51532  1.09484
C   0.34403  4.78680  0.80783
H  -0.12704  5.35679  1.61797
C   1.58821  3.34692 -1.25963
C  -5.06236  1.40358 -1.19877
C  -4.21445 -2.04838 -0.24076
H  -3.20389 -2.12860  0.19996
C  -4.23965 -2.90608 -1.51633
H  -5.24175 -2.94615 -1.97519
H  -3.94682 -3.93812 -1.26272
H  -3.52438 -2.52262 -2.25861
C   2.31993 -3.56434  1.70324
```

H 2.97105 -2.71093 1.44453
C 5.11301 0.63600 -0.15293
H 5.06637 1.07965 -1.16617
H 6.09651 0.95349 0.25444
C -1.95153 -0.97673 3.48978
C -2.83600 2.67190 -0.78678
H -1.90685 2.29245 -0.32328
C -5.88975 2.56080 -1.66998
H -5.37939 3.15485 -2.44518
H -6.15672 3.24669 -0.84677
H -6.83147 2.19336 -2.10652
C 0.34598 -3.04594 -1.61122
C -5.44369 0.08445 -1.05314
C -2.48787 3.10756 -2.21839
H -2.12726 2.25062 -2.80883
H -1.67903 3.85477 -2.17321
H -3.34539 3.56305 -2.74214
C 0.74982 2.98754 2.52600
H 1.12868 1.95115 2.52809
C 2.25888 2.61842 -2.42090
H 2.71222 1.71048 -1.98925
C -3.36551 3.81927 0.08589
H -4.23121 4.32979 -0.36708
H -2.56037 4.56192 0.20741
H -3.65097 3.45771 1.08715
C 3.91640 0.73381 2.68229
H 3.37511 1.33163 3.43388
H 4.96116 0.62128 3.02274
H 3.46011 -0.27128 2.66656
C 0.38731 -4.76869 0.61552
H 0.41892 -5.45626 1.46935
C -1.13091 -0.74143 4.78717
H -1.74952 -0.97956 5.67100
H -0.80601 0.30885 4.86275
H -0.23384 -1.38084 4.80804
C 5.05602 -0.90587 -0.23116
H 5.00050 -1.34887 0.78180
H 6.00273 -1.29239 -0.66418
C -2.36319 -2.47088 3.41489
H -1.47556 -3.12297 3.39939
H -2.94964 -2.67536 2.50512
H -2.98038 -2.73491 4.29238
C 1.57715 3.81282 3.53930
H 1.24609 4.86686 3.56099
H 2.65198 3.80663 3.29809
H 1.45759 3.40535 4.55933
C 1.22891 2.17368 -3.48344
H 0.45447 1.52794 -3.03603
H 1.72200 1.60811 -4.29434
H 0.72526 3.04468 -3.94078
C 1.68007 -3.26757 3.07724
H 1.00753 -4.08931 3.38583
H 1.09471 -2.33588 3.04584
H 2.45940 -3.16892 3.85447
C 3.38720 3.44951 -3.07020
H 2.99681 4.36464 -3.55066
H 3.90057 2.86059 -3.85104
H 4.14057 3.76031 -2.32676
C 4.18679 -3.52240 -1.56479
H 4.58043 -3.96540 -0.63578
H 4.98353 -3.58236 -2.32675
H 3.34353 -4.14545 -1.90437
C 3.80625 -0.87423 -3.03756
H 3.23530 -1.43510 -3.79500
H 4.86344 -0.85976 -3.36033
H 3.44326 0.16519 -3.04330
C 0.36844 -2.22776 -2.90443
H 1.02485 -1.35989 -2.72338
C -0.72626 2.94861 2.97831
H -0.80533 2.52346 3.99502
H -1.33279 2.32432 2.30167
H -1.17127 3.95937 3.01049
C 3.19499 -4.83580 1.81183
H 4.01116 -4.68158 2.54004
H 3.64292 -5.11440 0.84471
H 2.60201 -5.69998 2.16176
C -6.78204 -0.52681 -1.33855
H -7.33662 -0.77830 -0.41706
H -6.70655 -1.44510 -1.94325
H -7.40178 0.18510 -1.90558
C -0.52981 -4.99978 -0.41657
H -1.21552 -5.85338 -0.36946
C -0.52777 -4.14884 -1.52790
H -1.20707 -4.35296 -2.36449
C -1.01394 -1.68717 -3.32357
H -0.91824 -1.04175 -4.21518
H -1.47016 -1.09057 -2.51567
H -1.70547 -2.50716 -3.59137
C 4.47907 3.34338 1.03417
H 4.68844 3.72519 0.02041
H 5.42349 3.37608 1.60531
H 3.76793 4.03896 1.50480
C 0.97210 -3.06165 -4.06055
H 0.33473 -3.93482 -4.28972
H 1.97569 -3.44350 -3.81079
H 1.05395 -2.45568 -4.98108
H -0.23073 0.29568 -0.83971

VI$_{Bu}$

SCF (BP86) Energy = -2500.22536733
Enthalpy 0K = -2498.945831
Enthalpy 298K = -2498.862882
Free Energy 298K = -2499.063243
Lowest Frequency = 16.5878 cm^{-1}
Second Frequency = 17.6092 cm^{-1}
SCF (BP86-D3BJ) Energy = -2500.63831815
SCF (C6H6) Energy = -2500.23240616
SCF (BS2) Energy = -3312.46112005

Cu -2.01667 -0.10990 -0.29142
Si 3.81490 -1.70344 -1.81937
Al 1.62776 0.03273 -0.00733
Si 3.95954 2.19856 -0.97235
N -4.75632 -1.37783 0.09663
N 2.80015 -1.44261 -0.37620
N -4.85535 0.78961 -0.04496
N 2.30222 1.80520 -0.42405
C -3.97300 -0.26343 -0.10368
H 1.20006 -0.09372 1.54043
C 0.89603 -3.75572 -0.59386
H 0.94642 -2.83331 -1.19717
C 1.96224 -3.64065 0.49519
C 1.48055 2.88449 0.05921
C 3.76459 -2.48042 1.68926
C 4.51415 1.10373 -2.44248
H 3.69392 1.05001 -3.18383
H 5.30073 1.71751 -2.92995
C 5.07853 -0.30196 -2.12856
H 5.77834 -0.25744 -1.27267
H 5.68742 -0.66241 -2.98454
C 2.83172 -2.50644 0.60074
C 5.35837 2.07329 0.33227
H 5.37266 1.12421 0.88777
H 5.30569 2.89415 1.06477
H 6.32573 2.16512 -0.19608
C 2.99068 -4.67095 2.46959
H 3.05874 -5.50448 3.17739
C -4.40398 2.18348 -0.30607
H -3.30936 2.07187 -0.39901
C 1.54193 3.32103 1.43107
C 4.81463 -3.32127 -1.67711
H 5.53150 -3.28756 -0.84052
H 5.38587 -3.48179 -2.60851
H 4.16910 -4.19911 -1.51490
C -4.18226 -2.74704 0.01501
H -3.10415 -2.55137 -0.12570
C 3.82584 -3.55658 2.59469
H 4.55313 -3.51695 3.41484
C 2.53490 2.74746 2.44309
H 3.10090 1.95052 1.93332
C -6.10280 -1.02797 0.29126
C -0.95277 -0.52295 -2.03024
C -0.51573 -3.83020 0.02861
H -0.62366 -4.71500 0.68188

H	-1.28331	-3.90093	-0.76345
H	-0.72024	-2.93144	0.63537
C	-6.16442	0.34865	0.20028
C	1.13202	-4.96637	-1.52510
H	2.11358	-4.90945	-2.02387
H	0.35522	-5.01735	-2.31034
H	1.09917	-5.91870	-0.96582
C	2.06365	-4.69609	1.42382
H	1.39783	-5.56179	1.31803
C	4.70306	-1.29993	1.91298
H	4.47711	-0.57785	1.11109
C	-0.11550	-0.22511	-1.12232
C	0.55722	3.56961	-0.81276
C	-4.69338	-3.50891	-1.22034
H	-4.09072	-4.42214	-1.35812
H	-4.60264	-2.89722	-2.13167
H	-5.74470	-3.82350	-1.11417
C	0.43838	3.27613	-2.31151
H	1.07293	2.39675	-2.51944
C	0.69368	4.35011	1.88657
H	0.75866	4.66044	2.93614
C	2.76423	-1.79050	-3.41233
H	2.04064	-2.62192	-3.38177
H	3.40499	-1.92298	-4.30221
H	2.20200	-0.85091	-3.54053
C	4.42787	-0.61375	3.26861
H	4.66533	-1.28412	4.11467
H	3.36829	-0.32450	3.35178
H	5.04761	0.29452	3.38025
C	-7.36096	1.23479	0.37186
H	-7.33053	1.79887	1.32057
H	-8.27810	0.62591	0.38378
H	-7.46626	1.96558	-0.44614
C	-1.01365	2.95067	-2.72569
H	-1.40448	2.08187	-2.17342
H	-1.06602	2.72549	-3.80642
H	-1.68231	3.81282	-2.54815
C	4.08499	4.01021	-1.57470
H	5.13319	4.34387	-1.47852
H	3.45823	4.69899	-0.98482
H	3.80435	4.10874	-2.63572
C	1.82896	2.11576	3.66218
H	1.21402	2.85770	4.20364
H	2.57258	1.71768	4.37480
H	1.17957	1.28739	3.33639
C	-4.93733	2.71251	-1.64796
H	-6.02215	2.90858	-1.62477
H	-4.72712	2.00072	-2.46171
H	-4.43455	3.66405	-1.88685
C	3.52841	3.83551	2.91614
H	4.01573	4.34459	2.06791
H	4.31563	3.39360	3.55259
H	3.01583	4.61161	3.51299
C	-4.35631	-3.55269	1.31227
H	-5.39802	-3.87660	1.46883
H	-4.03127	-2.97471	2.19133
H	-3.73271	-4.45999	1.25507
C	6.19309	-1.69175	1.80094
H	6.83952	-0.80529	1.93302
H	6.42640	-2.13701	0.81928
H	6.47542	-2.42712	2.57588
C	0.92321	4.46995	-3.17015
H	0.25394	5.34026	-3.04170
H	0.91672	4.20313	-4.24246
H	1.93842	4.79290	-2.90170
C	-4.67706	3.12847	0.87297
H	-4.15142	4.08091	0.69483
H	-4.28911	2.70106	1.81051
H	-5.74819	3.35755	0.99368
C	-0.26344	4.59803	-0.30155
H	-0.94640	5.11678	-0.98513
C	-7.21579	-1.98633	0.58983
H	-7.27902	-2.80650	-0.14378
H	-8.17970	-1.45509	0.56610
H	-7.11988	-2.44102	1.59136
C	-0.21371	4.99158	1.03941
H	-0.85728	5.79614	1.41218
C	-1.84032	0.76875	2.37041
C	-1.43701	1.38699	1.38233
H	-0.94057	2.12817	0.76752
C	-2.23900	0.00219	3.56511
C	-1.73828	0.77934	4.81661
H	-0.64367	0.89461	4.79944
H	-2.19220	1.78269	4.86945
H	-2.01948	0.22098	5.72668
C	-1.56226	-1.39358	3.51964
H	-1.79952	-1.95535	4.44019
H	-1.91747	-1.97531	2.65307
H	-0.46864	-1.29791	3.43023
C	-3.78107	-0.14072	3.63962
H	-4.26639	0.84632	3.72280
H	-4.18001	-0.64557	2.74549
H	-4.05545	-0.73338	4.52992
C	-1.53092	-0.93285	-3.35735
C	-2.94706	-0.34731	-3.57793
H	-3.64268	-0.67324	-2.78789
H	-2.92047	0.75497	-3.57701
H	-3.33724	-0.68110	-4.55572
C	-0.60409	-0.42034	-4.49223
H	-1.03909	-0.69264	-5.47060
H	-0.49500	0.67454	-4.45358
H	0.39596	-0.87277	-4.42264
C	-1.59903	-2.47989	-3.42998
H	-0.59932	-2.92420	-3.30029
H	-1.99478	-2.79286	-4.41282
H	-2.25893	-2.88549	-2.64652

TS(E)$_{Bu}$

SCF (BP86) Energy = -2500.21084028
Enthalpy 0K = -2498.932201
Enthalpy 298K = -2498.850279
Free Energy 298K = -2499.046440
Lowest Frequency = -165.3391 cm^{-1}
Second Frequency = 10.7565 cm^{-1}
SCF (BP86-D3BJ) Energy = -2500.62569856
SCF (C6H6) Energy = -2500.21667501
SCF (BS2) Energy = -3312.44672491

Cu	2.13231	-0.47796	0.02489
Si	-3.89134	-0.19820	2.01504
Al	-1.39335	0.12408	-0.07226
Si	-3.40620	2.65705	-0.55525
N	4.98423	-1.56338	0.01032
N	-2.86286	-0.84519	0.69803
N	4.91171	0.60026	-0.19252
N	-1.75190	2.00613	-0.27754
C	4.11120	-0.50096	-0.01173
H	-1.01190	-0.69627	-1.45256
C	-1.21267	-3.09454	1.83393
H	-1.08713	-2.00874	1.97672
C	-2.39294	-3.30123	0.88500
C	-0.69477	2.97913	-0.40270
C	-4.22017	-2.54402	-0.56936
C	-4.57284	2.50173	0.96313
H	-4.05145	2.99535	1.80627
H	-5.41529	3.18309	0.71641
C	-5.13137	1.13571	1.41609
H	-5.73886	0.68839	0.60813
H	-5.83868	1.28559	2.25910
C	-3.14470	-2.21265	0.32631
C	-4.20455	1.87229	-2.10623
H	-3.83590	0.85228	-2.28899
H	-3.96359	2.47506	-2.99831
H	-5.30456	1.82697	-2.01873
C	-3.77789	-4.94316	-0.30228
H	-4.02436	-5.98487	-0.53512
C	4.30848	1.94881	-0.38671
H	3.24223	1.78110	-0.14550
C	-0.01871	3.19014	-1.65398
C	-4.93060	-1.58514	2.81361
H	-5.61472	-2.07218	2.10085
H	-5.53559	-1.16137	3.63479
H	-4.28845	-2.37398	3.23852
C	4.48099	-2.95985	0.10576

```
H   3.41511 -2.81925  0.36132
C  -4.51039 -3.89200 -0.85934
H  -5.34017 -4.11751 -1.54006
C  -0.40834  2.45523 -2.94026
H  -1.05806  1.61062 -2.65263
C   6.31239 -1.13545 -0.16095
C   1.23728 -0.18618  1.88970
C   0.08896 -3.64532  1.20853
H   0.00952 -4.72887  1.00583
H   0.94475 -3.49706  1.89030
H   0.31951 -3.13353  0.25958
C   6.26480  0.23883 -0.28914
C  -1.45534 -3.73459  3.21977
H  -2.35769 -3.32512  3.70392
H  -0.59687 -3.54948  3.89148
H  -1.58608 -4.82920  3.14670
C  -2.72426 -4.63200  0.56053
H  -2.13962 -5.44555  1.00704
C  -5.09325 -1.48771 -1.24508
H  -4.70691 -0.50643 -0.92856
C   0.36303 -0.05039  0.97913
C  -0.33237  3.82469  0.70207
C   5.14492 -3.76530  1.23523
H   4.55975 -4.68322  1.40966
H   5.16843 -3.19502  2.17742
H   6.17228 -4.07376  0.98477
C  -1.04389  3.75745  2.05339
H  -1.87365  3.04078  1.93943
C   0.99238  4.16890 -1.74816
H   1.47506  4.33720 -2.71834
C  -2.80830  0.54654  3.39671
H  -2.32371 -0.25824  3.97464
H  -3.41514  1.15027  4.09488
H  -2.01406  1.19206  2.99084
C  -4.99639 -1.56896 -2.78538
H  -5.39912 -2.52590 -3.16330
H  -3.95233 -1.49037 -3.12941
H  -5.57459 -0.75474 -3.25662
C   7.40455  1.19805 -0.45149
H   7.24629  1.90110 -1.28574
H   8.33280  0.64436 -0.66178
H   7.57936  1.79688  0.45961
C  -0.10373  3.21709  3.14880
H   0.29435  2.23524  2.85207
H  -0.63443  3.10899  4.11201
H   0.75059  3.90008  3.31113
C  -3.36267  4.54033 -0.88811
H  -4.30820  4.82601 -1.38335
H  -2.53061  4.84629 -1.54112
H  -3.28765  5.12683  0.04130
C   0.81343  1.88983 -3.69462
H   1.48126  2.69376 -4.05353
H   0.48557  1.32597 -4.58611
H   1.39456  1.20651 -3.05679
C   4.85115  3.00507  0.58757
H   5.86968  3.33697  0.32845
H   4.85258  2.63279  1.62444
H   4.18926  3.88548  0.54489
C  -1.20552  3.38061 -3.89128
H  -2.11182  3.78385 -3.41346
H  -1.51430  2.83259 -4.79975
H  -0.58749  4.23969 -4.21025
C   4.55040 -3.67965 -1.25217
H   5.58927 -3.82610 -1.59264
H   4.00288 -3.11605 -2.02373
H   4.08432 -4.67525 -1.16365
C  -6.57567 -1.58504 -0.81607
H  -7.17109 -0.78513 -1.29234
H  -6.69702 -1.49271  0.27562
H  -7.01744 -2.55150 -1.11858
C  -1.63010  5.11605  2.49905
H  -0.83355  5.85528  2.69828
H  -2.20666  4.99587  3.43357
H  -2.29939  5.54983  1.73913
C   4.39084  2.39416 -1.85531
H   3.77351  3.29712 -1.98715
H   3.99410  1.61506 -2.52546
H   5.42266  2.63394 -2.16381
C   0.69870  4.77337  0.55981
H   0.96097  5.40294  1.41879
C   7.51808 -2.02577 -0.15816
H   7.75001 -2.41840  0.84767
H   8.39925 -1.45960 -0.49800
H   7.40591 -2.88942 -0.83457
C   1.37024  4.95430 -0.65352
H   2.14843  5.71956 -0.75508
C   0.69688 -1.56931 -2.48229
C   1.74449 -1.10899 -1.94934
H   2.76303 -1.04408 -2.32922
C  -0.20428 -2.31849 -3.38709
C  -1.16669 -1.38438 -4.15581
H  -1.80028 -0.82897 -3.44773
H  -0.61162 -0.65781 -4.77139
H  -1.81041 -1.98582 -4.82041
C  -1.00038 -3.41140 -2.63481
H  -1.59153 -3.99429 -3.36192
H  -0.32454 -4.10132 -2.10355
H  -1.68715 -2.96482 -1.90053
C   0.76161 -3.00548 -4.40789
H   1.35385 -2.26003 -4.96391
H   1.45105 -3.69782 -3.89775
H   0.16037 -3.58246 -5.13196
C   1.92158 -0.28032  3.22304
C   2.89270  0.91331  3.41599
H   3.68430  0.88505  2.64899
H   2.37031  1.87906  3.34362
H   3.36930  0.84768  4.41033
C   0.84286 -0.27851  4.33893
H   1.33372 -0.35228  5.32582
H   0.23781  0.63975  4.31990
H   0.16297 -1.13836  4.22465
C   2.74280 -1.58824  3.33714
H   2.09985 -2.47742  3.23831
H   3.23803 -1.62928  4.32372
H   3.51745 -1.62400  2.55512
```

E-int$_{Bu}$

SCF (BP86) Energy = -2500.27547793
Enthalpy 0K = -2498.990391
Enthalpy 298K = -2498.908352
Free Energy 298K = -2499.107300
Lowest Frequency = 11.0064 cm^{-1}
Second Frequency = 18.1707 cm^{-1}
SCF (BP86-D3BJ) Energy = -2500.68298076
SCF (C6H6) Energy = -2500.27938816
SCF (BS2) Energy = -3312.51147397

```
Cu  1.76641 -0.36063 -0.23311
Si -4.09456  2.24609  0.04349
Al -1.72313  0.03895 -0.16285
Si -3.87525 -1.21750  1.91847
N   4.26902  1.05279 -0.85955
N  -2.34314  1.77880 -0.05089
N   4.73646 -1.05743 -0.63610
N  -2.74260 -1.34223  0.51679
C   3.68438 -0.17526 -0.66919
H   3.77987  0.12765  2.10695
C  -1.58185  2.84971 -2.76212
H  -2.14356  1.93043 -2.51917
C  -1.09384  3.45119 -1.44221
C  -2.57187 -2.65594 -0.08335
C  -0.94754  3.55364  1.01881
C  -5.28274  0.04897  1.64066
H  -5.83367 -0.24283  0.72604
H  -5.98620 -0.15531  2.47524
C  -4.94230  1.55652  1.61189
H  -4.33230  1.83748  2.49114
H  -5.87582  2.14969  1.70481
C  -1.44008  2.91108 -0.16256
C  -2.92394 -0.70921  3.48958
H  -2.35987  0.22719  3.33871
H  -2.20215 -1.48532  3.79153
H  -3.62063 -0.54517  4.33090
C   0.18398  5.24114 -0.35440
```

H 0.79269 6.14903 -0.42804
C 4.51066 -2.50407 -0.39373
H 3.41058 -2.58833 -0.44016
C -1.60122 -3.58073 0.41672
C -4.26566 4.14194 0.07538
H -3.80507 4.60722 0.95952
H -5.34126 4.39310 0.07738
H -3.81033 4.60866 -0.81237
C 3.43788 2.28641 -0.88070
H 2.40632 1.88714 -0.87750
C -0.14731 4.70636 0.89504
H 0.21489 5.19902 1.80472
C -0.66702 -3.26225 1.58485
H -0.76997 -2.18672 1.81020
C 5.66768 0.94849 -0.93235
C 0.77670 -0.78195 -1.93934
C -0.40032 2.45692 -3.67476
H 0.19441 3.34000 -3.96900
H -0.76839 1.98662 -4.60416
H 0.26707 1.74599 -3.16236
C 5.96655 -0.39100 -0.79050
C -2.53362 3.80563 -3.51902
H -3.41401 4.07902 -2.91457
H -2.89371 3.33596 -4.45177
H -2.01823 4.74263 -3.79570
C -0.28615 4.60474 -1.50795
H -0.03908 5.02294 -2.49090
C -1.25200 3.04322 2.42900
H -1.83456 2.11203 2.31626
C -0.10271 -0.41660 -1.08108
C -3.40254 -3.05168 -1.17999
C 3.62269 3.10569 -2.16696
H 2.82393 3.86389 -2.21721
H 3.54421 2.46514 -3.06059
H 4.58851 3.63642 -2.19643
C -4.47807 -2.13318 -1.75764
H -4.54956 -1.26893 -1.07596
C -1.48945 -4.85093 -0.18531
H -0.75053 -5.55793 0.20852
C -5.07486 1.65887 -1.48229
H -4.69154 2.12706 -2.40366
H -6.13428 1.95289 -1.37167
H -5.04683 0.56780 -1.62137
C 0.04505 2.70973 3.19636
H 0.64814 3.61884 3.37290
H 0.67204 1.98772 2.64835
H -0.19645 2.28267 4.18617
C 7.31216 -1.04977 -0.82716
H 7.46353 -1.74593 0.01446
H 8.10209 -0.28501 -0.76196
H 7.48258 -1.61492 -1.76106
C -4.07728 -1.60585 -3.15395
H -3.12331 -1.05183 -3.11843
H -4.84990 -0.92902 -3.56030
H -3.94925 -2.43890 -3.86793
C -4.73912 -2.89069 2.19002
H -5.29355 -2.86521 3.14446
H -4.03369 -3.73493 2.22426
H -5.46385 -3.09798 1.38515
C 0.81192 -3.52498 1.22767
H 1.01206 -4.60333 1.08998
H 1.46993 -3.15338 2.03045
H 1.08808 -2.99596 0.30029
C 5.11316 -3.39053 -1.49710
H 6.21095 -3.45662 -1.42961
H 4.84563 -3.02051 -2.50028
H 4.71654 -4.41445 -1.39424
C -1.04244 -4.05885 2.85688
H -2.07237 -3.85171 3.19050
H -0.35911 -3.80560 3.68714
H -0.96354 -5.14638 2.67800
C 3.61048 3.11148 0.40351
H 4.61332 3.56572 0.48040
H 3.43160 2.47709 1.28596
H 2.86627 3.92506 0.40907
C -2.10018 4.03626 3.25744
H -2.27619 3.63466 4.27114
H -3.08279 4.23408 2.79998
H -1.58194 5.00520 3.37006
C -5.86936 -2.80183 -1.81390
H -5.88994 -3.64662 -2.52479
H -6.63224 -2.07538 -2.14564
H -6.17357 -3.19119 -0.82781
C 4.94564 -2.92365 1.01905
H 4.64874 -3.97094 1.19805
H 4.45283 -2.28918 1.77135
H 6.03829 -2.85964 1.15704
C -3.23505 -4.32489 -1.75777
H -3.87087 -4.61300 -2.60299
C 6.60796 2.09205 -1.16426
H 6.55114 2.48072 -2.19672
H 7.64559 1.76307 -0.99652
H 6.41761 2.93626 -0.48126
C -2.28930 -5.23024 -1.26748
H -2.17980 -6.22169 -1.72013
C 2.79109 -0.07991 2.56053
C 1.71623 -0.21924 1.74110
H 0.76763 -0.41745 2.28004
C 2.88162 -0.16104 4.09278
C 3.32808 1.22114 4.63877
H 4.28202 1.53710 4.17890
H 2.57482 1.99622 4.41960
H 3.47841 1.18651 5.73411
C 3.96614 -1.20262 4.47185
H 4.12120 -1.23355 5.56643
H 3.67667 -2.21588 4.14184
H 4.93633 -0.95591 4.00339
C 1.55040 -0.57043 4.75144
H 0.74810 0.15222 4.52975
H 1.21737 -1.56021 4.39291
H 1.66324 -0.62628 5.84921
C 1.10572 -1.37895 -3.28155
C 1.54254 -2.85200 -3.06875
H 2.45069 -2.90738 -2.44822
H 0.74698 -3.43067 -2.57104
H 1.76047 -3.32322 -4.04426
C -0.16242 -1.36874 -4.17331
H 0.06040 -1.81730 -5.15843
H -0.96976 -1.95250 -3.70221
H -0.52541 -0.34169 -4.33688
C 2.24592 -0.60042 -3.97867
H 1.94785 0.44115 -4.18608
H 2.49762 -1.08114 -4.94082
H 3.14719 -0.58028 -3.34480

TS(E2)$_{Bu}$
SCF (BP86) Energy = -2500.27386835
Enthalpy 0K = -2498.988578
Enthalpy 298K = -2498.907484
Free Energy 298K = -2499.101802
Lowest Frequency = -32.9543 cm^{-1}
Second Frequency = 16.5960 cm^{-1}
SCF (BP86-D3BJ) Energy = -2500.69392376
SCF (C6H6) Energy = -2500.27768424
SCF (BS2) Energy = -3312.50762595

Cu 1.47473 -0.35453 -0.30516
Si -3.80346 2.24768 -0.21133
Al -1.42834 0.02984 -0.18502
Si -3.76074 -1.14569 1.78024
N 4.07876 1.06671 -0.48012
N -2.04720 1.78946 -0.12229
N 4.49412 -1.06167 -0.36954
N -2.50423 -1.32198 0.49759
C 3.45829 -0.15752 -0.42983
H 3.07777 -0.13512 2.13665
C -1.06683 2.79845 -2.80254
H -1.57770 1.84454 -2.58475
C -0.73607 3.46254 -1.46279
C -2.38465 -2.65086 -0.08360
C -0.84353 3.67361 0.99243
C -5.14327 0.10409 1.33949
H -5.61393 -0.22035 0.39179
H -5.91534 -0.07501 2.11737

```
C  -4.80279  1.61039  1.28928
H  -4.28224  1.92390  2.21378
H  -5.74071  2.20375  1.26831
C  -1.17903  2.94963 -0.19940
C  -2.97810 -0.58835  3.42438
H  -2.38961  0.33720  3.30580
H  -2.30378 -1.36118  3.82748
H  -3.76109 -0.39150  4.17828
C   0.30617  5.38204 -0.34048
H   0.85609  6.32786 -0.39719
C   4.24074 -2.51991 -0.26122
H   3.15159 -2.59030 -0.42830
C  -1.54489 -3.64465  0.51118
C  -3.98768  4.14576 -0.26191
H  -3.54065  4.66385  0.59844
H  -5.06663  4.38230 -0.28361
H  -3.53204  4.57346 -1.16919
C   3.28427  2.32479 -0.50032
H   2.24381  1.96030 -0.58947
C  -0.11295  4.87414  0.89363
H   0.11850  5.42995  1.80978
C  -0.66002 -3.36855  1.72635
H  -0.73653 -2.29250  1.95555
C   5.47628  0.93890 -0.44368
C   0.82111 -0.89239 -2.21547
C   0.20721  2.47857 -3.61328
H   0.77235  3.39459 -3.86129
H  -0.05944  1.99210 -4.56841
H   0.87011  1.79962 -3.05433
C   5.74200 -0.41271 -0.37053
C  -2.01359  3.67203 -3.66021
H  -2.95579  3.90212 -3.13754
H  -2.26617  3.15661 -4.60413
H  -1.53710  4.63338 -3.92324
C  -0.00205  4.66645 -1.50176
H   0.30911  5.06416 -2.47508
C  -1.25250  3.20627  2.39180
H  -1.73772  2.22166  2.27305
C  -0.19648 -0.55695 -1.52743
C  -3.14592 -3.00635 -1.24318
C   3.59195  3.19615 -1.72717
H   2.82172  3.98117 -1.79818
H   3.56236  2.60166 -2.65492
H   4.57319  3.69361 -1.65714
C  -4.07167 -2.01528 -1.94162
H  -4.07911 -1.10848 -1.31531
C  -1.50082 -4.93890 -0.04620
H  -0.86336 -5.69547  0.42614
C  -4.67236  1.64859 -1.80277
H  -4.10537  1.93245 -2.70371
H  -5.66207  2.13690 -1.86268
H  -4.83985  0.56264 -1.83659
C  -0.01507  3.02813  3.29554
H   0.50635  3.98818  3.46053
H   0.69724  2.31773  2.84785
H  -0.31528  2.64673  4.28769
C   7.07783 -1.09173 -0.33357
H   7.14182 -1.85449  0.45992
H   7.86576 -0.34879 -0.13315
H   7.33044 -1.58384 -1.28974
C  -3.53293 -1.62353 -3.33556
H  -2.52101 -1.19151 -3.25937
H  -4.19123 -0.87954 -3.81862
H  -3.47642 -2.50431 -4.00034
C  -4.66217 -2.80317  2.03618
H  -5.33861 -2.71625  2.90469
H  -3.97504 -3.64337  2.21805
H  -5.27378 -3.06514  1.15692
C   0.81977 -3.67063  1.40850
H   0.98351 -4.74684  1.21795
H   1.46381 -3.37101  2.25190
H   1.14569 -3.10518  0.51966
C   4.95321 -3.33231 -1.35657
H   6.03461 -3.42922 -1.17038
H   4.80684 -2.88315 -2.35197
H   4.53298 -4.35173 -1.37835
C  -1.10184 -4.16494  2.97569
H  -2.13184 -3.91846  3.28146
H  -0.43527 -3.94675  3.82947
H  -1.06075 -5.25360  2.79135
C   3.38707  3.08605  0.82972
H   4.38944  3.51743  0.99310
H   3.14165  2.41781  1.66974
H   2.65721  3.91201  0.82395
C  -2.25787  4.15697  3.08301
H  -2.48433  3.79564  4.10192
H  -3.21094  4.23303  2.53659
H  -1.84209  5.17613  3.17743
C  -5.52559 -2.52662 -2.04558
H  -5.59832 -3.42050 -2.69013
H  -6.17769 -1.75065 -2.48516
H  -5.93475 -2.79600 -1.05721
C   4.53296 -3.04868  1.15165
H   4.21216 -4.10134  1.22386
H   3.97757 -2.46728  1.90251
H   5.60789 -3.00985  1.39663
C  -3.05035 -4.30714 -1.77344
H  -3.63616 -4.56038 -2.66499
C   6.45463  2.07181 -0.51870
H   6.49960  2.52121 -1.52658
H   7.46614  1.70784 -0.27922
H   6.21967  2.87862  0.19423
C  -2.24119 -5.28106 -1.18132
H  -2.18968 -6.29324 -1.59711
C   2.04541 -0.17650  2.53000
C   1.01652 -0.12798  1.63714
H   0.02422 -0.16976  2.13438
C   2.04535 -0.31421  4.06186
C   2.74510  0.92620  4.67984
H   3.75853  1.05618  4.25854
H   2.17705  1.85019  4.48686
H   2.85012  0.81166  5.77465
C   2.88857 -1.55968  4.44342
H   2.96361 -1.65931  5.54207
H   2.43745 -2.48696  4.04955
H   3.91670 -1.48267  4.04582
C   0.63583 -0.47403  4.65821
H  -0.00862  0.38795  4.42342
H   0.14093 -1.37908  4.26591
H   0.68963 -0.56671  5.75778
C   1.40373 -1.46863 -3.47445
C   1.64690 -2.98490 -3.24961
H   2.36289 -3.15771 -2.43119
H   0.70590 -3.49810 -2.99355
H   2.05689 -3.43932 -4.16962
C   0.37926 -1.30302 -4.62800
H   0.78521 -1.74015 -5.55819
H  -0.56601 -1.81494 -4.38718
H   0.15908 -0.23979 -4.81287
C   2.72859 -0.77261 -3.86350
H   2.56517  0.29875 -4.06920
H   3.14316 -1.23607 -4.77641
H   3.47117 -0.85252 -3.05417
```

E_{Bu}

SCF (BP86) Energy = -2500.29882861
Enthalpy 0K = -2499.012939
Enthalpy 298K = -2498.931179
Free Energy 298K = -2499.127426
Lowest Frequency = 16.8691 cm^{-1}
Second Frequency = 22.0538 cm^{-1}
SCF (BP86-D3BJ) Energy = -2500.72332069
SCF (C6H6) Energy = -2500.30357021
SCF (BS2) Energy = -3312.52982717

```
Si -3.48040 -1.58987  1.88115
Al -1.20383  0.05093  0.20237
Si -3.76665  2.14214  0.60356
N  -2.04860  1.77702  0.22248
N  -2.40445 -1.42142  0.45518
C  -1.26476  2.94412 -0.09528
C  -3.12803 -2.64070 -1.60380
C  -2.33394 -2.57318 -0.41139
C  -0.54667  3.65458  0.93039
```

C -1.23232 3.47426 -1.43220
C -4.32276 1.26272 2.21361
H -3.51685 1.34658 2.96734
H -5.15721 1.87952 2.60679
C 0.25760 -1.23971 -2.12728
C -0.55334 -3.71346 1.10675
H -0.66688 -2.74702 1.62585
C -1.48251 -3.68923 -0.10893
C -4.06916 -1.50927 -1.99962
H -3.97954 -0.75240 -1.20502
C 0.15123 -0.12936 1.72994
C -2.00750 2.83945 -2.59013
H -2.47252 1.91807 -2.20042
C -0.51486 4.65646 -1.70288
H -0.51513 5.05367 -2.72529
C -4.48224 -3.20885 1.76626
H -5.18656 -3.18207 0.91804
H -5.07201 -3.34056 2.69047
H -3.84724 -4.09854 1.63413
C 0.08938 -0.12729 -1.35159
H 0.60403 0.77975 -1.72339
C 1.05844 -0.35104 2.58203
C -1.45620 -4.80498 -0.97033
H -0.80701 -5.65283 -0.71914
C -0.50633 3.19281 2.39274
H -0.91579 2.16878 2.42078
C -3.05373 -3.77070 -2.44082
H -3.66969 -3.79847 -3.34793
C -2.22903 -4.85829 -2.13508
H -2.19253 -5.73539 -2.79044
C -2.52014 -1.57753 3.53580
H -1.82079 -2.42573 3.61594
H -3.22766 -1.63430 4.38243
H -1.93716 -0.64796 3.64119
C -3.65432 -0.84569 -3.33032
H -2.61920 -0.47018 -3.27952
H -4.32050 0.00439 -3.56277
H -3.71485 -1.55737 -4.17388
C -5.07731 1.70292 -0.72441
H -6.02070 2.20448 -0.43902
H -5.29014 0.62766 -0.80500
H -4.79938 2.07045 -1.72445
C 0.14048 4.84260 0.60517
H 0.65098 5.39466 1.40356
C -4.79078 -0.20436 2.06248
H -5.51210 -0.29449 1.22888
H -5.35870 -0.51037 2.96695
C 0.92519 -3.83483 0.67291
H 1.11182 -4.77410 0.12170
H 1.58831 -3.82687 1.55614
H 1.20951 -2.99484 0.01457
C -4.01936 4.02975 0.79653
H -3.30232 4.53235 1.45978
H -5.03643 4.20668 1.19006
H -3.95439 4.53106 -0.18363
C -5.54155 -1.97294 -2.06576
H -5.69277 -2.72776 -2.85841
H -6.20803 -1.12050 -2.28917
H -5.87065 -2.42223 -1.11349
C -0.90490 -4.84755 2.09638
H -1.93735 -4.75827 2.47158
H -0.22557 -4.83022 2.96849
H -0.80773 -5.84081 1.62224
C -1.09338 2.44823 -3.77121
H -0.59366 3.33052 -4.21055
H -1.68457 1.97049 -4.57283
H -0.31622 1.73660 -3.45300
C 0.93981 3.14412 2.92916
H 1.39606 4.14919 2.97637
H 1.57765 2.50145 2.30232
H 0.95043 2.73901 3.95661
C 0.16579 5.35226 -0.69794
H 0.69248 6.28662 -0.92244
C -3.12556 3.77688 -3.10593
H -3.81575 4.08154 -2.30298
H -3.71786 3.27793 -3.89401
H -2.70014 4.69793 -3.54373
C -1.35272 4.07732 3.33969
H -1.21080 3.75793 4.38799
H -2.42845 4.01522 3.11882
H -1.05197 5.13847 3.27042
N 4.65528 -0.88621 -0.25699
N 4.09966 1.18109 -0.64881
C 5.78154 -0.27174 -0.82646
C 5.42695 1.03957 -1.07625
C 3.60894 0.00089 -0.14240
Cu 1.83053 -0.22495 0.59044
C 4.56154 -2.26793 0.28248
H 3.48969 -2.36242 0.53247
C 6.24723 2.11421 -1.72265
H 5.90092 2.34307 -2.74554
H 7.29667 1.78955 -1.79679
H 6.23525 3.05509 -1.14909
C 7.08059 -0.95268 -1.13465
H 7.48137 -1.51170 -0.27304
H 7.83564 -0.20154 -1.41353
H 6.99433 -1.65830 -1.97955
C 3.28717 2.43028 -0.61894
H 2.29430 2.07453 -0.28624
C 5.37563 -2.42110 1.57942
H 6.46150 -2.35950 1.39684
H 5.17161 -3.40819 2.02724
H 5.10312 -1.64688 2.31356
C 4.90986 -3.34944 -0.75330
H 4.40766 -3.16319 -1.71515
H 4.56719 -4.32722 -0.37672
H 5.99432 -3.42706 -0.93087
C 3.80998 3.41587 0.43809
H 3.92354 2.92223 1.41647
H 3.08003 4.23417 0.54728
H 4.77881 3.85991 0.15342
C 3.11536 3.07363 -2.00275
H 4.02726 3.59178 -2.34188
H 2.30528 3.81862 -1.94038
H 2.83078 2.32374 -2.75699
H -0.27298 -2.15885 -1.83483
C 1.79108 -0.62504 3.85618
C 2.14574 -2.13275 3.93621
H 1.23731 -2.75447 3.88295
H 2.81238 -2.42608 3.10895
H 2.66026 -2.34778 4.88975
C 3.09071 0.21370 3.94701
H 3.63720 -0.04245 4.87183
H 3.74693 0.01777 3.08255
H 2.86702 1.29186 3.96471
C 0.85972 -0.26606 5.04599
H -0.06485 -0.86433 5.01825
H 1.38053 -0.46965 5.99852
H 0.58060 0.79952 5.02431
C 1.03326 -1.43868 -3.43083
C 2.02709 -0.30486 -3.73897
H 2.57044 -0.51437 -4.67780
H 1.51192 0.66265 -3.86570
H 2.76769 -0.19614 -2.92806
C 0.00118 -1.55663 -4.58591
H 0.51115 -1.81299 -5.53303
H -0.74286 -2.34285 -4.37222
H -0.54001 -0.60700 -4.73137
C 1.79690 -2.78273 -3.32978
H 1.10360 -3.61675 -3.12489
H 2.33250 -3.00329 -4.27107
H 2.53526 -2.75113 -2.51056

INT(E-P)$_{Bu}$
SCF (BP86) Energy = -2734.86622080
Enthalpy 0K = -2733.443331
Enthalpy 298K = -2733.352098
Free Energy 298K = -2733.568712
Lowest Frequency = 13.6405 cm^{-1}
Second Frequency = 18.7265 cm^{-1}
SCF (BP86-D3BJ) Energy = -2735.33059650
SCF (C6H6) Energy = -2734.87292209
SCF (BS2) Energy = -3547.15342883

Si	4.22226	-0.10681	-2.11919
Al	1.67176	0.22220	-0.04730
Si	3.28907	3.14432	-0.16680
N	1.78587	2.15631	-0.13093
N	3.23208	-0.68572	-0.73847
C	0.58115	2.92158	0.06769
C	4.84119	-1.81415	0.83444
C	3.77115	-1.88167	-0.12172
C	-0.20534	3.35032	-1.05756
C	0.16869	3.34959	1.37766
C	4.37563	2.82064	-1.70843
H	3.73222	2.93718	-2.60191
H	5.06193	3.69433	-1.73026
C	0.99415	-1.75158	2.13087
H	0.67349	-2.42571	1.31593
C	2.12754	-3.41662	-1.44551
H	1.60769	-2.44961	-1.56259
C	3.31136	-3.18778	-0.50183
C	5.37605	-0.49366	1.38146
H	4.82640	0.30783	0.86408
C	-0.02787	-0.32927	-1.16315
C	0.99797	3.07360	2.63503
H	1.82078	2.40289	2.33742
C	-0.99088	4.13662	1.52649
H	-1.28026	4.46646	2.53158
C	5.55731	-1.38535	-2.59888
H	6.31241	-1.50409	-1.80489
H	6.07462	-1.03818	-3.51115
H	5.14136	-2.38471	-2.80031
C	1.37759	-0.49039	1.80950
H	1.68436	0.13307	2.66798
C	-0.74636	-0.78483	-2.10903
C	3.95918	-4.33702	-0.00409
H	3.61634	-5.32388	-0.33585
C	0.20850	3.03920	-2.49698
H	0.93839	2.21337	-2.44177
C	5.44069	-2.99382	1.31790
H	6.26369	-2.91048	2.03800
C	5.02642	-4.25816	0.89345
H	5.51845	-5.16501	1.26157
C	3.15061	0.22466	-3.66362
H	2.85916	-0.71850	-4.15318
H	3.71827	0.82474	-4.39723
H	2.22891	0.77701	-3.41752
C	5.09723	-0.36954	2.89687
H	4.01772	-0.45263	3.10531
H	5.45382	0.60086	3.28636
H	5.61254	-1.16576	3.46359
C	4.38244	2.92681	1.39501
H	4.17118	3.72421	2.12646
H	5.45358	2.99185	1.13193
H	4.21861	1.96484	1.90076
C	-1.35091	4.14475	-0.85275
H	-1.91919	4.49134	-1.72460
C	5.19015	1.51490	-1.79401
H	5.81354	1.38864	-0.88999
H	5.91179	1.57783	-2.63631
C	1.13147	-4.44169	-0.85242
H	1.57628	-5.44965	-0.78048
H	0.23243	-4.53658	-1.48511
H	0.80637	-4.14989	0.15977
C	2.90210	5.02683	-0.24666
H	3.01789	5.42604	-1.26807
H	3.62854	5.56183	0.39060
H	1.89040	5.28783	0.10051
C	6.88372	-0.29567	1.10628
H	7.49400	-1.04997	1.63475
H	7.21323	0.69899	1.45698
H	7.11975	-0.37265	0.03183
C	2.58325	-3.89204	-2.84520
H	3.21419	-3.14028	-3.34622
H	1.71529	-4.09592	-3.49942
H	3.16982	-4.82568	-2.77218
C	0.19384	2.37834	3.75455
H	-0.59793	3.04263	4.14822
H	0.85619	2.12708	4.60226
H	-0.28393	1.45261	3.39973

C	-0.97552	2.58781	-3.37226
H	-1.68684	3.41268	-3.56202
H	-1.51993	1.76028	-2.89231
H	-0.61633	2.24318	-4.35879
C	-1.75646	4.53821	0.42802
H	-2.63844	5.17446	0.56490
C	1.61174	4.37928	3.19676
H	2.20385	4.91833	2.44108
H	2.26853	4.16083	4.05795
H	0.81962	5.06591	3.54703
C	0.89733	4.24602	-3.17535
H	1.18298	3.99683	-4.21343
H	1.80465	4.55463	-2.63753
H	0.21616	5.11572	-3.21099
N	-4.63771	-1.85359	-0.31300
N	-4.91473	0.18180	-1.03086
C	-6.02220	-1.66694	-0.45214
C	-6.19818	-0.37504	-0.90921
C	-3.94675	-0.71655	-0.65412
Cu	-2.02230	-0.42074	-0.53288
C	-3.92149	-3.07581	0.14353
H	-2.86300	-2.82333	-0.05369
C	-7.48150	0.31630	-1.25814
H	-7.54190	1.33128	-0.83322
H	-8.33345	-0.25605	-0.85909
H	-7.62919	0.40256	-2.34917
C	-7.07019	-2.70359	-0.18032
H	-7.11285	-3.47691	-0.96758
H	-8.06243	-2.22787	-0.13819
H	-6.91347	-3.21691	0.78234
C	-4.55317	1.55268	-1.48668
H	-3.44996	1.51320	-1.54250
C	-4.28172	-4.31658	-0.69064
H	-5.27735	-4.71661	-0.44115
H	-3.54722	-5.11204	-0.48327
H	-4.25115	-4.09896	-1.77058
C	-4.07229	-3.30646	1.65500
H	-3.71734	-2.42684	2.21367
H	-3.45793	-4.17144	1.95551
H	-5.11505	-3.52296	1.94294
C	-5.09249	1.86828	-2.89116
H	-4.87687	1.05079	-3.59710
H	-4.59546	2.77837	-3.26487
H	-6.17730	2.06135	-2.89246
C	-4.92343	2.62338	-0.45058
H	-6.01424	2.72936	-0.32461
H	-4.52274	3.59614	-0.77733
H	-4.46850	2.39021	0.52376
C	-1.92378	0.00828	1.74460
C	-2.83546	0.33771	2.51277
H	-0.87203	-0.25328	1.54591
C	0.95195	-2.44417	3.49495
C	-0.46961	-3.02499	3.70790
H	-1.22599	-2.22159	3.73919
H	-0.73999	-3.71539	2.88807
H	-0.52495	-3.59146	4.65559
C	1.96990	-3.61485	3.46630
H	1.74486	-4.32043	2.64779
H	2.99560	-3.24489	3.30478
H	1.94244	-4.17710	4.41827
C	1.29287	-1.49520	4.65930
H	1.23431	-2.03407	5.62177
H	2.31510	-1.09122	4.56503
H	0.59483	-0.64154	4.70306
C	-3.91950	0.73437	3.43152
C	-5.30259	0.31790	2.86233
H	-6.09511	0.62584	3.56669
H	-5.49787	0.79669	1.89005
H	-5.36600	-0.77345	2.72651
C	-3.68882	0.01591	4.79205
H	-4.47891	0.31249	5.50414
H	-3.71957	-1.07989	4.67508
H	-2.71235	0.28958	5.22272
C	-3.88383	2.27054	3.65312
H	-2.91895	2.58059	4.08386
H	-4.01953	2.81664	2.70601
H	-4.69032	2.56432	4.34779

C -1.19071 -1.39607 -3.41286
C -2.47770 -0.74263 -3.97379
H -2.33086 0.32671 -4.18776
H -2.75615 -1.24533 -4.91686
H -3.31224 -0.84984 -3.26225
C -0.04775 -1.22839 -4.44538
H 0.86980 -1.72761 -4.09939
H -0.35038 -1.68057 -5.40675
H 0.18386 -0.16572 -4.61697
C -1.46962 -2.90618 -3.21220
H -2.26554 -3.06317 -2.46596
H -1.79527 -3.35567 -4.16727
H -0.56614 -3.43232 -2.87195

TS1(E-P)$_{Bu}$
SCF (BP86) Energy = -2734.85145687
Enthalpy 0K = -2733.431147
Enthalpy 298K = -2733.341434
Free Energy 298K = -2733.552467
Lowest Frequency = -796.8211 cm^{-1}
Second Frequency = 14.6666 cm^{-1}
SCF (BP86-D3BJ) Energy = -2735.31934963
SCF (C6H6) Energy = -2734.85662956
SCF (BS2) Energy = -3547.13808802

Si 4.00382 -0.38518 -2.18058
Al 1.48280 0.22512 -0.22405
Si 3.37154 2.97717 -0.36645
N 1.78550 2.12400 -0.24723
N 3.03097 -0.81059 -0.72074
C 0.67187 3.02985 -0.05563
C 4.65952 -2.01541 0.76503
C 3.50002 -2.02284 -0.08694
C -0.11170 3.47273 -1.17441
C 0.37546 3.58319 1.23772
C 4.37342 2.52455 -1.93300
H 3.70071 2.64246 -2.80419
H 5.10786 3.35343 -2.02020
C 0.92636 -1.62179 2.15671
H 0.60248 -2.39963 1.44605
C 1.66073 -3.46016 -1.25921
H 1.20079 -2.46554 -1.38606
C 2.89834 -3.29777 -0.37227
C 5.36385 -0.72786 1.18940
H 4.85463 0.09815 0.67034
C -0.12894 -0.30606 -1.36076
C 1.21123 3.27926 2.48508
H 1.94707 2.50835 2.20075
C -0.68351 4.50116 1.38067
H -0.89103 4.91916 2.37303
C 5.19956 -1.79501 -2.65630
H 5.99256 -1.93870 -1.90528
H 5.68304 -1.53447 -3.61490
H 4.69358 -2.76445 -2.78216
C 0.88086 -0.31572 1.73377
H 1.16952 0.42833 2.49157
C -0.95235 -0.74790 -2.22728
C 3.48181 -4.47977 0.12927
H 3.02684 -5.44332 -0.12764
C 0.18594 3.03173 -2.60918
H 0.83963 2.14559 -2.54240
C 5.19698 -3.22648 1.24423
H 6.09096 -3.19265 1.87842
C 4.63087 -4.46254 0.92376
H 5.07291 -5.39518 1.29057
C 2.88161 -0.04885 -3.68502
H 2.50889 -0.99213 -4.11432
H 3.45362 0.47812 -4.46961
H 2.00617 0.57063 -3.43103
C 5.22602 -0.49165 2.71018
H 4.16597 -0.43058 3.00750
H 5.71979 0.44984 3.00896
H 5.68982 -1.31176 3.28730
C 4.49097 2.71143 1.16855
H 4.40722 3.56546 1.86053
H 5.55115 2.62883 0.86923
H 4.22781 1.80395 1.73067
C -1.15488 4.39915 -0.97527
H -1.72887 4.74546 -1.84283
C 5.10349 1.16946 -1.99234
H 5.76908 1.04940 -1.11861
H 5.77630 1.14123 -2.87580
C 0.60804 -4.40203 -0.62795
H 0.95443 -5.45057 -0.60394
H -0.32707 -4.38526 -1.21376
H 0.36198 -4.11399 0.40791
C 3.14202 4.87614 -0.51413
H 3.06779 5.20369 -1.56370
H 4.03727 5.36195 -0.08561
H 2.25791 5.26179 0.01540
C 6.85785 -0.69761 0.79456
H 7.43344 -1.47899 1.32228
H 7.30613 0.27631 1.06126
H 7.00310 -0.85206 -0.28716
C 2.03659 -3.99185 -2.66293
H 2.68946 -3.28843 -3.20330
H 1.13458 -4.15745 -3.27979
H 2.57038 -4.95650 -2.58806
C 0.36065 2.73141 3.65221
H -0.29646 3.51789 4.06626
H 1.01058 2.38805 4.47740
H -0.27731 1.89354 3.32870
C -1.08674 2.63382 -3.38068
H -1.74022 3.50340 -3.57599
H -1.66218 1.88135 -2.82024
H -0.81967 2.20604 -4.36381
C -1.45504 4.91284 0.29036
H -2.26286 5.64155 0.42119
C 1.98121 4.52724 2.98115
H 2.62630 4.95937 2.20116
H 2.61568 4.27129 3.84882
H 1.27866 5.31655 3.30429
C 0.93519 4.12287 -3.40918
H 1.11959 3.78592 -4.44536
H 1.90601 4.37070 -2.95598
H 0.33987 5.05265 -3.45676
N -4.58277 -1.90483 -0.15960
N -4.95160 0.11433 -0.87343
C -5.97506 -1.71797 -0.11779
C -6.20915 -0.43507 -0.57141
C -3.94040 -0.77501 -0.60412
Cu -2.01072 -0.38458 -0.50691
C -3.80609 -3.09725 0.26799
H -2.78503 -2.86028 -0.08365
C -7.52445 0.25892 -0.75738
H -7.52444 1.27673 -0.33355
H -8.32020 -0.30689 -0.24781
H -7.81201 0.34113 -1.82067
C -6.97637 -2.74808 0.30928
H -7.10565 -3.54943 -0.43989
H -7.96035 -2.27414 0.45116
H -6.70452 -3.22674 1.26473
C -4.64138 1.48973 -1.34677
H -3.56407 1.42830 -1.58498
C -4.26395 -4.38967 -0.42845
H -5.21699 -4.77032 -0.02785
H -3.50423 -5.17197 -0.26567
H -4.37378 -4.24411 -1.51555
C -3.75147 -3.22213 1.79848
H -3.32814 -2.30360 2.23454
H -3.10359 -4.07061 2.07624
H -4.74740 -3.40581 2.23652
C -5.39386 1.86469 -2.63363
H -5.31698 1.06842 -3.39149
H -4.94216 2.77799 -3.05456
H -6.45940 2.07909 -2.45318
C -4.80480 2.52498 -0.22489
H -5.85810 2.64843 0.08073
H -4.43222 3.50323 -0.57008
H -4.20434 2.22647 0.64796
C -1.81373 0.20053 1.40853
C -2.48329 0.55568 2.40618
H -0.38403 -0.06020 1.35007
C 1.18212 -2.17609 3.54966

C -0.15567 -2.84589 3.98231
H -0.98200 -2.11551 3.99500
H -0.43137 -3.66569 3.29589
H -0.05048 -3.27351 4.99555
C   2.27997 -3.26596 3.49648
H   2.03831 -4.04526 2.75453
H   3.25529 -2.84070 3.21477
H   2.37903 -3.74995 4.48494
C   1.55728 -1.09129 4.57651
H   1.70857 -1.54840 5.56984
H   2.49252 -0.57605 4.30069
H   0.76481 -0.32982 4.67075
C -3.32657 0.94995 3.55913
C -4.73901 0.31783 3.40426
H -5.38268 0.63035 4.24634
H -5.21627 0.63847 2.46389
H -4.68349 -0.78275 3.40201
C -2.69152 0.43716 4.88123
H -3.32670 0.72393 5.73855
H -2.59620 -0.66142 4.87594
H -1.69012 0.87059 5.03552
C -3.47388 2.49377 3.63384
H -2.49207 2.98151 3.72553
H -3.96421 2.89119 2.73086
H -4.08767 2.76913 4.51037
C -1.47896 -1.34340 -3.50770
C -2.73299 -0.59609 -4.02485
H -2.51035 0.45513 -4.26262
H -3.09618 -1.08643 -4.94560
H -3.53620 -0.62672 -3.27161
C -0.37483 -1.28766 -4.59244
H   0.50619 -1.87249 -4.28662
H -0.76029 -1.70915 -5.53814
H -0.05006 -0.25207 -4.78032
C -1.87428 -2.82210 -3.26674
H -2.66943 -2.89046 -2.50692
H -2.25058 -3.26598 -4.20583
H -1.01134 -3.41498 -2.92652

INT2(E-P)$_{Bu}$
SCF (BP86) Energy = -2499.05378531
Enthalpy 0K = -2497.790282
Enthalpy 298K = -2497.708320
Free Energy 298K = -2497.907964
Lowest Frequency = 10.9348 cm^{-1}
Second Frequency = 14.1376 cm^{-1}
SCF (BP86-D3BJ) Energy = -2499.45578811
SCF (C6H6) Energy = -2499.05830424
SCF (BS2) Energy = -3311.29069158

Si -4.05835 2.20832 -0.11833
Al -1.65237 0.04787 -0.19686
Si -3.85680 -1.22241 1.80119
N -2.67393 -1.34708 0.44095
N -2.29419 1.78095 -0.10505
C -2.47244 -2.66426 -0.14069
C -1.00134 3.59381 1.02780
C -1.39757 2.92231 -0.17357
C -3.28068 -3.08979 -1.24287
C -1.49456 -3.56505 0.38786
C -5.29310 -0.01334 1.42776
H -5.78216 -0.33850 0.48949
H -6.03561 -0.22565 2.22564
C -1.38481 2.84138 -2.77717
H -1.94726 1.91519 -2.56443
C -0.95592 3.43757 -1.43435
C -1.45372 3.13939 2.41649
H -2.14784 2.29415 2.26906
C -0.07185 -0.41646 -1.18267
C -0.57091 -3.20933 1.55382
H -0.65788 -2.12557 1.74365
C -1.36278 -4.84685 -0.18540
H -0.62014 -5.53644 0.23139
C -4.27489 4.09878 -0.07934
H -4.00780 4.53877 0.89341
H -5.33732 4.32919 -0.27446
H -3.66964 4.60479 -0.84766
C   0.83732 -0.78385 -2.00005
C -0.12452 4.57617 -1.46010
H   0.19644 4.97577 -2.42932
C -4.34846 -2.19171 -1.86518
H -4.42910 -1.30389 -1.21583
C -0.16504 4.72380 0.94415
H   0.13439 5.22975 1.86917
C   0.27681 5.22205 -0.28600
H   0.91185 6.11372 -0.33022
C -4.91930 1.60829 -1.70965
H -4.53268 2.14281 -2.59276
H -6.00322 1.81193 -1.64156
H -4.79472 0.52997 -1.88818
C -0.25994 2.63706 3.25573
H   0.28353 1.81563 2.75959
H -0.60978 2.28151 4.24209
H   0.45970 3.45632 3.43780
C -2.97207 -0.63461 3.38129
H -2.24766 -1.38551 3.73585
H -3.69965 -0.45000 4.19160
H -2.41619 0.30294 3.21074
C -3.09393 -4.37388 -1.78985
H -3.71464 -4.68583 -2.63792
C -4.99836 1.50310 1.38973
H -4.46777 1.82063 2.30740
H -5.95425 2.06773 1.39963
C -0.17334 2.46861 -3.65744
H   0.41908 3.35939 -3.93221
H -0.51048 1.99947 -4.59910
H   0.48734 1.76142 -3.13182
C -4.67286 -2.91544 2.09666
H -5.36609 -3.17191 1.27845
H -5.25565 -2.87999 3.03378
H -3.94084 -3.73346 2.17736
C -2.20326 4.24467 3.19575
H -1.54566 5.10746 3.40294
H -2.54881 3.85469 4.16943
H -3.08371 4.62297 2.65138
C -2.31812 3.80102 -3.55327
H -3.21640 4.06580 -2.97198
H -2.64850 3.34016 -4.50138
H -1.79690 4.74236 -3.80328
C   0.90913 -3.49631 1.22192
H   1.11177 -4.58087 1.15142
H   1.55771 -3.06657 2.00190
H   1.18886 -3.02624 0.26383
C -3.92676 -1.71569 -3.27402
H -3.79114 -2.57401 -3.95595
H -2.97222 -1.16261 -3.24337
H -4.69197 -1.05272 -3.71596
C -2.14714 -5.25937 -1.26670
H -2.02329 -6.26005 -1.69482
C -0.96425 -3.95957 2.84817
H -1.99177 -3.72585 3.17137
H -0.28006 -3.69064 3.67274
H -0.90166 -5.05352 2.70413
C -5.73946 -2.86121 -1.91738
H -6.49579 -2.14915 -2.29254
H -6.06142 -3.20830 -0.92122
H -5.74821 -3.73549 -2.59196
N   4.32022 1.07658 -0.79933
N   4.76977 -1.03282 -0.53928
C   5.72028 0.97316 -0.76106
C   6.00778 -0.36627 -0.59672
C   3.72600 -0.15020 -0.64847
Cu 1.78687 -0.33495 -0.27824
C   3.49345 2.31327 -0.81710
H   2.46550 1.92250 -0.93526
C   7.35100 -1.02780 -0.53721
H   7.44442 -1.71508 0.32017
H   8.13690 -0.26394 -0.42844
H   7.58009 -1.60371 -1.45182
C   6.67406 2.11770 -0.92104
H   6.69016 2.51083 -1.95320
H   7.69709 1.78821 -0.68067
H   6.43448 2.95854 -0.24920
C   4.52259 -2.46598 -0.24504

H 3.43200 -2.55968 -0.38947
C 3.79039 3.21872 -2.02183
H 4.74896 3.75399 -1.92580
H 2.99248 3.97607 -2.09268
H 3.80041 2.64242 -2.96170
C 3.55374 3.03885 0.53586
H 3.27503 2.34324 1.34407
H 2.82946 3.87018 0.53194
H 4.55508 3.45593 0.74050
C 5.22433 -3.41130 -1.23405
H 5.04796 -3.10310 -2.27774
H 4.81997 -4.42934 -1.10641
H 6.31150 -3.46761 -1.06485
C 4.81756 -2.79117 1.22743
H 5.89070 -2.70074 1.46774
H 4.51239 -3.82869 1.44457
H 4.24387 -2.11525 1.88057
C 1.76538 -0.12945 1.64249
C 2.01640 -0.18107 2.86151
C 2.29543 -0.24757 4.31709
C 3.37522 -1.32649 4.60316
H 3.57859 -1.39006 5.68813
H 3.04227 -2.31973 4.25726
H 4.32066 -1.08137 4.09034
C 2.82295 1.12292 4.82307
H 3.04279 1.07453 5.90560
H 3.74926 1.40296 4.29352
H 2.07982 1.91888 4.65621
C 1.00517 -0.61955 5.09663
H 0.21063 0.12329 4.92143
H 0.62589 -1.60374 4.77353
H 1.20748 -0.66527 6.18272
C 1.25477 -1.38208 -3.31574
C 1.70329 -2.84678 -3.07313
H 0.89689 -3.43458 -2.60465
H 1.96886 -3.32007 -4.03553
H 2.58649 -2.88382 -2.41596
C 0.03178 -1.39067 -4.26918
H -0.33463 -0.36817 -4.45349
H 0.31171 -1.83841 -5.23972
H -0.79126 -1.98328 -3.83823
C 2.41568 -0.58763 -3.95835
H 3.28598 -0.55435 -3.28368
H 2.71796 -1.06825 -4.90566
H 2.11327 0.44879 -4.18307

P_{Bu}
SCF (BP86) Energy = -2499.07895187
Enthalpy 0K = -2497.815323
Enthalpy 298K = -2497.733748
Free Energy 298K = -2497.929942
Lowest Frequency = 3.8397 cm^{-1}
Second Frequency = 23.4100 cm^{-1}
SCF (BP86-D3BJ) Energy = -2499.49533578
SCF (C6H6) Energy = -2499.08466624
SCF (BS2) Energy = -3311.31229168

Cu 1.68314 -0.00228 0.00060
Al -1.40321 0.00054 -0.00049
Si -4.05636 -1.50156 -1.24811
Si -4.05538 1.50955 1.24235
C 1.77535 1.59117 -3.08855
C 1.77127 -1.59880 3.08718
N -2.43091 1.52500 0.48050
N -2.43388 -1.52161 -0.48215
N 4.50541 -1.04810 -0.29382
N 4.50389 1.04567 0.29547
C 0.03175 0.58640 -1.28455
C 0.02979 -0.58849 1.28417
C -1.83018 -2.82377 -0.33002
C -1.82235 2.82516 0.33074
C 1.03658 0.93611 -1.96009
C 3.65855 -0.00190 0.00111
C 1.03374 -0.94079 1.95968
C -0.98719 3.37500 1.35981
C -0.99476 -3.37742 -1.35679
C -2.08312 -3.61675 0.83818
C -0.76921 -2.67457 -2.69871
H -1.17922 -1.65429 -2.60673
C -2.06977 3.61988 -0.83748
C 0.72486 -2.55091 -3.05606
H 1.26865 -1.98215 -2.28499
H 0.84817 -2.02146 -4.01799
H 1.20208 -3.54127 -3.16815
C 5.85176 0.66305 0.18939
C -0.76904 2.67125 2.70250
H -1.18201 1.65234 2.60873
C -5.42493 0.76515 0.12860
H -6.37197 1.05938 0.62935
H -5.42260 1.32425 -0.82693
C 5.85273 -0.66313 -0.18909
C -1.45408 -4.86802 0.98642
H -1.65082 -5.45740 1.89028
C -4.03896 -0.54653 -2.90003
H -3.60152 0.45761 -2.78669
H -5.06603 -0.42538 -3.28881
H -3.44969 -1.08345 -3.66200
C -5.42672 -0.75360 -0.13774
H -6.37334 -1.04519 -0.64084
H -5.42829 -1.31270 0.81778
C -3.03560 3.17123 -1.93462
H -3.51603 2.24754 -1.57069
C 3.98691 -2.38264 -0.69991
H 2.89890 -2.28959 -0.52826
C -0.60710 -5.38561 0.00061
H -0.13499 -6.36608 0.12879
C -0.38727 4.63689 1.17052
H 0.23944 5.05185 1.96944
C -4.65126 3.29015 1.59498
H -3.86107 3.94583 1.99150
H -5.47187 3.25459 2.33343
H -5.04785 3.76594 0.68287
C -0.40064 -4.64176 -1.16569
H 0.22628 -5.05958 -1.96296
C -4.65604 -3.28042 -1.60275
H -3.86740 -3.93688 -2.00106
H -5.47730 -3.24222 -2.34035
H -5.05250 -3.75698 -0.69099
C -1.43524 4.86859 -0.98374
H -1.62777 5.45933 -1.88760
C -0.58800 5.38204 0.00403
H -0.11149 6.36057 -0.12271
C 3.98347 2.37874 0.70398
H 2.89540 2.28409 0.53349
C -3.04861 -3.16314 1.93351
H -3.52497 -2.23802 1.56790
C -4.14233 4.21325 -2.21265
H -3.72879 5.13960 -2.65021
H -4.68150 4.49489 -1.29324
H -4.87756 3.81049 -2.93187
C -1.51224 -3.39698 -3.84833
H -1.14180 -4.43101 -3.97048
H -1.35451 -2.86879 -4.80623
H -2.59733 -3.45264 -3.66765
C 0.72300 2.54270 3.06646
H 1.20302 3.53144 3.18084
H 1.26847 1.97208 2.29798
H 0.84013 2.01293 4.02899
C 3.24044 1.12080 -3.22146
H 3.72770 1.66112 -4.05233
H 3.28688 0.04279 -3.44622
H 3.81365 1.30067 -2.29995
C -2.29506 2.82659 -3.24362
H -3.00101 2.48509 -4.02199
H -1.55778 2.02696 -3.06786
H -1.75971 3.70853 -3.64082
C -4.04565 0.55523 2.89476
H -3.61165 -0.45059 2.78331
H -5.07428 0.43813 3.28065
H -3.45665 1.09044 3.65814
C 3.23594 -1.12793 3.22312
H 3.28127 -0.05058 3.45141
H 3.81018 -1.30431 2.30158
H 3.72276 -1.67054 4.05275

C 7.03545 -1.55305 -0.42785
H 6.98307 -2.07394 -1.39800
H 7.15110 -2.32073 0.35685
H 7.95695 -0.95107 -0.43573
C -2.30795 -2.81982 3.24279
H -3.01331 -2.47537 4.02042
H -1.56801 -2.02269 3.06683
H -1.77565 -3.70313 3.64103
C 7.03312 1.55519 0.42665
H 6.98135 2.07549 1.39715
H 7.14585 2.32348 -0.35786
H 7.95582 0.95504 0.43266
C 4.51188 3.52461 -0.17725
H 5.54695 3.80382 0.07695
H 3.88314 4.41561 -0.01546
H 4.46877 3.26994 -1.24751
C -1.51459 3.39614 3.84896
H -1.36289 2.86733 4.80750
H -2.59866 3.45571 3.66355
H -1.14098 4.42884 3.97277
C 4.21687 2.65537 2.19784
H 3.80631 1.84695 2.82051
H 3.70829 3.59209 2.47781
H 5.28729 2.77263 2.43531
C 4.21892 -2.66134 -2.19360
H 5.28927 -2.77655 -2.43238
H 3.80556 -1.85507 -2.81717
H 3.71213 -3.59975 -2.47119
C 1.73349 -3.13337 2.85211
H 2.23168 -3.65485 3.68960
H 2.24833 -3.40397 1.91688
H 0.69654 -3.49489 2.77374
C 4.51840 -3.52617 0.18255
H 3.89162 -4.41882 0.02231
H 4.47536 -3.27015 1.25248
H 5.55393 -3.80341 -0.07192
C -4.15982 -4.20038 2.21143
H -3.75058 -5.12805 2.65021
H -4.69931 -4.48056 1.29174
H -4.89409 -3.79394 2.92957
C 1.73685 3.12641 -2.85801
H 2.25027 3.39989 -1.92286
H 0.69975 3.48792 -2.78212
H 2.23605 3.64552 -3.69636
C 1.03642 -1.26823 4.41610
H 1.03398 -0.18261 4.60527
H 1.55260 -1.76626 5.25652
H -0.00667 -1.61700 4.39574
C 1.04210 1.25646 -4.41734
H 1.55855 1.75286 -5.25855
H -0.00140 1.60416 -4.39887
H 1.04095 0.17036 -4.60375

TS(Z)$_{Bu}$

SCF (BP86) Energy = -2500.20203910
Enthalpy 0K = -2498.922996
Enthalpy 298K = -2498.841294
Free Energy 298K = -2499.037967
Lowest Frequency = -537.2026 cm^{-1}
Second Frequency = 11.0225 cm^{-1}
SCF (BP86-D3BJ) Energy = -2500.62457061
SCF (C6H6) Energy = -2500.21667501
SCF (BS2) Energy = -3312.43579972

Cu 2.05224 -0.13710 0.07833
Si -3.70164 -1.45598 1.96271
Al -1.43337 0.03532 0.11985
Si -3.77072 2.29985 0.71989
N 4.87186 -1.23292 -0.37153
N -2.68514 -1.35478 0.48800
N 4.80367 0.93799 -0.28513
N -2.09244 1.82082 0.25789
C 4.00753 -0.17761 -0.18335
H -0.73567 -0.20444 -1.45554
C -1.09424 -3.87785 1.05264
H -1.02388 -2.90417 1.56762
C -1.97755 -3.67480 -0.18414
C -1.25803 2.96578 -0.04092
C -3.54388 -2.41428 -1.60539
C -4.31832 1.44926 2.34878
H -3.47552 1.47808 3.06537
H -5.08875 2.12340 2.77720
C -4.89097 0.02015 2.22479
H -5.66552 -0.01933 1.43595
H -5.42486 -0.25389 3.16013
C -2.72396 -2.47192 -0.42855
C -5.14877 1.96207 -0.57113
H -4.79484 2.06785 -1.60764
H -5.95401 2.70316 -0.41711
H -5.59925 0.96353 -0.46996
C -2.84989 -4.67301 -2.25296
H -2.89854 -5.51770 -2.94876
C 4.23558 2.30637 -0.14180
H 3.19033 2.10649 0.15199
C -1.27955 3.57866 -1.34126
C -4.85116 -2.98327 1.90065
H -5.83656 -2.70228 1.49113
H -5.01752 -3.37427 2.91966
H -4.45457 -3.79998 1.27872
C 4.40180 -2.64009 -0.30373
H 3.33596 -2.52697 -0.03224
C -3.57782 -3.50447 -2.49576
H -4.21194 -3.43975 -3.38808
C -2.09516 3.02421 -2.51242
H -2.51745 2.05786 -2.18590
C 6.18411 -0.78441 -0.60014
C 1.23834 -0.53424 1.94812
C 0.33969 -4.31993 0.68284
H 0.34107 -5.24709 0.08322
H 0.92699 -4.52179 1.59493
H 0.86042 -3.53993 0.10636
C 6.13830 0.59393 -0.54549
C -1.70599 -4.91101 2.02959
H -2.71132 -4.61775 2.36888
H -1.06620 -5.02864 2.92341
H -1.79267 -5.90291 1.55038
C -2.06934 -4.74733 -1.09608
H -1.50942 -5.66704 -0.88914
C -4.46422 -1.22938 -1.88624
H -4.23183 -0.46802 -1.12583
C 0.27688 -0.24094 1.17117
C -0.44736 3.57262 0.98053
C 5.09800 -3.43441 0.81505
H 4.56086 -4.38442 0.97295
H 5.09280 -2.87635 1.76491
H 6.14041 -3.68571 0.56106
C -0.31774 2.99971 2.39724
H -0.70373 1.96632 2.36814
C -0.54257 4.75701 -1.57288
H -0.57979 5.21665 -2.56739
C -2.62633 -1.50887 3.54167
H -2.00374 -2.41690 3.59217
H -3.26350 -1.47951 4.44353
H -1.95499 -0.63424 3.57774
C -4.24867 -0.59309 -3.27479
H -4.43392 -1.31254 -4.09267
H -3.22164 -0.20963 -3.38801
H -4.94352 0.25326 -3.41887
C 7.27219 1.56323 -0.69252
H 7.02776 2.39419 -1.37332
H 8.15200 1.04829 -1.10881
H 7.57784 2.00215 0.27366
C 1.15708 2.94623 2.85030
H 1.77043 2.36640 2.14139
H 1.23725 2.47036 3.84320
H 1.59680 3.95457 2.94736
C -3.86281 4.19179 0.98255
H -4.87512 4.43557 1.35235
H -3.71354 4.73532 0.03555
H -3.13124 4.58947 1.69998
C -1.21876 2.77262 -3.76055
H -0.85008 3.72072 -4.19211
H -1.80781 2.26518 -4.54542
H -0.34389 2.15030 -3.51822

C 4.90013 3.09988 0.99462
H 5.91606 3.44021 0.73730
H 4.95096 2.50354 1.92028
H 4.29455 3.99753 1.20070
C -3.25821 3.96839 -2.90125
H -3.93867 4.16680 -2.05854
H -3.85397 3.53277 -3.72363
H -2.86994 4.94163 -3.25197
C 4.47679 -3.35525 -1.66345
H 5.51467 -3.57916 -1.96004
H 4.00731 -2.75371 -2.45647
H 3.93874 -4.31562 -1.59858
C -5.94635 -1.63868 -1.71555
H -6.61169 -0.76608 -1.84529
H -6.13430 -2.07082 -0.71870
H -6.23819 -2.39643 -2.46495
C -1.13483 3.78683 3.44942
H -0.85277 4.85532 3.45298
H -0.94469 3.38658 4.46177
H -2.21794 3.72344 3.26629
C 4.19720 3.05723 -1.48101
H 3.65378 4.00697 -1.34407
H 3.64886 2.45900 -2.22591
H 5.20423 3.29773 -1.86158
C 0.26132 4.75743 0.69264
H 0.84902 5.23001 1.48789
C 7.37972 -1.65930 -0.82667
H 7.69329 -2.19424 0.08721
H 8.23339 -1.04257 -1.14793
H 7.21021 -2.41281 -1.61297
C 0.21861 5.36012 -0.56840
H 0.76684 6.28858 -0.76277
C 0.71752 0.11051 -2.03270
C 1.35761 1.15109 -1.61049
H 1.01476 1.97877 -0.98513
C 0.84035 -0.98037 -3.07681
C -0.32653 -0.88944 -4.08883
H -1.29041 -1.06394 -3.58564
H -0.35796 0.09312 -4.58593
H -0.19996 -1.66699 -4.86258
C 0.81134 -2.37512 -2.42056
H 0.90211 -3.16117 -3.19073
H 1.64743 -2.49019 -1.70945
H -0.13291 -2.53848 -1.87685
C 2.18131 -0.76637 -3.82402
H 2.18592 0.20371 -4.34637
H 3.02698 -0.76758 -3.11723
H 2.32780 -1.57088 -4.56786
C 1.93757 -0.92217 3.22262
C 3.29711 -0.19755 3.37486
H 3.96895 -0.44190 2.53632
H 3.16365 0.89595 3.39902
H 3.77903 -0.50722 4.31920
C 1.02669 -0.56259 4.42645
H 1.52876 -0.84610 5.36881
H 0.81461 0.51725 4.45988
H 0.06721 -1.09955 4.36993
C 2.17583 -2.45300 3.23466
H 1.21934 -2.99769 3.20101
H 2.70780 -2.74155 4.15893
H 2.78165 -2.76982 2.37109

Z_{Bu}

SCF (BP86) Energy = -2500.28228281
Enthalpy 0K = -2498.995965
Enthalpy 298K = -2498.914463
Free Energy 298K = -2499.110972
Lowest Frequency = 11.1875 cm^{-1}
Second Frequency = 18.1785 cm^{-1}
SCF (BP86-D3BJ) Energy = -2500.70635428
SCF (C6H6) Energy = -2500.27938922
SCF (BS2) Energy = -3312.51359339

Cu 1.79900 -0.14937 0.28795
Si -3.52727 -1.72154 1.78180
Al -1.28733 0.03255 0.18327
Si -3.90246 2.06688 0.69324
N 4.71179 -0.92084 -0.26983
N -2.42531 -1.49081 0.37841
N 4.28669 1.19417 -0.54749
N -2.17523 1.75658 0.29623
C 3.70082 0.00847 -0.17043
H 1.64771 0.96179 -2.58416
C -0.67285 -3.85761 1.12916
H -0.77033 -2.89036 1.65044
C -1.49074 -3.76530 -0.16388
C -1.41248 2.94385 -0.01242
C -3.04169 -2.67249 -1.73066
C -4.46129 1.07832 2.23295
H -3.68120 1.14285 3.01518
H -5.32465 1.65062 2.63186
C -4.88064 -0.38457 1.97571
H -5.55756 -0.44474 1.10286
H -5.48047 -0.76327 2.83105
C -2.30342 -2.62928 -0.50124
C -5.18577 1.65860 -0.66958
H -5.07836 2.31075 -1.54998
H -6.19061 1.84179 -0.24455
H -5.15962 0.61522 -1.01411
C -2.12384 -4.88004 -2.26015
H -2.05186 -5.74201 -2.93237
C 3.52599 2.47255 -0.49261
H 2.49428 2.13714 -0.27783
C -1.42511 3.51579 -1.33323
C -4.52337 -3.33871 1.58670
H -5.33990 -3.19350 0.85868
H -4.98438 -3.60635 2.55385
H -3.92423 -4.19339 1.24008
C 4.50647 -2.33482 0.14141
H 3.44015 -2.35081 0.43041
C -2.91991 -3.77961 -2.59187
H -3.48577 -3.78515 -3.53139
C -2.28558 2.95655 -2.46772
H -2.75388 2.03043 -2.09381
C 5.90658 -0.32803 -0.71625
C 1.17266 -0.38852 2.32420
C 0.82934 -4.08119 0.84567
H 0.99992 -4.97955 0.22643
H 1.38458 -4.21688 1.78964
H 1.26052 -3.21607 0.31639
C 5.63530 1.01469 -0.88728
C -1.18263 -4.98004 2.06474
H -2.23839 -4.84072 2.34457
H -0.58713 -5.00957 2.99562
H -1.09336 -5.97082 1.58392
C -1.43218 -4.86447 -1.04505
H -0.81864 -5.73088 -0.76949
C -4.01721 -1.56319 -2.10920
H -4.01270 -0.86158 -1.25988
C 0.16549 -0.17772 1.58675
C -0.64063 3.61564 0.99929
C 5.34459 -2.69903 1.38007
H 5.00420 -3.67019 1.77642
H 5.23221 -1.94577 2.17551
H 6.41632 -2.80113 1.14362
C -0.60942 3.16115 2.46149
H -1.05178 2.15075 2.49865
C -0.66701 4.67061 -1.61078
H -0.69434 5.08955 -2.62380
C -2.58504 -1.71585 3.44777
H -1.94191 -2.60216 3.57020
H -3.30007 -1.69343 4.28950
H -1.94312 -0.82186 3.52323
C -3.57683 -0.78337 -3.36496
H -3.50193 -1.44568 -4.24670
H -2.59429 -0.30702 -3.21180
H -4.30452 0.01247 -3.60500
C 6.55222 2.09514 -1.37478
H 6.29169 2.43736 -2.39155
H 7.58710 1.72119 -1.40957
H 6.54696 2.97842 -0.71522
C 0.82827 3.08168 3.01382
H 1.44836 2.40071 2.41043
H 0.81798 2.70996 4.05364

H	1.31557	4.07282	3.03714
C	-4.21502	3.92647	1.05844
H	-5.16673	4.22753	0.58658
H	-3.42405	4.58896	0.67436
H	-4.31881	4.11443	2.14029
C	-1.46440	2.59991	-3.72576
H	-0.98770	3.49393	-4.16727
H	-2.11897	2.16095	-4.49946
H	-0.67412	1.87033	-3.49158
C	3.98068	3.35424	0.68140
H	4.98989	3.77157	0.52544
H	3.97736	2.78407	1.62378
H	3.27799	4.19671	0.78710
C	-3.40410	3.95684	-2.84844
H	-4.00449	4.25769	-1.97439
H	-4.08350	3.51729	-3.60094
H	-2.97614	4.87731	-3.28518
C	4.70447	-3.33559	-1.00859
H	5.76211	-3.42770	-1.30454
H	4.11269	-3.05423	-1.89268
H	4.36709	-4.33172	-0.67727
C	-5.45675	-2.09786	-2.28377
H	-6.16182	-1.26406	-2.45219
H	-5.79215	-2.65560	-1.39326
H	-5.53580	-2.77809	-3.15077
C	-1.44191	4.09473	3.37193
H	-1.03674	5.12290	3.35589
H	-1.41493	3.73945	4.41789
H	-2.49325	4.14664	3.05592
C	3.50581	3.22625	-1.83180
H	2.70427	3.98239	-1.79522
H	3.29249	2.54461	-2.67111
H	4.45418	3.74974	-2.03531
C	0.08919	4.77647	0.66829
H	0.64971	5.29137	1.45749
C	7.18821	-1.05335	-0.99429
H	7.55207	-1.62801	-0.12659
H	7.97413	-0.32804	-1.25534
H	7.09777	-1.75254	-1.84384
C	0.09449	5.30741	-0.62603
H	0.65919	6.21816	-0.85540
C	1.02303	0.11642	-2.23842
C	0.13779	0.38237	-1.21980
H	0.26014	1.45915	-0.95913
C	1.21833	-1.09077	-3.15607
C	0.30513	-0.88920	-4.39713
H	0.49124	0.08423	-4.88429
H	0.50120	-1.68286	-5.14117
H	-0.75801	-0.93639	-4.11616
C	0.87633	-2.42415	-2.48153
H	0.96748	-3.26048	-3.19675
H	1.56348	-2.62053	-1.64086
H	-0.15047	-2.42955	-2.08633
C	2.68718	-1.10211	-3.64606
H	2.92750	-0.18037	-4.20730
H	3.39062	-1.16988	-2.79930
H	2.86669	-1.95933	-4.31917
C	1.94838	-0.67264	3.57501
C	3.24607	0.16952	3.64042
H	3.88265	-0.02120	2.76054
H	3.01727	1.24708	3.66862
H	3.81399	-0.08530	4.55276
C	1.04702	-0.33661	4.79437
H	1.58926	-0.55893	5.73091
H	0.76536	0.72754	4.80042
H	0.12301	-0.93673	4.77536
C	2.30758	-2.17863	3.63907
H	1.39579	-2.79697	3.64785
H	2.87942	-2.38896	4.56062
H	2.91844	-2.48108	2.77458

References

1. H. -Y. Liu, R. J. Schwamm, M. S. Hill, M. F. Mahon, C. L. McMullin, and N. A. Rajabi, *Angew. Chem. Int. Ed.* **2021**, *60*, 14390-14393.

2. O. V. Dolomanov, L. J. Bourhis, R. J. Gildea, J. A. K. Howard, H. Puschmann, H. *J. Appl. Cryst.* **2009**, *42*, 339-341.

3. G. M. Sheldrick, *Acta Cryst*. **2015**, A71, 3-8.

4. G. M. Sheldrick, *Acta Cryst*. **2015**, C71, 3-8.

5. Gaussian 16, Revision C.01, M. J. Frisch, G. W. Trucks, H. B. Schlegel, G. E. Scuseria, M. A. Robb, J. R. Cheeseman, G. Scalmani, V. Barone, G. A. Petersson, H. Nakatsuji, X. Li, M. Caricato, A. V. Marenich, J. Bloino, B. G. Janesko, R. Gomperts, B. Mennucci, H. P. Hratchian, J. V. Ortiz, A. F. Izmaylov, J. L. Sonnenberg, D. Williams-Young, F. Ding, F. Lipparini, F. Egidi, J. Goings, B. Peng, A. Petrone, T. Henderson, D. Ranasinghe, V. G. Zakrzewski, J. Gao, N. Rega, G. Zheng, W. Liang, M. Hada, M. Ehara, K. Toyota, R. Fukuda, J. Hasegawa, M. Ishida, T. Nakajima, Y. Honda, O. Kitao, H. Nakai, T. Vreven, K. Throssell, J. A. Montgomery, Jr., J. E. Peralta, F. Ogliaro, M. J. Bearpark, J. J. Heyd, E. N. Brothers, K. N. Kudin, V. N. Staroverov, T. A. Keith, R. Kobayashi, J. Normand, K. Raghavachari, A. P. Rendell, J. C. Burant, S. S. Iyengar, J. Tomasi, M. Cossi, J. M. Millam, M. Klene, C. Adamo, R. Cammi, J. W. Ochterski, R. L. Martin, K. Morokuma, O. Farkas, J. B. Foresman, and D. J. Fox, Gaussian, Inc., Wallingford CT, 2016.

6. D. Andrae, U. Häußermann, M. Dolg, H. Stoll, H. Preuß, H., Energy-adjusted ab initio pseudopotentials for the second and third row transition elements. *Theoretica chimica acta* 1990**,** **77** (2), 123-141.

7. W. J. Hehre, R. Ditchfield, J. A. Pople, Self—Consistent Molecular Orbital Methods. XII. Further Extensions of Gaussian—Type Basis Sets for Use in Molecular Orbital Studies of Organic Molecules. *The Journal of Chemical Physics* 1972, **56** (5), 2257-2261.

8. P. C. Hariharan, J. A. Pople, The influence of polarization functions on molecular orbital hydrogenation energies. *Theoretica chimica acta* 1973, **28** (3), 213-222.

9. A. Höllwarth, M. Böhme, S. Dapprich, A. W. Ehlers, A. Gobbi, V. Jonas, K. F. Köhler, R. Stegmann, A. Veldkamp, G. Frenking, G., A set of d-polarization functions for pseudo-potential basis sets of the main group elements Al-Bi and f-type polarization functions for Zn, Cd, Hg. *Chemical Physics Letters* 1993, **208** (3), 237-240.

10. A. D. Becke, Density-functional exchange-energy approximation with correct asymptotic behavior. *Physical Review A* 1988, **38** (6), 3098-3100.

11. J. P. Perdew, Density-functional approximation for the correlation energy of the inhomogeneous electron gas. *Physical Review B* 1986, **33** (12), 8822-8824.

12. D. Figgen, G. Rauhut, M. Dolg, H. Stoll, Energy-consistent pseudopotentials for group 11 and 12 atoms: adjustment to multi-configuration Dirac–Hartree–Fock data. *Chemical Physics* 2005, **311** (1), 227-244.

13. K. A. Peterson, C. Puzzarini, Systematically convergent basis sets for transition metals. II. Pseudopotential-based correlation consistent basis sets for the group 11 (Cu, Ag, Au) and 12 (Zn, Cd, Hg) elements. *Theoretical Chemistry Accounts* 2005, **114** (4), 283-296.

14. B. Mennucci, Polarizable continuum model. *Wiley Interdisciplinary Reviews: Computational Molecular Science* 2012, **2** (3), 386-404.

15. S. Grimme, S. Ehrlich, L. Goerigk, L., Effect of the damping function in dispersion corrected density functional theory. *Journal of Computational Chemistry* 2011, **32** (7), 1456-1465.
